# Design, Synthesis, and Structure–Activity Relationships of Substituted Phenyl Cyclobutylureas as Potential Modulators of Inflammatory Responses

**DOI:** 10.3390/ph19030355

**Published:** 2026-02-25

**Authors:** Atziri Corin Chavez Alvarez, Antoine Carpentier, Ahmed Sahli, Martin Perreault, Aichatou Diallo Ngon, Emmanuel Moreau

**Affiliations:** 1 UMR INSERM 1240 IMoST, Université Clermont Auvergne, 58 rue Montalembert, 63000 Clermont-Ferrand, France; aichatou.diallo_ngon@etu.uca.fr; 2 Centre de Recherche du CHU de Québec-Université Laval, Centre Hospitalier de l’Université Laval, 2705 Boul Laurier, Québec, QC G1V 0E8, Canada; antoine.carpentier.1@ulaval.ca (A.C.); ahmed.sahli.1@ulaval.ca (A.S.); martin.perreault.2@ulaval.ca (M.P.)

**Keywords:** substituted phenyl cyclobutylureas, medicinal chemistry, structure–activity relationships (SAR), preliminary biological evaluation

## Abstract

**Background/Objectives**: Chronic inflammation underlies many immune-mediated conditions, yet current anti-inflammatory therapies are often limited by incomplete efficacy or safety concerns. Small molecules inspired by soluble epoxide hydrolase (sEH) inhibitors represent a promising scaffold for early-stage exploration. This study describes the design, synthesis, and preliminary biological evaluation of three series of arylurea derivatives (ACBUs) to establish structure–activity relationships and guide chemical optimization. **Methods**: The compounds were assessed for effects on keratinocyte proliferation, human sEH activity, and the expression of selected inflammatory markers using IL-17A/TNF-α-stimulated HaCaT cells, a relevant in vitro model for preliminary anti-inflammatory profiling. **Results**: A total of 23 novel ACBU derivatives were synthesized and evaluated. Most compounds showed low antiproliferative activity, allowing selection based on cytotoxicity and solubility. Compounds **4b**, **10b**, and **16b** consistently displayed the most favorable profiles in these preliminary assays. Docking studies provided structural rationales supporting the observed trends and guided further optimization within the series. **Conclusions:** Compound **4b** emerged as the most active candidate in preliminary screening, serving as a reference for ongoing SAR studies. These results highlight the potential of the arylurea scaffold for further chemical optimization and demonstrate the value of early-stage biological profiling in guiding our further studies.

## 1. Introduction

Inflammation is a triggered adaptive response that is initiated by harmful and foreign stimuli, notably wounds, irritants, and pathogens. However, under certain circumstances, such as persistent infection or immune-mediated inflammatory diseases (IMIDs), the inflammatory response may become chronic and deleterious [1]. IMIDs comprise a large and heterogeneous group of chronic disorders characterized by dysregulated immune responses affecting multiple organs, either simultaneously or sequentially [2]. Despite their diversity, IMIDs most frequently involve barrier and interface tissues, including the gastrointestinal tract, such as Crohn’s disease and ulcerative colitis (the two main forms of inflammatory bowel disease), the joints (e.g., rheumatoid arthritis, psoriatic arthritis, and axial spondylarthritis), and the skin (e.g., psoriasis) [3,4,5]. IMIDs affect approximately 6% of the Western population, but their prevalence has increased in Asia and South America in recent years. These diseases are chronic and often debilitating, resulting in a heavy burden in terms of direct healthcare costs and indirect social costs, including lost productivity and long-term disability. Beyond their economic impact, IMIDs profoundly affect patients’ quality of life [6,7,8].

Historically, pharmacological management of IMIDs relied primarily on non-steroidal anti-inflammatory drugs (NSAIDs, e.g., acetaminophen, ibuprofen and naproxen), glucocorticoids (e.g., dexamethasone and prednisone), and antileukotrienes (e.g., montelukast and zileuton). Although these agents provide rapid symptomatic relief, they rarely modify the underlying disease mechanisms, and their long-term use, particularly of systemic glucocorticoids, is associated with substantial toxicity. The advent of biological therapies targeting key inflammatory cytokines, such as tumor necrosis factor (TNF), and interleukins, or their receptors, as well as immune-selective anti-inflammatory derivatives (ImSAIDs, e.g., tripeptide FEG (phenylalanine–glutamine–glycine), has drastically improved disease control [9,10,11]. More recently, targeted synthetic antirheumatic drugs, particularly Janus kinase (JAK) inhibitors, have further expanded the therapeutic arsenal by modulating intracellular signaling downstream of multiple cytokine receptors [12,13]. Despite these major advances, IMIDs remain incurable; treatment focuses mainly on disease control and symptom management. Current challenges include long-term safety concerns, variable patient responses, and persistent residual symptoms, all of which underscore the need for continued therapeutic innovation [1].

**Figure 1:**
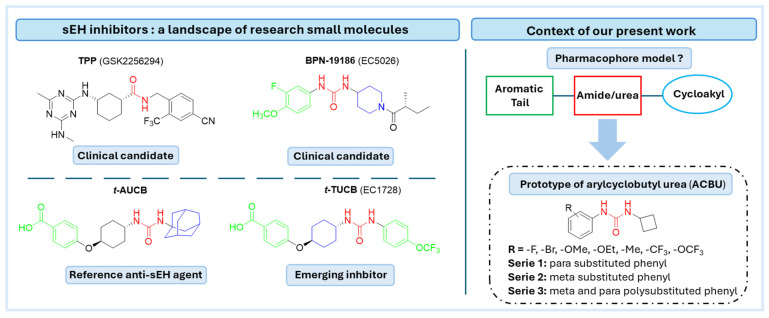
Context of the development of the new ACBUs series.

In this context, there has been growing interest in strategies that seek to restore endogenous mechanisms for resolving inflammation, rather than simply suppressing inflammatory mediators. Lipid mediator metabolism has emerged as a complementary regulatory axis in inflammation. In particular, soluble epoxide hydrolase (sEH), degrades epoxyeicosatrienoic acids (EETs), endogenous anti-inflammatory lipid mediators, into their corresponding dihydroxyeicosatrienoic acids (DHETs), which display markedly weaker biological activity. Inhibition of sEH increases EETs levels, reducing the activation of pro-inflammatory pathways, including NF-κB, and STAT3, and decreasing the production of key cytokines such as IL-6 and IL-23 [14]. Consequently, inhibition of IL-6 production should be interpreted as a functional downstream readout of sEH modulation rather than a direct measure of the enzymatic target. It should be emphasized that IL-6 inhibition does not necessarily imply strong she-related activity, but rather reflects the ability of a compound to effectively engage this signaling axis in a cellular context. Structurally, several sEH inhibitors (sEHi) have progressed to early clinical trials (Figure 1) [15,16,17].

Among these, GSK2256294 has completed Phase I clinical trials in humans, showing sustained, dose-dependent inhibition of sEH activity, along with a favorable safety and tolerability profile in healthy volunteers [18]. Its therapeutic potential has been explored in conditions involving endothelial dysfunction, metabolic and pulmonary inflammation, and neuroinflammation. Other examples include EC5026 (BPN-19186), an orally active and potent sEHi that has completed Phase 1a and 1b studies for pain and inflammation, and EC1728, which remains in preclinical development due to formulation and pharmacokinetic challenges [19]. Collectively, these studies highlight both the feasibility of sEH inhibition as an anti-inflammatory strategy and the need for novel derivatives with improved potency, selectivity, and pharmacokinetic properties. Notably, sEHi reduces IL-6 production indirectly by modulating upstream pro-inflammatory pathways, providing a rationale for designing molecules that may impact targeting IL-6-mediated inflammation.

Molecular docking studies of sEH inhibitors (sEHi) have delineated key structural features that underpin the rational design of potent and selective compounds. In general, sEHi are designed to mimic key features of the transition state geometry associated with the enzymatic hydrolysis of EETs, within the sEH active site [20]. This mimicry is primarily conferred by a polar pharmacophore, most commonly a urea, amide, or carbamate motif, which represents the core determinant of biological activity. Importantly, this functional group establishes a characteristic hydrogen bonding network with the catalytic triad of sEH, notably involving Asp335 (or Asp333), and the tyrosines Tyr383 and Tyr466 [20]. The carbonyl oxygen interacts with both tyrosines, whereas the amino group serves as a hydrogen bond donor toward the aspartate [21]. The active site of sEH, located at the bottom of a long hydrophobic tunnel, requires the presence of lipophilic groups to facilitate effective ligand insertion. Spacers such as phenylene, benzyl, or cyclohexyl moieties enable precise positioning of the pharmacophore within the active site and maintain optimal alignment with the catalytic triad of residues [20,21]. Inhibitor potency is heavily influenced by the nature and position of adjacent aromatic substituents. In general, lipophilic and electron-donating groups, particularly in the para position, can help the binding pose and favor an optimal orientation of the pharmacophore, whereas the ortho- or meta- positions may introduce steric constraints that compromise productive interactions within the active site. Affinity can be further enhanced by introducing bulky hydrophobic motifs, such as adamantyl, cyclohexyl, or substituted phenyl rings, that efficiently occupy the enzymatic tunnel. In addition, halogenation, especially fluorination, improves both inhibitory potency and metabolic stability, as fluorine may also form halogen-bond interactions with specific residues lining the binding pocket [22].

These design principles have formed the basis for developing ACBU-type inhibitors, which are engineered to mimic the transition state while optimizing both the occupancy of the hydrophobic tunnel in sEH and the physicochemical properties necessary for systemic administration. Importantly, in the present work, structural modifications were intentionally introduced to explore whether fine-tuning these interactions could translate into enhanced modulation of IL-6-driven inflammatory responses, beyond classical sEH inhibition. Given the inflammatory relevance of sEH modulation, psoriasis represents an appropriate IMID model in which to explore this therapeutic potential. These chronic immune-mediated inflammatory skin disorders are characterized by keratinocyte hyperproliferation and a cytokine-driven inflammatory loop dominated by IL-17, IL-23, and IL-6 [23,24]. Beyond its cutaneous manifestations, psoriasis is now recognized as a systemic disease associated with multiple comorbidities, including psoriatic arthritis, cardiovascular disease, and metabolic disorders, reflecting its shared immunopathological mechanisms with other IMIDs [25]. Keratinocytes serve as a major source of IL-6, which plays a central role in amplifying and sustaining psoriatic inflammation. This well-characterized cytokine network thus provides a relevant framework for evaluating compounds targeting IL-6-mediated pathways.

Building on these considerations, the present study aimed at the design and synthesis of three series of potential small-molecule inhibitors (Figure 1, ACBUs). Structural modifications were introduced with the goal of enhancing IL-6 inhibitory activity while minimizing cytotoxicity and maintaining favorable physicochemical properties toward sEH. The biological profiles of the synthesized ACBUs were explored using in vitro assays, including cytotoxicity measurements, preliminary assessment of pro-inflammatory marker expression, sEH inhibition assays, and docking studies, to support ongoing SAR investigations.

## 2. Results and Discussion

### 2.1. Design and Synthesis of ACBUs Derivatives

In sEHi, the urea moiety constitutes the central pharmacophore and was therefore conserved in this preliminary study. Structural modifications were instead focused on the aryl tail and the cycloalkyl substituent to explore structure–activity relationships. The appended cycloalkyl group is known to occupy a hydrophobic pocket of the enzyme and plays a key role in modulating both potency and metabolic stability. In this context, a cyclobutyl substituent was selected based on its recognized ability to favorably influence pharmacokinetic properties when used as a replacement for other cycloalkyl groups. Compared with cyclopropyl substituents, cyclobutyl rings exhibit reduced ring strain, while maintaining lower conformational flexibility than larger cyclohexyl or adamantyl groups [26,27]. This balance was expected to enhance metabolic stability while preserving favorable hydrophobic interactions within the binding pocket, thereby maintaining target engagement. To investigate preliminary structure–activity relationships within our ACBU series, a focused set of substituents was introduced at the 3-, 4-, and 5-positions of the phenyl ring (Figure 1). These substituents have been selected to systematically modulate the electronic properties (electron-donating vs. electron-withdrawing groups), the steric bulkiness, and the lipophilicity, while maintaining limited structural complexity. Halogens (F, Br), alkyl and alkoxy groups (CH_3_, OMe, OEt), fluorinated motifs (CF_3_, OCF_3_, OCHF_2_), and a polar nitrile group were selected to probe the tolerance of the aryl tail region, and to evaluate their impact on both biological potency and physicochemical properties. Substitutions were preferentially introduced at meta and para positions to minimize conformational perturbations of the urea moiety.

**Scheme 1:**
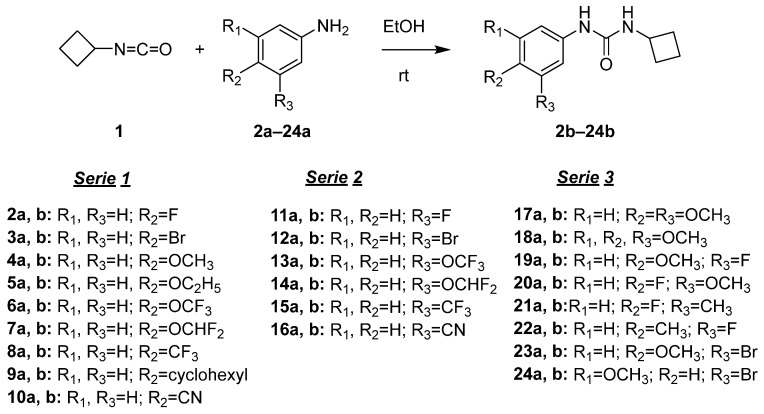
Synthesis of substituted phenylcyclobutylureas (ACBUs) **2b–24b**.

In the present work, three series of ACBUs were prepared in low to excellent yields by the nucleophilic addition of a selected aniline **2a–24a** (1.0 eq.) to isocyanatocyclobutane **1** (1.2 eq.) in ethanol (Scheme 1). The new compounds **2b–24b** were characterized by ^1^H, ^13^C NMR, IR, MS analysis, and their purity was confirmed to be higher than 95% by UHPLC-UV (See Supplemental Materials).

### 2.2. Evaluation of the Antiproliferative Activity in HaCaT Cells

HaCaT keratinocytes were selected as an in vitro model for preliminary evaluation of the synthesized compounds because they represent a well-characterized human keratinocyte line that retains key functional features, including proliferative capacity. This model allowed us to assess cytotoxicity and early biological profiles of the compounds in a controlled cellular setting. The, the antiproliferative activity (IC_50_) of ACBUs **2b–24b** was evaluated using the 3-(4,5-dimethylthiazol-2-yl)-2,5-diphenyltetrazolium bromide (MTT) cell viability assay [28]. The results are shown in Table 1, expressed as the IC_50_. Most of the compounds exhibited very low antiproliferative activity (IC_50_ > 100 µM). However, ACBUs **5b**, **8b**, **14b** and **18b** were discarded due to their antiproliferative activities that could potentially lead to cell death, 15, 15, 12 and 12 µM, respectively. Moreover, ACBUs **2b**, **3b**, **12b** and **22b** were also discarded based on their poor solubility in the used culture medium (>10 µM). Indeed, when no significant effect was observed at this maximal soluble concentration (10 µM), the activity was therefore reported as >10 µM.

Of the 23 synthesized ACBU compounds, the 15 remaining compounds retained acceptable solubility and low cytotoxicity in this cellular model, supporting the selection of the cyclobutyl moiety as a balanced cycloalkyl substituent, providing favorable physicochemical properties and efficient hydrophobic pocket occupancy.

### 2.3. Evaluation of the Antiproliferative Activity in HDFn Cells

Human dermal fibroblasts (HDFn) were selected as a complementary in vitro model to further validate the biological effects of the selected ACBUs, addressing potential limitations associated with the use of HaCaT keratinocytes alone. Unlike HaCaT cells, which harbor mutations in the p53 tumor suppressor gene that may confer partial resistance to apoptosis, HDFn cells possess an intact p53 signaling pathway, representing a non-transformed, apoptosis-competent cellular system. The inclusion of HDFn cells allowed us to assess whether the observed antiproliferative effects were dependent on p53 status, and to distinguish between general cytotoxicity and selective antiproliferative activity. Cell viability was evaluated using the MTT assay under the same experimental conditions applied to HaCaT cells, and IC_50_ values were calculated accordingly. Compounds exhibiting limited solubility in the culture medium (**2b**, **3b**, **12b**, and **22b**) were excluded from this assay. As shown in Table 2 below, most compounds displayed minimal antiproliferative effects in HDFn cells (IC_50_ > 100 µM), confirming a favorable safety profile in non-tumoral cells. Notably, IC_50_ values were generally higher in HDFn compared to HaCaT cells, indicating lower antiproliferative sensitivity in this apoptosis-competent model. Among all tested compounds, ACBU **9b** was the only derivative that showed measurable antiproliferative activity in HDFn cells (IC_50_ = 71 µM) while remaining inactive in HaCaT cells (IC_50_ > 100 µM). Compounds showing pronounced antiproliferative activity were considered unsuitable for further development due to their potential to induce non-specific cytotoxicity in healthy dermal cells.

### 2.4. Evaluation of IL-6 Production Inhibition

**Figure 2:**
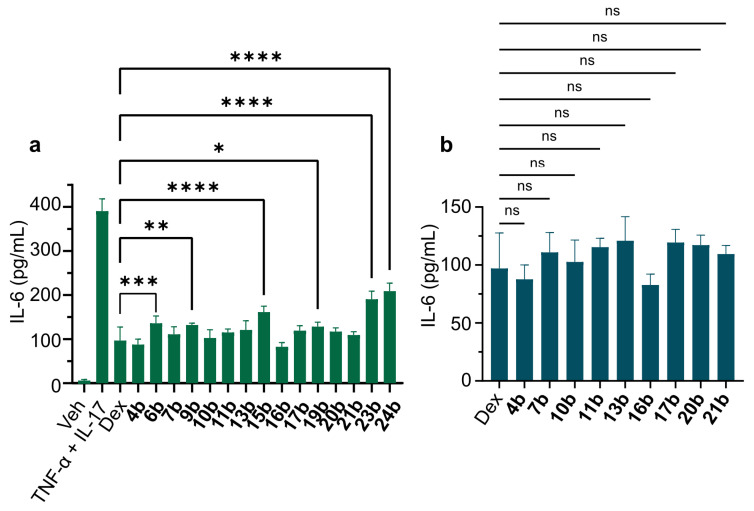
Inhibition of IL-6 production by (**a**) all soluble and non-antiproliferative ACBUs (10 µM), and dexamethasone (Dex, 1 µM) in HaCaT cells stimulated with TNF-α and IL-17A and (**b**) ACBUs (10 µM) that exhibit similar activity to dexamethasone (Dex, 1 µM) in the same cell model. ns: non-significant. One way ANOVA with Dunnett’s multiple comparisons test against dexamethasone were performed (* *p*  <  0.05, ** *p*  <  0.01, *** *p*  <  0.001, **** *p*  <  0.0001).

The inhibitory effects of ACBUs on the production of the inflammatory mediator IL-6 were subsequently evaluated. To assess their impact on IL-6 levels, HaCaT cells were stimulated by the addition of IL-17A and TNFα, which are two well-established pro-inflammatory cytokines in the culture medium, following the protocol described by Chiricozzi [29]. Afterward, an ELISA assay using a commercially available kit was performed to quantify the level of extracellular IL-6 produced by the stimulated cells in the presence or absence of the selected drugs. As shown in Figure 2, dexamethasone (dex,1 µM) was used as a positive control, and it reduced the levels of IL-6 by 75%. All disubstituted analogs (series 3), including para/meta-substituted derivatives **17b**, **19b**, **20b**, **21b**, **23b**, and **24b**, exhibited markedly lower IL-6 inhibition than dexamethasone. Collectively, these data indicate that introducing two aromatic substituents is generally detrimental to IL-6 modulation, potentially due to increased steric hindrance and/or altered physicochemical properties that impair cellular activity. Consistent with this trend, the meta-substituted derivatives (series 2) also displayed limited inhibitory effects on IL-6 production. In particular, compounds bearing fluorinated substituents such as F, OCF_3_, or CF_3_ (**11b**, **13b**, and **15b**) displayed only weak activity, consistent with previous reports indicating that meta-substitution can negatively affect biological performance. In contrast, compound **16b**, bearing a meta-cyano substituent, displayed IL-6 inhibition comparable to dexamethasone, although the difference did not reach statistical significance. Within the para-substituted series (series 1), fluorinated derivatives (CF_3_, OCHF_2_) and the cyclohexyl analogues (**6b**, **7b**, and **9b**) also showed IL-6 inhibition clearly below that of the reference compound, indicating that increased lipophilicity alone is insufficient to improve the anti-inflammatory profile in this scaffold. Notably, compounds **4b** (para-methoxy), and **10b** (para-cyano; the positional isomer of **16b**), showed a trend toward higher IL-6 inhibition, although the effects remained modest and did not reach statistical significance. Overall, these results highlight that IL-6 inhibition across these series is strongly influenced by both the nature and the position of the aromatic substituent. Monosubstituted para-analogues bearing small polar groups appear to be better tolerated, whereas bulky fluorinated substituents or disubstitution patterns are generally detrimental to activity.

### 2.5. Evaluation of Cell Death via Annexin v/Propidium Iodide (PI) Staining

**Figure 3:**
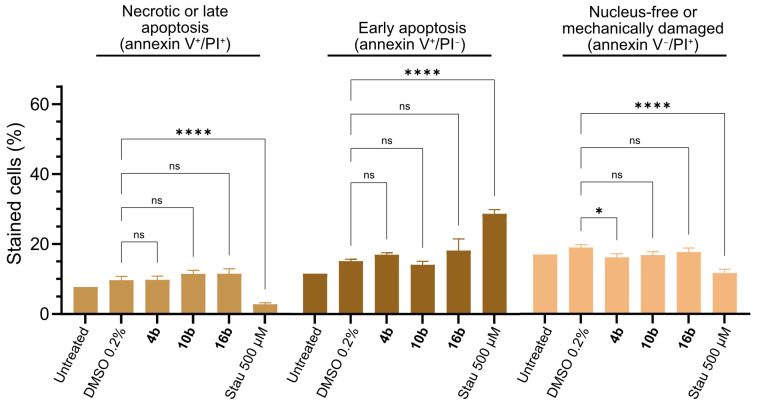
Annexin V/propidium iodide (PI) staining of cells to determine their cell death status (% of stained cells) after treatment with ACBUs **4b**, **10b** and **16b** (100 µM) and staurosporine (stau, 500 nM) after a 48 h treatment. Untreated and cells treated with DMSO 0.2% were used as negative controls (ns: non-significant, * *p*  <  0.05, **** *p*  <  0.0001).

The Annexin V/propidium iodide (PI) assay was performed to evaluate the apoptotic profile induced by the tested compounds, allowing discrimination between viable cells, late apoptosis/necrosis, early apoptosis, and mechanical damage after 48 h of treatment. This experiment was performed to ensure the safety of the utilization of the compounds for further analysis and to avoid interference caused by cell death. Under the evaluated conditions, ACBUs **4b**, **10b** and **16b** at their maximal solubilities (100 µM), none of the compounds induced late apoptosis or late apoptosis/necrosis. ACBU **4b** showed a slight increase in the PI-positive/Annexin V-negative quadrant, suggesting minor mechanical membrane damage that might be attributed to the scraping procedure of the methodology. In contrast, treatment with the positive control staurosporine (500 nM) resulted in a marked accumulation of cells in early apoptosis, confirming the validity and sensitivity of the assay (Figure 3).

### 2.6. Flow Cytometry Analysis of THP1-Derived M1 Macrophage-Activation Markers iNOS, CD86, and CD163

The analysis of macrophage activation markers by flow cytometry in PMA-differentiated THP-1 cells stimulated with LPS, IFNγ, and TNFα was used to assess preliminary effects of ACBU compounds on macrophage activation, without implying direct mechanistic conclusions. This experimental model induces a robust pro-inflammatory response and classical macrophage activation, characterized by the upregulation of iNOS and co-stimulatory molecules. Consequently, variations in the expression of iNOS, CD86, and CD163 under these conditions provide a preliminary readout of potential trends in macrophage activation, rather than evidence of direct modulation of specific signaling pathways. Treatment with ACBU **4b** markedly altered the activation profile of PMA-differentiated THP-1 macrophages stimulated with LPS, IFNγ, and TNFα. Flow cytometry analyses showed a reduction in iNOS and CD86 expression, two hallmarks of classical M1 activation, together with increased CD163 expression and decreased LAP levels. These changes indicate a shift toward an M2-like or regulatory macrophage phenotype. In contrast to compound **4b**, treatment with compound **10b** did not induce significant changes in the expression of iNOS, CD86, or CD163 in PMA-differentiated THP-1 macrophages, while a selective reduction in LAP levels was observed. This profile suggests that **10b** does not substantially interfere with classical or alternative macrophage activation programs, although subtle effects cannot be excluded at this stage. Similarly, compound **16b** induced a decrease in iNOS and LAP expression, without affecting the surface levels of CD86 or CD163. This pattern indicates a partial attenuation of pro-inflammatory effector functions, particularly nitric oxide production, without a concomitant shift toward an M2-like or regulatory macrophage phenotype. Such effects are interpreted as preliminary trends in selective modulation of macrophage activation, which may involve signaling changes, without claiming direct involvement of specific pathways. As expected, dexamethasone, used as an anti-inflammatory control, markedly reduced iNOS and LAP expression while significantly increasing CD163 levels, with no detectable changes in CD86 expression. This response is consistent with its well-established role in promoting a regulatory macrophage phenotype, further validating the phenotypic markers used to assess macrophage polarization in this experimental system [30,31,32].

**Figure 4:**
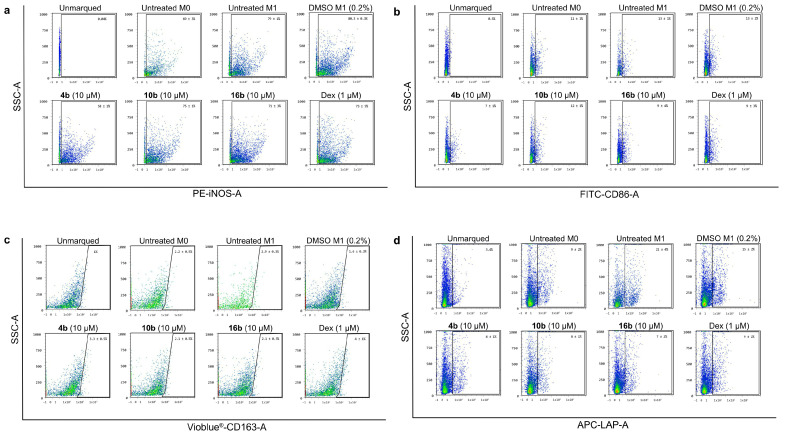
Representative scatter plots for (**a**) iNOS^+^, (**b**) CD86^+^, (**c**) CD163^+^ and (**d**) LAP^+^ cell populations on THP-1 M1-polarized macrophages after 24 h treatment with the selected ACBUs **4b**, **10b** and **16b** (10 µM). THP-1 M1-polarized macrophages treated with dexamethasone (1 µM) were used as positive control while untreated THP-1 M0-polarized macrophages, untreated THP-1 M1-polarized macrophages and THP-1 M1-polarized macrophages treated with DMSO (0.2 %) were used as negative controls. M0: PMA stimulated THP-1 M0-polarized macrophages. M1: PMA, LPS, IFNγ, and TNFα-stimulated THP-1 M1-polarized macrophages. Dex: Dexamethasone.

**Figure 5:**
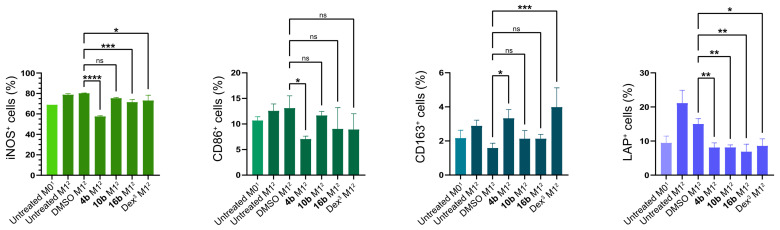
Histograms summarizing the effect of selected ACBUs **4b**, **10b** and **16b** (10 µM) on THP-1 M1-polarized macrophages of the expression of iNOS, CD86, CD163 and LAP after 24 h treatment. THP-1 M1-polarized macrophages treated with dexamethasone (1 µM) were used as positive control while untreated THP-1 M0-polarized macrophages, untreated THP-1 M1-polarized macrophages and THP-1 M1-polarized macrophages treated with DMSO (0.2 %) were used as negative controls. ^1^ M0: PMA stimulated THP-1 M0-polarized macrophages. ^2^ M1: PMA, LPS, IFNγ, and TNFα-stimulated THP-1 M1-polarized macrophages. ^3^ Dex: Dexamethasone. ns: non-significant. * *p* <  0.05, ** *p*  <  0.01, *** *p*  <  0.001, **** *p*  <  0.0001.

Overall, these findings reflect preliminary trends in macrophage activation markers, where classical pro-inflammatory features, such as iNOS and co-stimulatory molecule expression, are typically associated with STAT1-driven programs, and regulatory markers like CD163 are often linked to STAT3-related pathways. Overall, these data provide an early indication that ACBU derivatives may influence macrophage activation states under inflammatory stimulation, although no direct inference about specific signaling mechanisms can be drawn at this stage (Figure 4 and Figure 5) [33].

### 2.7. Evaluation of sEH Inhibition

**Figure 6:**
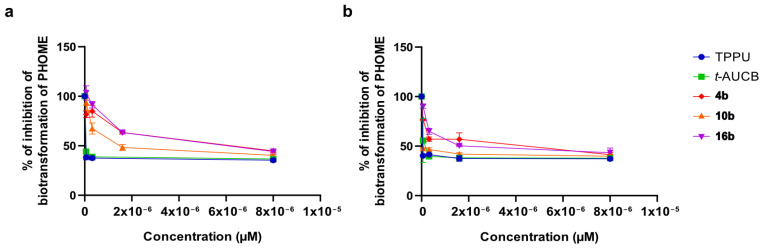
Inhibition of biotransformation of sEH known substrate PHOME (% of inhibition) of ACBUs **4b**, **10b** and **16b** in (**a**) human and (**b**) mouse sEH.

To determine the ability of the selected ACBUs to inhibit sEH, a fluorometric enzymatic assay was performed using the synthetic substrate of sEH (3-phenyl-oxiranyl)-acetic acid cyano-(6-methoxy-naphthalen-2-yl)-methyl ester (PHOME) together with human and murine recombinant sEHs (Figure 6) [34]. Hydrolysis of the PHOME by sEH generates the highly fluorescent substrate 6-methoxy-2-naphthaldehyde that was monitored using spectrofluorimetry. 1-Trifluoromethoxyphenyl-3-(1-propionylpiperidin-4-yl) urea (TPPU) and *trans*-4-[4-(3-adamantan-1-yl-ureido)-cyclohexyloxy]-benzoic acid (*t*-AUCB), which are known as very potent sEH inhibitors were used as positive controls [35,36]. The three selected ACBUs displayed moderate inhibition of sEH at 250 nM (10–85%), remaining less potent than the well-established sEH inhibitors TPPU and *t*-AUCB used as positive controls (Table 3). Compounds **4b**, **10b** and **16b** exhibited relatively potent inhibition of both the murine (64, 70, and 85%, respectively) and the human sEHs (76, 70, and 64%, respectively). However, none outperformed TPPU or *t*-AUCB in terms of inhibition, and we therefore did not pursue the sEH inhibitor route further.

### 2.8. Docking Results and Binding Preference

Although the compounds were initially designed to inhibit sEH through engagement of the EH catalytic triad, no significant inhibition of sEH activity was experimentally observed (Table 3). To better understand this apparent discrepancy, molecular docking studies were conducted on both the C-terminal EH domain and the N-terminal phosphatase domain of sEH.

Molecular docking analysis indicates that compounds **4b**, **10b**, and **16b** exhibit moderate binding affinity towards the sEH hydrolase domain. As summarized in Table S1, the predicted binding free energies range from −5.8 to −7.0 kcal/mol. Specifically, compound **10b** displayed the most favorable score (−7.0 kcal/mol), whereas **4b** exhibited the least favorable interaction energy (−5.8 kcal/mol). In sharp contrast, the potent reference inhibitors *t*-AUCB and TPPU yielded significantly lower energies of −10.3 and −9.5 kcal/mol, respectively.

To quantify the magnitude of this energetic gap, theoretical affinity ratios were derived from the ΔΔG values. Relative to the benchmark inhibitor TPPU, the synthesized compounds are predicted to bind significantly less tightly, with estimated affinity reductions of approximately 68-fold for **10b**, 221-fold for **16b**, and 513-fold for **4b**. The contrast is even more pronounced when compared to the highly potent *t*-AUCB, where the theoretical binding preference shifts by factors ranging from ~260-fold (for **10b**) to nearly 2000-fold (for **4b**) (Table S2).

**Figure 7:**
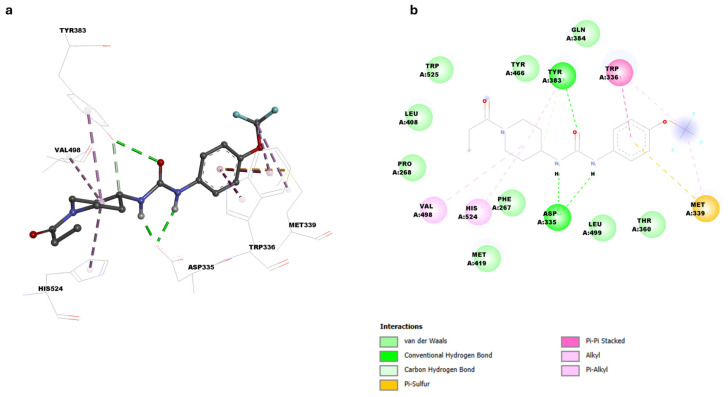
Predicted binding mode of the reference inhibitor **TPPU** within the active site of human soluble Epoxide Hydrolase (sEH) (PDB: 3I28). (**a**) 3D surface representation showing the ligand occupancy in the hydrophobic tunnel. (**b**) 2D interaction map highlighting the critical hydrogen-bond network (green dashed lines) with the catalytic triad residues Asp335, Tyr383, and Tyr466, as well as key hydrophobic interactions (pink/purple spheres) with Phe267, Val498, and Met339.

**Figure 8:**
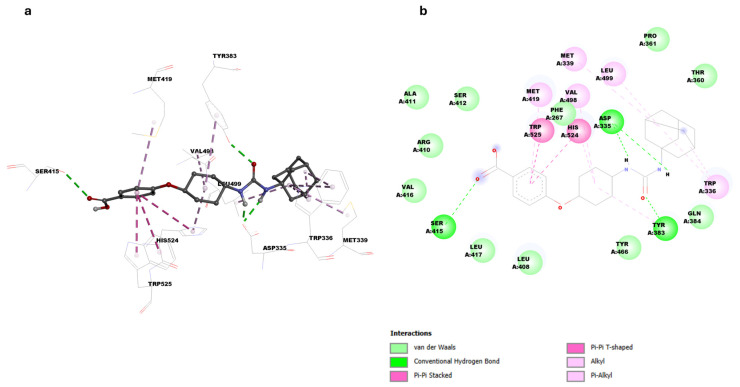
Molecular docking pose of the reference inhibitor ***t*-AUCB** in the sEH catalytic pocket. (**a**) 3D visualization of the inhibitor fit. (**b**) 2D diagram detailing the stabilizing interactions; the urea pharmacophore forms strong hydrogen bonds with Asp335, Tyr383, and Tyr466, while the adamantyl group engages in extensive hydrophobic contacts with residues such as Trp336, Val498, and Leu499.

**Figure 9:**
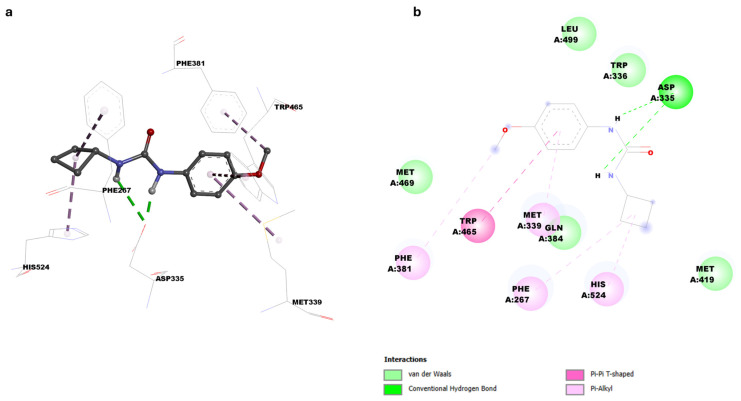
Docking pose and interaction profile of compound **4b** in the sEH active site. (**a**) 3D view of the best-ranked conformation (−5.8 kcal/mol). (**b**) 2D interaction diagram showing a predominance of non-specific hydrophobic interactions (Pi-Alkyl, Alkyl) with Phe267, Phe381, and Met469. Note the absence of the canonical hydrogen-bond triad observed for the reference inhibitors.

**Figure 10:**
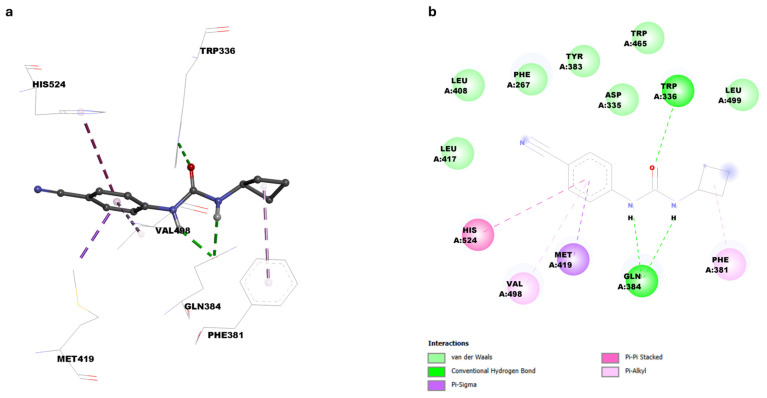
Predicted binding mode of compound **10b** in the sEH hydrolase domain. (**a**) 3D visualization of the ligand orientation. (**b**) 2D interaction map for the top-ranked pose (−7.0 kcal/mol), illustrating hydrophobic contacts with Val498 and Trp336. The binding is primarily driven by Van der Waals forces rather than the specific polar anchoring required for high-affinity inhibition.

**Figure 11:**
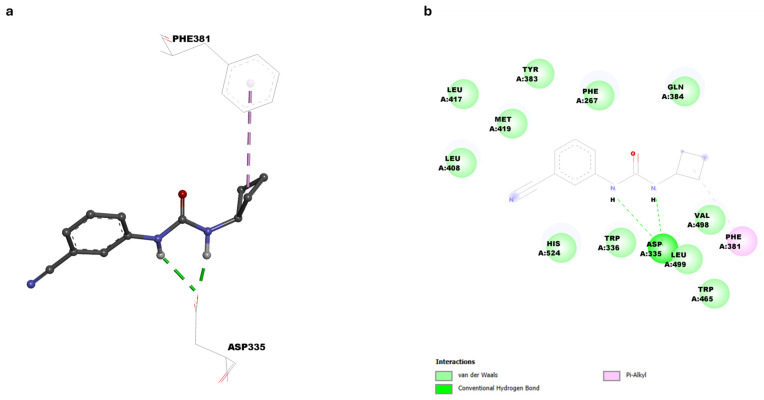
Molecular modeling results for compound **16b** within the sEH active site. (**a**) 3D representation of the docked ligand. (**b**) 2D diagram summarizing the intermolecular forces; the binding is supported by hydrophobic interactions with residues such as Phe267 and Met339, consistent with the moderate affinity score (−6.3 kcal/mol) and micromolar inhibitory potency.

Visual inspection of the binding modes (Figure 7, Figure 8, Figure 9, Figure 10 and Figure 11) elucidates the structural basis for this energetic difference. High-affinity inhibitors typically anchor via a specific hydrogen-bond network involving the catalytic triad residues Tyr383, Tyr466, and Asp335 [37,38]. In contrast, the docking poses of **4b**, **10b**, and **16b** reveal that steric constraints likely prevent the optimal engagement of these residues. Their binding is driven primarily by non-specific hydrophobic interactions with Phe267, Val498, and Trp336, resulting in a weaker “lock-and-key” fit compared to the reference standards.

The molecular docking results provide a structural rationale for the biological screening data presented in Table 3. Experimentally, compounds **4b**, **10b**, and **16b** inhibited human sEH activity with IC_50_ values in the low micromolar range (2.2–5.6 µM). This potency is markedly lower than that of benchmark urea inhibitors, which typically function in the low-nanomolar range [38,39].

A strong qualitative concordance is observed between the in silico and in vitro rankings. Compound **10b** (Table 3, IC_50_ = 2.2 µM) exhibited the most favorable docking score (−7.0 kcal/mol), whereas **4b** was consistently identified as the weakest congener in both evaluations. From a thermodynamic perspective, the calculated binding energies are compatible with dissociation constants in the micromolar range. This contrasts with the scores lower than −9.0 kcal/mol typically required for nanomolar affinity [40]. Structurally, this modest activity is attributed to the lack of a robust hydrogen-bond network with the catalytic tyrosines. Importantly, the docking simulations successfully predicted the potency trend. While the absolute magnitude of the fold-differences varies, which is a common discrepancy between empirical scoring functions and kinetic parameters, the directional accuracy is robust. Most notably, the analysis correctly identified compound **4b** as the least favorable analog of the series (−5.8 kcal/mol), aligning perfectly with the experimental outcome where it exhibited the weakest inhibitory potency (Table 3, highest IC_50_ of 5.6 µM). This concordance confirms that the docking protocol provides a reliable structural basis for rationalizing the limited efficacy of these derivatives. Consequently, the observed trends in cellular assays cannot be fully explained by sEH inhibition alone, highlighting the need for further studies to explore the structural features influencing biological profiles.

### 2.9. Evaluation of Key Inflammatory Genes Expression IL-6 and IL-23

Subsequently, we evaluated the effects of ACBUs **4b**, **10b**, and **16b**, which demonstrated the strongest inhibitory activity on IL-6 production, on the expression of key inflammatory markers. Experiments were performed in HaCaT cells stimulated with TNFα and IL-17A. The mRNA expression levels of IL-6 and IL-23, two pivotal pro-inflammatory genes, were quantified by real-time polymerase chain reaction (qPCR). Dexamethasone (1 μM) was employed as a positive control. The results of the qPCR analyses are summarized in Table 4. ACBUs **4b**, **10b**, and **16b** reduced IL-6 expression by 24%, 29%, and 17%, respectively, and inhibited IL-23 expression by 40%, 50%, and 21%, respectively.

Overall, the observed decreases in IL-6 and IL-23 mRNA levels highlight trends in compound activity and can guide further optimization in the SAR study, without implying confirmed anti-inflammatory effects.

### 2.10. Evaluation of the Biotransformation of ACBUs by Microsomes

Finally, we have determined the metabolic stability of two compounds **4b** and **10b** in the presence of liver microsomes. We exposed them to human liver microsomes for 1 h to assess their half-life (t_1/2_) in vitro. Half-lives colligated in Table 5 show that compounds have half-life of 180 and 420 min. In addition, compounds **4b** and **10b** exhibited half-lives ranging from 121 min to 257 min in both human and murine skin microsomes fractions, suggesting limited metabolic degradation.

## 3. Materials and Methods

### 3.1. Chemistry

#### 3.1.1. Material and Instrumentation

All chemicals were supplied either by Sigma-Aldrich Canada (Oakville, ON, Canada), VWR International (Mont-Royal, QC, Canada), Enamine LLC (Princetown, NJ, USA), Oakwood Chemical (Estill, SC, USA) or Combi-Blocks (San Diego, CA, USA) and used as received unless specified otherwise. Liquid flash chromatography was performed on silica gel F60, 60 Å, 40–63 μm supplied by Silicycle (Québec City, QC, Canada) using an FPX flash purification system (Biotage, Charlottesville, VA, USA) and using solvent mixtures expressed as volume/volume ratios. Progression of the chemical reaction was monitored by TLC using precoated silica gel 60 F254 TLC plates (VWR International, Mont-Royal, QC, Canada). The chromatograms and spots were visualized under UV light at 254 nm. IR spectra were recorded on an FT-IR spectrophotometer Nicolet iS10 (Thermofisher scientific, Villebon-sur-Yvette, France). ^1^H and ^13^C NMR spectra were recorded on a Bruker AM-300 spectrometer (Bruker, Bremen, Germany). Chemical shifts (δ) are reported in parts per million (ppm). Exact mass analyses were performed on an Agilent 1200 HPLC (Agilent Inc, Mississauga, ON, Canada) coupled to an Exactive mass spectrometer (Thermo Fisher Scientific Inc., Mississauga, ON, Canada) using a reverse phase Kinetex column 2.6 µm C18 100 Å, 150 mm × 4.6 mm (Phenomenex, Torrance, CA, USA). Melting points were determined using an OptiMelt MPA100 melting point apparatus (Stanford Research Systems, Sunnyvale, CA, USA). The purity of the final compounds was >95% as assessed by UHPLC-UV at a wavelength of 280 nm Waters ACQUITY Arc system equipped with 2998 PDA detector (Waters, Mississauga, ON, Canada). The compounds were solubilized in DMSO and eluted using MeOH/H_2_O linear gradient (1.0 mL/min) on a CORTECS C18 reversed phase column 3.0 mm × 50 mm × 2.7 µM equipped with CORTECS C18 VanGuard Cartridges 2.1 mm × 5 mm × 2.7 µM.

#### 3.1.2. General Synthesis of ACBUs

The appropriate aniline **2a–24a** (0.35 mmol, 1.0 eq.) was dissolved in ethanol (2 mL) and cyclobutyl isocyanate **1** (1.2 eq.) was added. The reaction mixture was stirred at room temperature for 72 h. Afterward, the reaction mixture was evaporated to dryness under reduced pressure. The residue was purified by trituration in diethyl ether followed by flash chromatography on silica gel using hexane/ethyl acetate (80:20, *v*:*v*) as eluent.

##### 1-Cyclobutyl-3-(4-fluorophenyl)urea (**2b**)

Compound **2b** was synthesized from cyclobutyl isocyanate **1** and 4-fluoroaniline **2a** according to the general protocol described above. Yield: 99%; White solid; m.p.: 207–211 °C; IR (ν, cm^−1^) 3311, 2972, 1626, 1601, 1569, 1500, 1241, 1208, 1150, 1091, 835; ^1^H NMR (300 MHz, DMSO-d_6_): δ 8.31 (brs, 1H, NH), 7.37–7.31 (m, 2H, Ar), 7.05–6.98 (m, 2H, Ar), 6.35 (d, *J* = 8.1 Hz, 1H, NH), 4.10 (hex, *J* = 8.2 Hz, 1H, CH), 2.21–2.11 (m, 2H, CH_2_), 1.88–1.74 (m, 2H, CH_2_), 1.64–1.50 (m, 2H, CH_2_). ^13^C NMR (75 MHz, DMSO-d_6_): δ 158.9, 155.8, 154.6, 137.2, 137.2, 119.8, 119.7, 115.6, 115.3, 44.9, 31.5, 14.8. HRMS-(ESI) *m*/*z*, calculated for [C_11_H_14_FN_2_O]^+^ ([M + H]^+^): 209.1090; found: 209.1081 (See Supplemental Materials).

##### 1-(4-Bromophenyl)-3-cyclobutylurea (**3b**)

Compound **3b** was synthesized from cyclobutyl isocyanate **1** and 4-bromoaniline **3a** according to the general protocol described above. Yield: 71%; White solid; m.p.: 204–208 °C; IR (ν, cm^−1^) 3297, 2968, 1639, 1543, 1477, 1402, 1299, 1258, 1240, 1068, 868; ^1^H NMR (300 MHz, DMSO-d_6_): δ 8.44 (brs, 1H, NH), 7.35 (s, 4H, Ar), 6.43 (d, *J* = 8.1 Hz, 1H, NH), 4.11 (hex, *J* = 8.1 Hz, 1H, CH), 2.21–2.13 (m, 2H, CH_2_), 1.90–1.77 (m, 2H, CH_2_), 1.64–1.52 (m, 2H, CH_2_).^13^C NMR (75 MHz, DMSO-d_6_): δ 153.9, 139.9, 131.3, 119.6, 112.3, 44.5, 31.0, 14.4. HRMS-(ESI) *m*/*z*, calculated for [C_11_H_14_BrN_2_O]^+^ (M + H]^+^): 269.0290; found: 269.0279 (See Supplemental Materials).

##### 1-Cyclobutyl-3-(4-methoxyphenyl)urea (**4b**)

Compound **4b** was synthesized from cyclobutyl isocyanate **1** and 4-methoxyaniline **4a** according to the general protocol described above. Yield: 99%; White solid; m.p.: 155–158 °C; IR (ν, cm^−1^) 3337, 2969, 1633, 1556, 1505, 1240, 1181, 1071, 826; ^1^H NMR (300 MHz, DMSO-d_6_): δ 8.07 (brs, 1H, NH), 7.25 (d, *J* = 7.3 Hz, 2H, Ar), 6.78 (d, *J* = 7.3 Hz, 2H, Ar), 6.25 (d, *J* = 8.1 Hz, 1H, NH), 4.10 (hex, *J* = 8.1 Hz, 1H, CH), 3.66 (s, 3H, OCH_3_), 2.23–2.09 (m, 2H, CH_2_), 1.90–1.71 (m, 2H, CH_2_), 1.64–1.51 (m, 2H, CH_2_). ^13^C NMR (75 MHz, DMSO-d_6_): δ 154.8, 154.4, 134.0, 119.9, 114.3, 55.5, 45.0, 31.8, 14.8. HRMS-(ESI) *m*/*z*, calculated for [C_12_H_17_N_2_O_2_]^+^ ([M + H]^+^): 221.1290; found: 221.1280 (See Supplemental Materials).

##### 1-Cyclobutyl-3-(4-ethoxyphenyl)urea (**5b**)

Compound **5b** was synthesized from cyclobutyl isocyanate **1** and 4-ethoxyaniline **5a** according to the general protocol described above. Yield: 26%; White solid; m.p.: 170–174 °C; IR (ν, cm^−1^) 3295, 2968, 1633, 1556, 1505, 1297, 1032, 826; ^1^H NMR (300 MHz, DMSO-d_6_): δ 8.06 (brs, 1H, NH), 7.22 (d, *J* = 8.4 Hz, 2H, Ar), 6.76 (d, *J* = 8.4 Hz, 2H, Ar), 6.25 (d, *J* = 8.1 Hz, 1H, NH), 4.09 (hex, *J* = 8.1 Hz, 1H, CH), 3.92 (q, *J* = 7.0 Hz, 2H, CH_2_), 2.20–2.11 (m, 2H, CH_2_), 1.86–1.71 (m, 2H, CH_2_), 1.64–1.53 (m, 2H, CH_2_), 1.27 (t, *J* = 7.0 Hz, 3H, CH_3_). ^13^C NMR (75 MHz, DMSO-d_6_): δ 154.8, 153.6, 133.9, 119.9, 114.9, 63.5, 44.9, 31.6, 15.2, 14.8. HRMS-(ESI) *m*/*z*, calculated for [C_13_H_19_N_2_O_2_ ([M + H]^+^): 235.1447; found: 235.1437 (See Supplemental Materials).

##### 1-Cyclobutyl-3-(4-(trifluoromethoxy)phenyl)urea (**6b**)

Compound **6b** was synthesized from cyclobutyl isocyanate **1** and 4-trifluoromethoxyaniline **6a** according to the general protocol described above. Yield: 84%; White solid; m.p.: 179–180 °C; IR (ν, cm^−1^) 3312, 2969, 1641, 1608, 1573, 1508, 1246, 1200, 1108; ^1^H NMR (300 MHz, DMSO-d_6_): δ 8.53 (brs, 1H, NH), 7.47 (d, *J* = 8.5 Hz, 2H, Ar), 7.20 (d, *J* = 8.5 Hz, 2H, Ar), 6.46 (d, *J* = 8.0 Hz, 1H, NH), 4.13 (hex, *J* = 8.0 Hz, 1H, CH), 2.29–2.12 (m, 2H, CH_2_), 1.95–1.77 (m, 2H, CH_2_), 1.68–1.50 (m, 2H, CH_2_). ^13^C NMR (75 MHz, DMSO-d_6_): δ 154.4, 142.5, 140.2, 122.0, 119.2, 44.9, 31.4, 14.8. HRMS-(ESI) *m*/*z*, calculated for [C_12_H_14_F_3_N_2_O_2_]^+^ ([M + H]^+^): 275.1007; found: 275.0994 (See Supplemental Materials).

##### 1-Cyclobutyl-3-(4-(difluoromethoxy)phenyl)urea (**7b**)

Compound **7b** was synthesized from cyclobutyl isocyanate **1** and 4- difluoromethoxyaniline **7a** according to the general protocol described above. Yield: 94%; White solid; m.p;: 169–170 °C; IR (ν, cm^−1^) 3299, 2976, 1628, 1601, 1574, 1506, 1214, 1122, 1097; ^1^H NMR (300 MHz, DMSO-d_6_): δ 8.38 (brs, 1H, NH), 7.39 (d, 2H, *J* = 8.4 Hz, Ar) 7.31–6.81 (m, 3H, Ar, CHF_2_), 6.40 (d, *J* = 8.1 Hz, 1H), 4.13 (m, 1H, CH), 2.20–2.14 (m, 2H, CH_2_), 1.90–1.77 (m, 2H, CH_2_), 1.64–1.52 (m, 2H, CH_2_). ^13^C NMR (75 MHz, DMSO-d_6_): δ 154.6, 145.2, 138.4, 120.1, 119.3, 117.1 (t, *J_C_*_–_*_F_* = 255,7), 44.9, 31.5, 14.8. HRMS-(ESI) *m*/*z*, calculated for [C_12_H_15_F_2_N_2_O_2_]^+^ ([M + H]^+^): 257.1102; found: 257.1087 (See Supplemental Materials).

##### 1-Cyclobutyl-3-(4-(trifluoromethyl)phenyl)urea (**8b**)

Compound **8b** was synthesized from cyclobutyl isocyanate **1** and 4- trifluoromethylaniline **8a** according to the general protocol described above. Yield: 92%; White solid; m.p.: 170–182 °C; IR (ν, cm^−1^) 3301, 2969, 1639, 1614, 1568, 1491, 1335, 1247, 1165, 1131, 1107, 700; ^1^H NMR (300 MHz, DMSO-d_6_): δ 8.73 (brs, 1H, NH), 7.58–7.50 (m, 4H, Ar), 6.54 (d, *J* = 8.2 Hz, 1H, NH), 4.12 (hex, *J* = 8.2 Hz, 1H, CH), 2.22–2.13 (m, 2H, CH_2_), 1.88–1.81 (m, 2H, CH_2_), 1.63–1.54 (m, 2H, CH_2_). ^13^C NMR (75 MHz, DMSO-d_6_): δ 154.2, 144.6, 126.9, 126.4 (m), 126.3, 126.3, 123.3, 121.7, 121.2, 117.7 (m), 44.9, 31.3, 14.9. HRMS-(ESI) *m***/***z*, calculated for [C_12_H_14_F_3_N_2_O]^+^ ([M + H]^+^): 259.1058; found: 259.1049 (See Supplemental Materials).

##### 1-Cyclobutyl-3-(4-cyclohexylphenyl)urea (**9b**)

Compound **9b** was synthesized from cyclobutyl isocyanate **1** and 4-cyclohexylaniline **9a** according to the general protocol described above. Yield: 63%; White solid; m.p.: 189–191 °C; IR (ν, cm^−1^) 3317, 2915, 2849, 1631, 1592, 1549, 1512, 1240, 655; ^1^H NMR (300 MHz, DMSO-d_6_): δ 8.14 (brs, 1H, NH), 7.22 (d, *J* = 8.4 Hz, 2H, Ar), 7.01 (d, *J* = 8.4 Hz, 2H, Ar), 6.29 (d, *J* = 8.3 Hz, 1H, NH), 4.08 (hex, *J* = 8.3 Hz, 1H, N-CH), 2.48–2.46 (m, 1H, CH), 2.19–2.05 (m, 2H, 2x CH), 1.82–1.72 (m, 7H, 7x CH), 1.63–1.51 (m, 2H, 2x CH), 1.3340–1.26 (m, 5H, 5x CH). ^13^C NMR (75 MHz, DMSO-d_6_): δ 154.2, 140.4, 138.1, 126.7, 117.9, 44.5, 43.1, 39.1, 34.2, 31.2, 26.5, 25.7, 14.4. HRMS-(ESI) *m***/***z*, calculated for [C_17_H_25_N_2_O]^+^ ([M + H]^+^): 273.1961; found: 273.1957 (See Supplemental Materials).

##### 1-(4-Cyanophenyl)-3-cyclobutylurea (**10b**)

Compound **10b** was synthesized from cyclobutyl isocyanate **1** and 4-cyanoaniline **10a** according to the general protocol described above. Yield: 27%; White solid; m.p.: 170–172 °C; IR (ν, cm^−1^) 3311, 2916, 2850, 2225, 1631, 1556, 1241, 653; ^1^H NMR (300 MHz, DMSO-d_6_): δ 8.87 (brs, 1H, NH), 7.65 (d, *J* = 9.0 Hz, 2H, Ar), 7.55 (d, *J* = 9.0 Hz, 2H, Ar), 6.64 (d, *J* = 8.0 Hz, 1H, NH), 4.13 (hex, *J* = 8.0 Hz, 1H, CH), 2.23–2.15 (m, 2H, CH_2_), 1.93–1.80 (m, 2H, CH_2_), 1.66–1.54 (m, 2H, CH_2_). ^13^C NMR (75 MHz, DMSO-d_6_): δ 153.6, 144.9, 133.2, 119.5, 117.5, 102.4, 44.5, 30.9, 14.5. HRMS-(ESI) *m***/***z*, calculated for [C_12_H_14_N_3_O]^+^ ([M + H]^+^), 216.1131; found: 216.1132 (See Supplemental Materials).

##### 1-Cyclobutyl-3-(3-fluorophenyl)urea (**11b**)

Compound **11b** was synthesized from cyclobutyl isocyanate **1** and 3-fluoroaniline **11a** according to the general protocol described above. Yield: 97%; White solid; m.p.: 133–138 °C; IR (ν, cm^−1^) 3315, 2988, 1632, 1600, 1482, 1440, 1272, 1241, 1141, 859, 786; ^1^H NMR (300 MHz, DMSO-d_6_): δ 8.53 (brs, 1H, NH), 7.42 (d, 1H, *J* = 12.3 Hz, Ar), 7.19 (q, *J* = 7.9 Hz, 1H, Ar), 6.99 (d, *J* = 8.2 Hz, 1H, Ar), 6.65 (td, *J* = 8.5, 2.6 Hz,1H, Ar), 6.45 (d, *J* = 8.0 Hz, 1H, NH), 4.11 (hex, *J* = 8.2 Hz, 1H, CH), 2.22–2.12 (m, 2H, CH_2_), 1.89–1.76 (m, 2H, CH_2_), 1.62–1.53 (m, 2H, CH_2_). ^13^C NMR (75 MHz, DMSO-d_6_): δ 164.5, 161.3, 154.4, 142.9, 142.7, 130.5, 130.4, 113.8 113.8, 107.8, 107.6, 104.9, 104.6, 44.9, 31.4, 14.9. HRMS-(ESI) *m*/*z*, calculated for [C_11_H_14_FN_2_O]^+^ ([M + H]^+^): 209.1090; found: 209.1084 (See Supplemental Materials).

##### 1-(3-Bromophenyl)-3-cyclobutylurea (**12b**)

Compound **12b** was synthesized from cyclobutyl isocyanate **1** and 3-bromoaniline **12a** according to the general protocol described above. Yield: 72%; White solid; m.p.: 177–180 °C; IR (ν, cm^−1^) 3312, 2967, 2852, 1634, 1590, 1543, 1240; ^1^H NMR (300 MHz, DMSO-d_6_): δ 8.46 (brs, 1H, NH), 7.74 (s, 1H, Ar), 7.17–7.12 (m, 2H, Ar), 7.05–6.96 (m, 1H, Ar), 6.43 (d, *J* = 8.0 Hz, 1H, NH), 4.07 (hex, *J* = 8.2 Hz, 1H, CH), 2.21–2.06 (m, 2H, CH_2_), 1.90–1.71 (m, 2H, CH_2_), 1.65–1.44 (m, 2H, CH_2_). ^13^C NMR (75 MHz, DMSO-d_6_): δ 153.9, 142.1, 130.5, 123.5, 121.7, 119.9, 116.4, 44.5, 30.9, 14.5. HRMS-(ESI) *m***/***z*, calculated for [C_11_H_14_BrN_2_O]^+^ ([M + H]^+^): 269.0290; found: 269.0277 (See Supplemental Materials).

##### 1-Cyclobutyl-3-(3-(trifluoromethoxy)phenyl)urea (**13b**)

Compound **13b** was synthesized from cyclobutyl isocyanate **1** and 3-trifluoromethoxyaniline **13a** according to the general protocol described above. Yield: 67%; White solid; m.p.: 137–138 °C; IR (ν, cm^−1^) 3311, 2968, 1641, 1609, 1574, 1508, 1247, 1200; ^1^H NMR (300 MHz, DMSO-d_6_): δ 8.63 (brs, 1H, NH), 7.62 (s, 1H, Ar), 7.29 (t, *J* = 8.1 Hz, 1H, Ar), 7.15 (d, *J* = 8.1 Hz 1H, Ar), 6.82 (d, *J* = 8.1 Hz, 1H, Ar), 6.49 (d, *J* = 8.1 Hz, 1H, NH), 4.10 (hex, *J* = 8.1 Hz, 1H, CH), 2.21–2.13 (m, 2H, CH_2_), 1.90–1.77 (m, 2H, CH_2_), 1.63–1.52 (m, 2H, CH_2_). ^13^C NMR (75 MHz, DMSO-d_6_): δ 154.3, 149.2, 142.7, 130.6, 116.7, 113.3, 110.1, 44.9, 31.3, 14.9. HRMS-(ESI) *m***/***z*, calculated for [C_12_H_14_F_3_N_2_O_2_]^+^ ([M + H]^+^): 275.1007; found: 275.0995 (See Supplemental Materials).

##### 1-Cyclobutyl-3-(3-(difluoromethoxy)phenyl)urea (**14b**)

Compound **14b** was synthesized from cyclobutyl isocyanate **1** and 3-difluoromethoxyaniline **14a** according to the general protocol described above. Yield: 77%; White solid; m.p.: 135–137 °C; IR (ν, cm^−1^) 3311, 2968, 1641, 1609, 1574, 1508, 1247, 1200; ^1^H NMR (300 MHz, DMSO-d_6_): δ 8.51 (brs, 1H, NH), 7.39 (d, *J* = 3.1 Hz 1H, Ar), 7.21 (t, *J* = 8.1 Hz, 1H, Ar), 7.13–6.88 (m, 2H, Ar, CHF2), 6.66 (d, *J* = 8.0 Hz, 1H, Ar), 6.44 (d, *J* = 8.2 Hz, 1H, NH), 4.10 (hex, *J* = 8.2 Hz, 1H, CH), 2.22–2.07 (m, 2H, CH_2_), 1.90–1.76 (m, 2H, CH_2_), 1.63–1.52 (m, 2H, CH_2_). ^13^C NMR (75 MHz, DMSO-d6): δ 154.4, 151.8, 142.5, 130.3, 116.9 (t, *J*_*C*–*F*_ = 255.0 Hz, CHF_2_), 114.7, 111.2, 108.3, 44.9, 31.4, 14.9. HRMS-(ESI) *m***/***z*, calculated for [C_12_H_15_F_2_N_2_O_2_]^+^ ([M + H]^+^): 257.1102; found: 257.1092 (See Supplemental Materials).

##### 1-Cyclobutyl-3-(3-(trifluoromethyl)phenyl)urea (**15b**)

Compound **15b** was synthesized from cyclobutyl isocyanate **1** and 3-trifluoromethylaniline **15a** according to the general protocol described above. Yield: 62%; White solid; m.p.: 162–165 °C; IR (ν, cm^−1^) 3320, 2970, 1639, 1614, 1567, 1491, 1335, 1246, 1165, 1130, 1107, 885; ^1^H NMR (300 MHz, DMSO-d_6_): δ 8.68 (brs, 1H, NH), 7.93 (s, 1H, Ar), 7.47–7.38 (m, 2H, Ar), 7.20–7.17 (m, 1H, Ar), 6.52 (d, *J* = 7.9 Hz, 1H, NH), 4.11 (hex, *J* = 7.9 Hz, 1H, CH), 2.21–2.12 (m, 2H, CH_2_), 1.89–1.81 (m, 2H, CH_2_), 1.64–1.54 (m, 2H, CH_2_). ^13^C NMR (75 MHz, DMSO-d_6_): δ 154.4, 141.7, 130.1 (m), 129.6 (m), 121.6 (m), 117.7 (m), 114.1 (m), 45.0, 31.3, 14.9. HRMS-(ESI) *m***/***z*, calculated for [C_12_H_14_F_3_N_2_O]^+^ ([M + H]^+^): 259.1058; found: 259.1049 (See Supplemental Materials).

##### 1-(3-Cyanophenyl)-3-cyclobutylurea (**16b**)

Compound **16b** was synthesized from cyclobutyl isocyanate **1** and 3-cyanoaniline **16a** according to the general protocol described above. Yield: 67%; White solid; m.p.: 170–173 °C; IR (ν, cm^−1^) 3675, 3309, 2974, 2229, 1641, 10,552, 1428, 1278, 1246; ^1^H NMR (300 MHz, DMSO-d_6_): δ 8.63 (brs, 1H, NH), 7.89–7.86 (m, 1H, Ar), 7.52(d, *J* = 7.7 1H, Ar), 7.36 (t, *J* = 7.7 Hz 1H, Ar), 7.27 (d, *J* = 7.7, 1H, Ar), 6.54 (d, *J* = 8.0 Hz, 1H, NH), 4.09 (hex, *J* = 8.0 Hz, 1H, CH), 2.20–2.09 (m, 2H, CH_2_), 1.88–1.76 (m, 2H, CH_2_), 1.63–1.49 (m, 2H, CH_2_). ^13^C NMR (75 MHz, DMSO-d_6_): δ 153.9, 141.4, 130.0, 124.5, 122.3, 120.2, 119.0, 111.5, 44.5, 30.9, 14.5. HRMS-(ESI) *m***/***z*, calculated for [C_12_H_14_N_3_O]^+^ ([M + H]^+^): 216.1131; found: 216.1132 (See Supplemental Materials).

##### 1-Cyclobutyl-3-(3,4-dimethoxyphenyl)urea (**17b**)

Compound **17b** was synthesized from cyclobutyl isocyanate **1** and 3,4-dimethoxyaniline **17a** according to the general protocol described above. Yield: 86%; White solid; m.p.: 135–137 °C; IR (ν, cm^−1^) 3299, 2971, 1626, 1605, 1566, 1511, 1265, 1232, 1133, 1026; ^1^H NMR (300 MHz, DMSO-d_6_): δ 8.11 (brs, 1H, NH), 7.13 (d, *J* = 2.1, 1H, Ar), 6.82–6.69 (m, 2H, Ar), 6.26 (d, *J* = 8.1 Hz, 1H, NH), 4.09 (hex, *J* = 8.1 Hz, 1H, CH), 3.70–3.63 (m, 6H, 2x OCH_3_), 2.24–2.08 (m, 2H, CH_2_), 1.90–1.71 (m, 2H, CH_2_), 1.65–1.47 (m, 2H, CH_2_). ^13^C NMR (75 MHz, DMSO-d_6_): δ 154.7, 149.2, 143.9, 134.6, 113.0, 110.0, 104.0, 56.3, 55.7, 44.9, 31.6, 14.8. HRMS-(ESI) *m***/***z*, calculated for [C_13_H_19_N_2_O_3_]^+^ ([M + H]^+^): 251.1396; found: 251.1388 (See Supplemental Materials).

##### 1-Cyclobutyl-3-(3,4,5-trimethoxyphenyl)urea (**18b**)

Compound **18b** was synthesized from cyclobutyl isocyanate **1** and 3,4,5-trimethoxyaniline **18a** according to the general protocol described above. Yield: 79%; White solid; m.p.: 165–177 °C; IR (ν, cm^−1^) 3313, 2971, 1636, 1608, 1556, 1505, 1411, 1320; ^1^H NMR (300 MHz, DMSO-d_6_): δ 8.20 (brs, 1H, NH), 6.71–6.70 (s, 2H, Ar), 6.29 (d, *J* = 8.0 Hz, 1H, Ar), 4.09 (hex, *J* = 8.0 Hz, 1H, NH), 3.69 (s, 6H, 2x OCH_3_), 3.57 (s, 3H, OCH_3_), 2.24–2.09 (m, 2H, CH_2_), 1.92–1.70 (m, 2H, CH_2_), 1.69–1.51 (m, 2H, CH_2_). ^13^C NMR (75 MHz, DMSO-d_6_): δ 154.6, 153.2, 137.0, 132.3, 95.9, 60.5, 56.0, 44.9, 31.8, 14.8. HRMS-(ESI) *m***/***z*, calculated for [C_14_H_21_N_2_O_4_]^+^ ([M + H]^+^): 281.1501; found: 281.1494 (See Supplemental Materials).

##### 1-Cyclobutyl-3-(3-fluoro-4-methoxyphenyl)urea (**19b**)

Compound **19b** was synthesized from cyclobutyl isocyanate **1** and 3-fluoro-4-methoxyaniline **19a** according to the general protocol described above. Yield: 77%; White solid; m.p.: 156–159 °C; IR (ν, cm^−1^) 3310, 2966, 1635, 1595, 1566, 1531, 1505, 1433, 1297, 1246, 1124, 1034; ^1^H NMR (300 MHz, DMSO-d_6_): δ 8.27 (brs, 1H, NH), 7.40–7.35 (m, 1H, Ar), 7.01–6.88 (m, 2H, Ar), 6.33 (d, *J* = 8.0 Hz, 1H, NH), 4.09 (hex, *J* = 8.0 Hz, 1H, CH), 3.74 (s, 3H, OCH_3_), 2.19–2.09 (m, 2H, CH_2_), 1.88–1.73 (m, 2H, CH_2_), 1.62–1.46 (m, 2H, CH_2_). ^13^C NMR (75 MHz, DMSO-d6): δ 154.6, 151.6 (d, *J_C_*_–_*_F_* = 239.3 Hz), 141.9 (d, *J_C_*_–_*_F_* = 11.2 Hz), 134.7 (d, *J_C_*_–_*_F_* = 9.8 Hz), 114.8 (d, *J_C_*_–_*_F_* = 3.0 Hz), 113.9 (d, *J_C_*_–_*_F_* = 3.0 Hz), 107.0 (d, *J_C_*_–_*_F_* = 22.0 Hz), 56.7, 44.9, 31.5, 14.8. HRMS-(ESI) *m***/***z*, calculated for [C_12_H_16_FN_2_O_2_]^+^ ([M + H]^+^): 239.1190; found: 239.1187 (See Supplemental Materials).

##### 1-Cyclobutyl-3-(4-fluoro-3-methoxyphenyl)urea (**20b**)

Compound **20b** was synthesized from cyclobutyl isocyanate **1** and 4-fluoro-3-methoxyaniline **20a** according to the general protocol described above. Yield: 71%; White solid; m.p.: 184–186 °C; IR (ν, cm^−1^) 3302, 2970, 1626, 1574, 1518, 1404, 1264, 1205, 1111; ^1^H NMR (300 MHz, DMSO-d_6_): δ 8.31 (brs, 1H, NH), 7.30 (dd, *J* = 8.0, 2.4 Hz 1H, Ar), 7.02–6.95 (m, 1H, Ar), 6.77–6.71 (m, 1H, Ar), 6.34 (d, *J* = 8.0 Hz, 1H, NH), 4.08 (hex, *J* = 8.0 Hz, 1H, CH), 3.74 (s, 3H, OCH_3_), 2.19–2.10 (m, 2H, CH_2_), 1.87–1.74 (m, 2H, CH_2_), 1.63–1.49 (m, 2H, CH_2_). ^13^C NMR (75 MHz, DMSO-d_6_): δ 154.2, 146.4 (d, *J_C_*_–_*_F_* = 235,5 Hz), 146.8 (d, *J_C_*_–_*_F_* = 18,7 Hz), 137.3 (d, *J_C_*_–_*_F_* = 2.2 Hz), 115.4 (d, *J_C_*_–_*_F_* = 18.0 Hz), 109.3 (d, *J_C_*_–_*_F_* = 6.0 Hz), 103.9, 55.7, 44.5, 31.0, 14.4. HRMS-(ESI) *m***/***z*, calculated for [C_12_H_16_FN_2_O_2_]^+^ ([M + H]^+^), 239.1190; found: 239.1186 (See Supplemental Materials).

##### 1-Cyclobutyl-3-(4-fluoro-3-methylphenyl)urea (**21b**)

Compound **21b** was synthesized from cyclobutyl isocyanate **1** and 4-fluoro-3-methylaniline **21a** according to the general protocol described above. Yield: 55%; White solid; m.p.: 183–185 °C; IR (ν, cm^−1^) 3310, 2967, 1636, 1621, 1499, 1249, 1214, 1175; ^1^H NMR (300 MHz, DMSO-d_6_): δ 8.21 (brs, 1H, NH), 7.26–7.21 (m, 1H, Ar), 7.17–7.11 (m, 1H, Ar), 6,93 (t, *J* = 9.2 Hz 1H, Ar), 6.33 (d, *J* = 8.0 Hz, 1H, NH), 4.09 (hex, *J* = 8.0 Hz, 1H, CH), 2.20–2.11 (m, 5H, CH_3_ and CH_2_), 1.87–1.74 (m, 2H, CH_2_), 1.64–1.50 (m, 2H, CH_2_). ^13^C NMR (75 MHz, DMSO-d_6_): δ 156.1 (d, *J_C_*_–_*_F_* = 235.3 Hz), 154.6, 136.9 (d, *J_C_*_–_*_F_* = 3.0 Hz), 124.3 (d, *J_C_*_–_*_F_* = 18.0 Hz), 121.0 (d, *J_C_*_–_*_F_* = 3.7 Hz), 117.2 (d, *J_C_*_–_*_F_* = 8.2 Hz), 115.1 (d, *J_C_*_–_*_F_* = 22.5 Hz), 44.9, 31.5, 14.8, 14.8. HRMS-(ESI) *m***/***z*, calculated for [C_12_H_16_FN_2_O ([M + H]^+^): 223.1241; found: 223.1238 (See Supplemental Materials).

##### 1-Cyclobutyl-3-(3-fluoro-4-methylphenyl)urea (**22b**)

Compound **22b** was synthesized from cyclobutyl isocyanate **1** and 3-fluoro-4-methylaniline **22a** according to the general protocol described above. Yield: 60%; White solid; m.p.: 169–170 °C; IR (ν, cm^−1^) 3286, 2969, 1625, 1594, 1509, 1410, 1265, 1247, 1107; ^1^H NMR (300 MHz, DMSO-d_6_): δ 8.41 (brs, 1H, NH), 7.39–7.34 (m, 1H, Ar), 7.06 (t, *J* = 8.7 Hz 1H, Ar), 6.89 (d, *J* = 8.2 Hz 1H, Ar), 6.41 (d, *J* = 8.0 Hz, 1H, NH), 4.11 (hex, *J* = 8.0 Hz, 1H, CH), 2.23–2.11 (m, 5H, CH_3_ and CH_2_), 1.89–1.75 (m, 2H, CH_2_), 1.65–1.51 (m, 2H, CH_2_). ^13^C NMR (75 MHz, DMSO-d_6_): δ 161.0 (d, *J_C_*_–_*_F_* = 237.8 Hz), 154.5, 140.3 (d, *J_C_*_–_*_F_* = 10,5 Hz), 131.6 (d, *J_C_*_–_*_F_* = 6,7 Hz), 116.2 (d, *J_C_*_–_*_F_* = 17.2 Hz), 113.6 (d, *J_C_*_–_*_F_* = 3.0 Hz), 44.9, 31.5, 14.9, 14.0, 13.9. HRMS-(ESI) *m***/***z*, calculated [C_12_H_16_FN_2_O]^+^ ([M + H]^+^): 223.1241; found: 223.1236 (See Supplemental Materials).

##### 1-(3-Bromo-4-methoxyphenyl)-3-cyclobutylurea (**23b**)

Compound **23b** was synthesized from cyclobutyl isocyanate **1** and 3-bromo-4-methoxylaniline **23a** according to the general protocol described above. Yield: 62%; White solid; m.p.: 176–179 °C; IR (ν, cm^−1^) 3297, 2972, 1623, 1602, 1563, 1493, 1246, 1225, 1084, 1019, 649; ^1^H NMR (300 MHz, DMSO-d_6_): δ 8.36 (brs, 1H, NH), 7.71–7.69 (m, 2H, Ar), 7.18–7.14 (m, 1H, Ar), 6.34 (d, *J* = 8.0 Hz, 1H, NH), 4.10 (hex, *J* = 8.0 Hz, 1H, CH), 2.25 (s, 3H, CH_3_), 2.16–2.07 (m, 2H, CH_2_), 1.86–1.76 (m, 2H, CH_2_), 1.68–1.46 (m, 2H, CH_2_). ^13^C NMR (75 MHz, DMSO-d_6_): δ 154.2, 150.0, 134.6, 122.5, 118.3, 112.8, 110.2, 56.3, 44.6, 31.1, 14.4. HRMS-(ESI) *m***/***z*, calculated for [C_12_H_16_BrN_2_O]^+^ ([M + H]^+^): 283.0441; found: 283.0432 (See Supplemental Materials).

##### 1-(3-Bromo-5-methoxyphenyl)-3-cyclobutylurea (**24b**)

Compound **24b** was synthesized from cyclobutyl isocyanate **1** and 3-bromo-5-methoxylaniline **24a** according to the general protocol described above. Yield: 20%; White solid; m.p.: 156–157 °C; IR (ν, cm^−1^) 3290, 2971, 2938, 1629, 1564, 1561, 1418, 1258, 1194, 1152, 1062, 684; ^1^H NMR (300 MHz, DMSO-d_6_): δ 8.45 (brs, 1H, NH), 7.21–7.20 (m, 1H, Ar), 6.90–6.86 (m, 1H, Ar), 6.63–6.60 (m, 1H, Ar), 6.42 (d, *J* = 8.0 Hz, 1H, NH), 4.09 (hex, *J* = 8.0 Hz, 1H, CH), 2.17–2.08 (m, 5H, CH_3_ and CH_2_), 1.87–1.74 (m, 2H, CH_2_), 1.59–1.48 (m, 2H, CH_2_). ^13^C NMR (75 MHz, DMSO-d_6_): δ 160.4, 153.8, 142.9, 122.0, 112.6, 109.4, 102.4, 55.4, 44.5, 31.014.5. HRMS-(ESI) *m***/***z*, calculated for [C_12_H_16_BrN_2_O]^+^ ([M + H]^+^): 283.0441; found: 283.0431 (See Supplemental Materials).

### 3.2. Biology Methods

#### 3.2.1. Cell Culture

The spontaneously immortalized human keratinocytes (HaCaT) and primary human dermal fibroblasts neonatal (HDFn) were obtained from Thermo Fisher Scientific Inc (Mississauga, ON, Canada). HaCaT and HDFn cell lines were cultured in DMEM medium containing high glucose concentration, glutamine and sodium pyruvate (Hyclone, Logan, UT, USA). Ten percent of fetal bovine serum (FBS, Invitrogen, Burlington, ON, Canada) and 2% penicillin-streptomycin (Thermo Fisher Scientific Inc, Mississauga, ON, Canada) were added to the culture medium. The cells were maintained at 37 °C in a moisture-saturated atmosphere containing 5% CO_2_. THP-1 cells were cultured in RPMI 1640 (ATCC by LGC Standards, Molsheim, France), supplemented with 10% of fetal bovine serum (FBS, Dutscher, Bernolsheim, France), 2% penicillin-streptomycin (Thermo Fisher Scientific, Strasbourg, France) and 2-Mercaptoethanol (Gibco, Thermo Fisher Scientific, Strasbourg, France) to a final concentration of 0.05 mM.

#### 3.2.2. Antiproliferative Activity

The cell proliferation assay was performed using 3-(4,5-dimethylthiazol-2-yl)-2,5-diphenyltetrazolium bromide (MTT). Cells were seeded in 96-well plates at 1 × 10^4^ cells/well. Drugs freshly solubilized in dimethyl sulfoxide (DMSO) at 40 mM were added to a fresh culture medium in serial dilutions starting at the maximal solubility in DMEM medium. Following treatment with DMSO or ACBUs, cells were incubated for 72 h. CA-4 was used as a positive antiproliferative control. DMSO was kept at a constant concentration of 0.5% (*v*/*v*) throughout the experiments. A volume of 10 μL of MTT (5 mg/mL in PBS) was added to the wells after the treatment period and the plates were kept in incubation for 4 h. Afterward, the cells were solubilized by adding 100 μL of 10% (*w*/*v*) sodium dodecyl sulfate (SDS) in 0.01 M HCl, and the plates were then incubated for 24 h in the dark. The plates were read at 595 nm using a SpectraMax^®^ i3x (Molecular Devices, San Jose, CA, USA). The IC50s were calculated using GraphPad Prism version 10.5.0. Values represent the average of three independent experiments performed in triplicate. The error is expressed as the standard deviation.

#### 3.2.3. Evaluation of the Inhibition of IL-6 Production

HaCaT cells (1 × 10^5^) were suspended in 500 μL of DMEM and incubated for 24 h in 24-well microtiter plates at 37 °C in a moisture-saturated atmosphere containing 5% of CO_2_. ACBUs (40 mM in DMSO) were diluted in fresh DMEM and 500 μL of the mixture was added to the well to reach a final concentration of 10 μM. Cells were pretreated for 1 h with either the ACBUs or dexamethasone (1 μM) as a positive control. Then, the culture medium was replaced with fresh DMEM containing 200 ng/mL IL-17A + 20 ng/mL TNF-α (Peprotech, Rocky Hill, NJ, USA). After 6 h of incubation, the culture medium was transferred to 1.5 mL tubes and kept at −80 °C until ELISA was performed. IL-6 concentration in cell medium was determined using an IL-6 human Duoset ELISA kit (Fisher Scientific, Ottawa, ON, Canada) following the manufacturer’s instructions. The absorbance was measured at 450 nm with a Tecan M-200 microplate reader. Values represent the average of two independent experiments were performed three times independently. The error is expressed as the standard deviation. A one-way ANOVA with a Dunnett’s multiple comparisons test was used to analyze significance between the experimental groups (GraphPad Prism V 10.5.0 software, San Diego, CA, USA). *p* ≤ 0.05 was considered statistically significant. * *p*  <  0.05, ** *p*  <  0.01, *** *p*  <  0.001, **** *p*  <  0.0001.

#### 3.2.4. Annexin v/Propidium Iodide (PI) Assay

Cell death was evaluated using an Annexin V/propidium iodide (PI) staining assay, employing the Annexin V-FITC kit according to the manufacturer’s instructions (Miltenyi Biotec, Paris, France), with minor adaptations. HaCaT cells were seeded at a density of 4 × 10^5^ cells per well in 6-well Nunc clear plates (Thermo Fisher Scientific, Strasbourg, France) and allowed to adhere prior to treatment. Cells were then exposed to ACBUs 4b, 10b, and 16b at their maximum solubility limits (100 µM). Staurosporine (500 nM) served as a positive control for apoptosis induction, while untreated cells and 0.2% DMSO were included as negative controls. Following 48 h of incubation, cells were collected and washed once with 1× Binding Buffer prepared from the supplied 20× stock. After centrifugation at 1200 rpm for 5 min, cell pellets (approximately 5 × 10^5^ cells) were gently resuspended in 50 µL of 1× Binding Buffer and incubated with 5 µL of Annexin V-FITC for 15 min at room temperature in the dark. Samples were subsequently washed, centrifuged again, and resuspended in 100 µL of 1× Binding Buffer. Immediately before flow cytometric analysis, 1 µL of PI was added to each sample. Data acquisition allowed discrimination of non-nucleated or mechanically damaged cells (Annexin V^−^/PI^+^), viable (Annexin V^−^/PI^−^), early apoptotic (Annexin V^+^/PI^−^), and late apoptotic or necrotic (Annexin V^+^/PI^+^) populations. The experiments were performed three times independently. The error is expressed as the standard deviation. A one-way ANOVA with a Dunnett’s multiple comparisons test was used to analyze significance between the experimental groups (GraphPad Prism V 10.5.0 software, San Diego, CA, USA). *p* ≤ 0.05 was considered statistically significant. * *p*  <  0.05, ** *p*  <  0.01, *** *p*  <  0.001, **** *p*  <  0.0001.

#### 3.2.5. sEH Inhibition

The inhibitory activity toward soluble epoxide hydrolase (sEH) was assessed using a fluorescence-based enzymatic assay. Recombinant human or murine sEH (Cayman Chemical; Ann Arbor, MI, USA) was diluted in Bis-Tris buffer (25 mM, pH 7.0) to a final concentration of 100 ng/mL and maintained on ice until use. Known sEH inhibitors TPPU and *t*-AUCB (Cayman Chemical, Ann Arbor, MI, USA) and ACBUs **4b**, **10b** and **16b** were prepared as 40 mM stock solutions in DMSO and serially diluted in Bis-Tris buffer to obtain final concentrations of 0, 1.28, 6.4, 32, 160, and 800 nM. DMSO was kept at a constant 0.1% concentration. The assay was performed in black 96-well microplates (Greiner Bio-One GmbH, Kremsmünster, Austria). Briefly, 50 µL of the sEH solution was added to each well, followed by 50 µL of inhibitor solution in Bis-Tris. Control wells contained sEH with 0.1% DMSO and were used to define maximal enzymatic activity. The plate was incubated for 5 min at room temperature with gentle shaking. The enzymatic reaction was initiated by adding 10 µL of the fluorogenic substrate PHOME (Cayman Chemical, Ann Arbor, MI, USA) to each well, resulting in a final substrate concentration of 0.25 µM. After incubation for an additional 10 min at room temperature in the dark, fluorescence was measured using a microplate reader at an excitation wavelength of 330 nm and an emission wavelength of 465 nm. The experiments were performed three times independently. Enzyme activity was expressed using the mean of all the experiments as the percentage of the control wells containing PHOME and DMSO, which were defined as 100% sEH activity. The error is expressed as the standard deviation.

#### 3.2.6. Flow Cytometry Evaluation of iNOS, CD86, CD163, and LAP in a THP1-Polarized Macrophage Inflammatory Model

THP-1 monocytes were differentiated into macrophages (M0) by seeding 2.5 × 10^5^ cells per well in 6-well plates and treating them with phorbol 12-myristate 13-acetate (PMA), following the classical differentiation protocol. Briefly, cells were exposed to PMA (commonly 100 ng/mL) for 24 h to induce adherence and macrophage-like differentiation, followed by a 24 h resting period in PMA-free medium to allow recovery and stabilization of the macrophage phenotype. A reinforced pro-inflammatory macrophage polarization (amplified M1) was then induced using lipopolysaccharide (LPS, 100 ng/mL), interferon-γ (IFN-γ, 20 ng/mL), and tumor necrosis factor-α (TNF-α, 20 ng/mL). Cells were subsequently treated with either ACBUs **4b**, **10b** and **16b** (10 µM), dexamethasone (1 µM) or DMSO 0.2% for 24 h. After treatment, cells were stained for flow cytometric analysis with antibodies against human iNOS (PE), CD86 (FITC), CD163 (VioBlue), and LAP (PE), according to the manufacturer’s recommendations (Miltenyi Biotec, Paris, France). Data acquisition was performed using a MACSQuant^®^ Analyzer 16 flow cytometer (Miltenyi Biotec, Paris, France), with a minimum of 10,000 events collected per sample. The experiments were performed three times independently. The error is expressed as the standard deviation. A one-way ANOVA with a Dunnett’s multiple comparisons test was used to analyze significance between the experimental groups (GraphPad Prism V 10.5.0 software, San Diego, CA, USA). *p* ≤ 0.05 was considered statistically significant. * *p*  <  0.05, ** *p*  <  0.01, *** *p*  <  0.001, **** *p*  <  0.0001.

#### 3.2.7. qPCR

Briefly, 2 × 10^5^ HaCaT cells/well were seeded in 12-well plates. The following day, the HaCaT cells were treated for 4 h with ACBUs (**4b**, **10b** and **16b**) (10 µM) or dexamethasone (1 µM) diluted at the final concentration in supplemented DMEM with a final DMSO concentration of 0.02% with or without TNF-α (10 ng/mL) and IL-17A (100 ng/mL) as previously described.15 After treatments, the HaCaT cells were washed once with PBS and stored at −80 °C prior to RNA extraction. The RNA extraction was achieved using EZ-10 spin column total RNA Minipreps Super Kit (Biobasic, Markham, ON, Canada) according to the manufacturer’s instructions. Total RNA was digested with DNAse I (Sigma, Oakville, ON, Canada) following the manufacturer’s instructions. Afterward, 500 ng of RNAs were reverse-transcribed with M-Mulv (New England biolabs, Whitby, ON, Canada) as previously described [41]. The primers used for qPCR amplification were designed using Primer3. The primers used were IL-6 forward, 5′-GTAGCCGCCCCACACAGA–3′, reverse, 5′–CATGTCTCCTTTCTCAGGGCTG–3′, IL-23 forward 5′-GGGACAACAGTCAGTTCTGCTT–3′, reverse 5′-AGAGAAGGCTCCCCTGTGAA–3 and U6 forward, 5′–CTCGCTTCGGCAGCACA–3′, reverse, 5′–AACGCTTCACGA ATTTGCGT–3′. qPCRs were achieved, using Universal SYBR Green Supermix (Bio-Rad, Hercules, CA, USA) on a CFX96 system (Bio-Rad, Hercules, CA, USA) [29]. A final dissociation curve was achieved to control the specificity of the amplification. Relative mRNA levels were normalized to U6. The experiments were performed three times independently. The error is expressed as the standard deviation.

#### 3.2.8. Evaluation of ACBUs Biotransformation

Microsomes (Mouse skin: 12.5 μL and human skin S9 fraction: 31.25 μL, Sekisui XenoTech, Kansas City, KS, USA; human liver: 25 μL, Corning, Tewksbury, MA, USA) were mixed in 200 μL potassium phosphate buffer 0.5 M (pH 7.4) containing 10 μL NADP+ (Fisher Scientific Inc, Mississauga, ON, Canada) and 10 μL Vivid^®^ regeneration system (Fisher Scientific Inc, Mississauga, ON, Canada). Volume was completed to 990 μL with ultrapure water to form the master mix [42]. The enzymatic reaction was initiated by adding 1 μL of the selected ACBUs solubilized in DMSO to 99 μL of the master mix. Assays were performed for 0, 30 and 60 min at 37 °C and stopped by adding 100 μL of cold MeOH, followed by centrifugation at 15,000 g for 5 min. Supernatants were separated and 20 μL of the solution were submitted to UHPLC-UV analysis (Waters ACQUITY Arc system equipped with 2998 PDA detector, CORTECS C18 reversed-phase column 3.0 mm × 50 mm × 2.7 µM equipped with CORTECS C18 VanGuard Cartridges 2.1 mm × 5 mm × 2.7 µM (Waters, Mississauga, ON, Canada)). The compounds were eluted using MeOH/H_2_O linear gradient (1.0 mL/min) and quantified using the AUC (Area Under Curve). Values represent the average of three independent experiments. The t_1/2_ was calculated using the following equations: t_1/2_ = ln (2/k). The error is expressed as the standard deviation.

### 3.3. Docking Studies

#### 3.3.1. Molecular Docking

Molecular docking was performed to compare the binding of three phenyl-urea derivatives (**4b**, **10b**, **16b**) to the catalytic domains of human soluble epoxide hydrolase (sEH; EPHX2), which comprises a C-terminal epoxide hydrolase (EH) domain [43,44]. The receptor model was the human sEH X-ray structure PDB: 3I28, 1,95 Å resolution (deposition author: Farrow, N.A.; PDB DOI: 10.2210/pdb3i28/pdb), with the associated structural/medicinal chemistry report used as the supporting primary citation [37]. Docking was carried out using AutoDock Vina v1.1.2 [45]. EH cavity center (76, −8, 65) with box size 22 × 18 × 27 Å, and phosphatase cavity center (29, 12, 85) with box size 20 × 16 × 20 Å. For each ligand in each cavity, 4 poses were generated and the top-ranked pose (lowest Vina affinity, kcal/mol) was retained for scoring and analysis [45]. Protein-ligand interaction maps (2D and 3D) were generated using Discovery Studio Visualizer v25.1.0.24284 (BIOVIA, Dassault Systèmes) [46]. As a standard limitation, docking scores were interpreted as ranking metrics rather than absolute binding free energies due to rigid-receptor approximation, lack of explicit waters/induced-fit, and empirical scoring [45].

#### 3.3.2. ΔΔG and Fold-Preference Calculations

To quantify the energetic separation between the synthesized compounds (**4b**, **10b**, **16b**) and the reference inhibitors (TPPU, *t*-AUCB), relative binding free energy differences (ΔΔG) and predicted affinity ratios were derived from the top-ranked AutoDock Vina scores. The docking score of the top-ranked pose for each ligand was used as the representative ΔΔG. The energetic gap for each test compound relative to a reference inhibitor was calculated as:

$$\Delta \Delta G=\Delta G_{\mathrm{test}}-\Delta G_{\mathrm{ref}}$$

where a positive ΔΔG indicates a less favorable predicted binding affinity compared to the reference. To estimate the magnitude of this difference, the theoretical ratio of dissociation constants (K_d_) was approximated using the Boltzmann relationship at 298 K (RT ≈ 0.593 kcal/mol):

Assuming the proportionality between docking score and ΔG for comparative purposes, the ratio of dissociation constants was approximated as:

$$\frac{K_{d,\mathrm{test}}}{K_{d,\mathrm{ref}}}\approx e^{\Delta \Delta G/RT}$$

It is important to note that while these ratios provide a normalized metric for comparing docking performance within this specific workflow, Vina scores are empirical scoring functions and do not represent rigorous absolute binding free energies [40]. Thus, these values should be interpreted as qualitative rankings of geometric fit and interaction potential rather than quantitative thermodynamic constants.

## 4. Conclusions

This study reports the design, synthesis, and preliminary biological evaluation of a new series of ACBU derivatives originally conceived as soluble epoxide hydrolase (sEH) inhibitors, inspired by canonical epoxide hydrolase–targeting scaffolds engaging the catalytic triad of the C-terminal EH domain. However, experimental evaluation revealed that, contrary to the initial design hypothesis, the observed biological effects cannot be attributed to classical sEH enzymatic inhibition. Molecular docking analyses instead suggest possible interactions within the N-terminal phosphatase cavity of sEH rather than the C-terminal epoxide hydrolase catalytic site, with a consistent ranking (**10b** > **16b** > **4b**) in this pocket. In a keratinocyte-based model (IL-17A/TNF-α–stimulated HaCaT cells), several ACBU derivatives, including **4b**, significantly reduced IL-6 secretion without inducing cytotoxicity, suggesting preliminary immunomodulatory potential. Nevertheless, given the early stage of compound optimization, these biological observations should be interpreted cautiously and primarily as indicators for SAR-guided refinement rather than as evidence of a defined anti-inflammatory mechanism.

Overall, the data presented here establish a structural and biological basis for further optimization of the ACBU scaffold. Future work will aim to improve potency and selectivity, and to explore, once suitable lead candidates have been identified, the mechanistic aspects underlying their modulation of IL-6–associated signaling pathways.

## Figures and Tables

**Table 1:** Antiproliferative activity (IC_50_) of ACBUs **2b–24b** on HaCaT cells.

Compound	Antiproliferative Activity (IC_50_, µM) ^1^	Compound	Antiproliferative Activity (IC_50_, µM) ^1^
**2b**	>10	**14b**	12 ± 4
**3b**	>10	**15b**	>100
**4b**	>100	**16b**	>100
**5b**	15 ± 7	**17b**	>100
**6b**	>100	**18b**	12 ± 4
**7b**	>100	**19b**	>100
**8b**	15 ± 7	**20b**	>100
**9b**	>100	**21b**	>100
**10b**	>100	**22b**	>10
**11b**	>100	**23b**	>100
**12b**	>10	**24b**	>100
**13b**	>100	**CA-4**	0.04 ± 0.01

^1^ IC_50_ = Drug concentration required to inhibit cell growth by 50%. The error is expressed as the standard deviation.

**Table 2:** Antiproliferative activity (IC_50_) of soluble ACBUs on HDFn cells.

Compound	Antiproliferative Activity (IC_50_, µM) ^1^	Compound	Antiproliferative Activity (IC_50_, µM) ^1^
**4b**	>100	**15b**	>100
**5b**	62 ± 4	**16b**	>100
**6b**	>100	**17b**	>100
**7b**	>100	**18b**	44 ± 9
**8b**	62 ± 4	**19b**	>100
**9b**	71 ± 2	**20b**	>100
**10b**	>100	**21b**	>100
**11b**	>100	**23b**	>100
**13b**	>100	**24b**	>100
**14b**	44 ± 9		

^1^ IC_50_ = Drug concentration required to inhibit cell growth by 50%. The error is expressed as the standard deviation.

**Table 3:** sEH hydrolase activity inhibition (IC_50_) by ACBUs **4b**, **10b**, and **16b**.

Compound	sEH Hydrolase Activity Inhibition (IC50, nM)	
	Human	Mouse
**TPPU** ^1^	0.014 ± 0.006	0.2 ± 0.1
***t*-AUCB** ^2^	0.4 ± 0.2	0.05 ± 0.02
**4b**	5600 ± 400	2400 ± 600
**10b**	2200 ± 400	90 ± 50
**16b**	4900 ± 500	2700 ± 700

^1^ TTPU = 1-Trifluoromethoxyphenyl-3-(1-propionylpiperidin-4-yl) urea. ^2^ t-AUCB = trans-4-[4-(3-adamantan-1-yl-ureido)-cyclohexyloxy]-benzoic acid.

**Table 4:** Inhibition of key inflammatory genes IL-6, IL-23 and TNF-α expression in HaCaT cells stimulated with TNFα and IL-17A by the selected ACBUs **4b**, **10b** and **16b** (10 μM).

Compound	IL-6 Expression Inhibition (%) ^1^	IL-23 Expression Inhibition (%) ^1^
**Dexamethasone**	70 ± 10	65 ± 9
**4b**	24 ± 5	40 ± 10
**10b**	27 ± 9	50 ± 10
**16b**	19 ± 5	21 ± 5

^1^ Results represent the reduction in the IL-6 and IL-23 expression compared to their expression in the cell treated only with IL-17A and TNF-α.

**Table 5:** Half-life (t_1/2_) of ACBUs **4b** and **10b** biotransformed in human liver microsomes and human and mouse skin microsomes.

Compound	Human Liver Microsomes (t_1/2_, min)	Human Skin Microsomes (t_1/2_, min)	Mouse Skin Microsomes (t_1/2_, min)
**4b**	180 ± 20	220 ± 40	120 ± 40
**10b**	420 ± 120	260 ± 10	230 ± 70

## Data Availability

The original contributions presented in this study are included in the article and Supplementary Material. Further inquiries can be directed to the corresponding authors.

## Supplementary Materials

The following supporting information can be downloaded at: https://www.mdpi.com/article/10.3390/ph19030355/s1, (spectra for characterization of the synthesized compounds).

## Abbreviations

The following abbreviations are used in this manuscript:

ACBU	Arylcyclobutylurea
Asp	Aspartic acid
*t*-AUCB	*trans*-4-[4-(3-adamantan-1-yl-ureido)-cyclohexyloxy]-benzoic acid
CA-4	Combretastatin A-4
CD86	Cluster of Differentiation 86
CD163	Cluster of Differentiation 163
Dex	Dexamethasone
DHETs	Dihydroxyeicosatrienoic acids
DMSO	Dimethylsulfoxide
EETs	Epoxyeicosatrienoic acids
HaCaT	Human Adult low Calcium, immortalized keratinocytes
HDFn	Human Dermal Fibroblasts, normal
HRMS-(ESI)	High-Resolution Mass Spectrometry with Electrospray Ionization
IC_50_	Inhibitory Concentration 50%
IL-6	Interleukine 6
IL-17	Interleukine 17
IL-23	interleukine 23
IFNγ	Interferon γ
IMIDs	Immune-mediated inflammatory diseases
iNOS	Inducible Nitric Oxide Synthase
IR	Infrared
JAK	Janus Kinase
LPS	Lipopolysaccharide
Met	Methionine
m.p	Melting point
MTT	3-(4,5-diméthylthiazol-2-yl)-2,5-diphényltétrazolium bromide
mRNA	Messenger ribonucleic acid
NF-κB	Nuclear Factor kappa-light-chain-enhancer of activated B cells
NMR	Nuclear Magnetic Resonance
NSAIDs	Non-steroidal anti-inflammatory drugs
Phe	Phenylalanine
PHOME	6-(Phenyl)O-methyl-7-hydroxycoumarinyl ester
PMA	Phorbol 12-myristate 13-acetate
qPCR	Quantitative Polymerase Chain Reaction, also called real-time PCR
sEH	Soluble hydrolyse enzyme
sEHi	Soluble hydrolyse enzyme inhibitor
STAT1	Signal Transducer and Activator of Transcription 1
STAT3	Signal Transducer and Activator of Transcription 3
Stau	Staurosporine
THP-1	Tissue Human Promonocyte (human monocytic leukemia cell line)
TNFα	Tumor necrosis factor
TPPU-	Trifluoromethoxyphenyl-3-(1-propionylpiperidin-4-yl) urea.
Trp	Tryptophane
Tyr	Tyrosine
UHPLC-UV	Ultra-High Performance Liquid Chromatography coupled with Ultraviolet detection
Val	valine
ΔG	Gibbs free energy change in a process (e.g., protein folding, ligand binding)
ΔΔG	Difference between two ΔG values
