# Supplementary material for: Design, Synthesis, and Structure–Activity Relationships of Substituted Phenyl Cyclobutylureas as Potential Modulators of Inflammatory Responses
Source: Pharmaceuticals (Basel). 2026 Feb 25;19(3):355. doi: 10.3390/ph19030355 (PMC13029360; doi:10.3390/ph19030355)
Supplement: Supplementary file 1 [file pharmaceuticals-19-00355-s001.zip › pharmaceuticals-4170998-supplementary.pdf]

Supplemental Material

Design, synthesis, and biological evaluation of a second generation of substituted phenyl cyclobutylureas as potential anti-inflammatory agents

Atziri Corin Chavez Alvarez[a, *], Antoine Carpentier[b], Ahmed Sahli[b], Martin Perreault[b], Aichatou Diallo Ngon[a] and Emmanuel Moreau[a, *]

[a] UMR INSERM 1240, Université Clermont-Auvergne,58 rue Montalembert, 63000 Clermont-Ferrand, France
[b] Centre de recherche du CHU de Québec-Université Laval, Centre hospitalier de l'Université Laval, 2705 Boul Laurier, Québec, QC G1V 0E8, Canada

***Corresponding authors:** Emmanuel Moreau, Ph.D., e-mail: emmanuel.moreau@uca.fr;

Atziri Corin Chavez Alvarez, PhD.,, e-mail: atziri_corin.chavez_alvarez@uca.fr.

Table of Contents

Compound 2b : ^{1}H, ^{13}C NMR, MS, IR spectra, and LC chromatogram (Figures **S1**-**S5**) Page 3

Compound 3b : ^{1}H, ^{13}C NMR, MS, IR spectra, and LC chromatogram (Figures **S6**-**S10**) Page 6

Compound 4b : ^{1}H, ^{13}C NMR, MS, IR spectra, and LC chromatogram (Figures **S11**-**S15**) Page 9

Compound 5b : ^{1}H, ^{13}C NMR, MS, IR spectra, and LC chromatogram (Figures **S16**-**S20**) Page 12

Compound 6b : ^{1}H, ^{13}C NMR, MS, IR spectra, and LC chromatogram (Figures **S21**-**S25**) Page 15

Compound 7b : ^{1}H, ^{13}C NMR, MS, IR spectra, and LC chromatogram (Figures **S26**-**30**) Page 18

Compound 8b : ^{1}H, ^{13}C NMR, MS, IR spectra, and LC chromatogram (Figures **S31**-**S35**) Page 21

Compound 9b : ^{1}H, ^{13}C NMR, MS, IR spectra, and LC chromatogram (Figures **S36**-**S40**) Page 24

Compound 10b : ^{1}H, ^{13}C NMR, MS, IR spectra, and LC chromatogram (Figures **S41**-**S45**) Page 27

Compound 11b : ^{1}H, ^{13}C NMR, MS, IR spectra, and LC chromatogram (Figures **S46**-**S50**) Page 30

Compound 12b : ^{1}H, ^{13}C NMR, MS, IR spectra, and LC chromatogram (Figures **S51**-**S55**) Page 33

Compound 13b : ^{1}H, ^{13}C NMR, MS, IR spectra, and LC chromatogram (Figures **S56**-**S60**) Page 36

Compound 14b : ^{1}H, ^{13}C NMR, MS, IR spectra, and LC chromatogram (Figures **S61**-**S65**) Page 39

Compound 15b : ^{1}H, ^{13}C NMR, MS, IR spectra, and LC chromatogram (Figures **S66**-**S70**) Page 42

Compound 16b : ^{1}H, ^{13}C NMR, MS, IR spectra, and LC chromatogram (Figures **S71**-**S75**) Page 45

Compound 17b : ^{1}H, ^{13}C NMR, MS, IR spectra, and LC chromatogram (Figures **S76**-**S80**) Page 48

Compound 18b : ^{1}H, ^{13}C NMR, MS, IR spectra, and LC chromatogram (Figures **S81**-**S85**) Page 51

Compound 19b : ^{1}H, ^{13}C NMR, MS, IR spectra, and LC chromatogram (Figures **S86**-**S90**) Page 54

Compound 20b : ^{1}H, ^{13}C NMR, MS, IR spectra, and LC chromatogram (Figures **S91**-**S95**) Page 57

Compound 21b : ^{1}H, ^{13}C NMR, MS, IR spectra, and LC chromatogram (Figures **S96**-**S100**) Page 60

Compound 22b : ^{1}H, ^{13}C NMR, MS, IR spectra, and LC chromatogram (Figures **S101**-**S105**) Page 63

Compound 23b : ^{1}H, ^{13}C NMR, MS, IR spectra, and LC chromatogram (Figures **S106**-**S110**) Page 66

Compound 24b : ^{1}H, ^{13}C NMR, MS, IR spectra, and LC chromatogram (Figures **S111**-**S115**) Page 69

Table S1 Page 72

Table S2 Page 73

Compound 2b

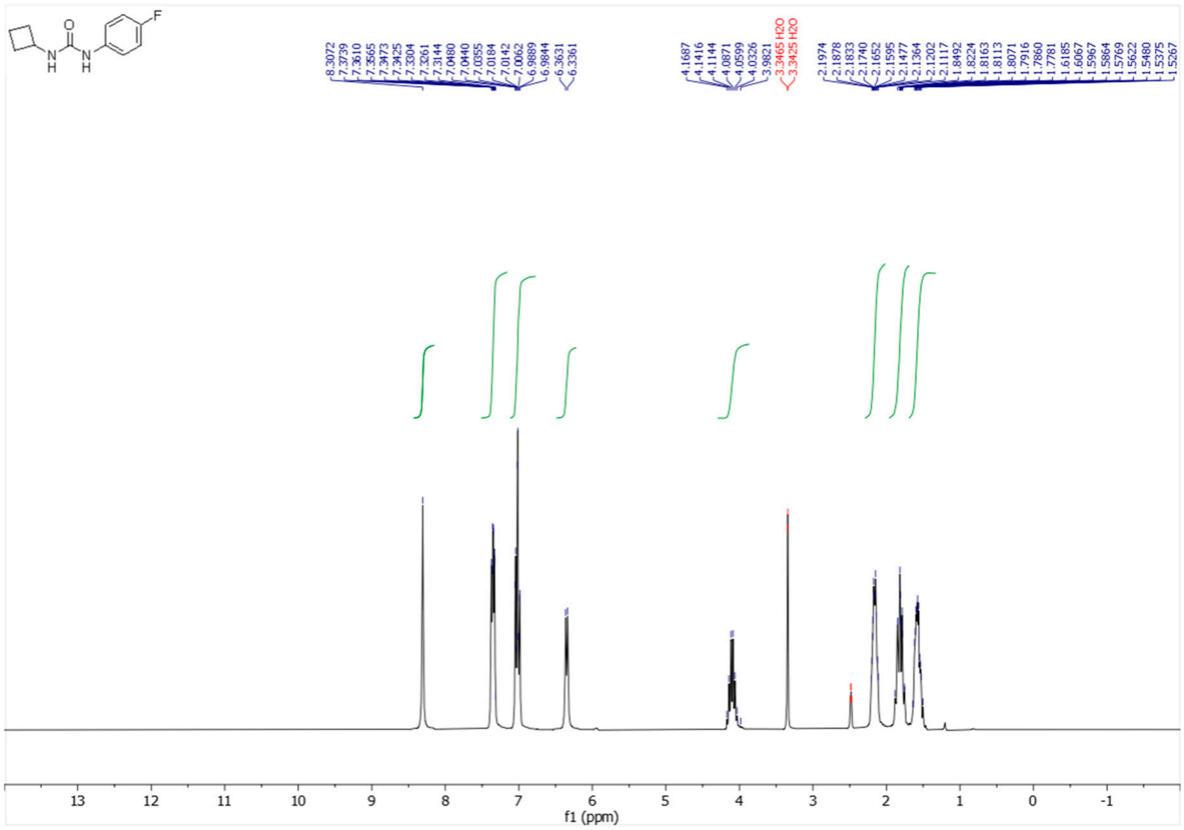

Figure S1 : ^{1}H NMR Spectrum of compound **(2b)**

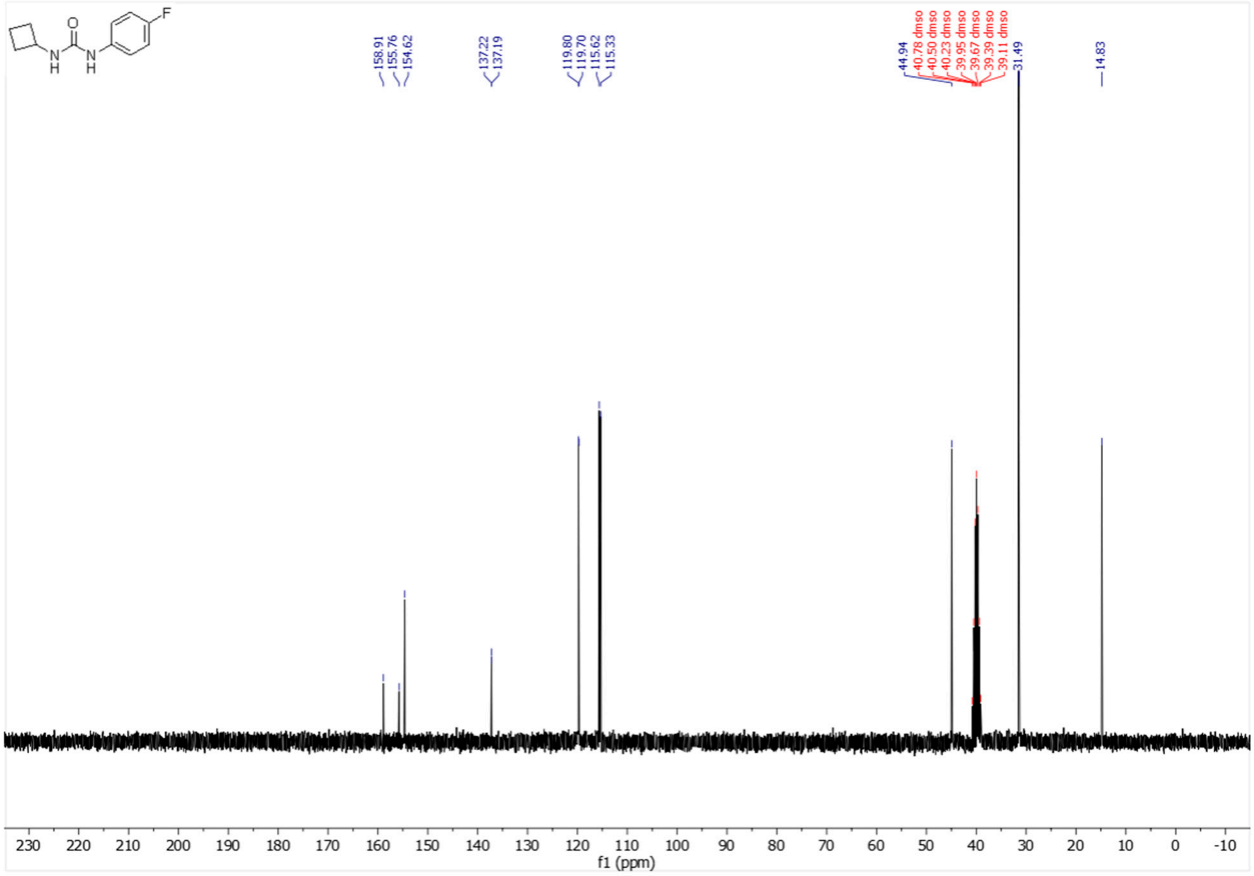

Figure S2 : ^{13}C NMR Spectrum of compound **(2b)**

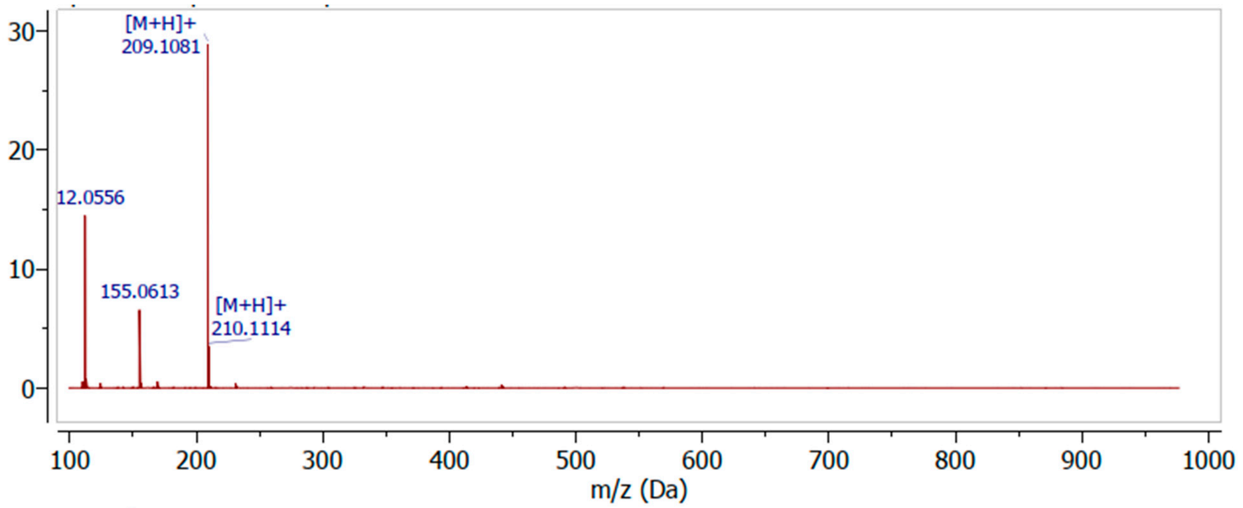

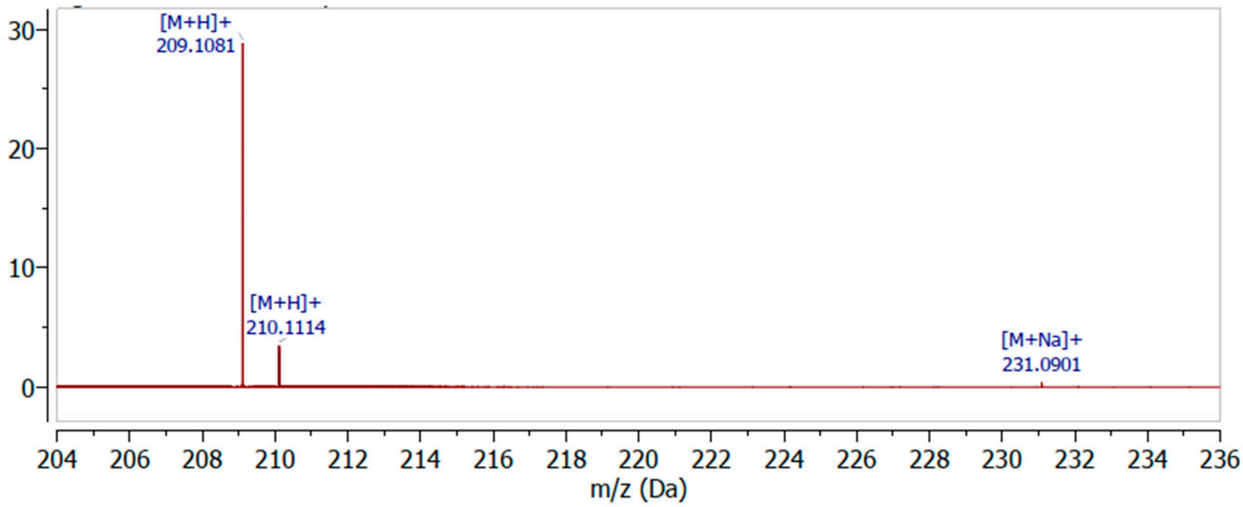

Composé trouvé: C11H13FN2O

Masse mesurée	Masse attendue	Intensité	Erreur (ppm)	Erreur (Da)	Ion identifié	Formule confirmée
209.1081	209.1085	67827604	-1.9	-0.0004	[M+H]+	C11H13FN2O
210.1114	210.1115	8149575	-0.4	-0.0001	[M+H]+	C11H13FN2O
231.0901	231.0904	1031315	-1.4	-0.0003	[M+Na]+	C11H13FN2O

Figure S3 : MS spectrum of compound **(2b)**

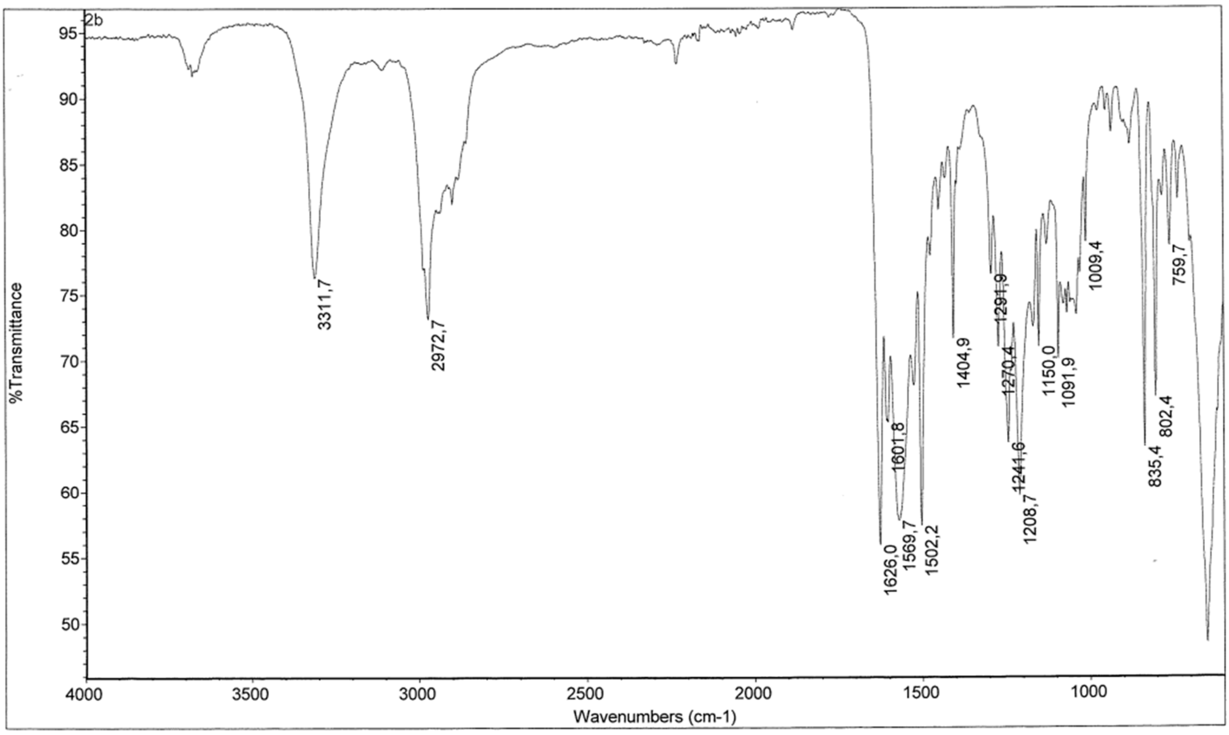

Figure S4 : IR spectrum of compound **(2b)**

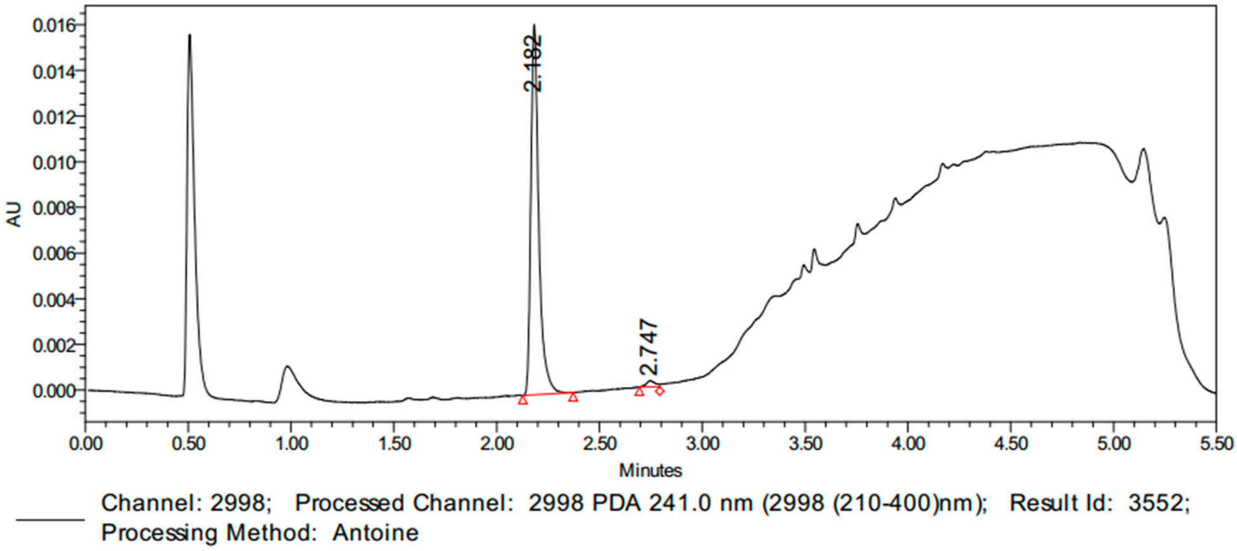

Processed Channel Descr.: 2998 PDA 241.0 nm (2998 (210-400)nm)

	Processed Channel Descr.	RT	Area	Height	% Height
1	2998 PDA 241.0 nm (2998 (210-400)nm)	2.182	43147	16226	98.35
2	2998 PDA 241.0 nm (2998 (210-400)nm)	2.747	850	272	1.65

Figure S5 : LC chromatogram of compound **(2b)**

Compound 3b

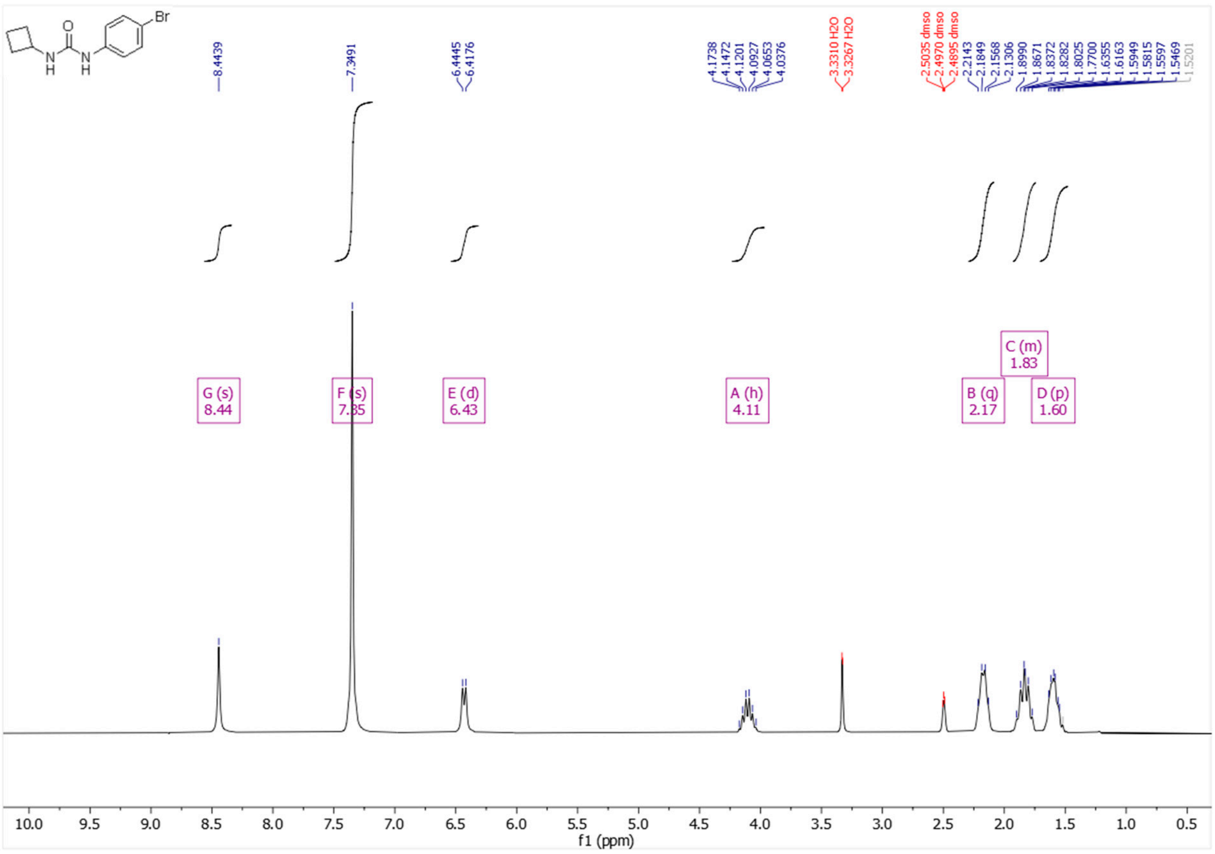

Figure S6 : ^{1}H NMR Spectrum of compound **(3b)**

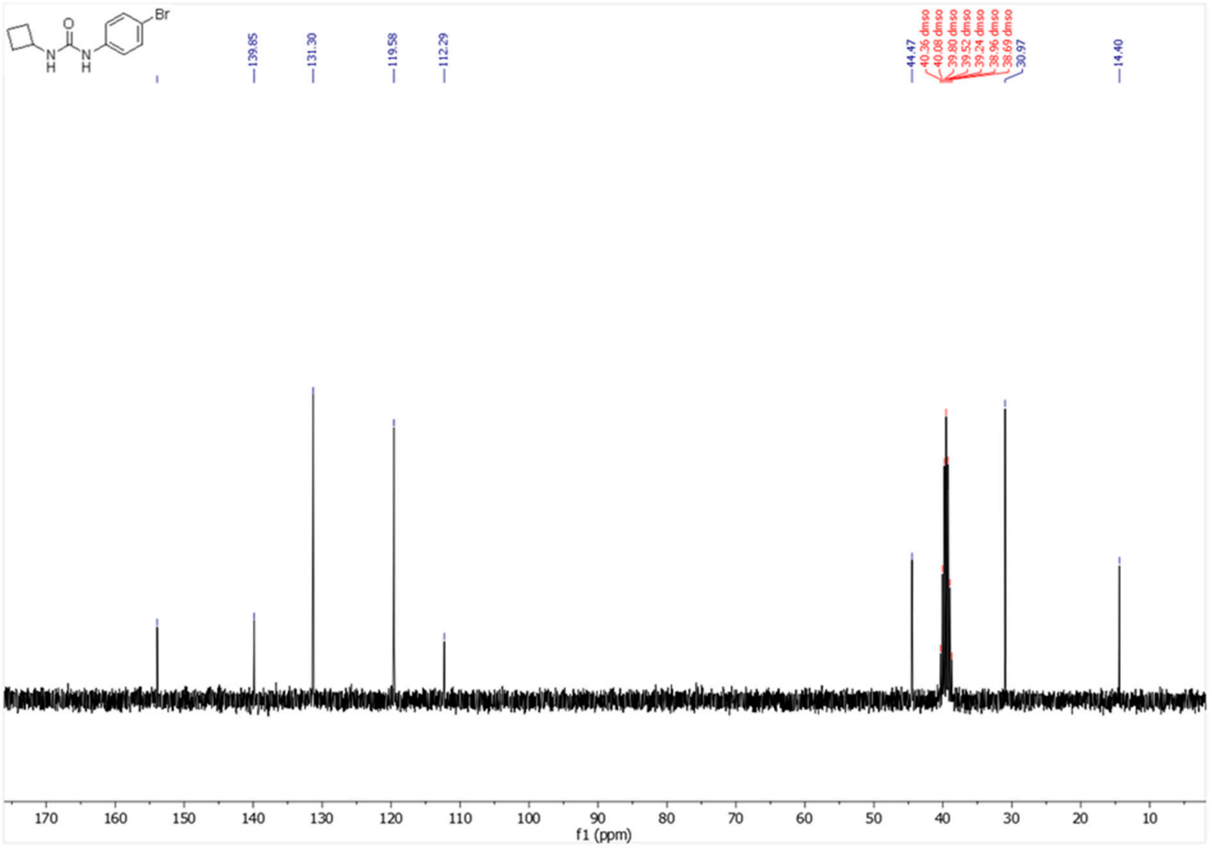

Figure S7 : ^{13}C NMR Spectrum of compound **(3b)**

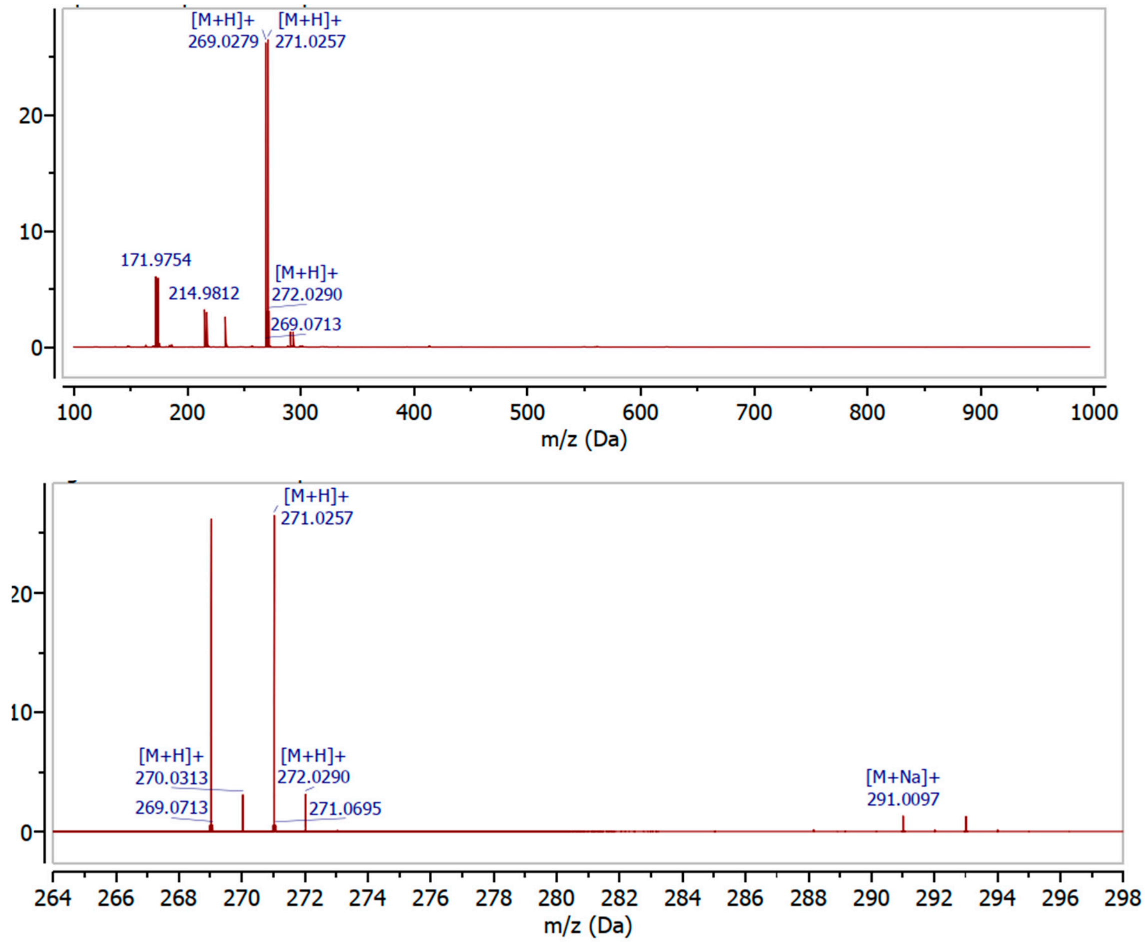

Composé trouvé: C11H13BrN2O

Masse mesurée	Masse attendue	Intensité	Erreur (ppm)	Erreur (Da)	Ion identifié	Formule confirmée
269.0279	269.0284	158490473	-1.8	-0.0005	[M+H]+	C11H13BrN2O
270.0313	270.0314	18986587	-0.7	-0.0002	[M+H]+	C11H13BrN2O
271.0257	271.0264	155845140	-2.8	-0.0008	[M+H]+	C11H13BrN2O
272.0290	272.0294	18511512	-1.6	-0.0004	[M+H]+	C11H13BrN2O
291.0097	291.0103	7844735	-2.3	-0.0007	[M+Na]+	C11H13BrN2O
293.0076	293.0084	7655169	-2.5	-0.0007	[M+Na]+	C11H13BrN2O

Figure S8 : MS spectrum of compound **(3b)**

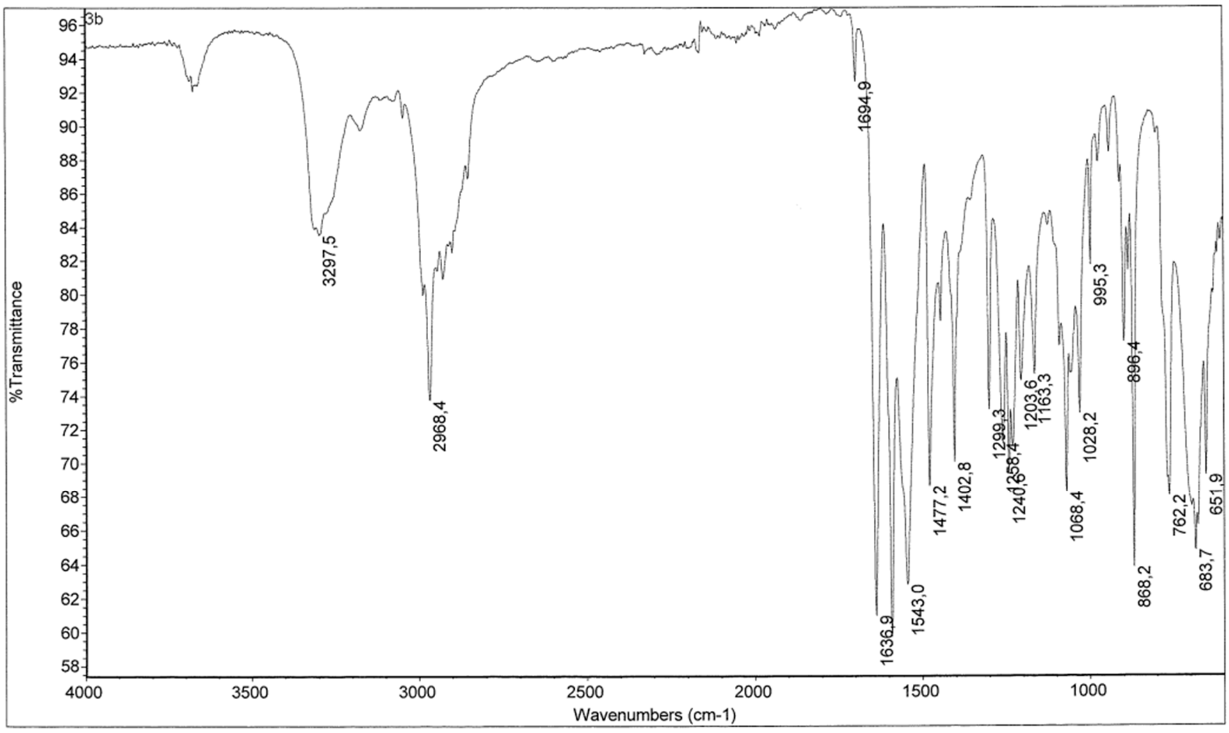

Figure S9 : IR spectrum of compound **(3b)**

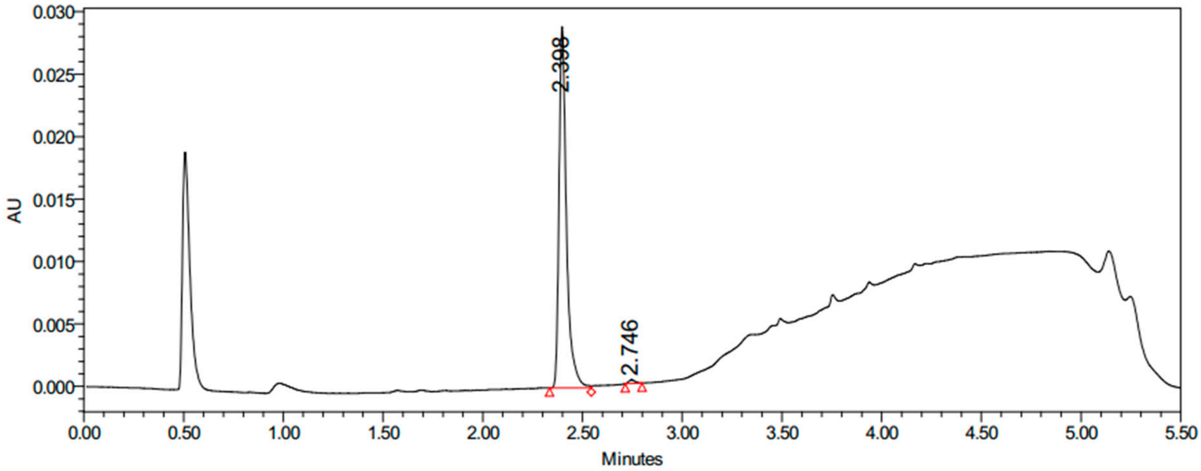

Channel: 2998; Processed Channel: 2998 PDA 241.0 nm (2998 (210-400)nm); Result Id: 3555; Processing Method: Antoine

Processed Channel Descr.: 2998 PDA 241.0 nm (2998 (210-400)nm)

	Processed Channel Descr.	RT	Area	Height	% Height
1	2998 PDA 241.0 nm (2998 (210-400)nm)	2.398	75676	28944	98.99
2	2998 PDA 241.0 nm (2998 (210-400)nm)	2.746	626	294	1.01

Figure S10 : LC chromatogram of compound **(3b)**

Compound 4b

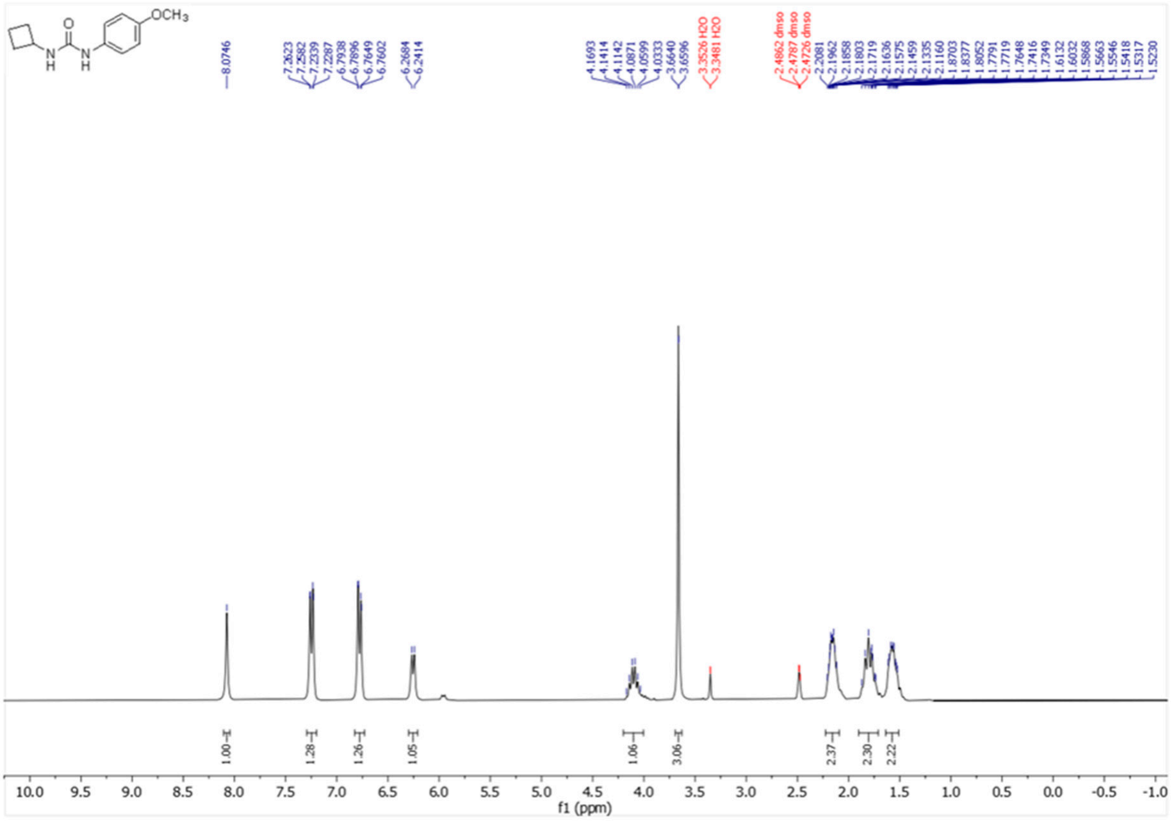

Figure S11 : ^{1}H NMR Spectrum of compound **(4b)**

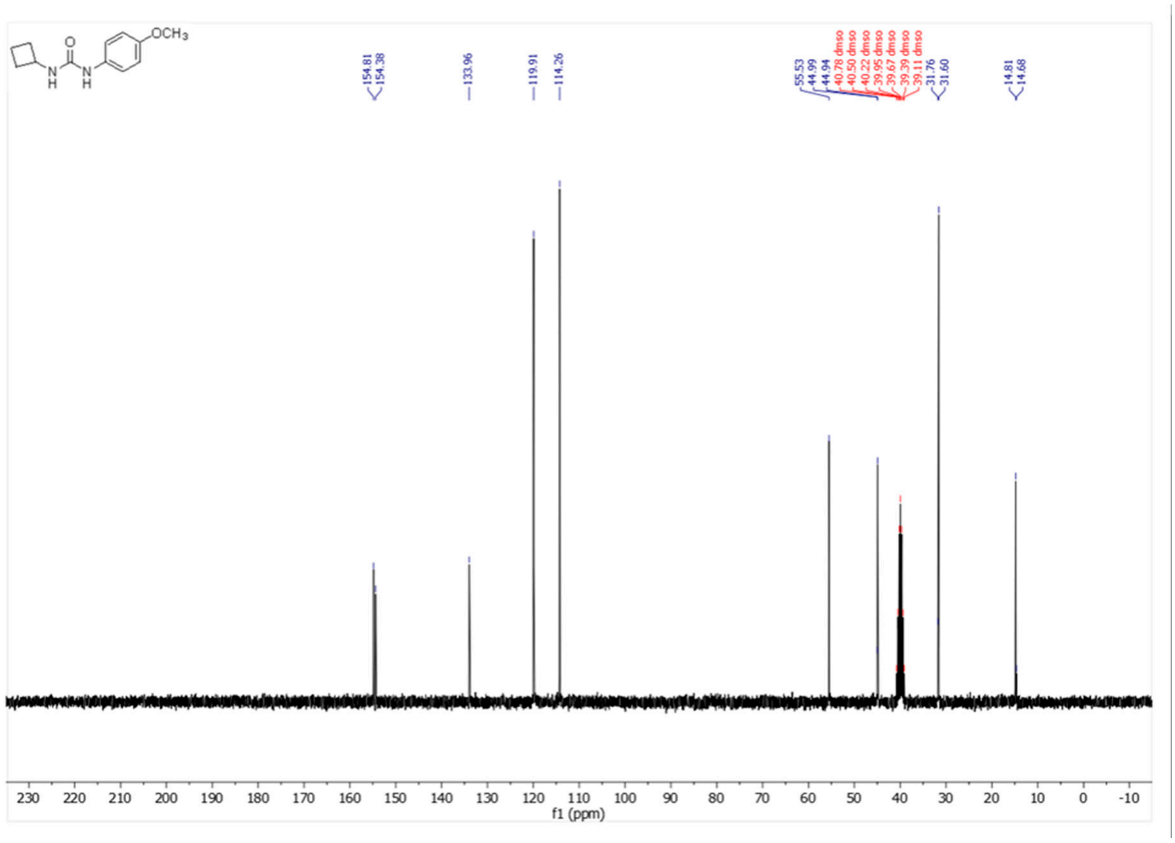

Figure S12 : ^{13}C NMR Spectrum of compound **(4b)**

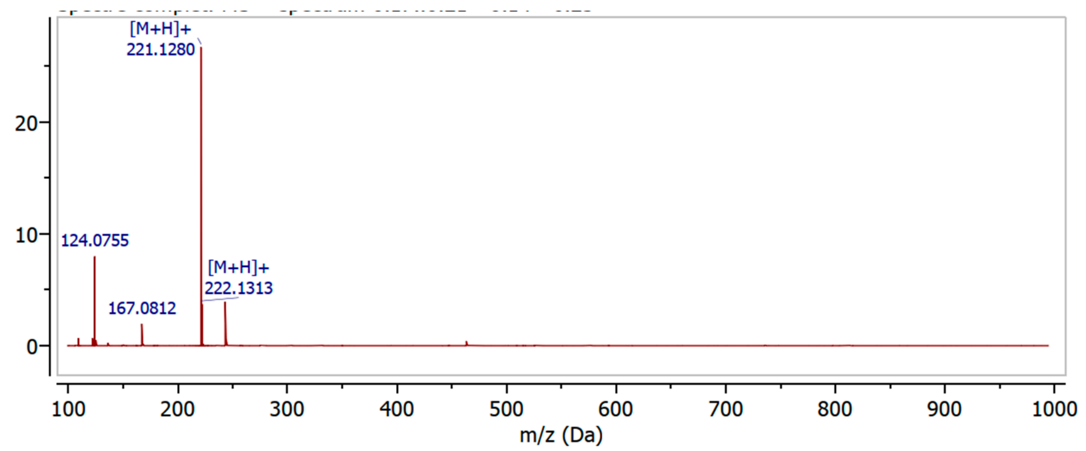

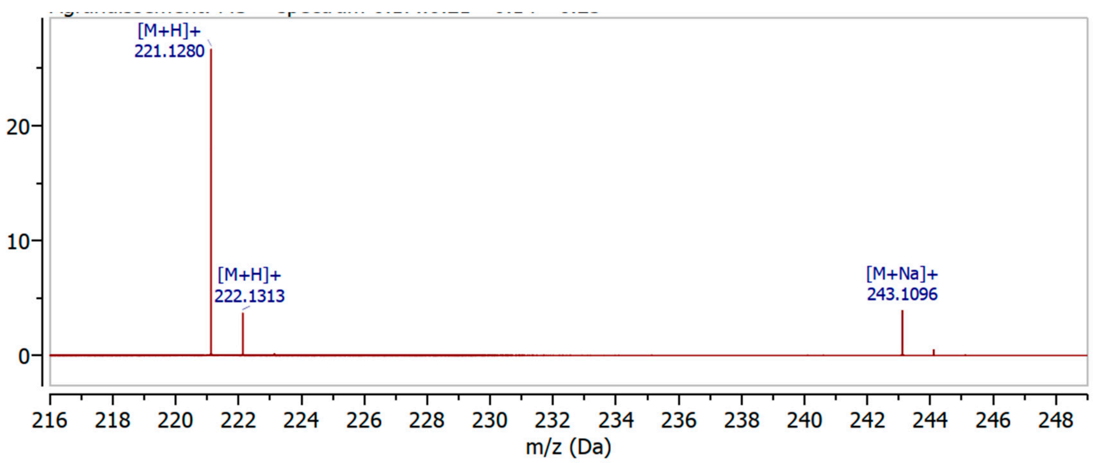

Composé trouvé: C12H16N2O2

Masse mesurée	Masse attendue	Intensité	Erreur (ppm)	Erreur (Da)	Ion identifié	Formule confirmée
221.1280	221.1285	274810161	-2.1	-0.0005	[M+H]+	C12H16N2O2
222.1313	222.1315	37347516	-1.0	-0.0002	[M+H]+	C12H16N2O2
243.1096	243.1104	36759706	-3.3	-0.0008	[M+Na]+	C12H16N2O2
244.1130	244.1135	4943474	-1.8	-0.0004	[M+Na]+	C12H16N2O2

<u>Figure S13 :</u> MS spectrum of compound **(4b)**

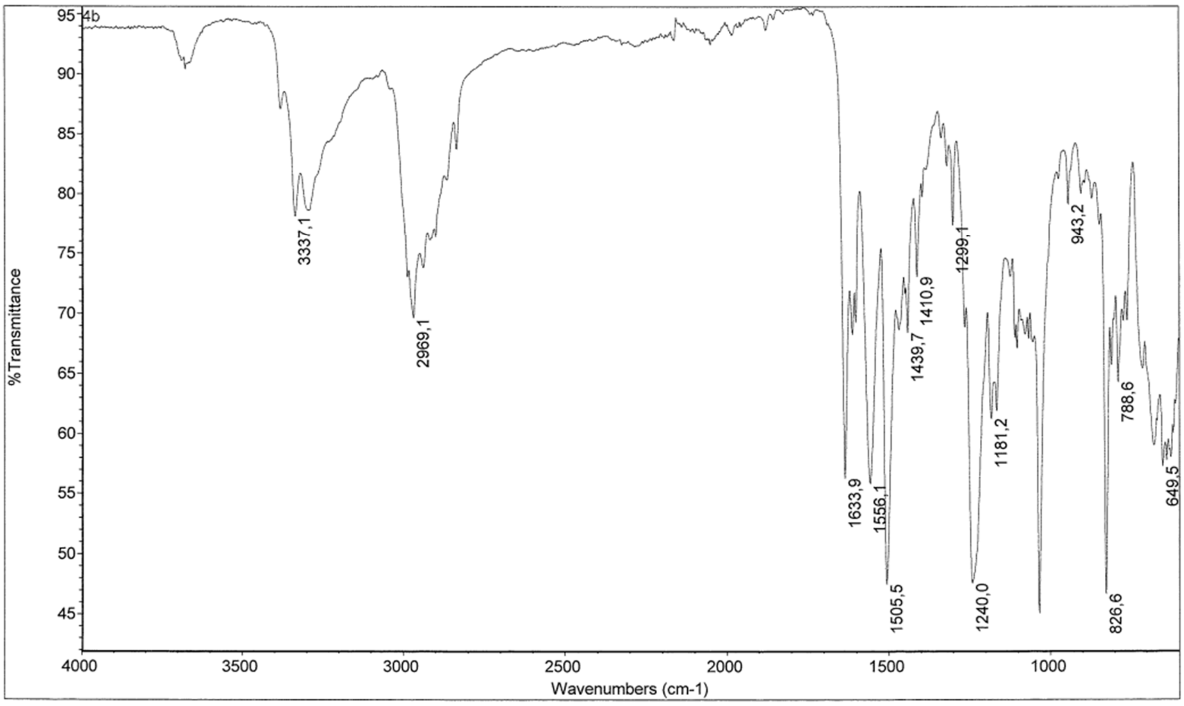

<u>Figure S14 :</u> IR spectrum of compound **(4b)**

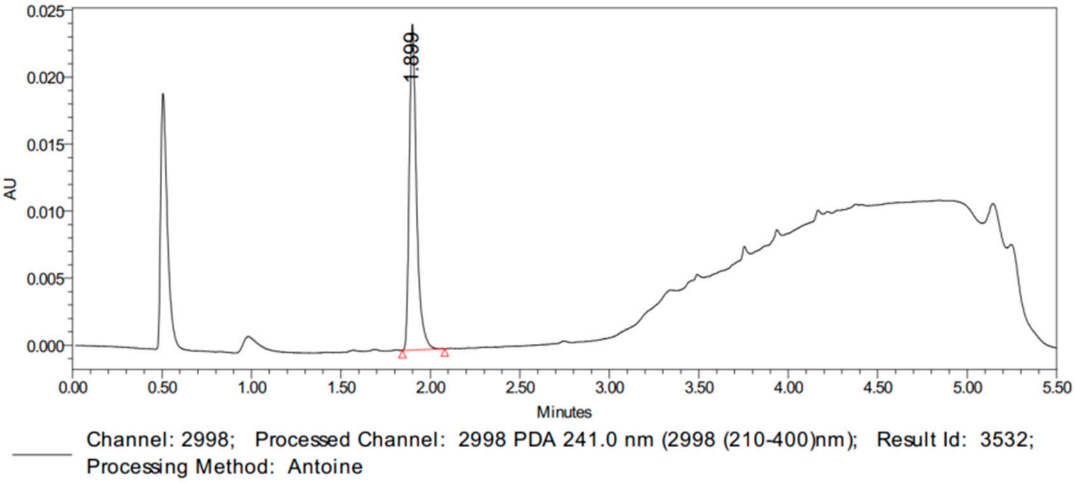

Processed Channel Descr.: 2998 PDA 241.0 nm (2998 (210-400)nm)

	Processed Channel Descr.	RT	Area	Height	% Height
1	2998 PDA 241.0 nm (2998 (210-400)nm)	1.899	68380	24324	100.00

<u>Figure S15 :</u> LC chromatogram of compound **(4b)**

Compound 5b

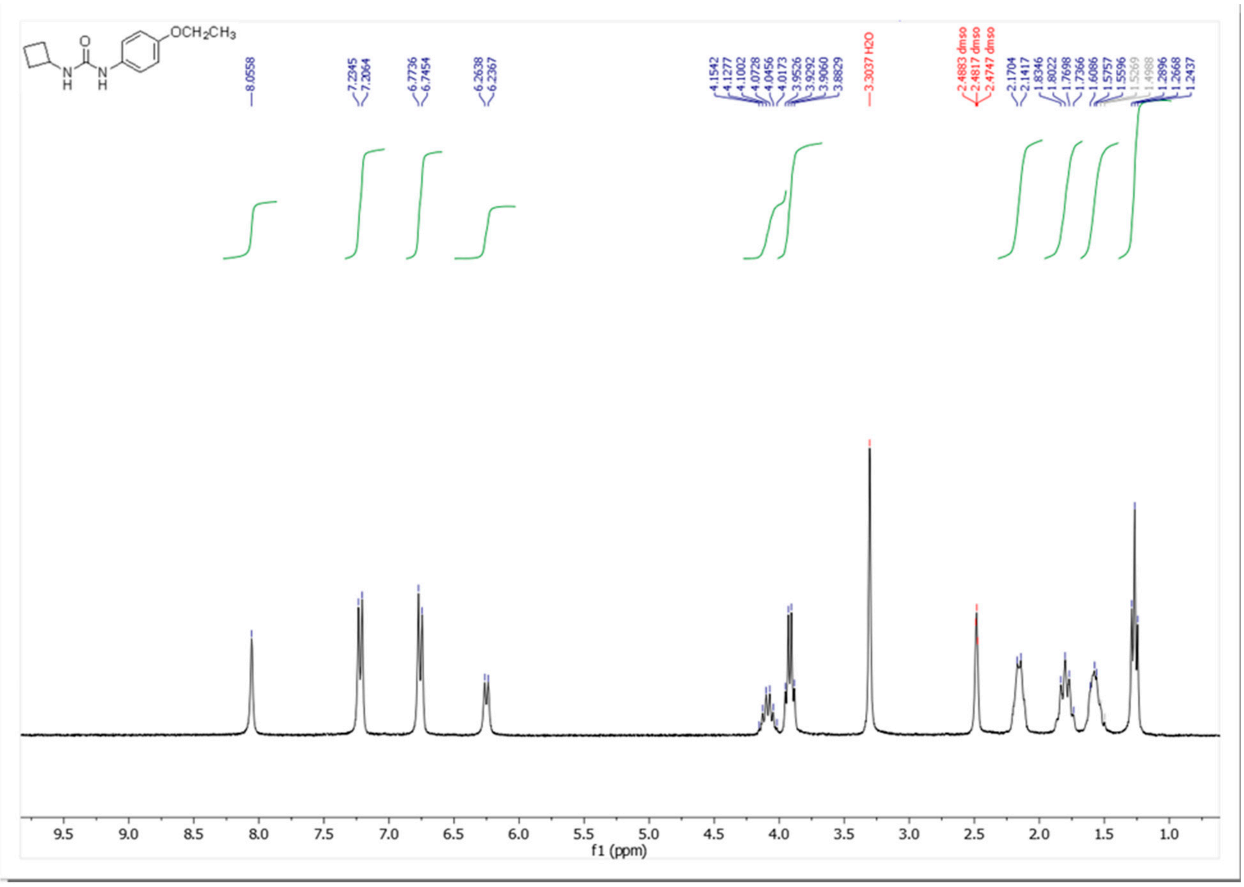

Figure S16 : ^{1}H NMR Spectrum of compound **(5b)**

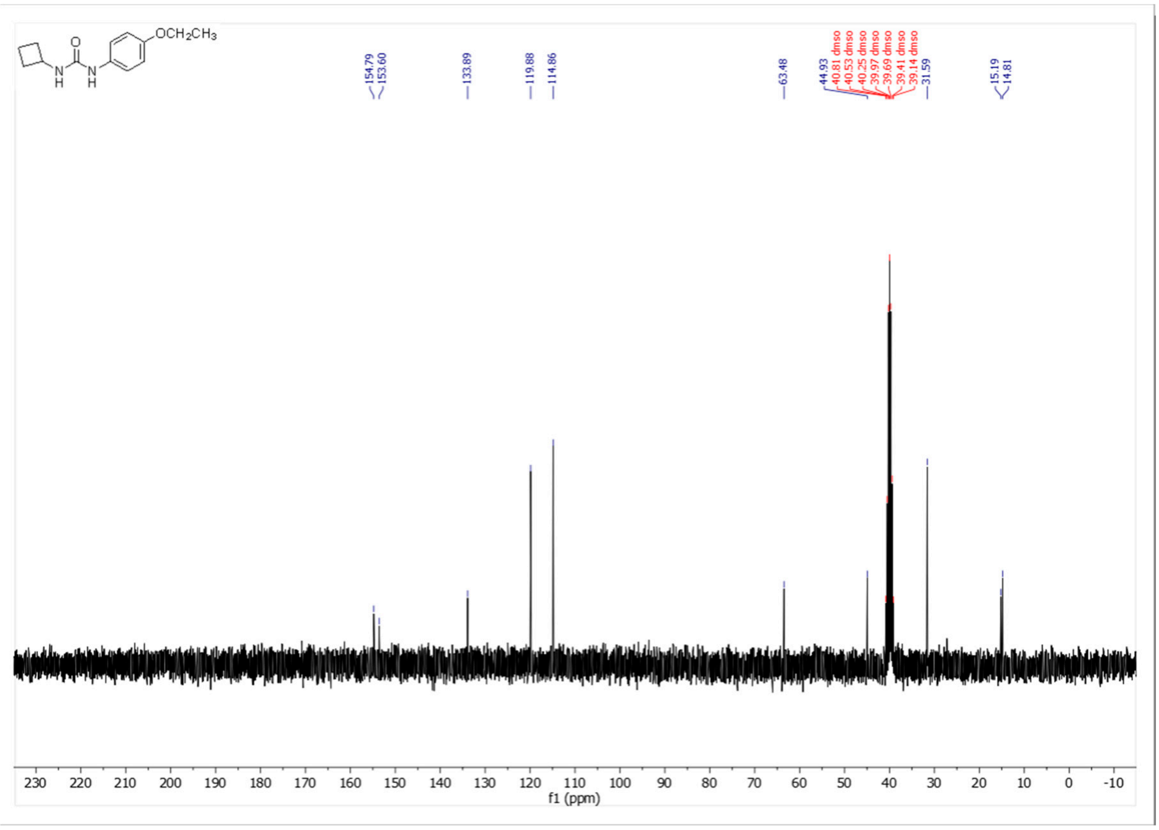

Figure S17 : ^{13}C NMR Spectrum of compound **(5b)**

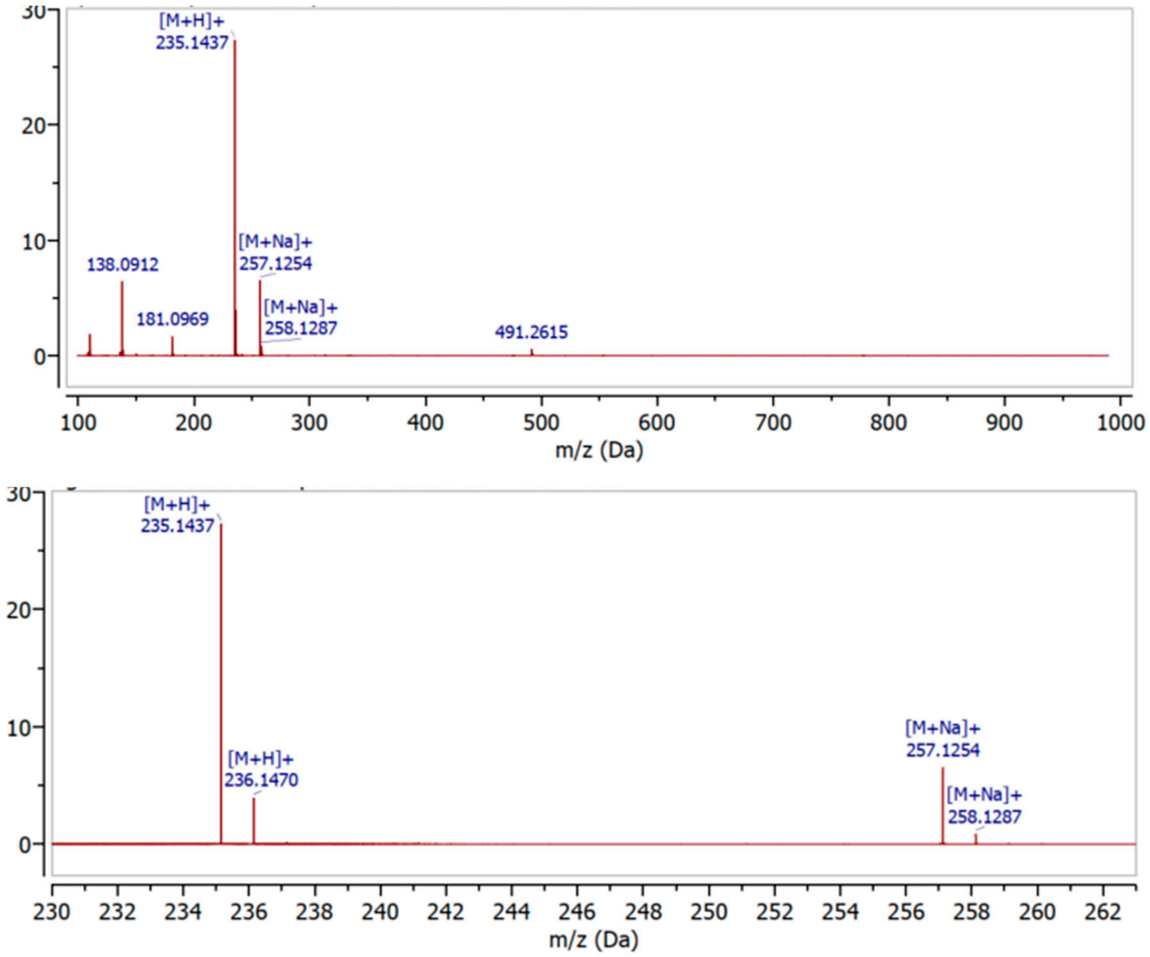

Composé trouvé: C13H18N2O2

Masse mesurée	Masse attendue	Intensité	Erreur (ppm)	Erreur (Da)	Ion identifié	Formule confirmée
235.1437	235.1441	505926655	-1.9	-0.0004	[M+H]+	C13H18N2O2
236.1470	236.1472	72452501	-0.9	-0.0002	[M+H]+	C13H18N2O2
257.1254	257.1260	114645009	-2.4	-0.0006	[M+Na]+	C13H18N2O2
258.1287	258.1291	15168775	-1.6	-0.0004	[M+Na]+	C13H18N2O2

Figure S18 : MS spectrum of compound **(5b)**

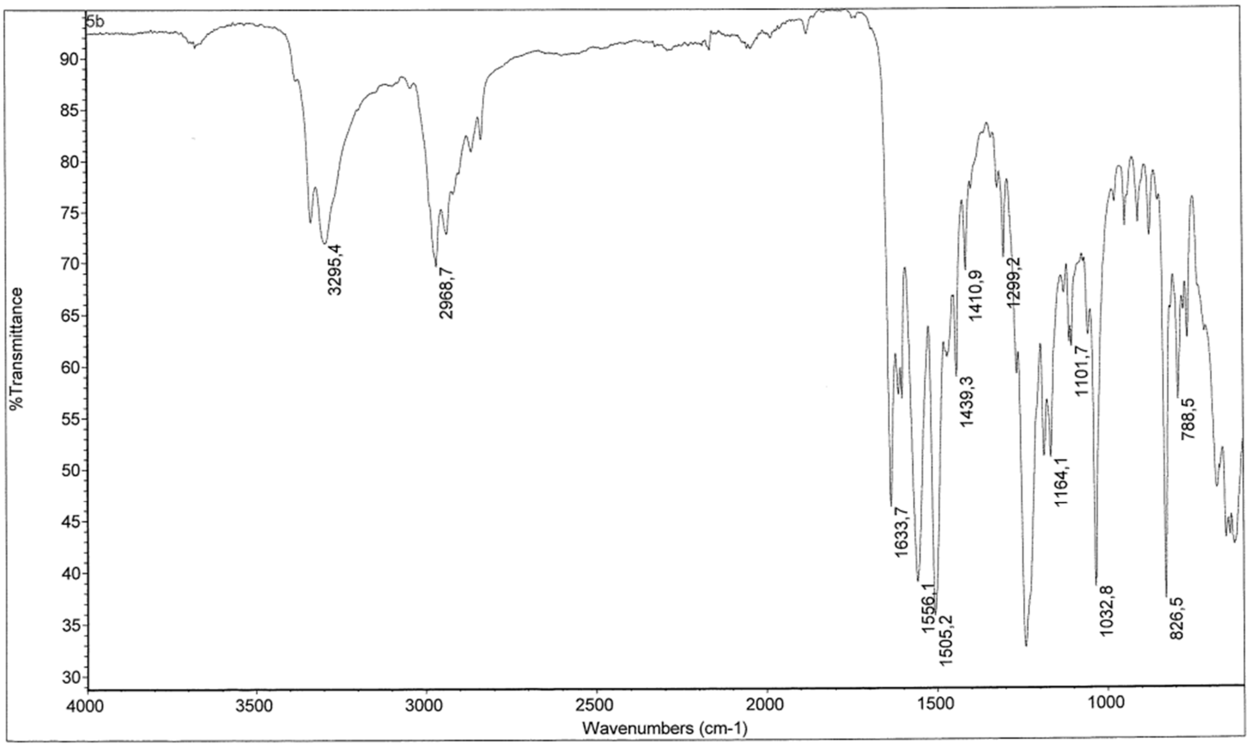

Figure S19 : IR spectrum of compound **(5b)**

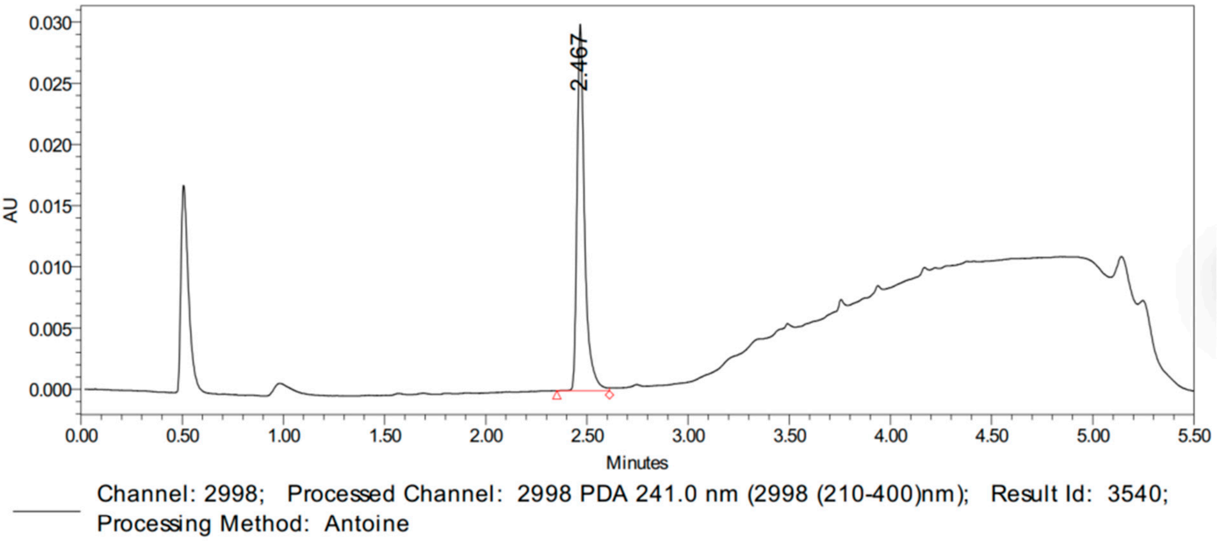

Processed Channel Descr.: 2998 PDA 241.0 nm (2998 (210-400)nm)

	Processed Channel Descr.	RT	Area	Height	% Height
1	2998 PDA 241.0 nm (2998 (210-400)nm)	2.467	77341	29938	100.00

Figure S20 : LC chromatogram of compound **(5b)**

Compound 6b

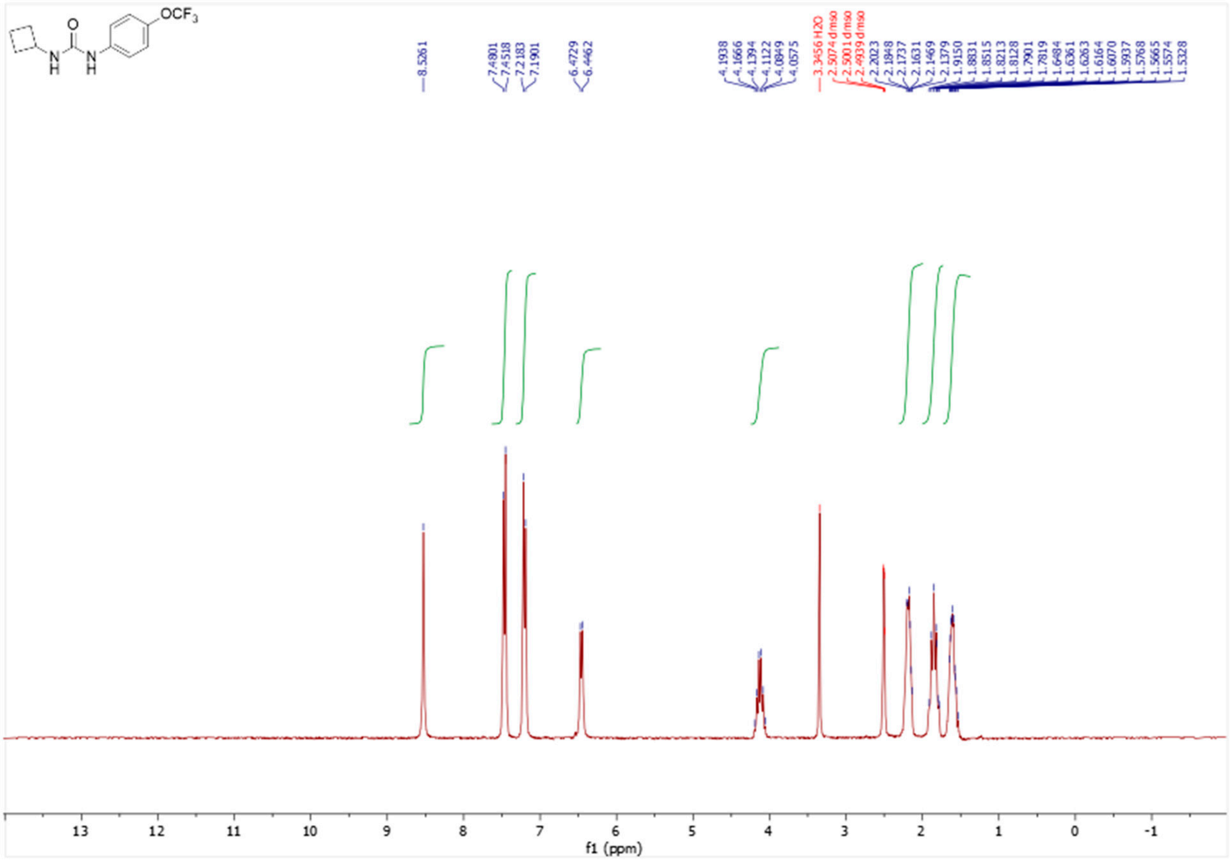

Figure S21 : ^{1}H NMR Spectrum of compound **(6b)**

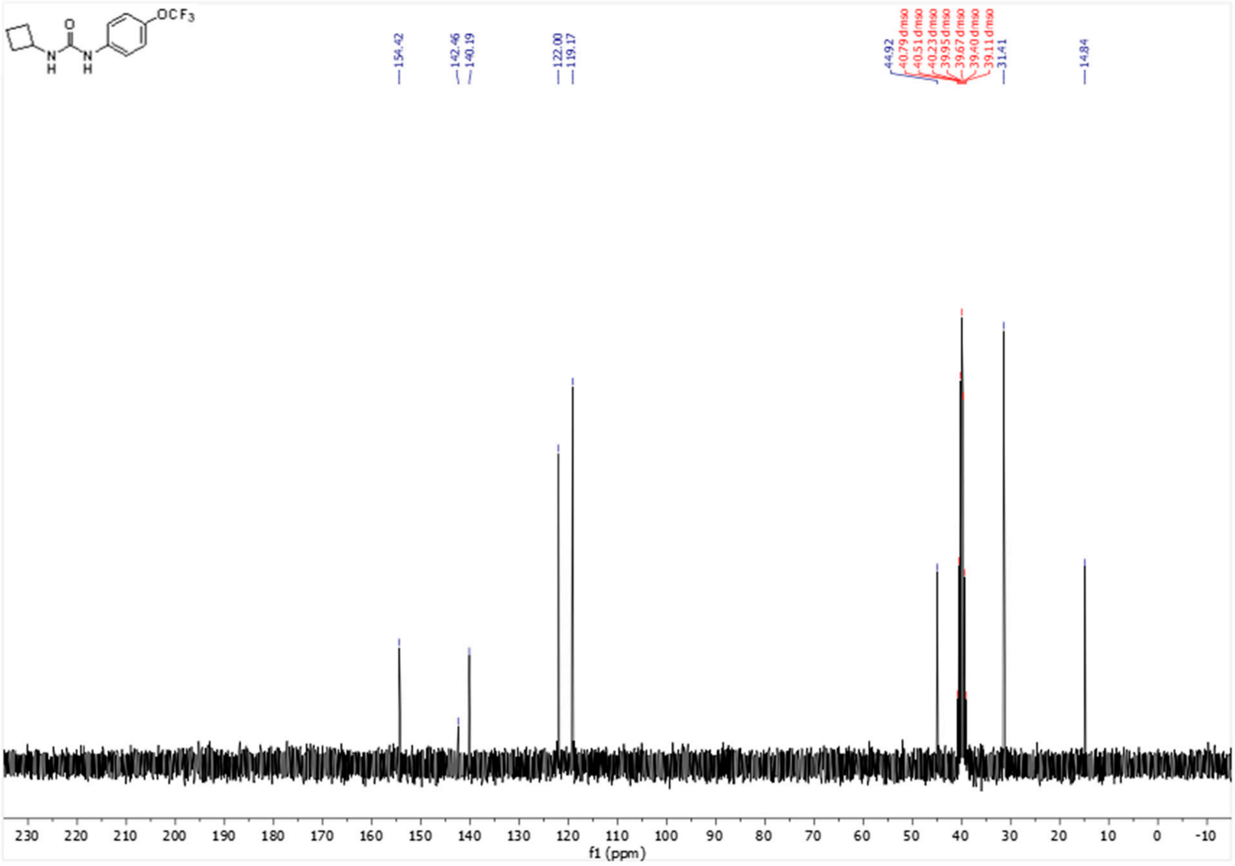

Figure S22 : ^{13}C NMR Spectrum of compound **(6b)**

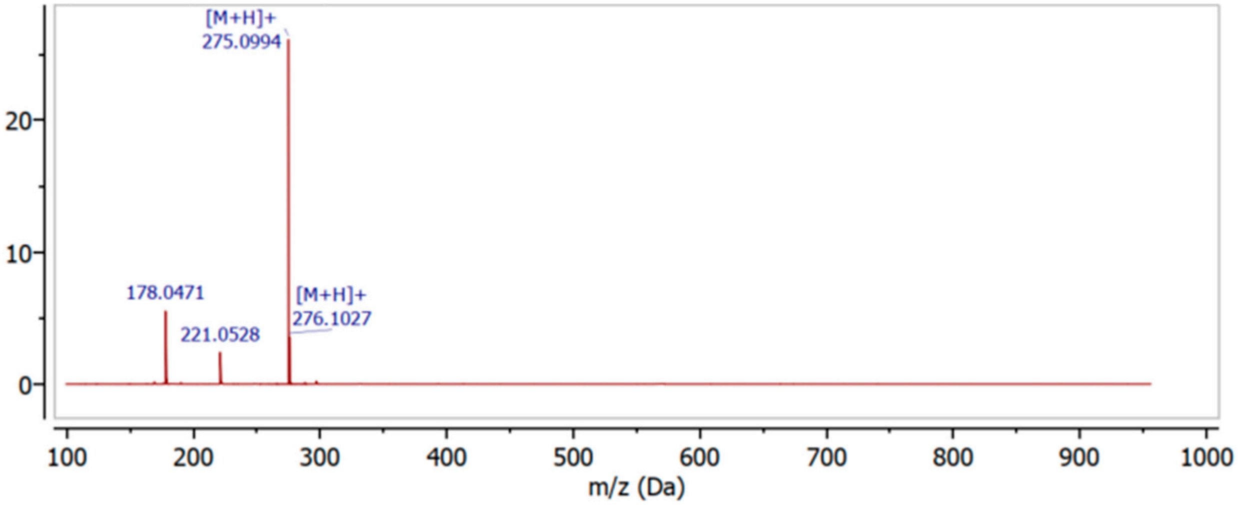

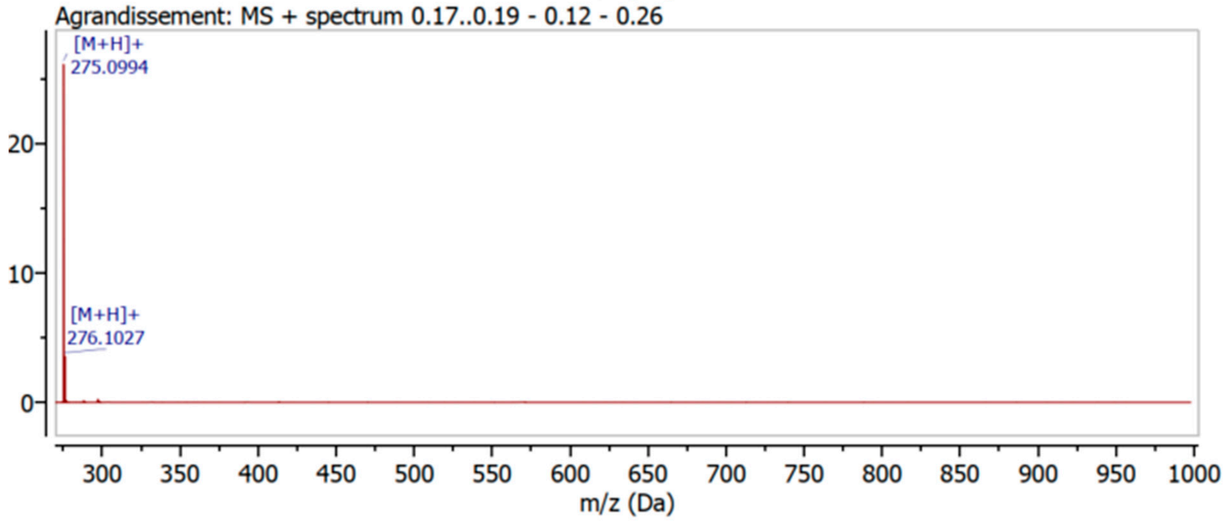

Composé trouvé: C12H13F3N2O2

Masse mesurée	Masse attendue	Intensité	Erreur (ppm)	Erreur (Da)	Ion identifié	Formule confirmée
275.0994	275.1002	306515373	-2.9	-0.0008	[M+H]+	C12H13F3N2O2
276.1027	276.1032	41299960	-2.0	-0.0006	[M+H]+	C12H13F3N2O2

Figure S23 : MS spectrum of compound **(6b)**

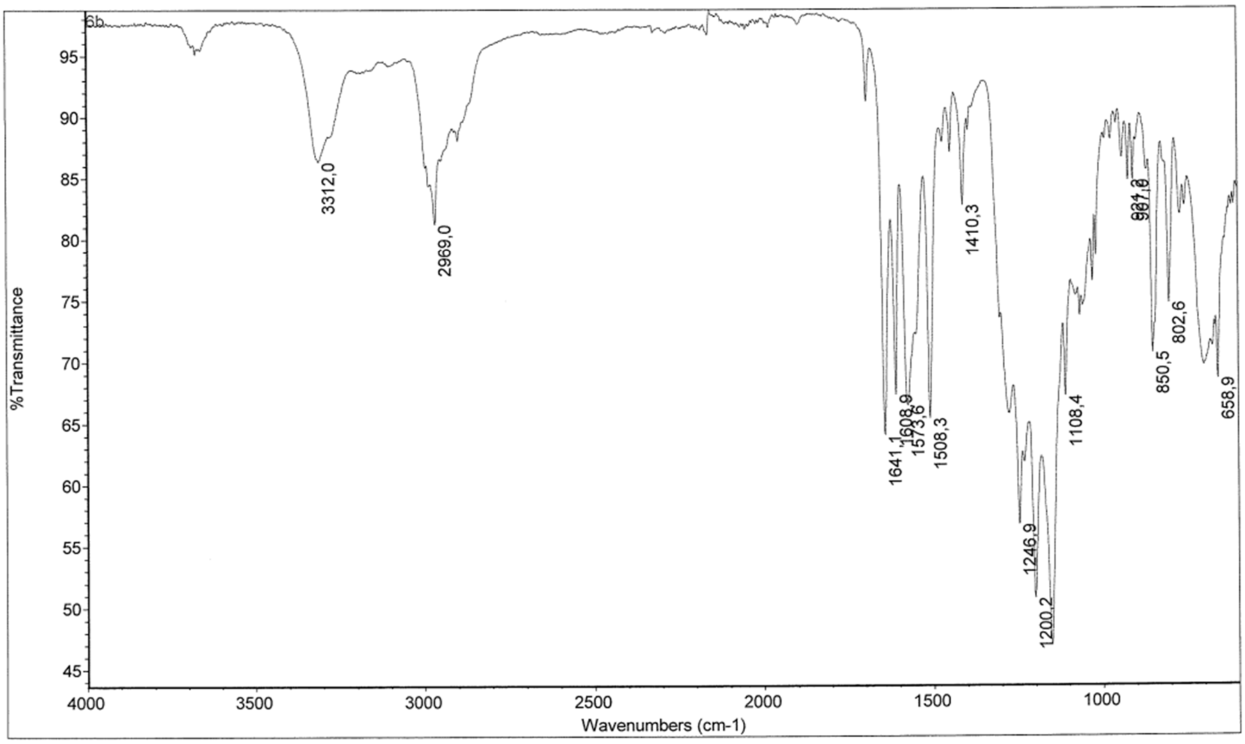

<u>Figure S24 :</u> IR spectrum of compound **(6b)**

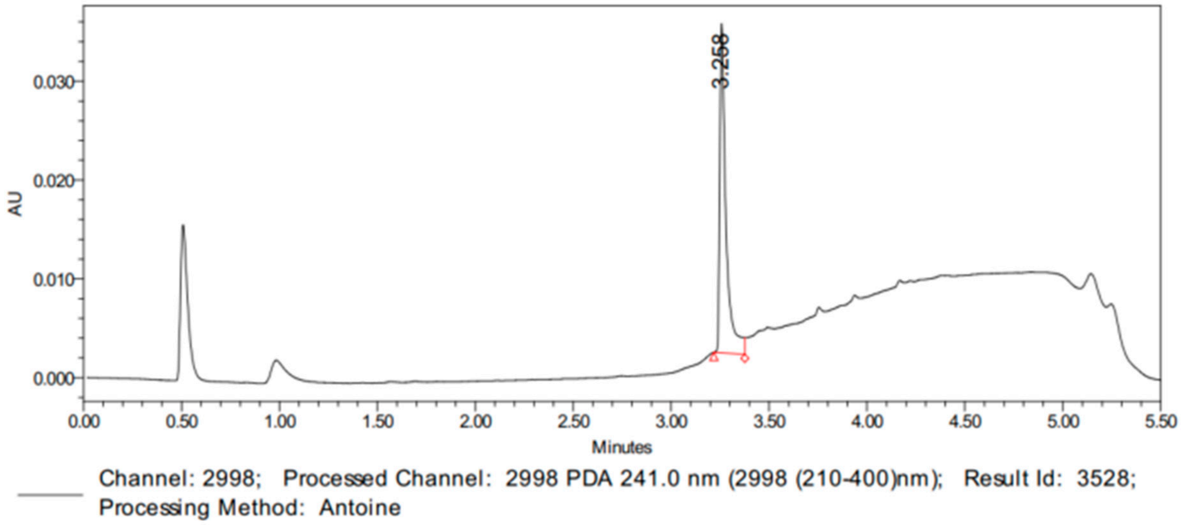

Processed Channel Descr.: 2998 PDA 241.0 nm (2998 (210-400)nm)

	Processed Channel Descr.	RT	Area	Height	% Height
1	2998 PDA 241.0 nm (2998 (210-400)nm)	3.258	73855	33317	100.00

<u>Figure S25 :</u> LC chromatogram of compound **(6b)**

Compound 7b

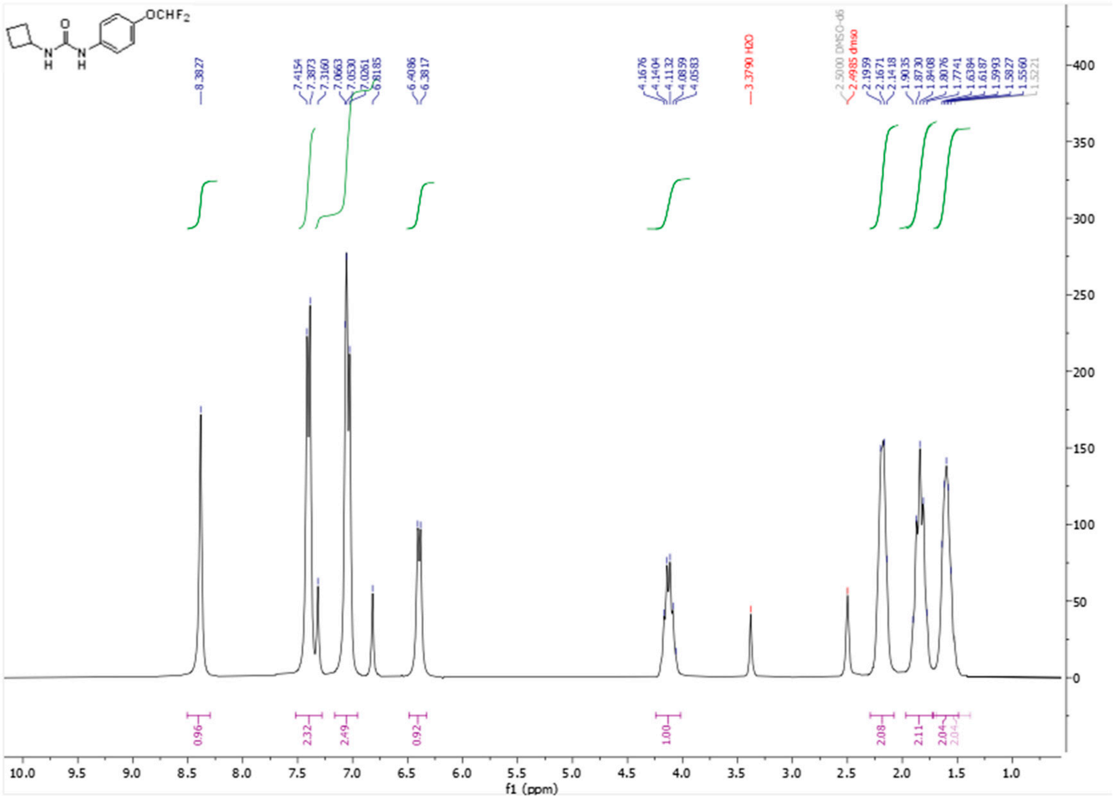

Figure S26 : ^{1}H NMR Spectrum of compound **(7b)**

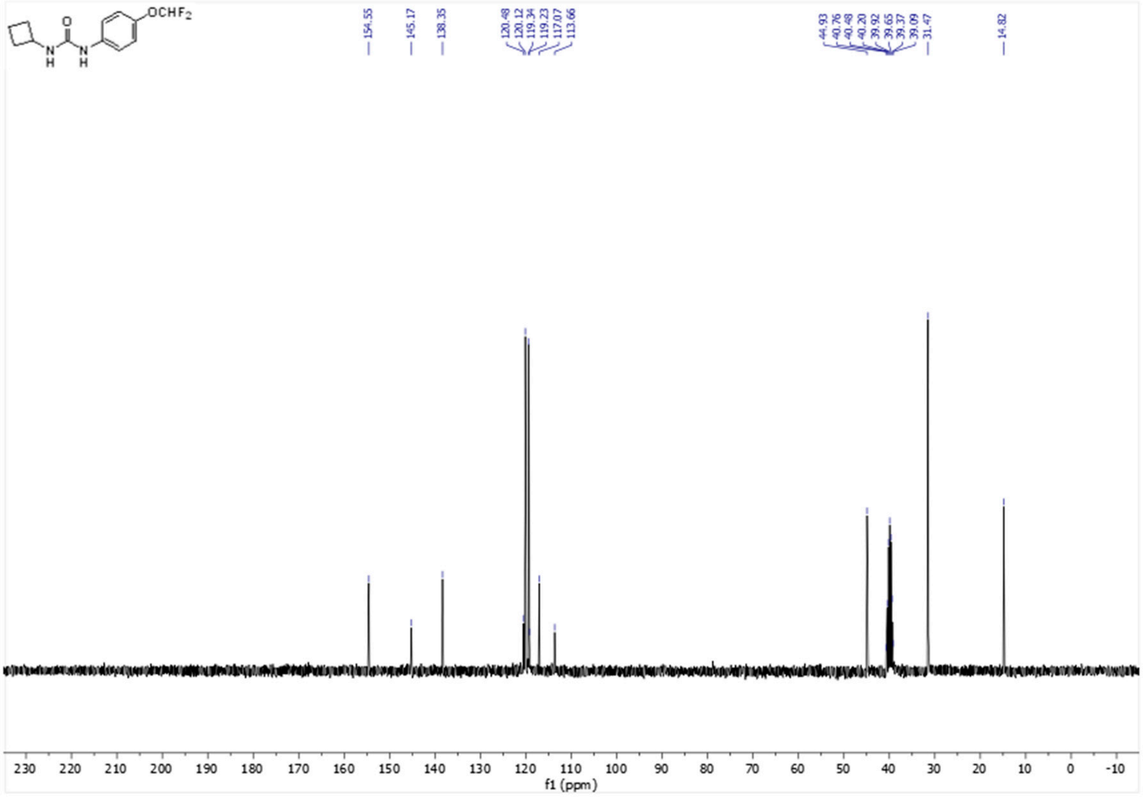

Figure S27 : ^{13}C NMR Spectrum of compound **(7b)**

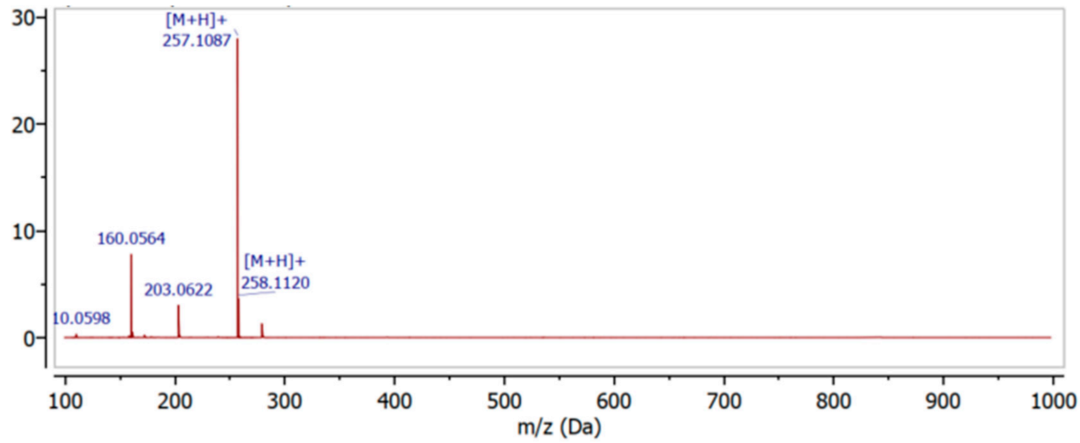

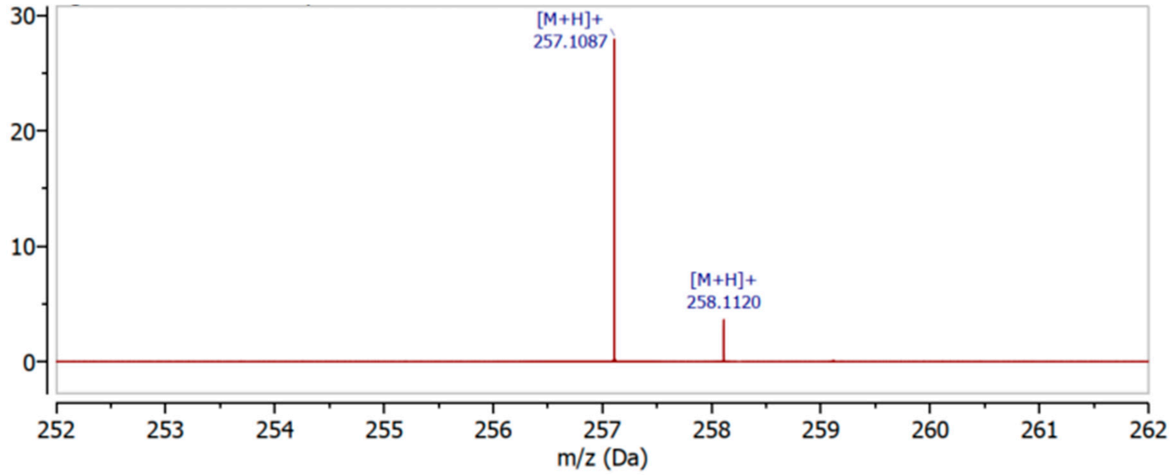

Composé trouvé: C12H14F2N2O2

Masse mesurée	Masse attendue	Intensité	Erreur (ppm)	Erreur (Da)	Ion identifié	Formule confirmée
257.1087	257.1096	415027505	-3.6	-0.0009	[M+H]+	C12H14F2N2O2
258.1120	258.1127	54775191	-2.8	-0.0007	[M+H]+	C12H14F2N2O2
279.0907	279.0916	20535194	-3.2	-0.0009	[M+Na]+	C12H14F2N2O2

<u>Figure S28 :</u> MS spectrum of compound **(7b)**

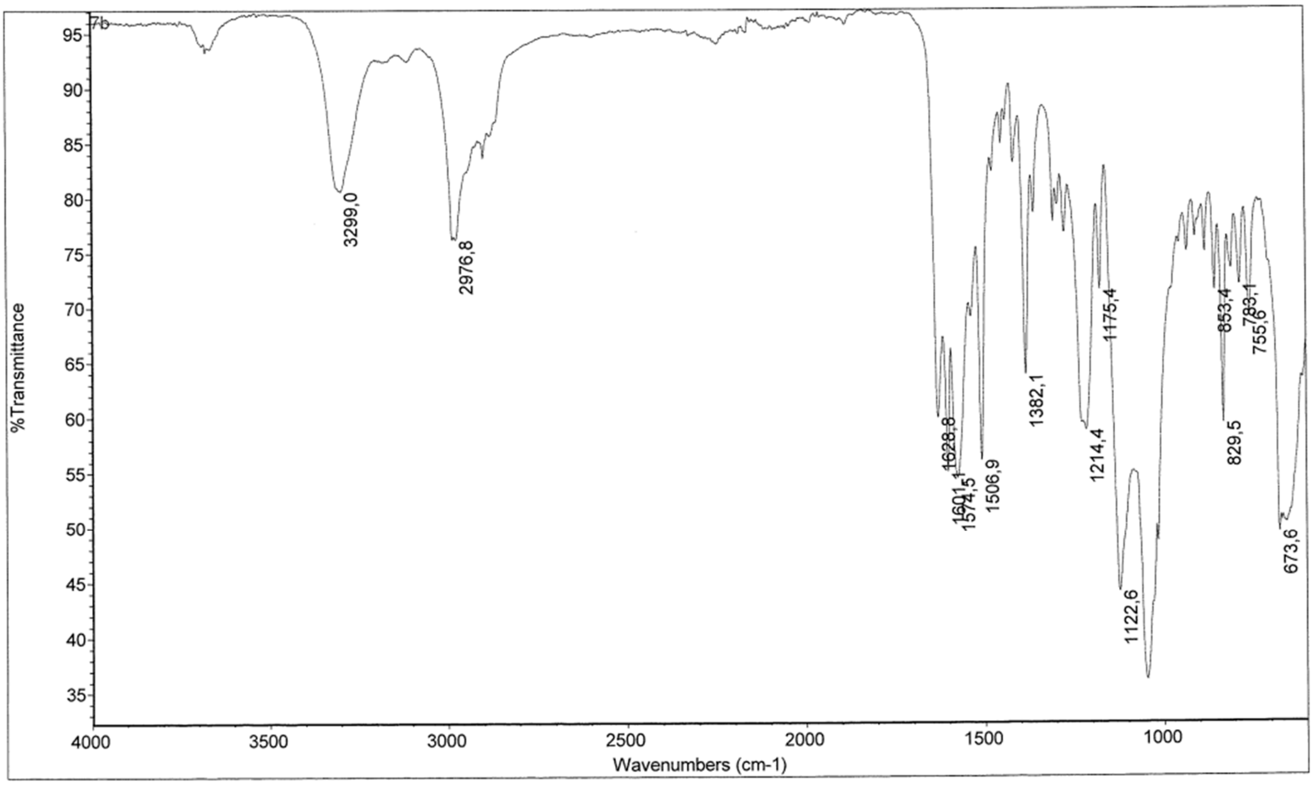

Figure S29 : IR spectrum of compound **(7b)**

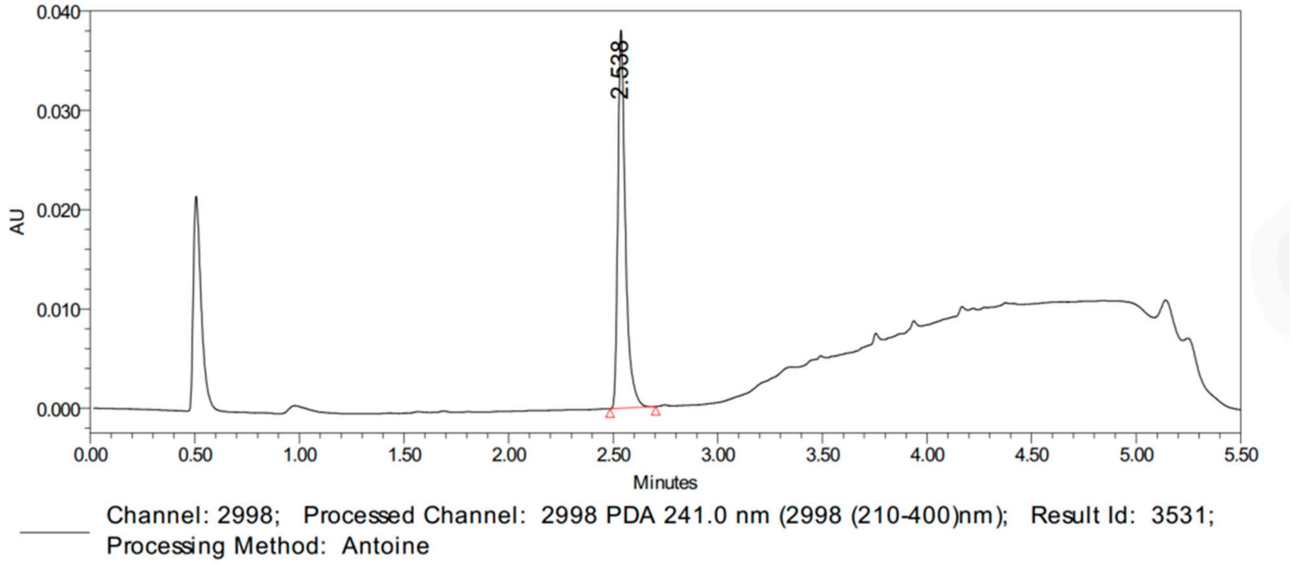

Processed Channel Descr.: 2998 PDA 241.0 nm (2998 (210-400)nm)

	Processed Channel Descr.	RT	Area	Height	% Height
1	2998 PDA 241.0 nm (2998 (210-400)nm)	2.538	94981	38129	100.00

Figure S30 : LC chromatogram of compound **(7b)**

Compound 8b

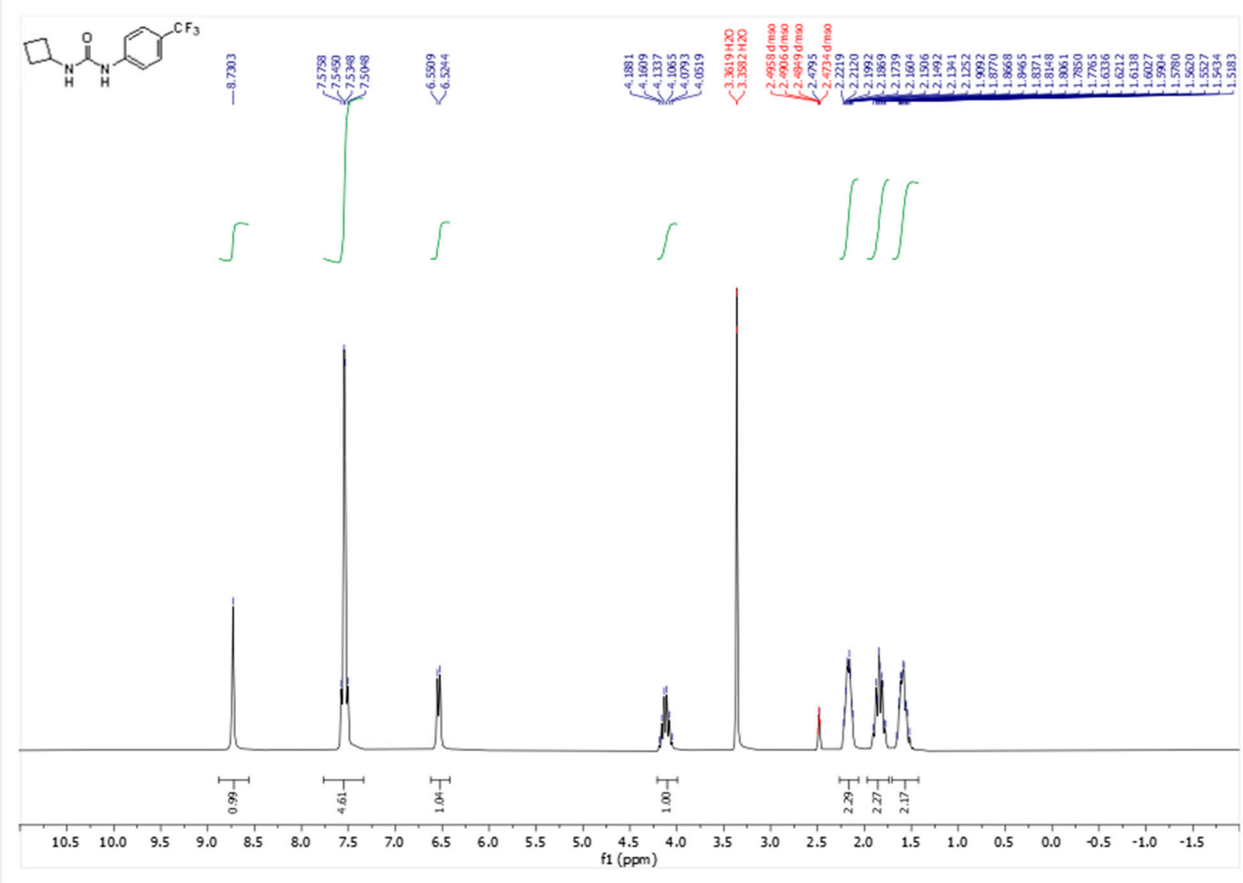

Figure S31 : ^{1}H NMR Spectrum of compound **(8b)**

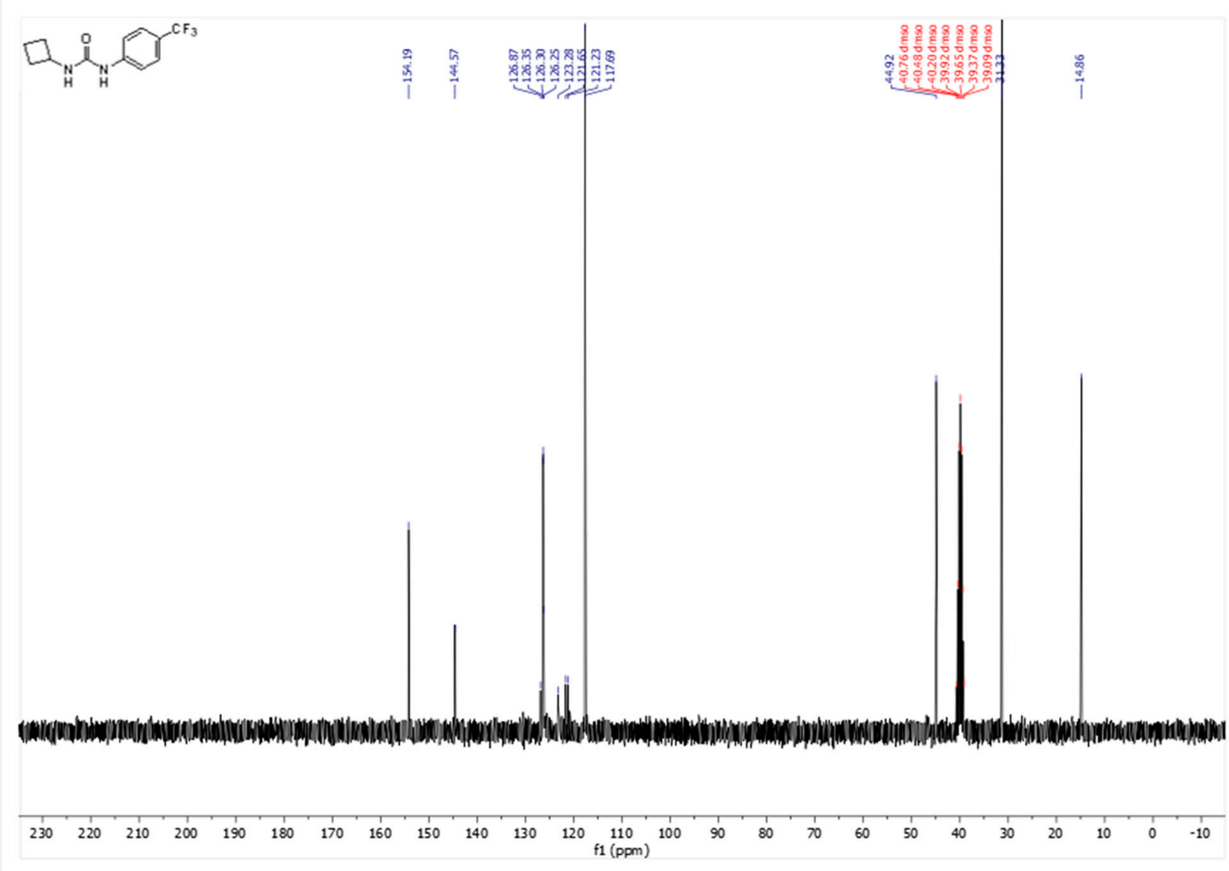

Figure S32 : ^{13}C NMR Spectrum of compound **(8b)**

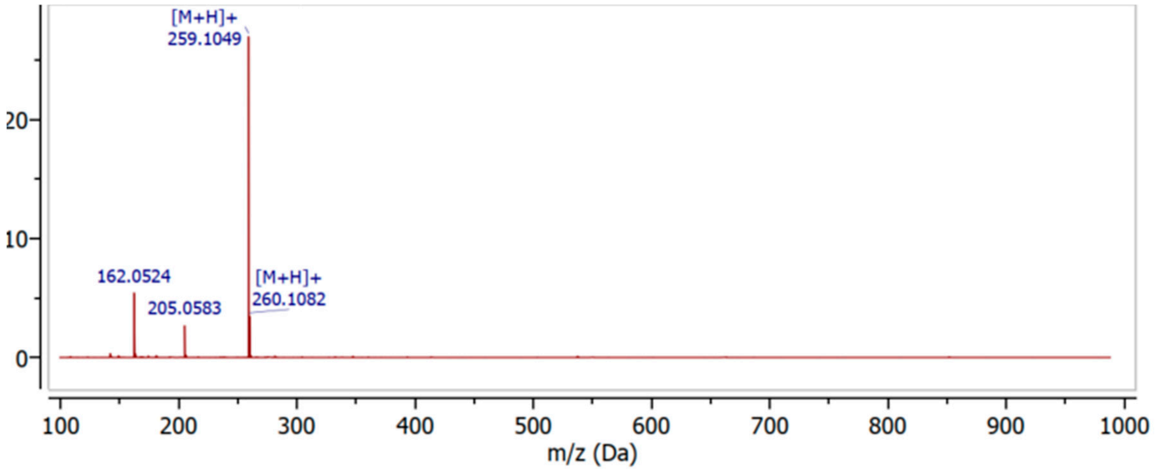

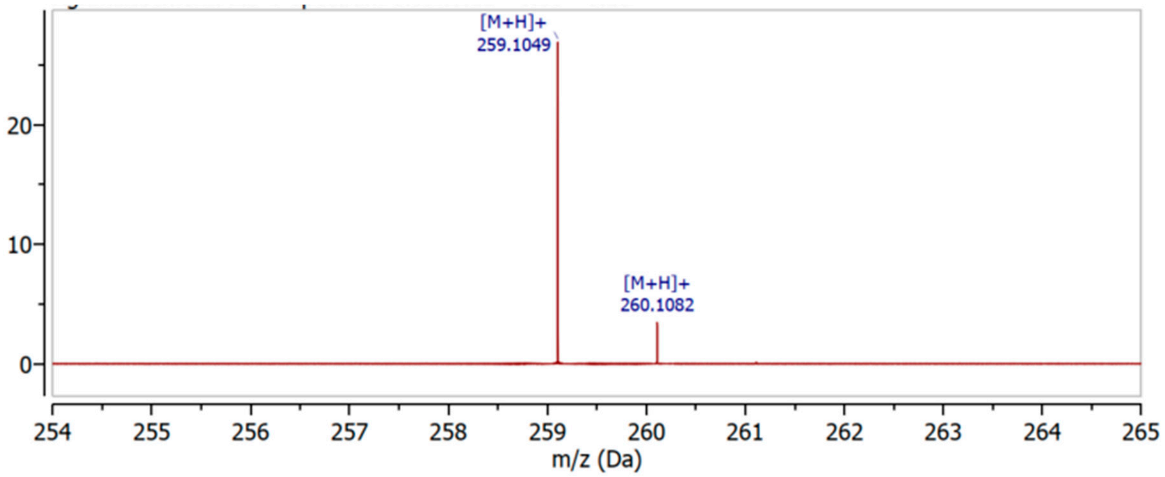

Composé trouvé: C12H13F3N2O

Masse mesurée	Masse attendue	Intensité	Erreur (ppm)	Erreur (Da)	Ion identifié	Formule confirmée
259.1049	259.1053	55570155	-1.4	-0.0004	[M+H]+	C12H13F3N2O
260.1082	260.1083	7302749	-0.4	-0.0001	[M+H]+	C12H13F3N2O

Figure S33 : MS spectrum of compound **(8b)**

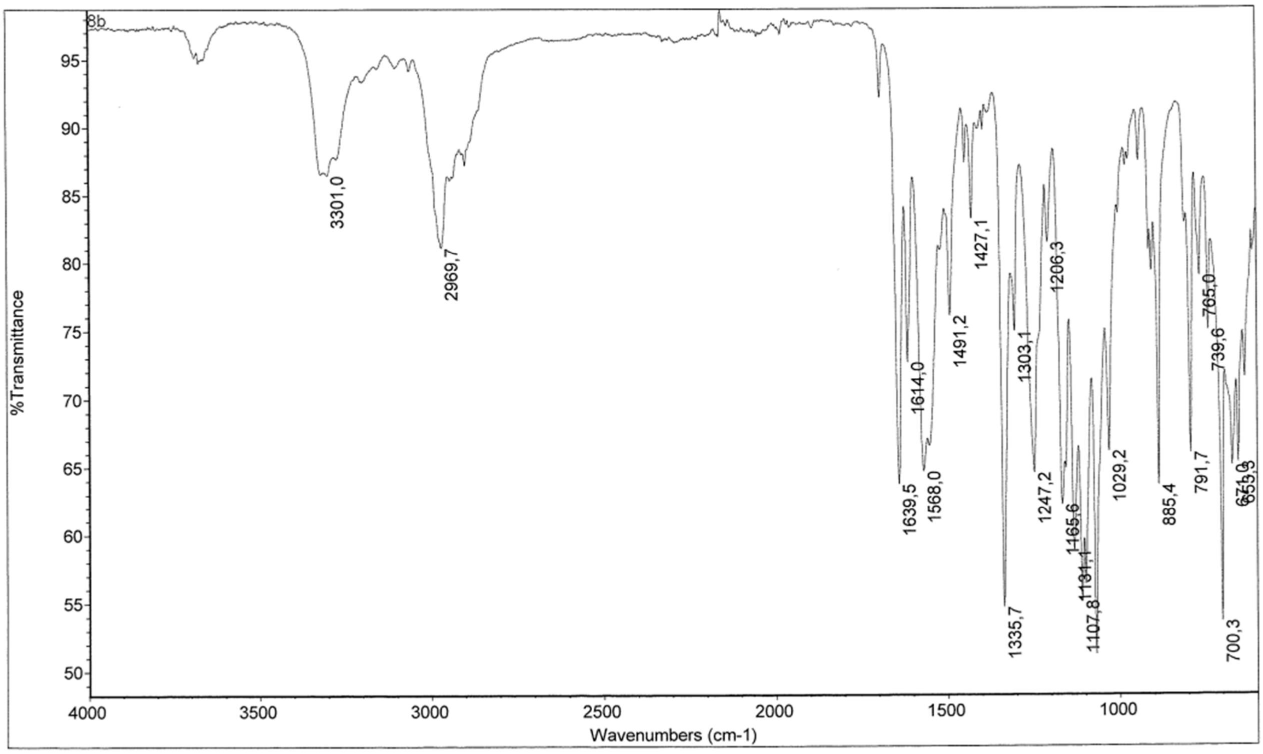

Figure S34 : IR spectrum of compound **(8b)**

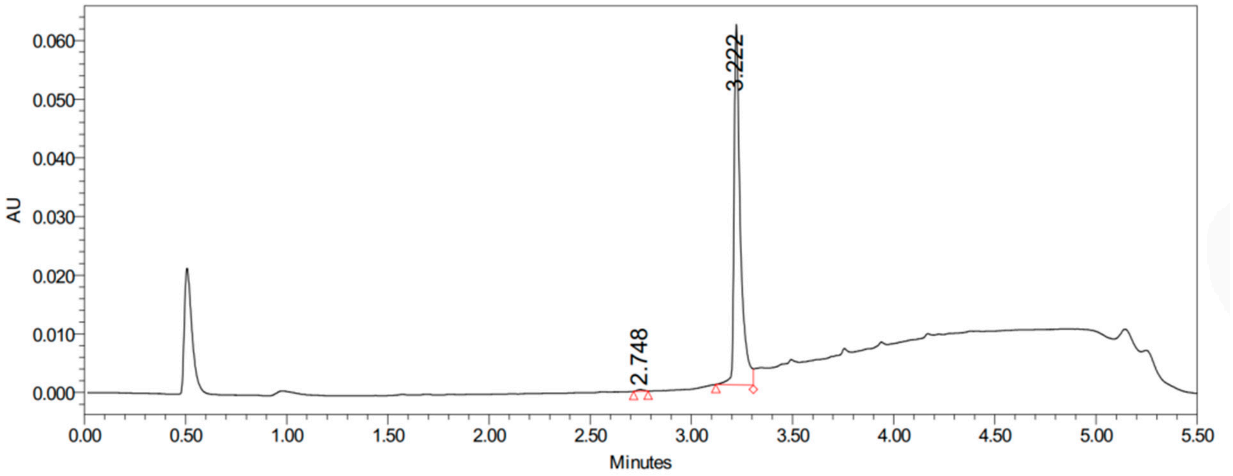

Channel: 2998; Processed Channel: 2998 PDA 241.0 nm (2998 (210-400)nm); Result Id: 3546; Processing Method: Antoine

Processed Channel Descr.: 2998 PDA 241.0 nm (2998 (210-400)nm)

	Processed Channel Descr.	RT	Area	Height	% Height
1	2998 PDA 241.0 nm (2998 (210-400)nm)	2.748	593	278	0.45
2	2998 PDA 241.0 nm (2998 (210-400)nm)	3.222	131585	61453	99.55

Figure S35 : LC chromatogram of compound **(8b)**

Compound 9b

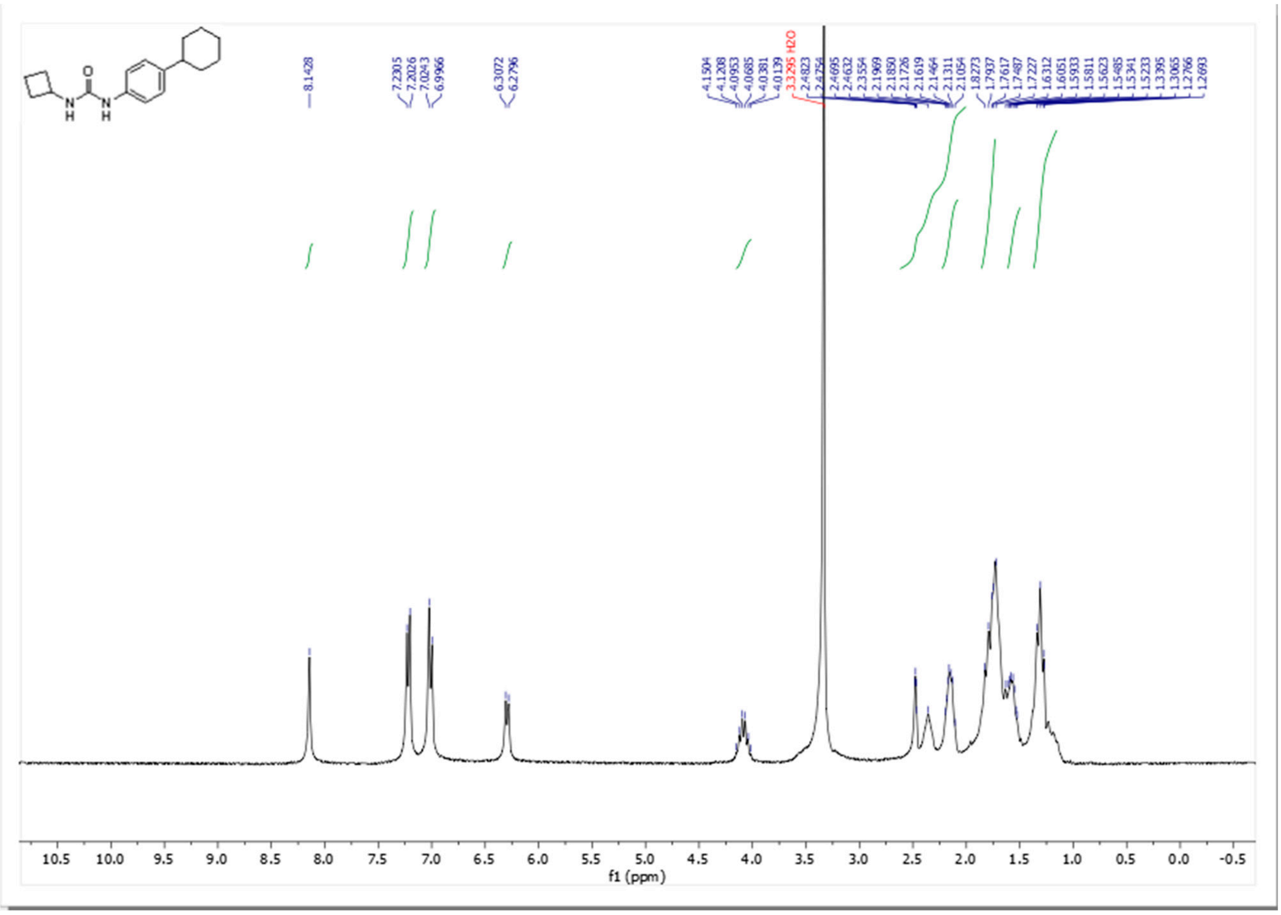

Figure S36 : ^{1}H NMR Spectrum of compound **(9b)**

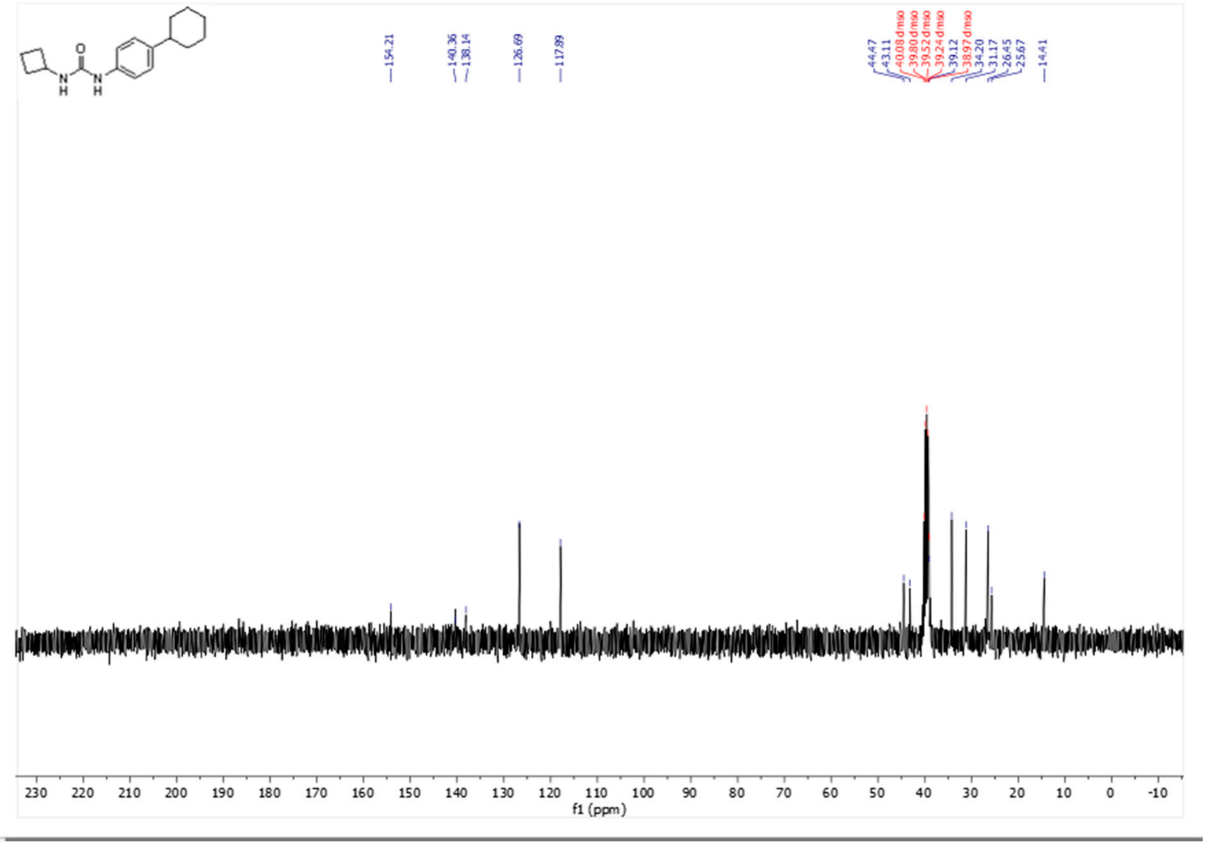

Figure S37 : ^{13}C NMR Spectrum of compound **(9b)**

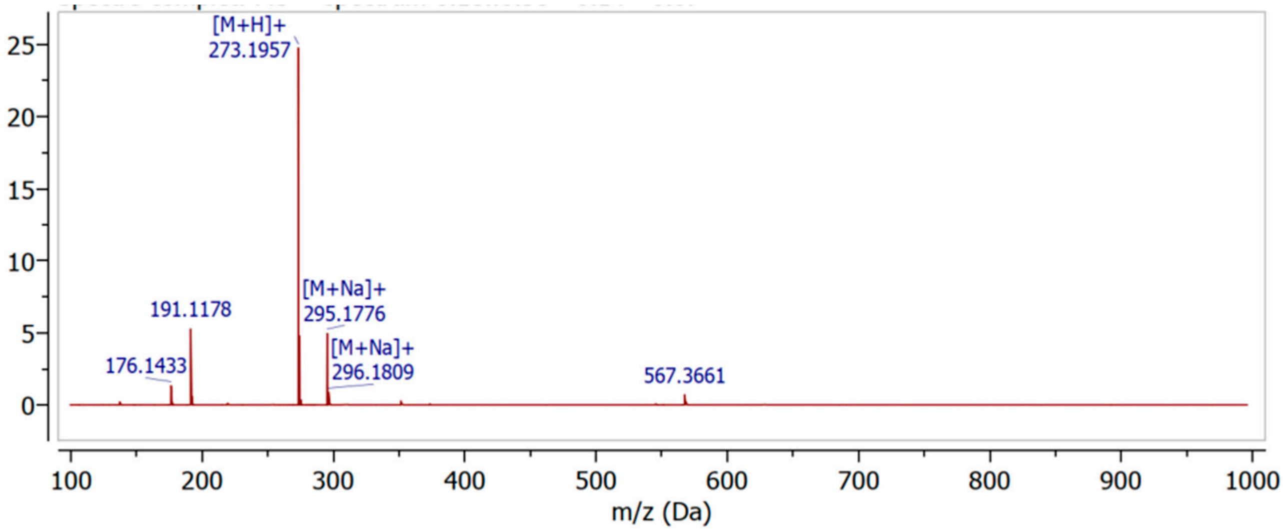

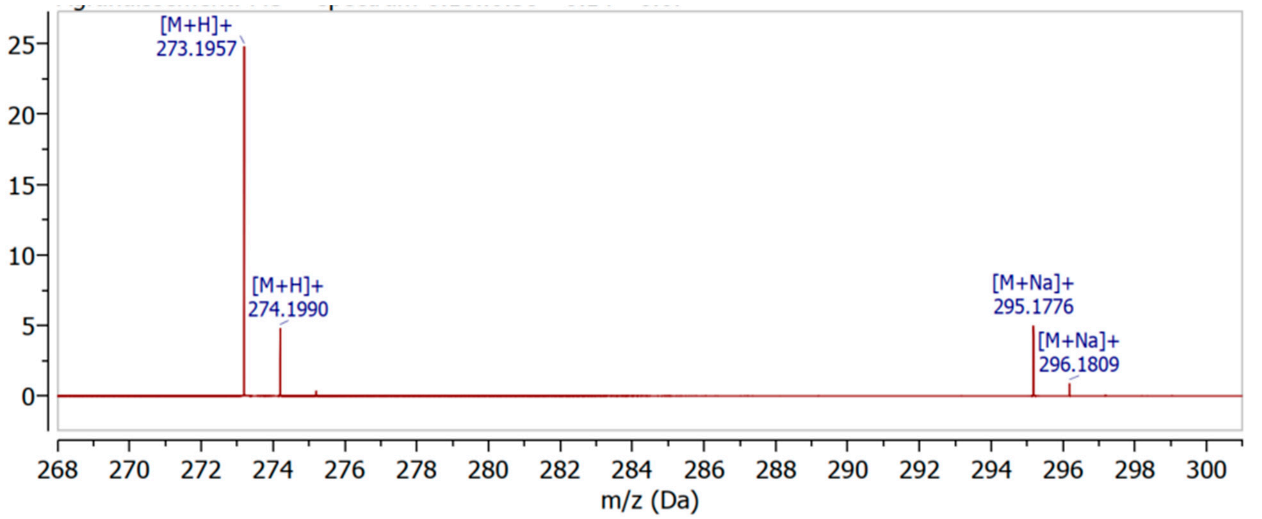

Composé trouvé: C17H24N2O

Masse mesurée	Masse attendue	Intensité	Erreur (ppm)	Erreur (Da)	Ion identifié	Formule confirmée
273.1957	273.1961	4162997004	-1.6	-0.0004	[M+H]+	C17H24N2O
274.1990	274.1993	777917594	-1.0	-0.0003	[M+H]+	C17H24N2O
295.1776	295.1781	791099097	-1.8	-0.0005	[M+Na]+	C17H24N2O
296.1809	296.1812	143795054	-1.1	-0.0003	[M+Na]+	C17H24N2O

Figure S38 : MS spectrum of compound **(9b)**

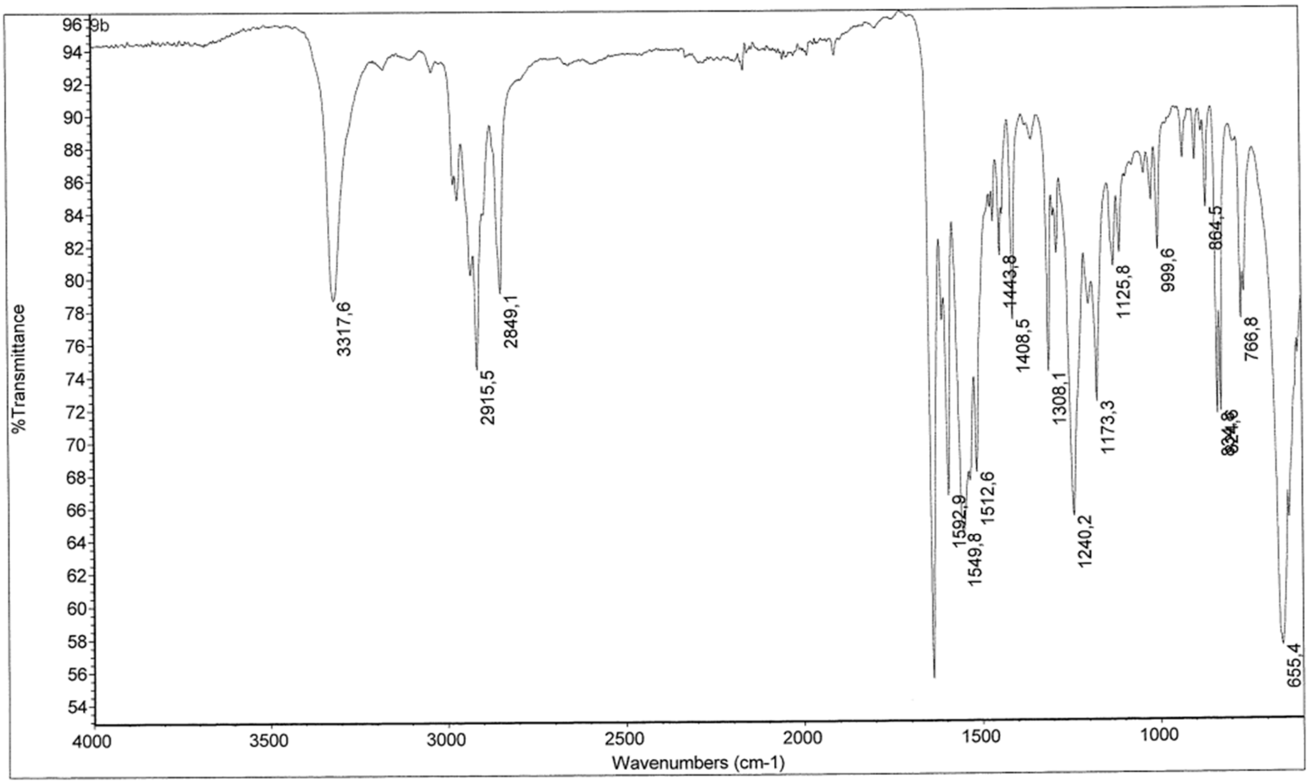

Figure S39 : IR spectrum of compound **(9b)**

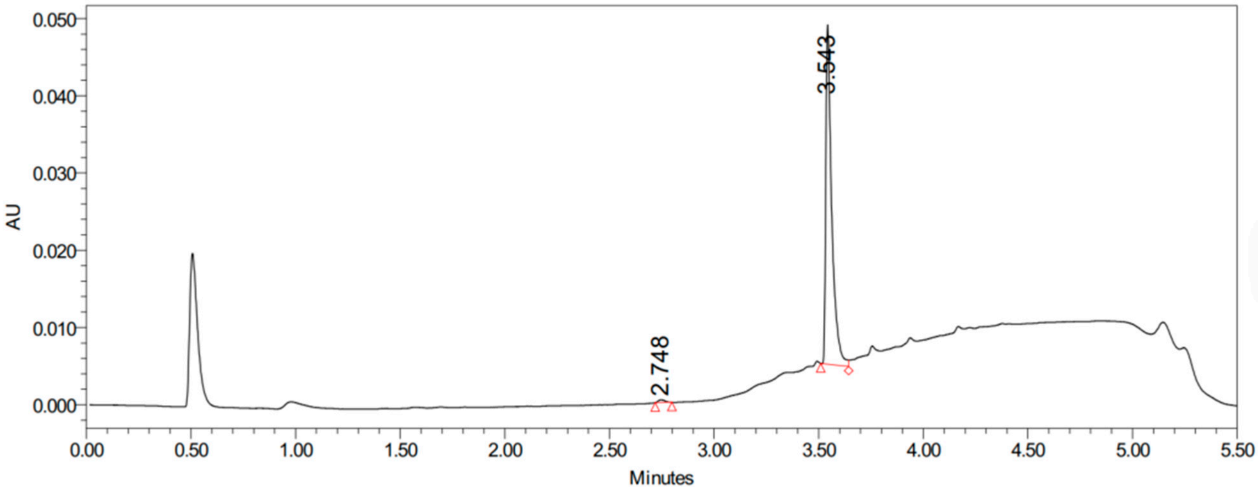

Channel: 2998; Processed Channel: 2998 PDA 241.0 nm (2998 (210-400)nm); Result Id: 3583; Processing Method: Antoine

Processed Channel Descr.: 2998 PDA 241.0 nm (2998 (210-400)nm)

	Processed Channel Descr.	RT	Area	Height	% Height
1	2998 PDA 241.0 nm (2998 (210-400)nm)	2.748	742	346	0.78
2	2998 PDA 241.0 nm (2998 (210-400)nm)	3.543	87187	43916	99.22

Figure S40 : LC chromatogram of compound **(9b)**

Compound 10b

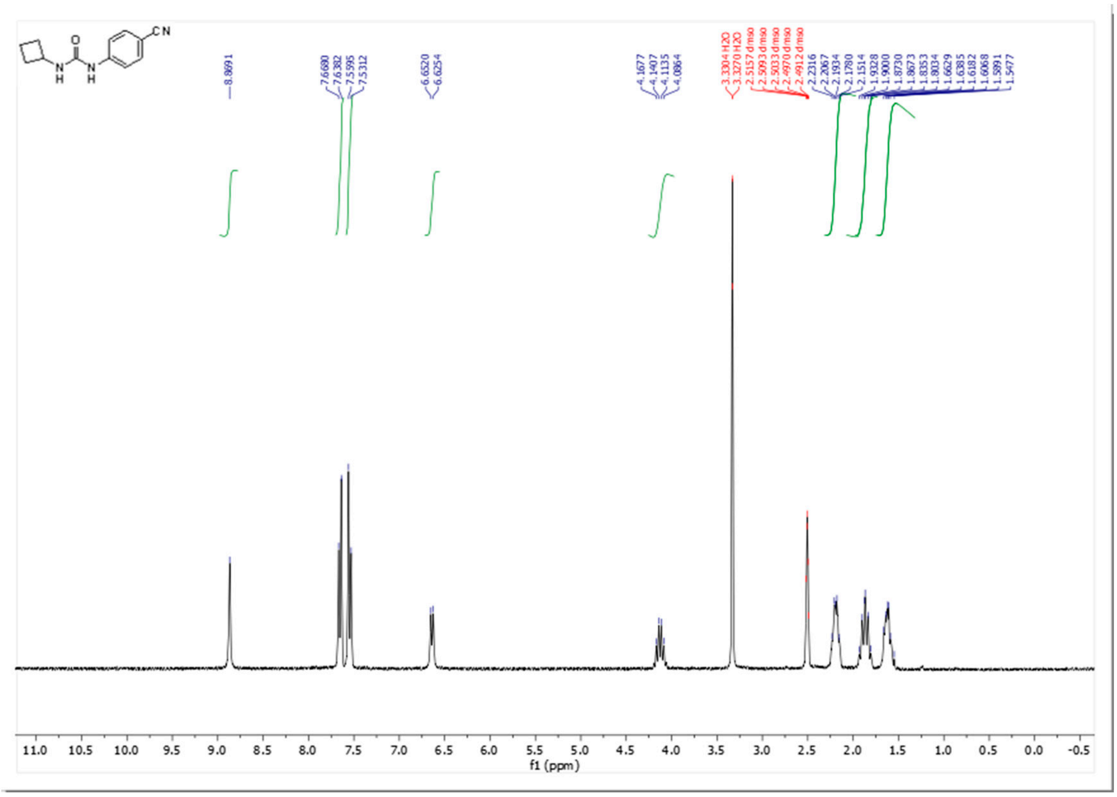

Figure S41 : ^{1}H NMR Spectrum of compound **(10b)**

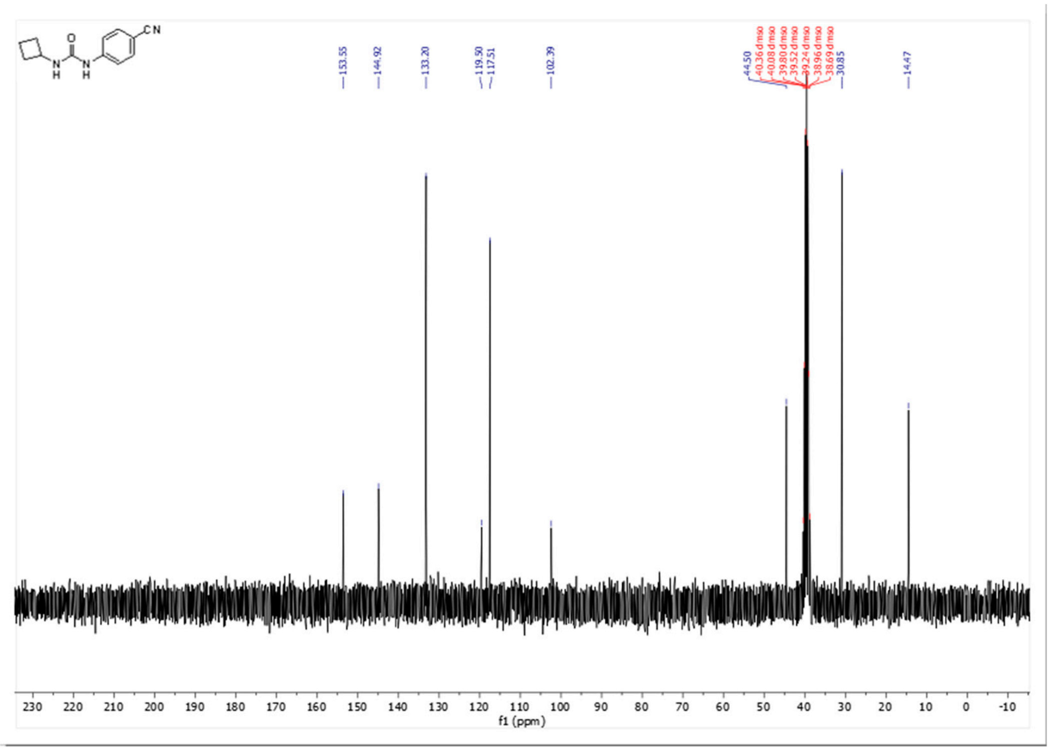

Figure S42 : ^{13}C NMR Spectrum of compound **(10b)**

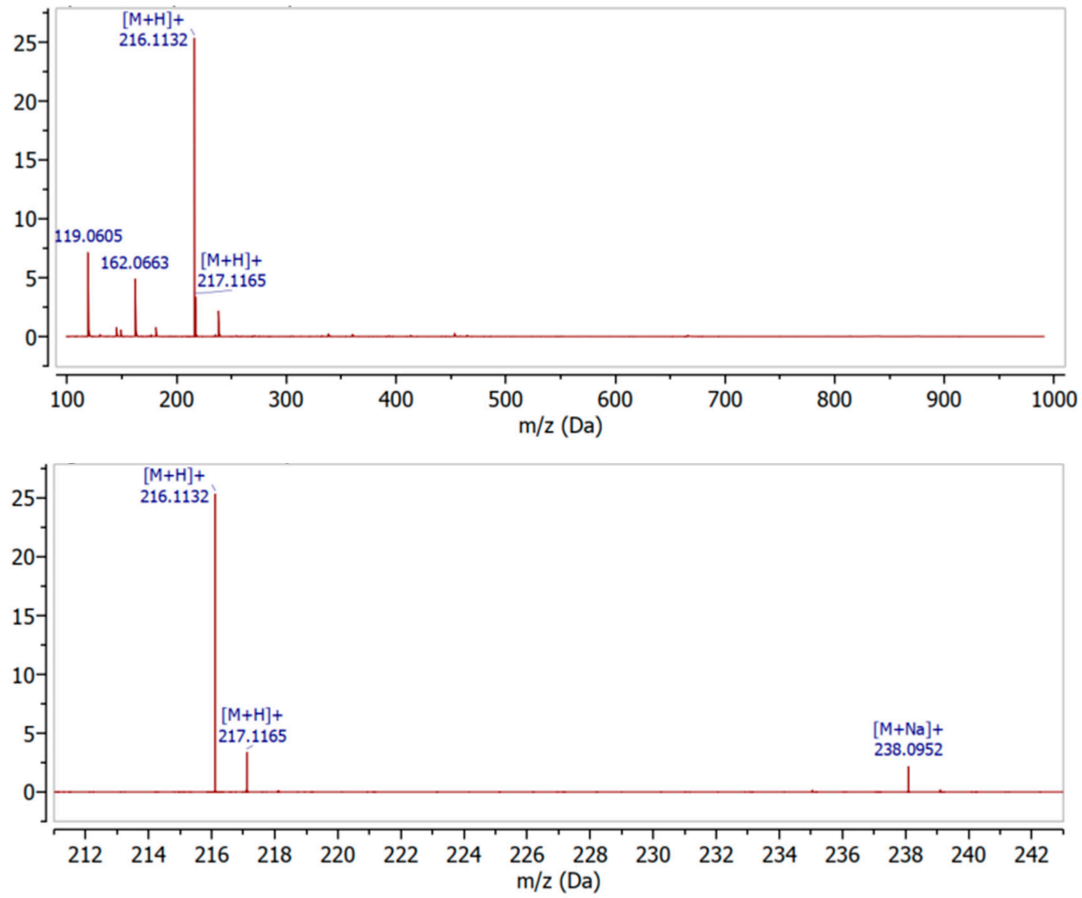

Composé trouvé: C12H13N3O

Masse mesurée	Masse attendue	Intensité	Erreur (ppm)	Erreur (Da)	Ion identifié	Formule confirmée
216.1132	216.1131	16530852	0.4	0.0001	[M+H]+	C12H13N3O
217.1165	217.1160	2137973	2.2	0.0005	[M+H]+	C12H13N3O
238.0952	238.0951	1405231	0.5	0.0001	[M+Na]+	C12H13N3O

Figure S43 : MS spectrum of compound **(10b)**

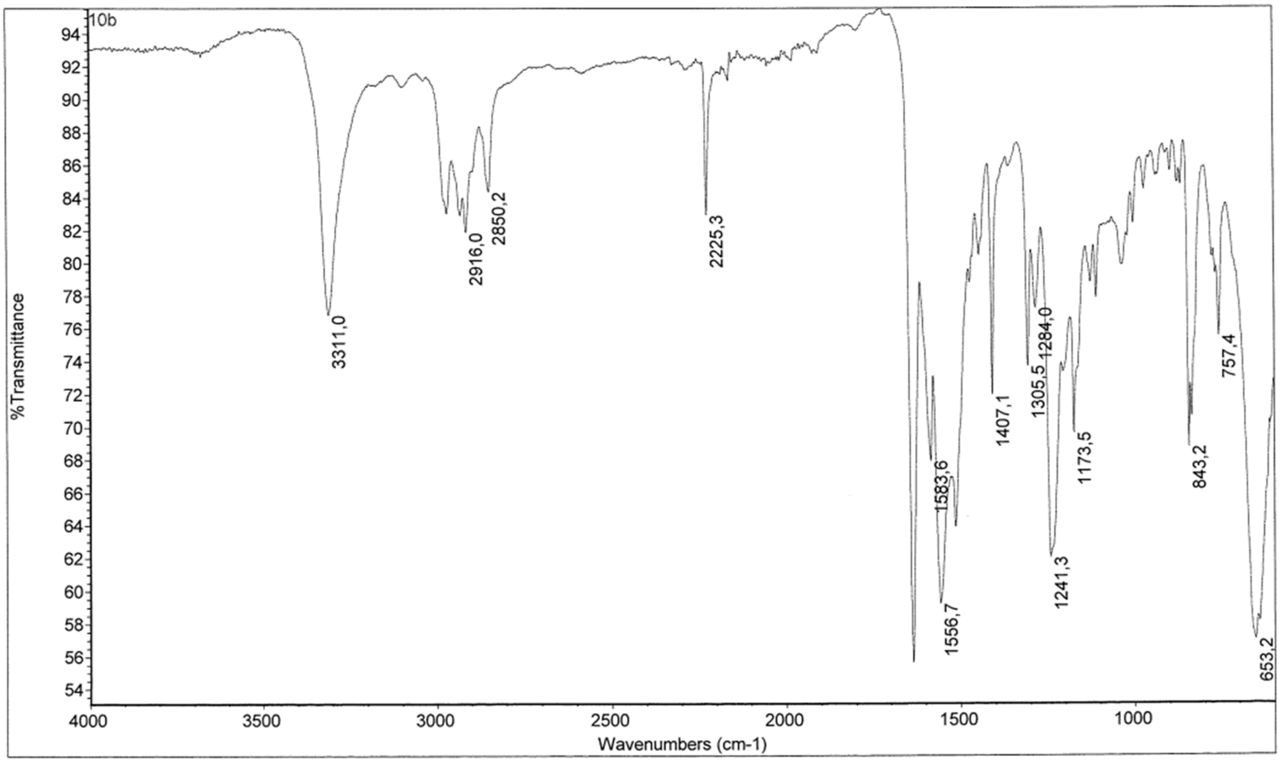

<u>Figure S44 :</u> IR spectrum of compound **(10b)**

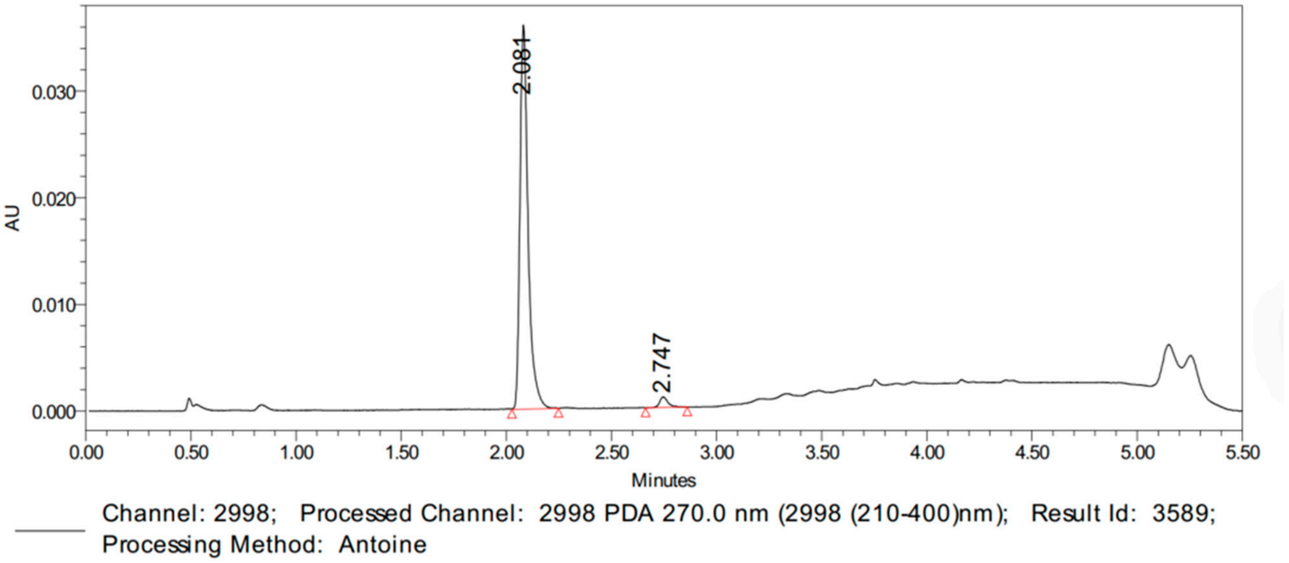

Processed Channel Descr.: 2998 PDA 270.0 nm (2998 (210-400)nm)

	Processed Channel Descr.	RT	Area	Height	% Height
1	2998 PDA 270.0 nm (2998 (210-400)nm)	2.081	95765	36023	97.32
2	2998 PDA 270.0 nm (2998 (210-400)nm)	2.747	2826	993	2.68

<u>Figure S45 :</u> LC chromatogram of compound **(10b)**

<u>Compound 11b</u>

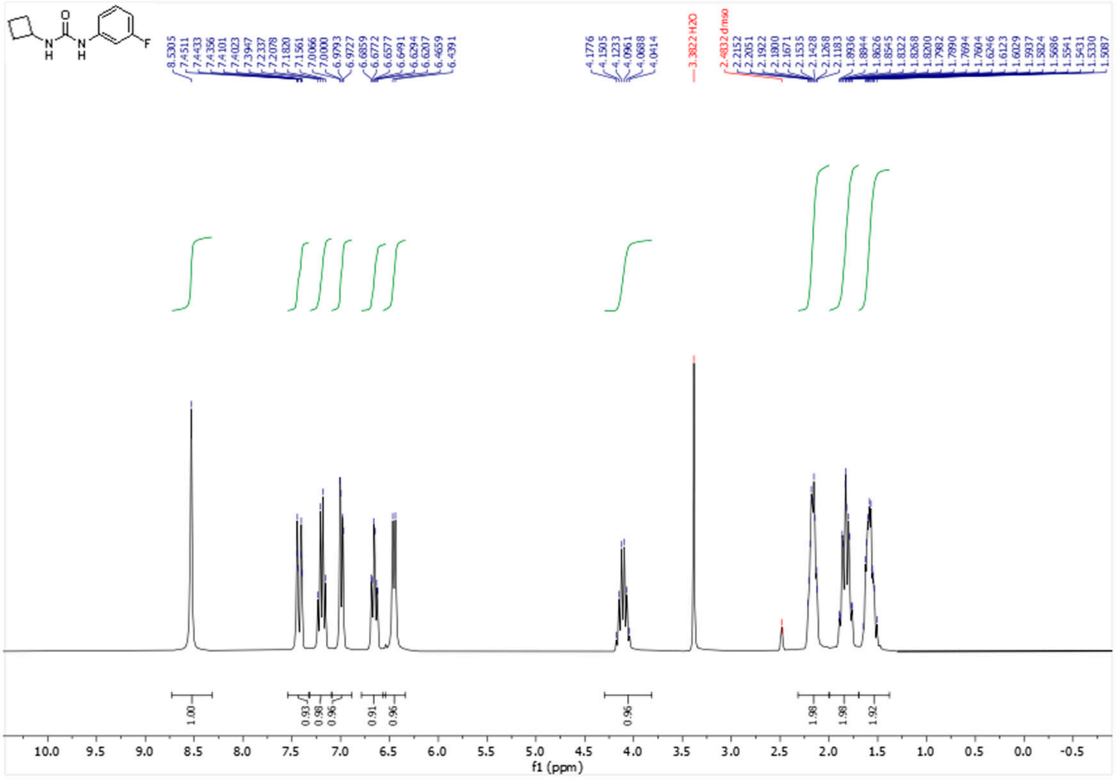

<u>Figure S46 :</u> ^{1}H NMR Spectrum of compound **(11b)**

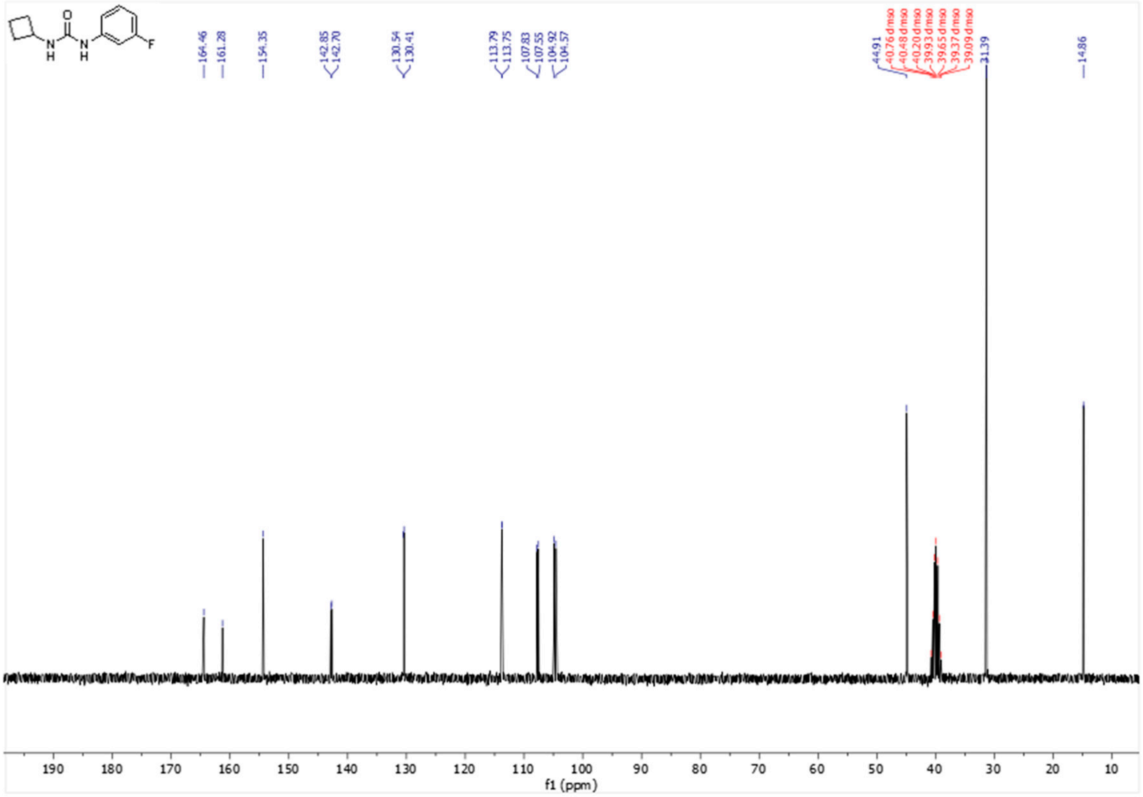

<u>Figure S47 :</u> ^{13}C NMR Spectrum of compound **(11b)**

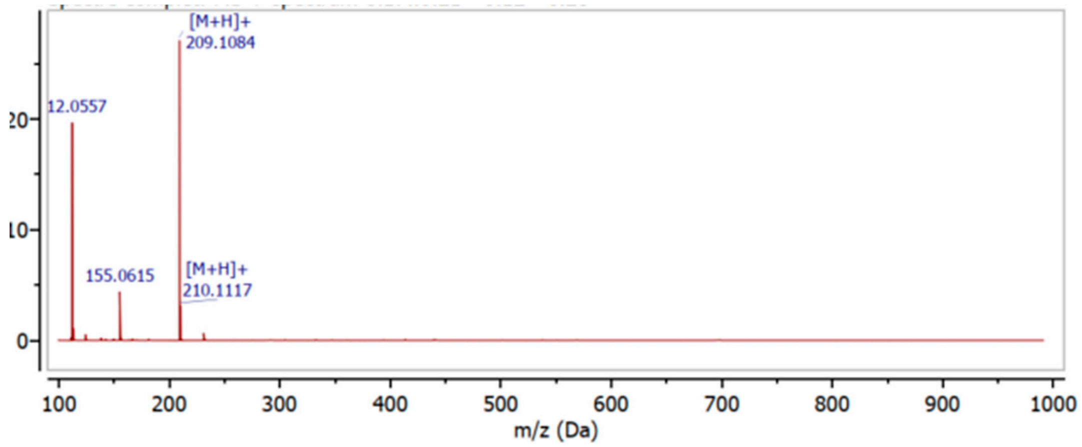

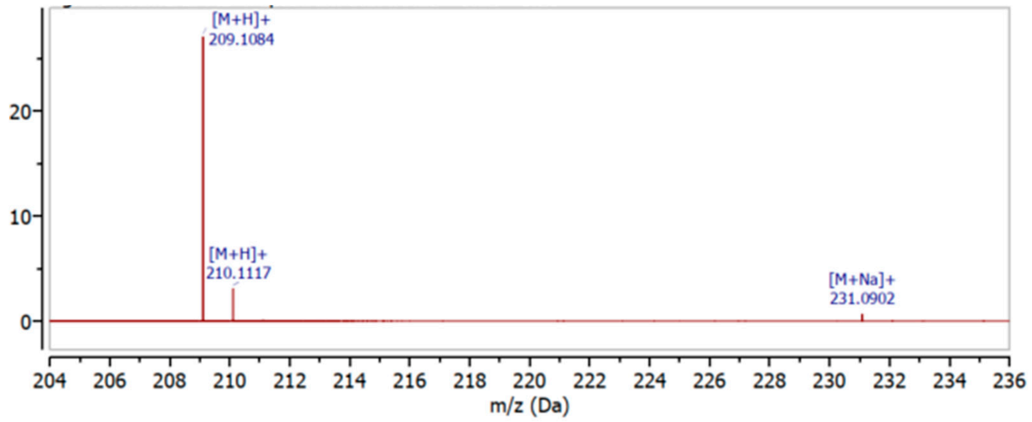

Composé trouvé: C11H13FN2O

Masse mesurée	Masse attendue	Intensité	Erreur (ppm)	Erreur (Da)	Ion identifié	Formule confirmée
209.1084	209.1085	109242215	-0.4	-0.0001	[M+H]+	C11H13FN2O
210.1117	210.1115	13001585	1.0	0.0002	[M+H]+	C11H13FN2O
231.0902	231.0904	2729037	-0.8	-0.0002	[M+Na]+	C11H13FN2O

Figure S48: MS spectrum of compound **(11b)**

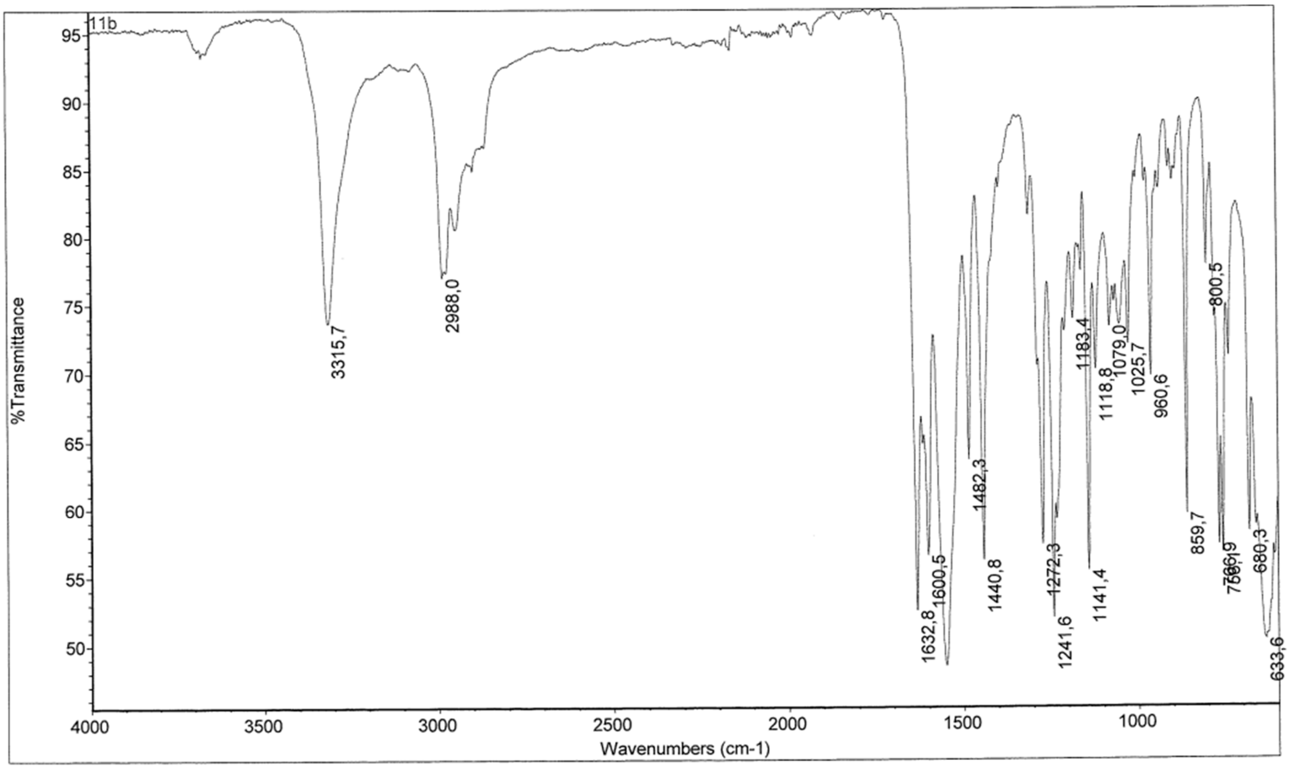

Figure S49 : IR spectrum of compound **(11b)**

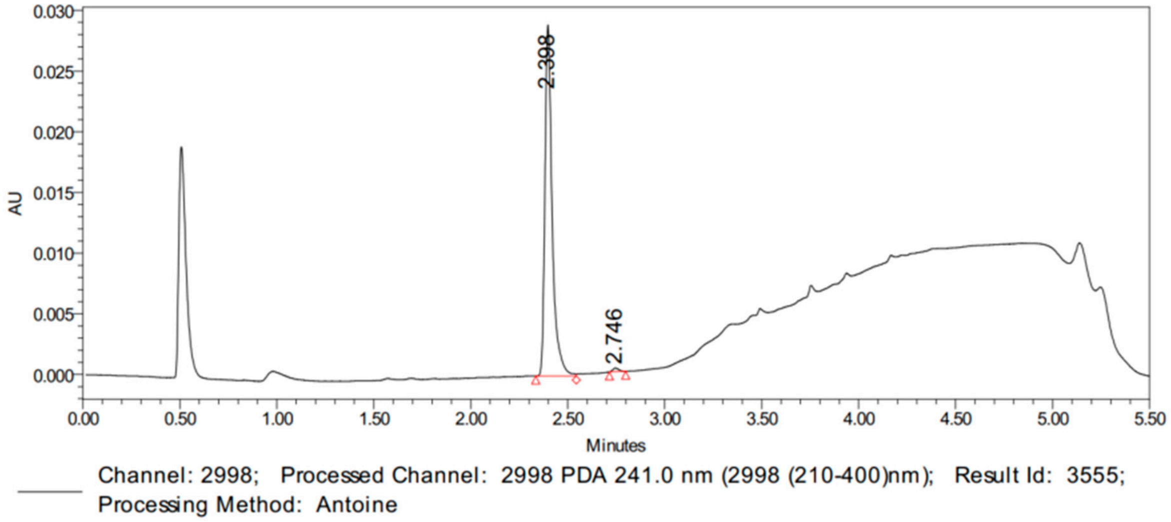

Processed Channel Descr.: 2998 PDA 241.0 nm (2998 (210-400)nm)

	Processed Channel Descr.	RT	Area	Height	% Height
1	2998 PDA 241.0 nm (2998 (210-400)nm)	2.398	75676	28944	98.99
2	2998 PDA 241.0 nm (2998 (210-400)nm)	2.746	626	294	1.01

Figure S50 : LC chromatogram of compound **(11b)**

Compound 12b

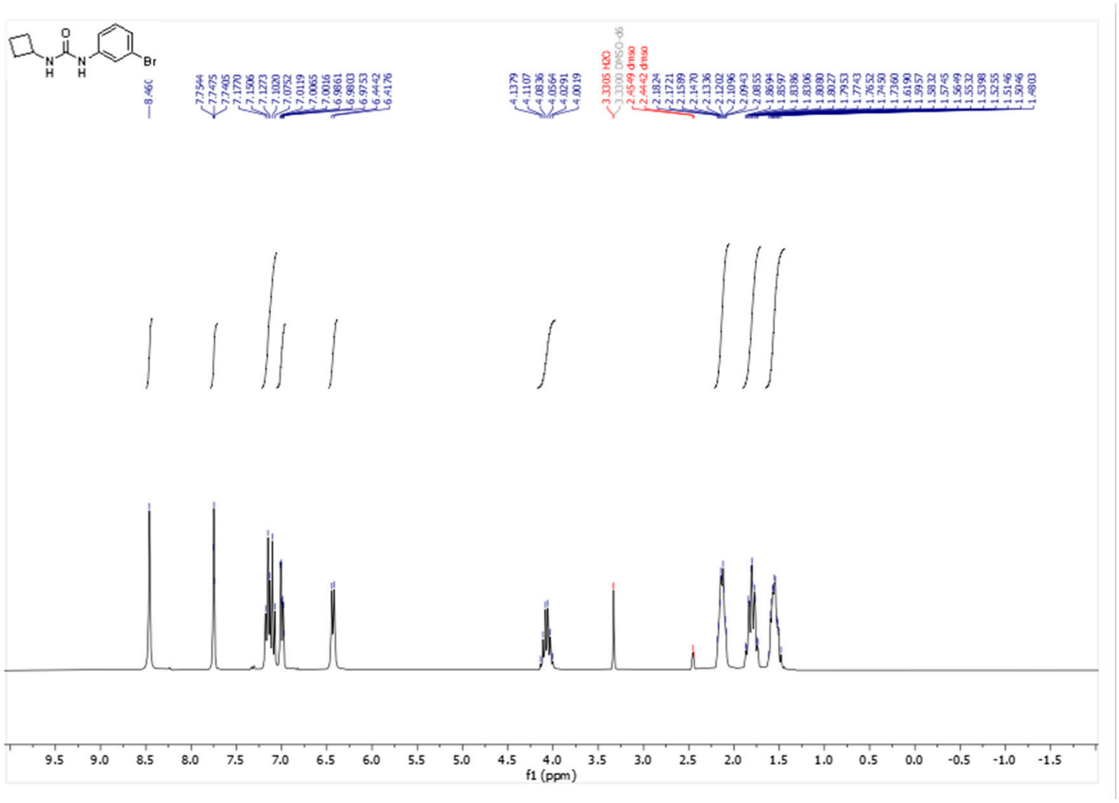

Figure S51 : ^{1}H NMR Spectrum of compound **(12b)**

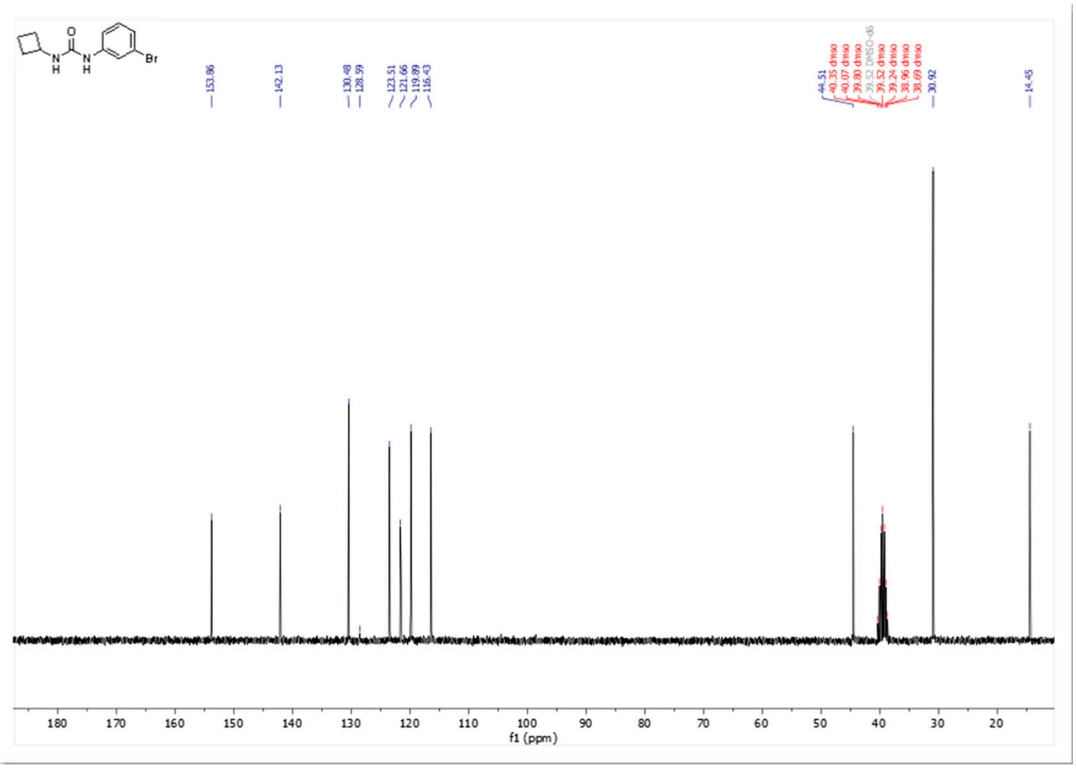

Figure S52 : ^{13}C NMR Spectrum of compound **(12b)**

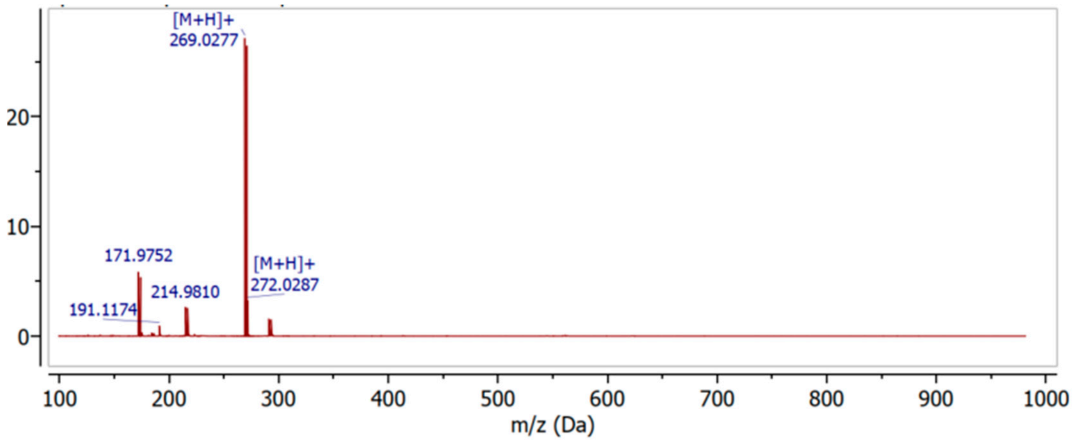

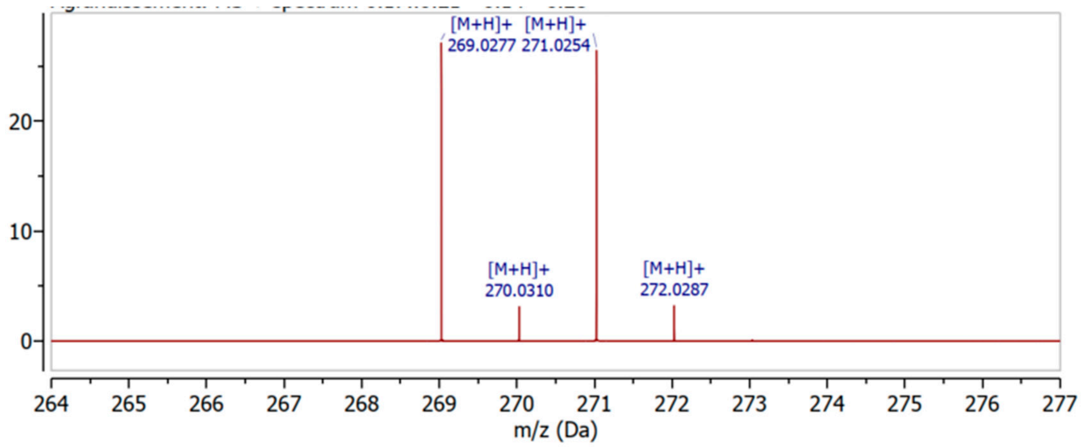

Composé trouvé: C11H13BrN2O

Masse mesurée	Masse attendue	Intensité	Erreur (ppm)	Erreur (Da)	Ion identifié	Formule confirmée
269.0277	269.0284	183028097	-2.7	-0.0007	[M+H]+	C11H13BrN2O
270.0310	270.0314	21370484	-1.6	-0.0004	[M+H]+	C11H13BrN2O
271.0254	271.0264	181179814	-3.7	-0.0010	[M+H]+	C11H13BrN2O
272.0287	272.0294	22009854	-2.5	-0.0007	[M+H]+	C11H13BrN2O
291.0094	291.0103	10434891	-3.3	-0.0010	[M+Na]+	C11H13BrN2O
293.0073	293.0084	10549615	-3.6	-0.0010	[M+Na]+	C11H13BrN2O

<u>Figure S53 :</u> MS spectrum of compound **(12b)**

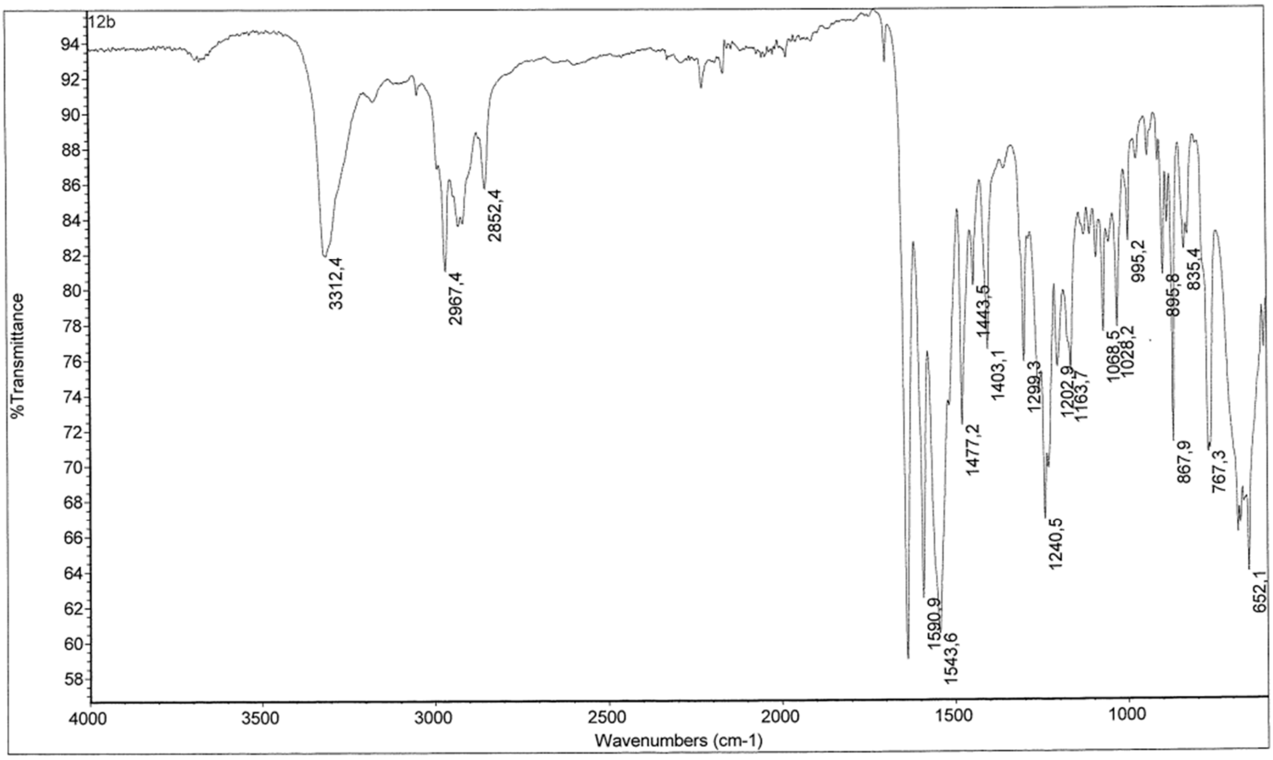

Figure S54 : IR spectrum of compound **(12b)**

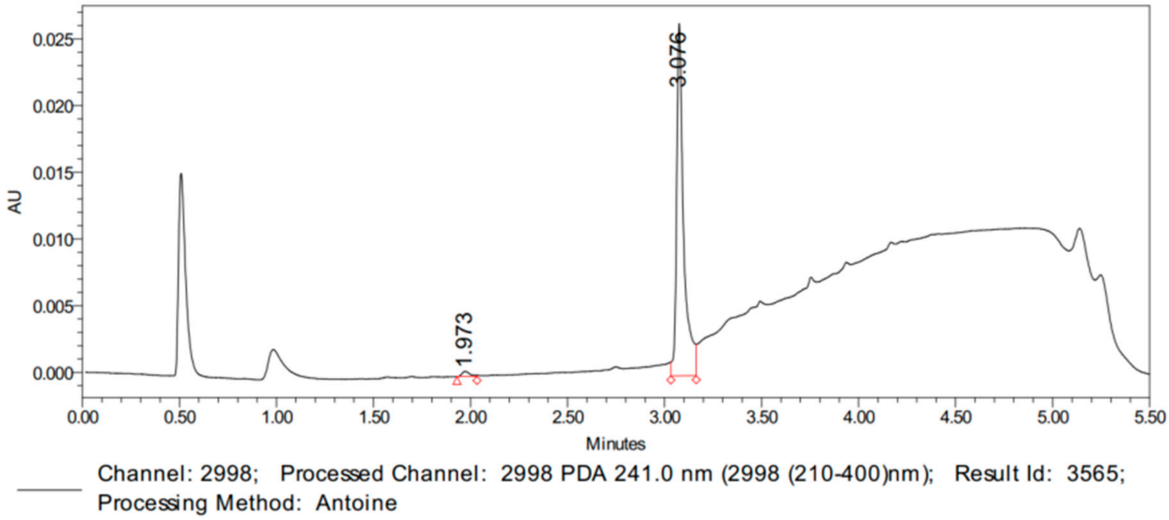

Processed Channel Descr.: 2998 PDA 241.0 nm (2998 (210-400)nm)

	Processed Channel Descr.	RT	Area	Height	% Height
1	2998 PDA 241.0 nm (2998 (210-400)nm)	1.973	1118	386	1.44
2	2998 PDA 241.0 nm (2998 (210-400)nm)	3.076	66396	26474	98.56

Figure S55 : LC chromatogram of compound **(12b)**

Compound 13b

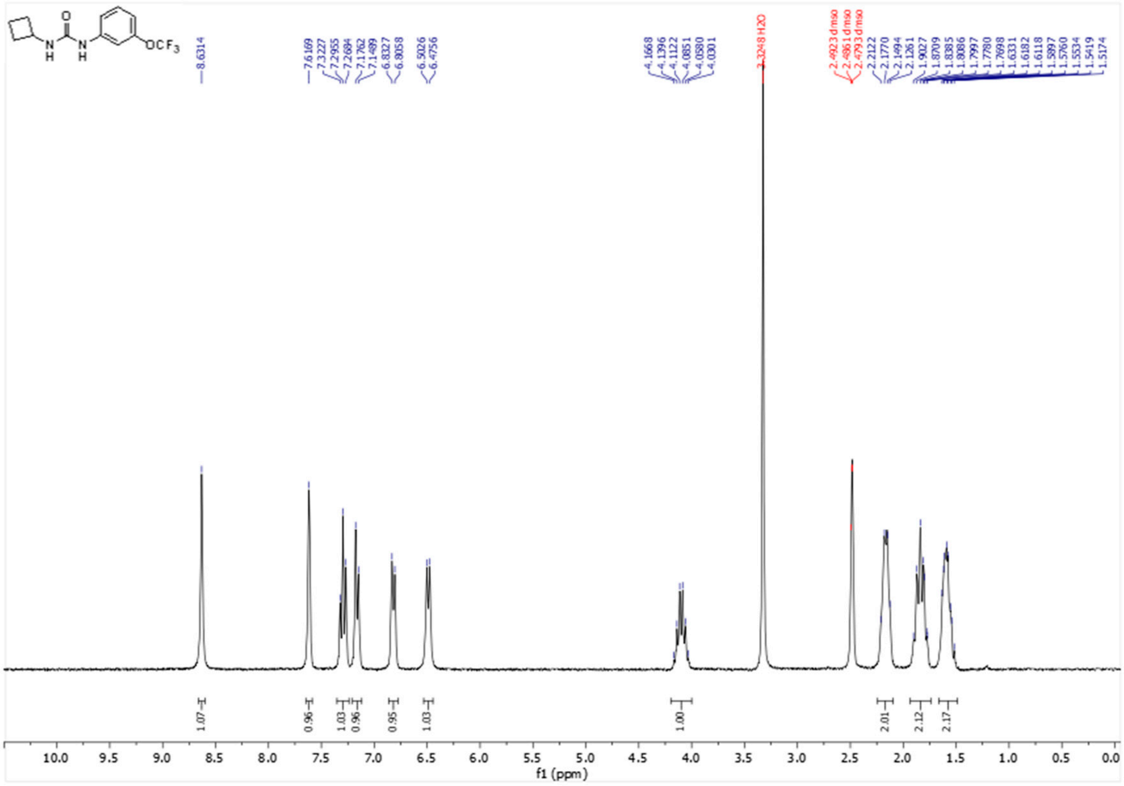

Figure S56 : ^{1}H NMR Spectrum of compound **(13b)**

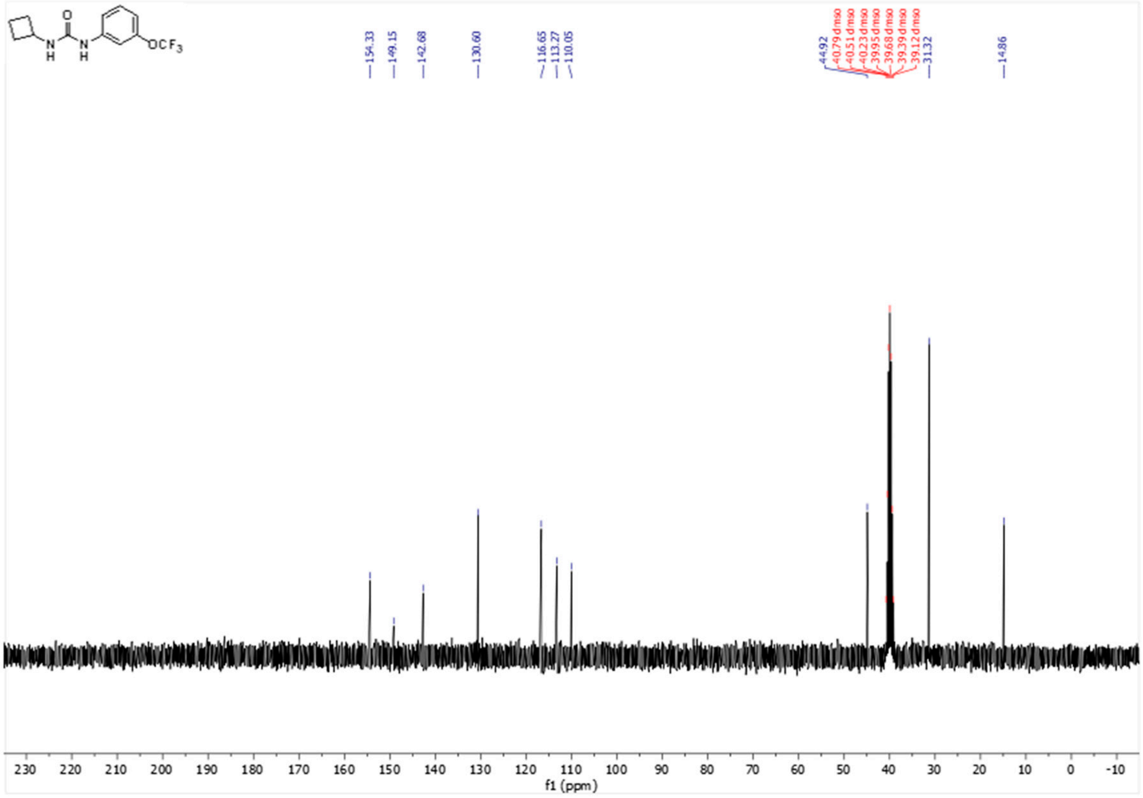

Figure S57 : ^{13}C NMR Spectrum of compound **(13b)**

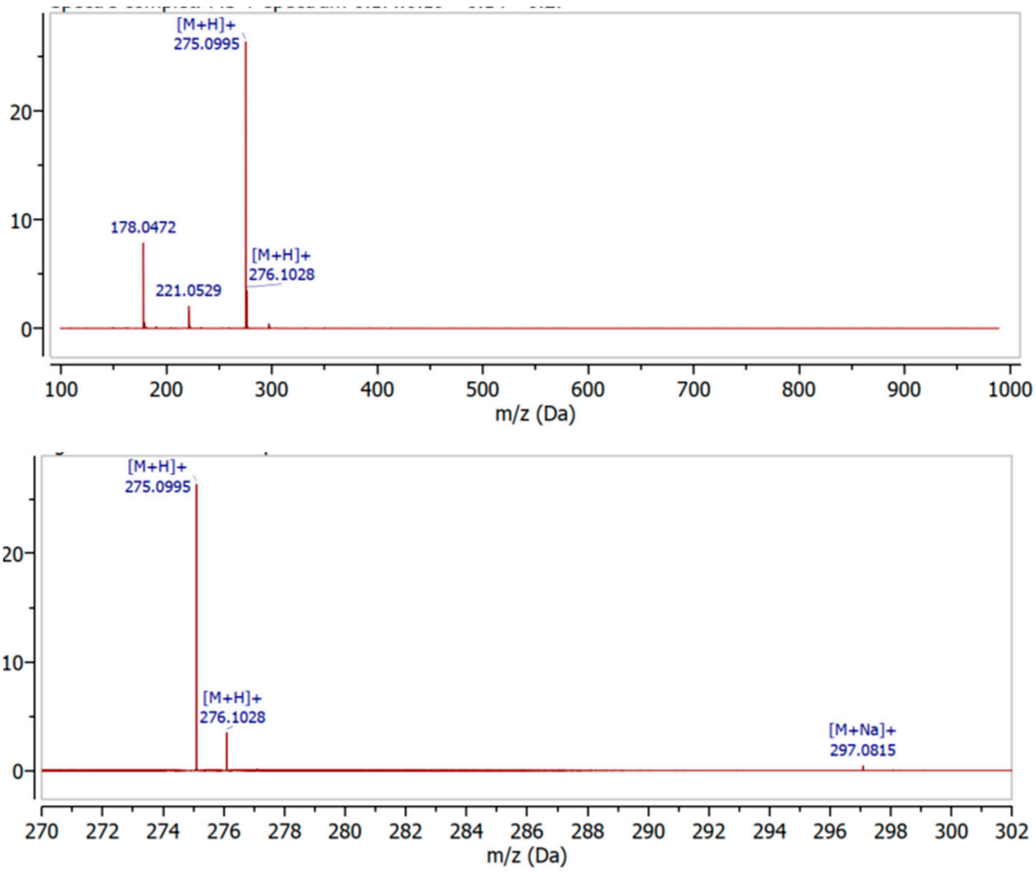

Composé trouvé: C12H13F3N2O2

Masse mesurée	Masse attendue	Intensité	Erreur (ppm)	Erreur (Da)	Ion identifié	Formule confirmée
275.0995	275.1002	249447615	-2.3	-0.0006	[M+H]+	C12H13F3N2O2
276.1028	276.1032	33002834	-1.5	-0.0004	[M+H]+	C12H13F3N2O2
297.0815	297.0821	4177071	-2.2	-0.0007	[M+Na]+	C12H13F3N2O2

<u>Figure S58 :</u> MS spectrum of compound **(13b)**

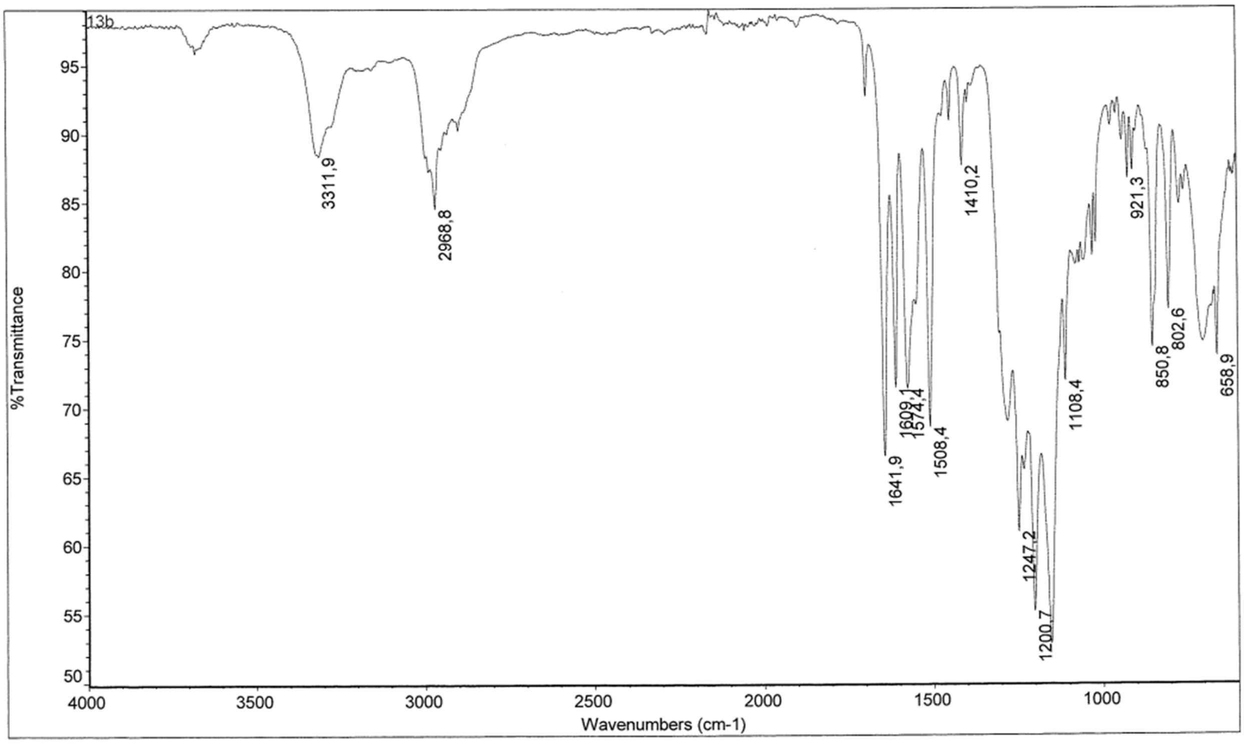

Figure S59 : IR spectrum of compound **(13b)**

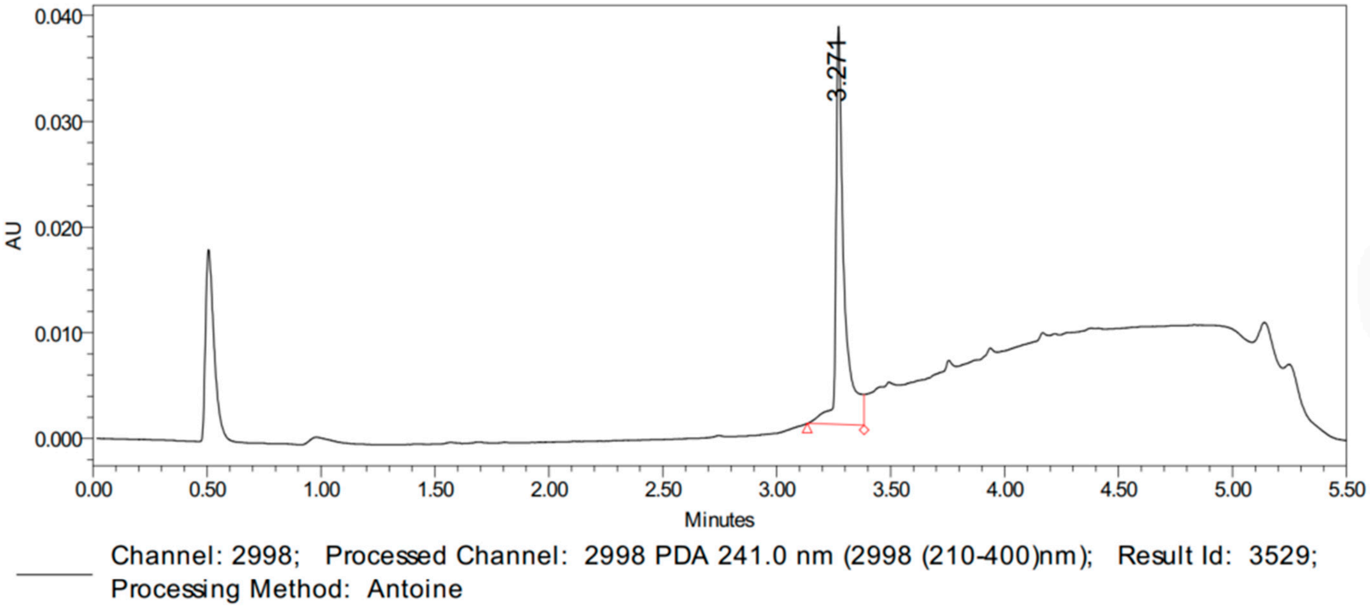

Processed Channel Descr.: 2998 PDA 241.0 nm (2998 (210-400)nm)

	Processed Channel Descr.	RT	Area	Height	% Height
1	2998 PDA 241.0 nm (2998 (210-400)nm)	3.271	95005	37660	100.00

Figure S60 : LC chromatogram of compound **(13b)**

Compound 14b

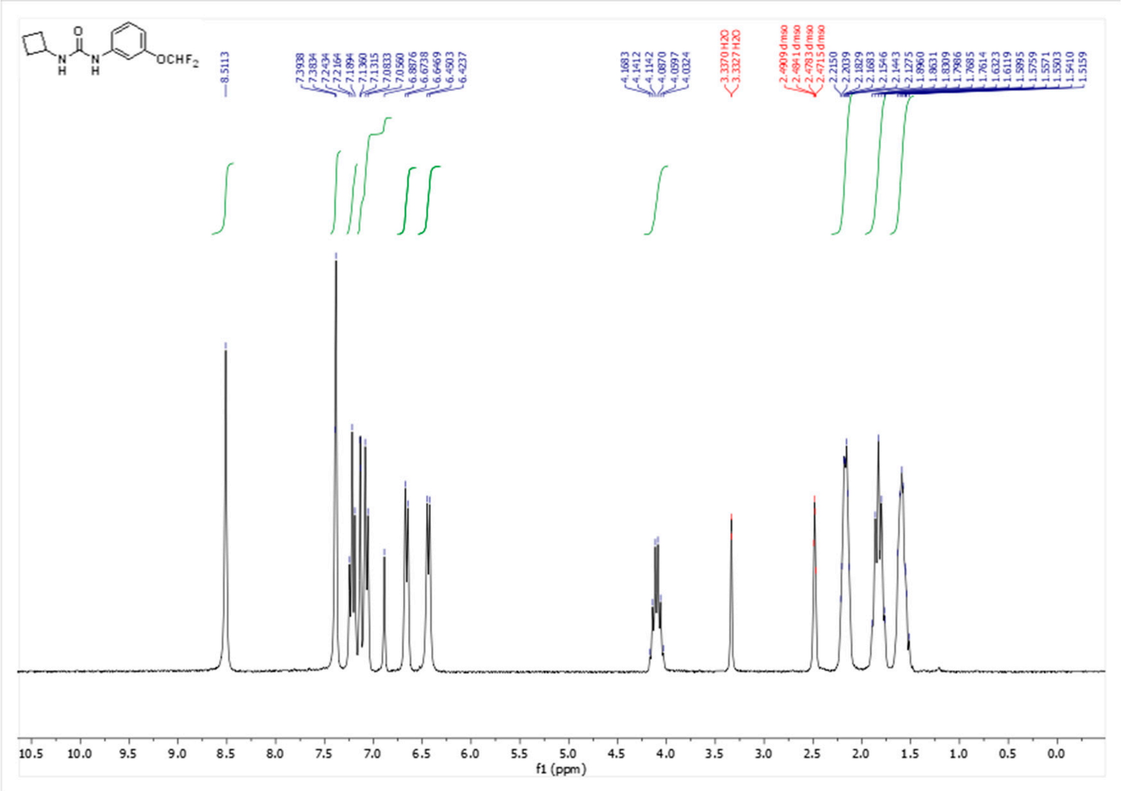

Figure S61 : ^{1}H NMR Spectrum of compound **(14b)**

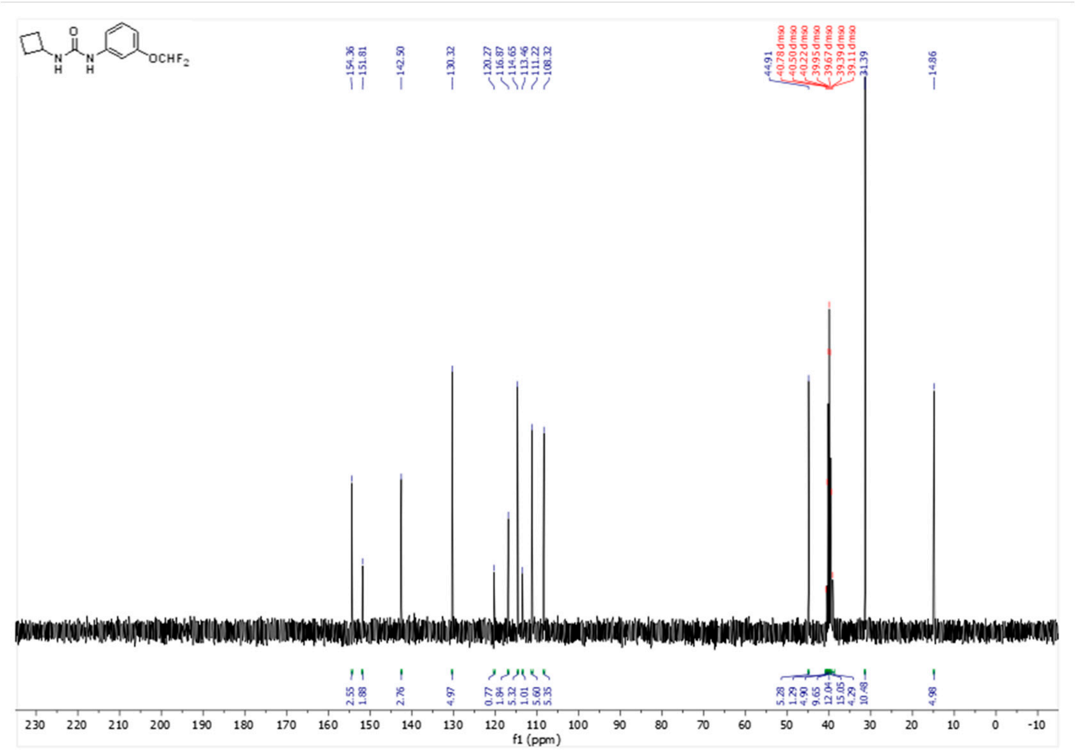

Figure S62 : ^{13}C NMR Spectrum of compound **(14b)**

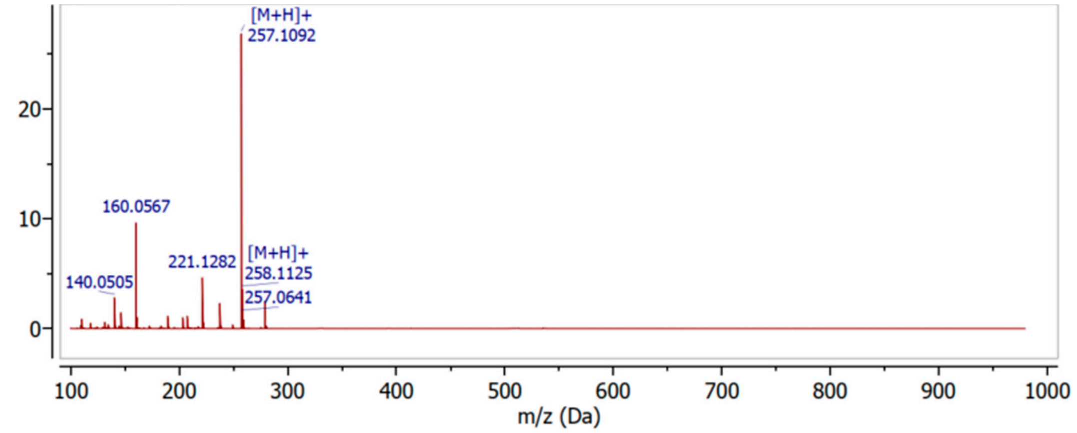

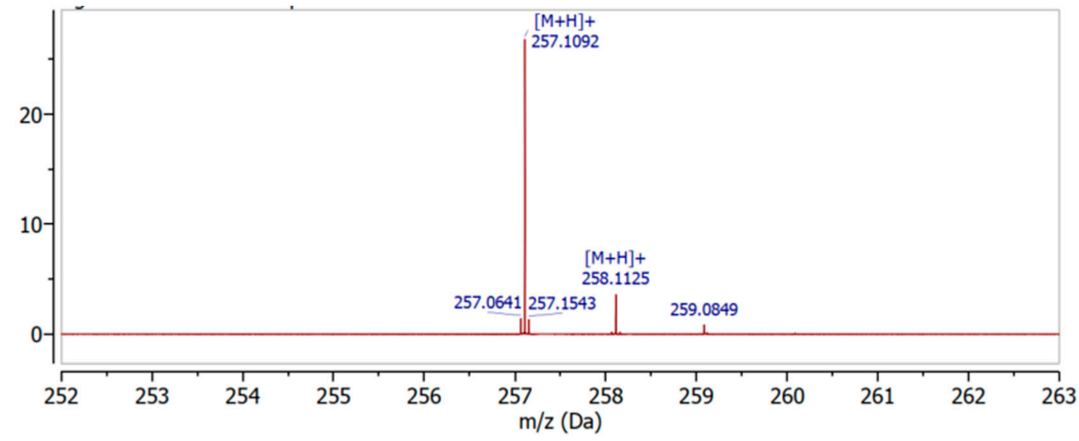

Composé trouvé: C12H14F2N2O2

Masse mesurée	Masse attendue	Intensité	Erreur (ppm)	Erreur (Da)	Ion identifié	Formule confirmée
257.1092	257.1096	304707738	-1.7	-0.0004	[M+H]+	C12H14F2N2O2
258.1125	258.1127	40191383	-0.7	-0.0002	[M+H]+	C12H14F2N2O2
279.0911	279.0916	28635430	-1.6	-0.0004	[M+Na]+	C12H14F2N2O2

<u>Figure S63 :</u> MS spectrum of compound **(14b)**

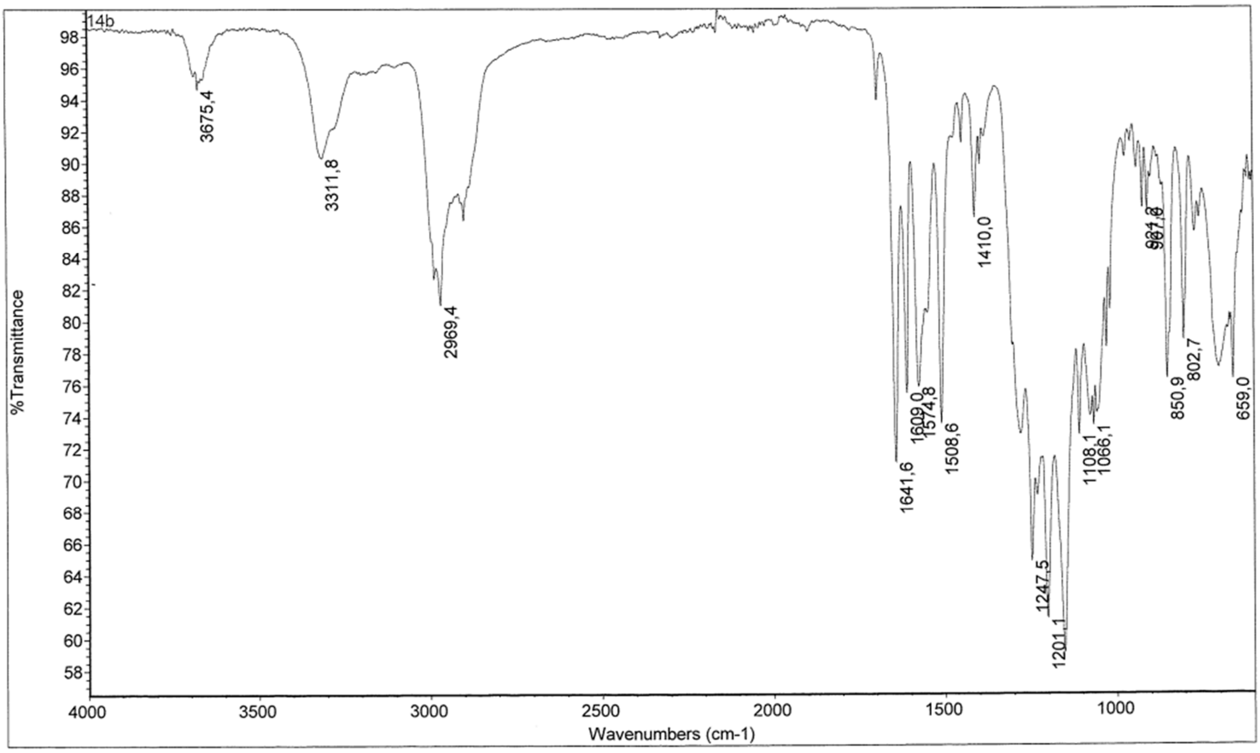

<u>Figure S64 :</u> IR spectrum of compound **(14b)**

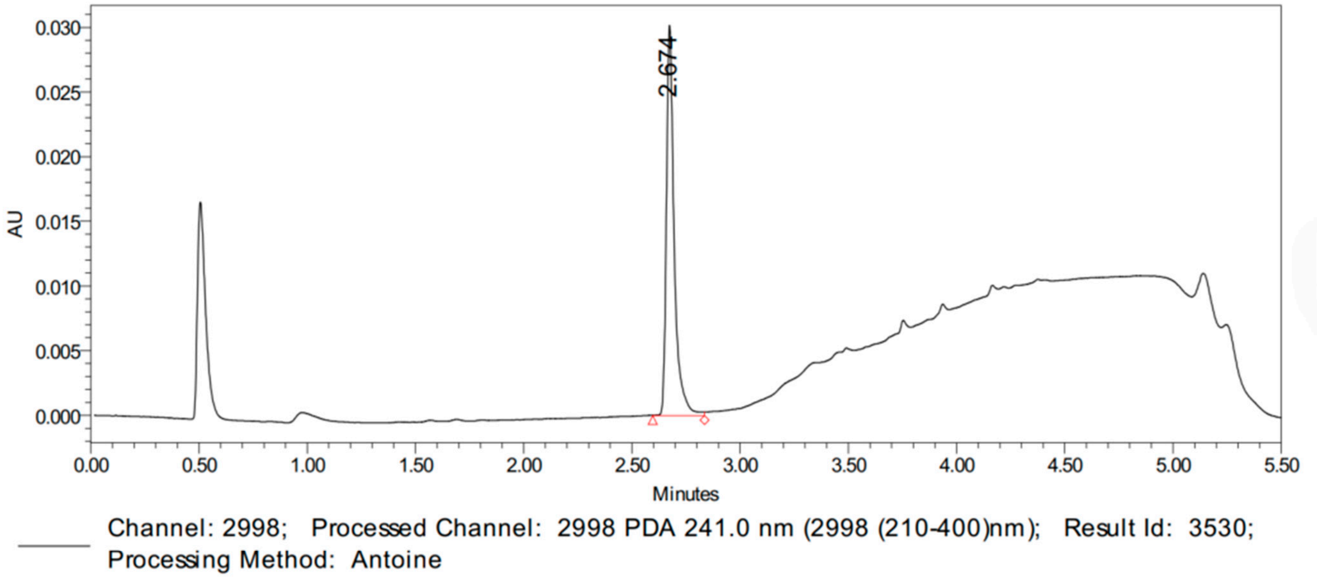

Processed Channel Descr.: 2998 PDA 241.0 nm (2998 (210-400)nm)

	Processed Channel Descr.	RT	Area	Height	% Height
1	2998 PDA 241.0 nm (2998 (210-400)nm)	2.674	76016	30177	100.00

<u>Figure S65 :</u> LC chromatogram of compound **(14b)**

Compound 15b

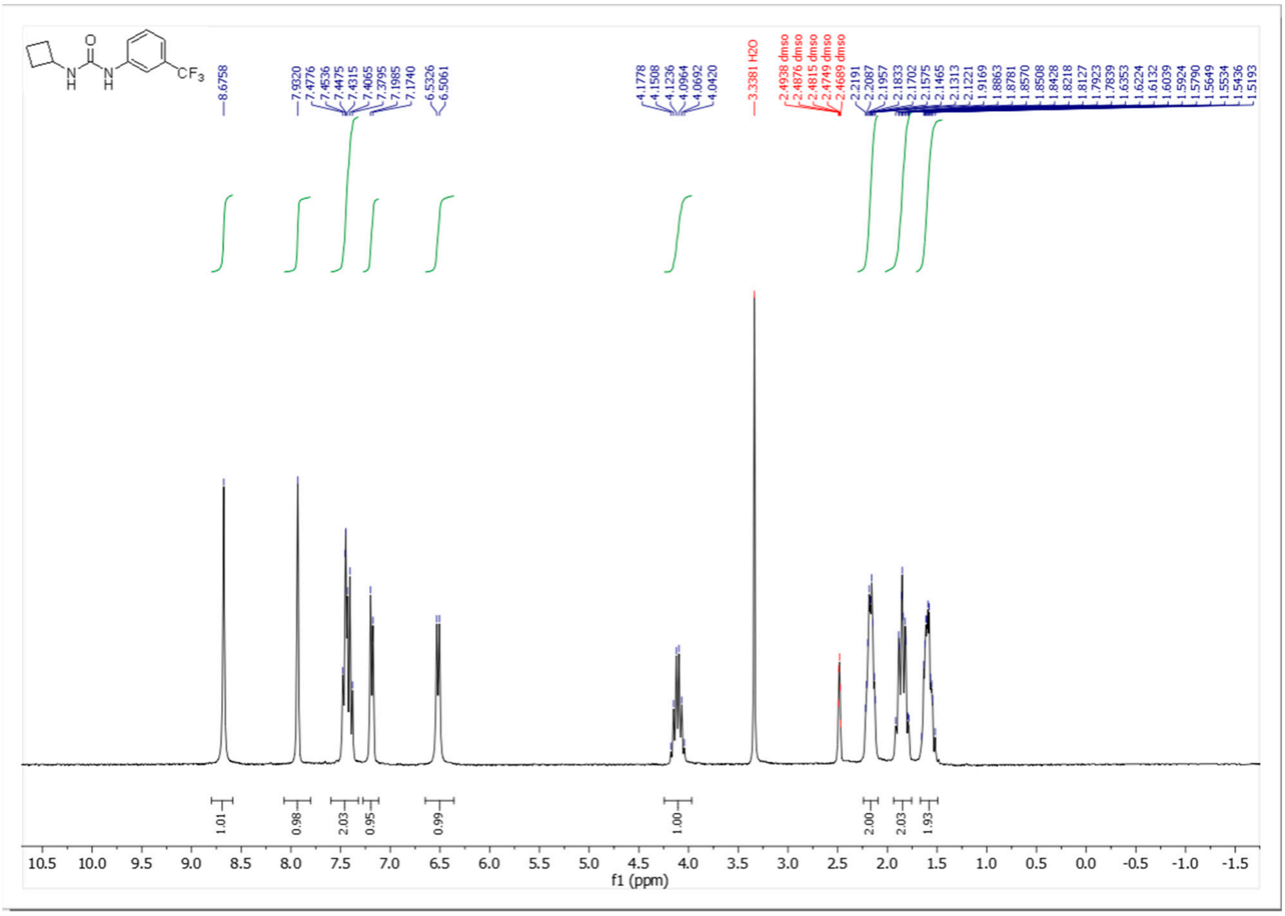

Figure S66 : ^{1}H NMR Spectrum of compound **(15b)**

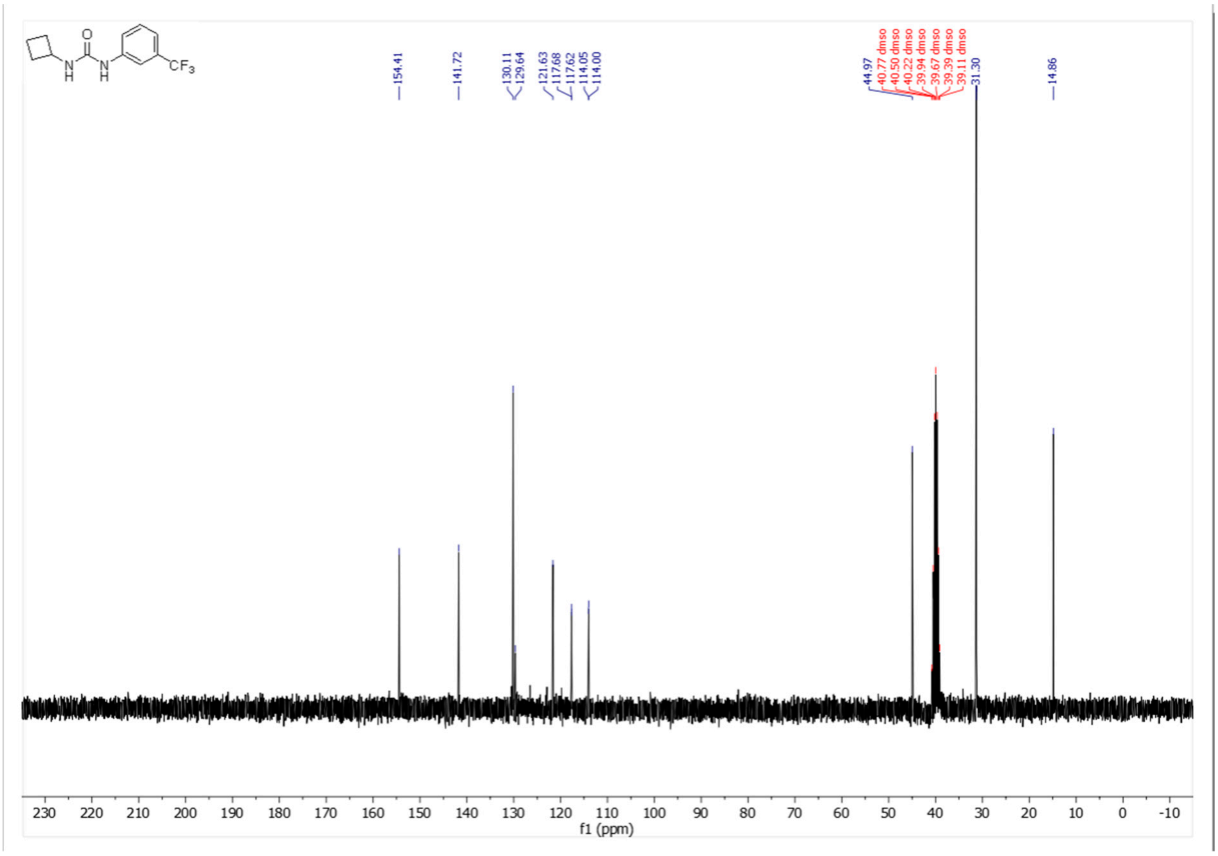

Figure S67 : ^{13}C NMR Spectrum of compound **(15b)**

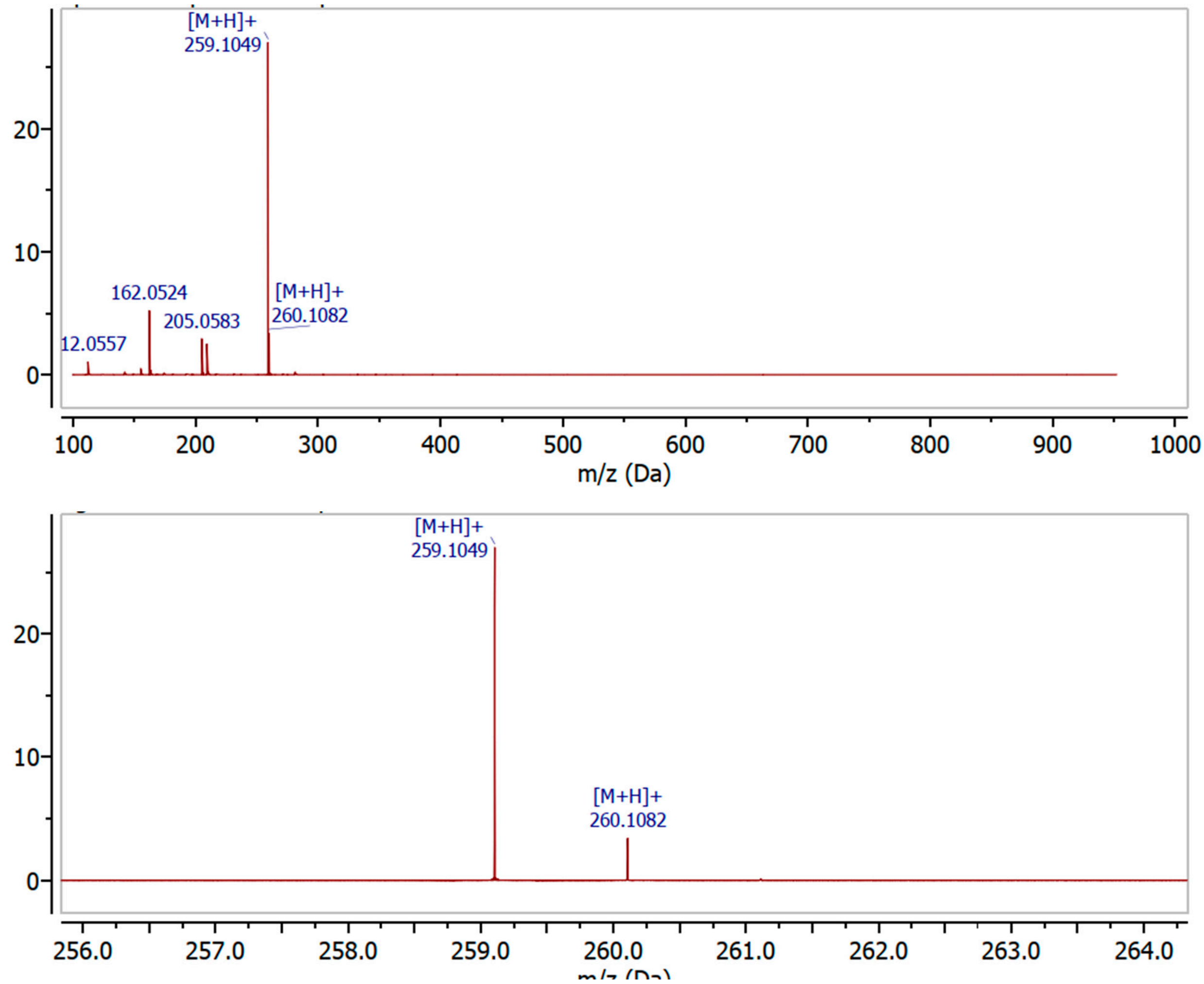

Composé trouvé: C12H13F3N2O

Masse mesurée	Masse attendue	Intensité	Erreur (ppm)	Erreur (Da)	Ion identifié	Formule confirmée
259.1049	259.1053	77132364	-1.5	-0.0004	[M+H]+	C12H13F3N2O
260.1082	260.1083	9970949	-0.4	-0.0001	[M+H]+	C12H13F3N2O

<u>Figure S68 :</u> MS spectrum of compound **(15b)**

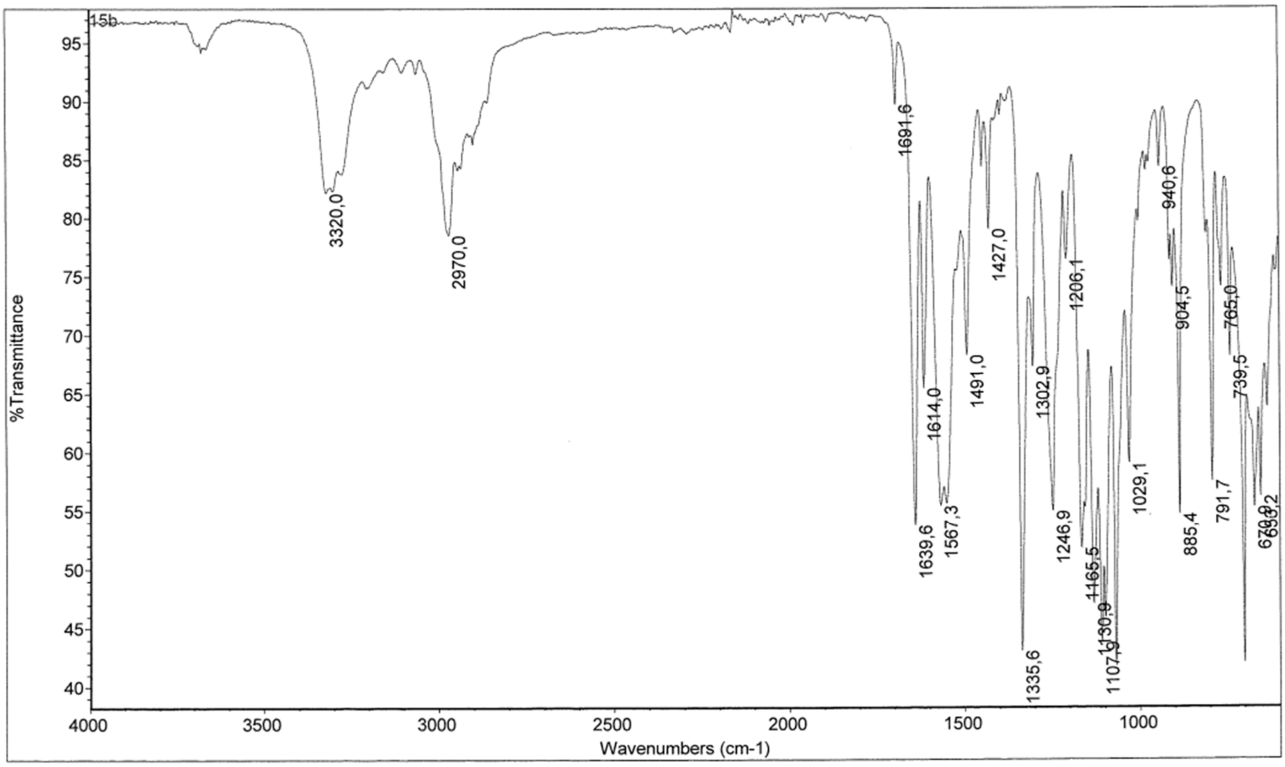

Figure S69 : IR spectrum of compound **(15b)**

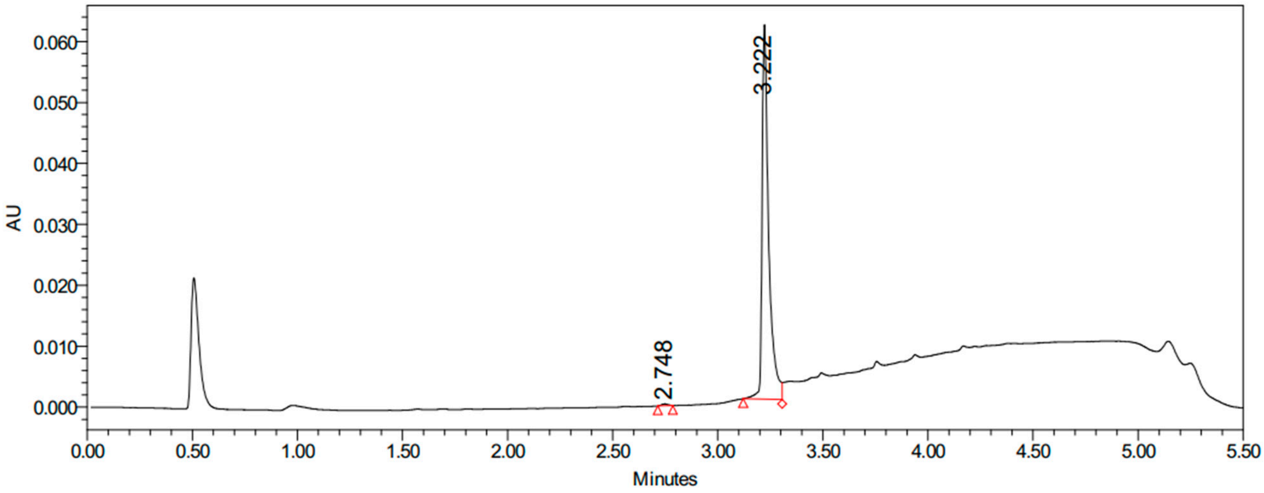

Channel: 2998; Processed Channel: 2998 PDA 241.0 nm (2998 (210-400)nm); Result Id: 3546; Processing Method: Antoine

Processed Channel Descr.: 2998 PDA 241.0 nm (2998 (210-400)nm)

	Processed Channel Descr.	RT	Area	Height	% Height
1	2998 PDA 241.0 nm (2998 (210-400)nm)	2.748	593	278	0.45
2	2998 PDA 241.0 nm (2998 (210-400)nm)	3.222	131585	61453	99.55

Figure S70 : LC chromatogram of compound **(15b)**

Compound 16b

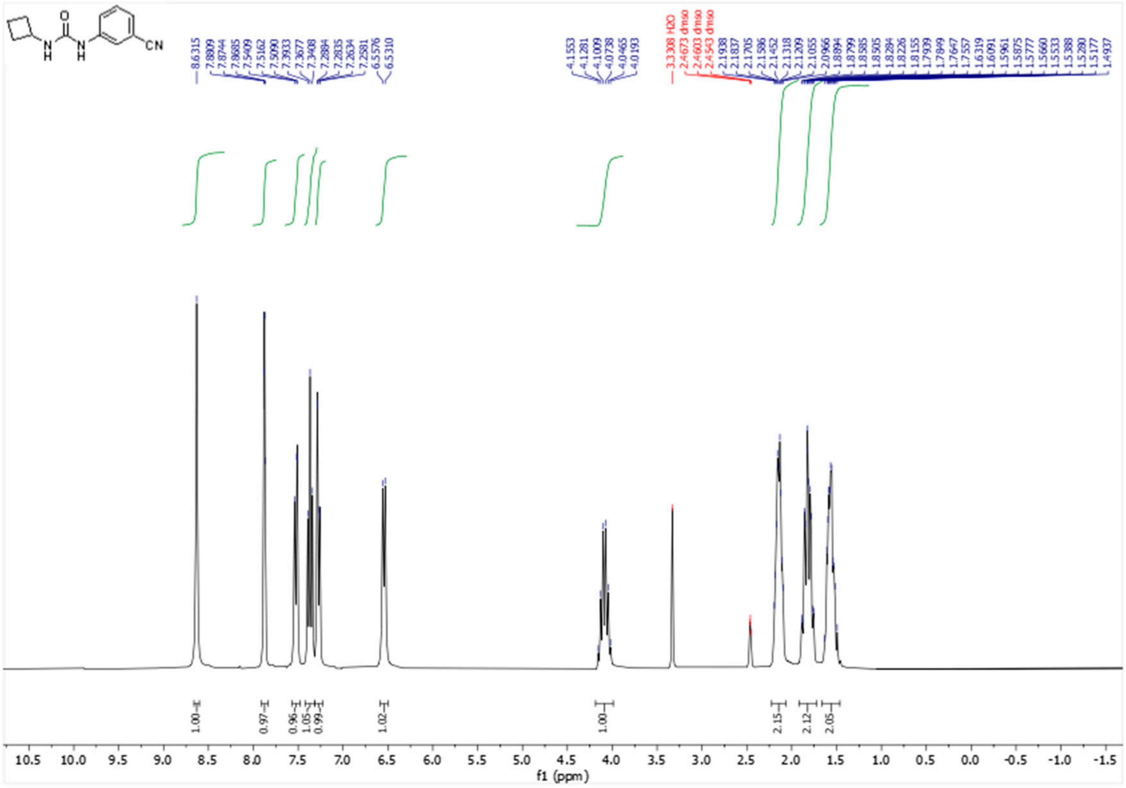

Figure S71: ^{1}H NMR Spectrum of compound **(16b)**

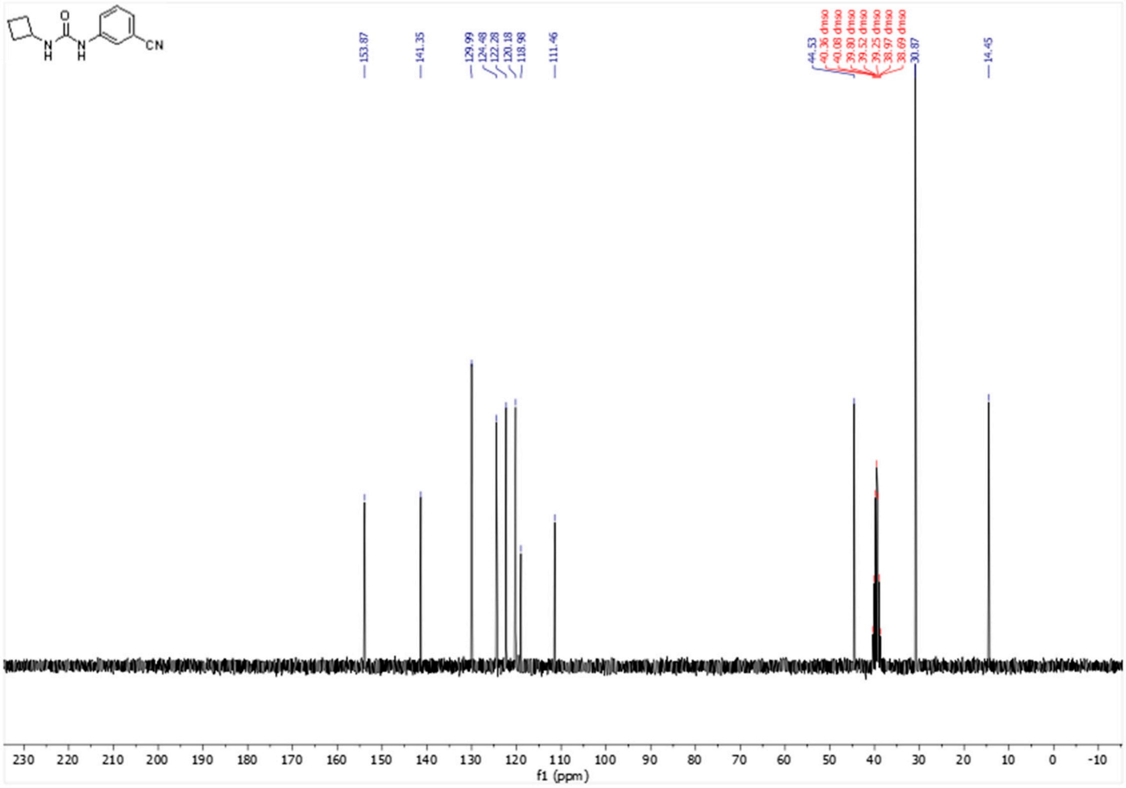

Figure S72 : ^{13}C NMR Spectrum of compound **(16b)**

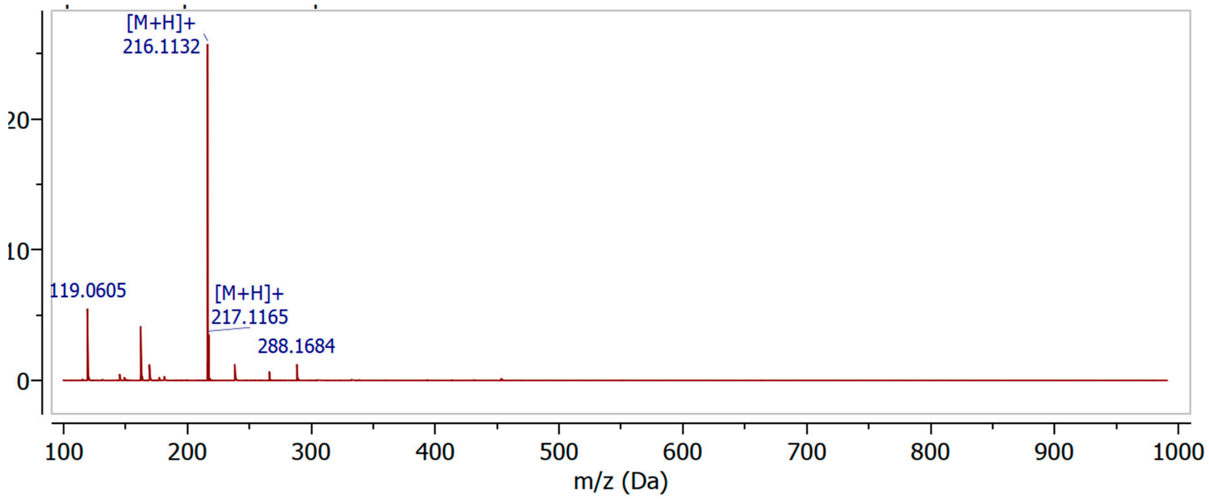

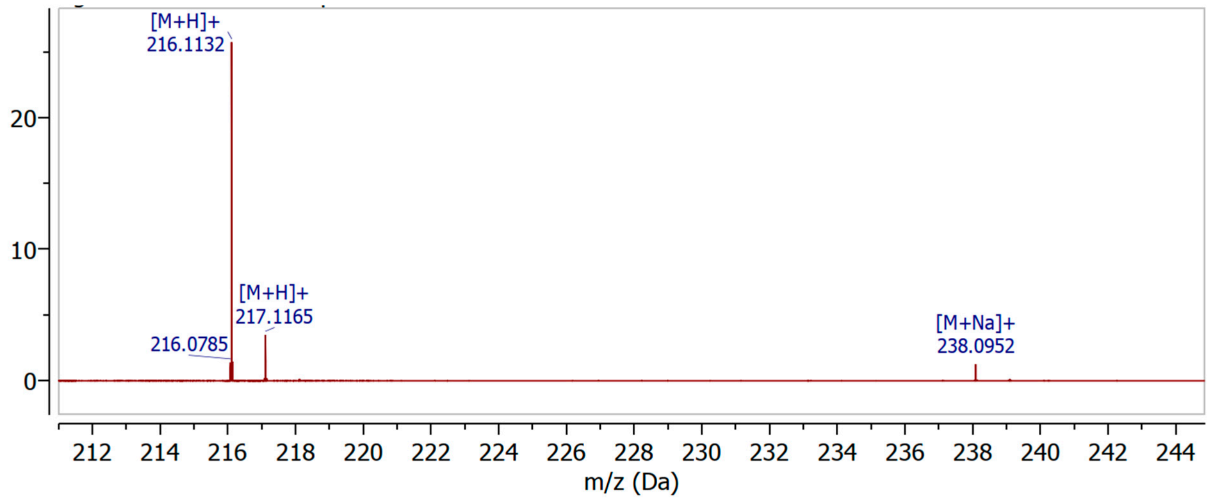

Composé trouvé: C12H13N3O

Masse mesurée	Masse attendue	Intensité	Erreur (ppm)	Erreur (Da)	Ion identifié	Formule confirmée
216.1132	216.1131	43459540	0.1	0.0000	[M+H]+	C12H13N3O
217.1165	217.1160	5651604	2.0	0.0004	[M+H]+	C12H13N3O
238.0952	238.0951	2084190	0.3	0.0001	[M+Na]+	C12H13N3O

Figure S73 : MS spectrum of compound **(16b)**

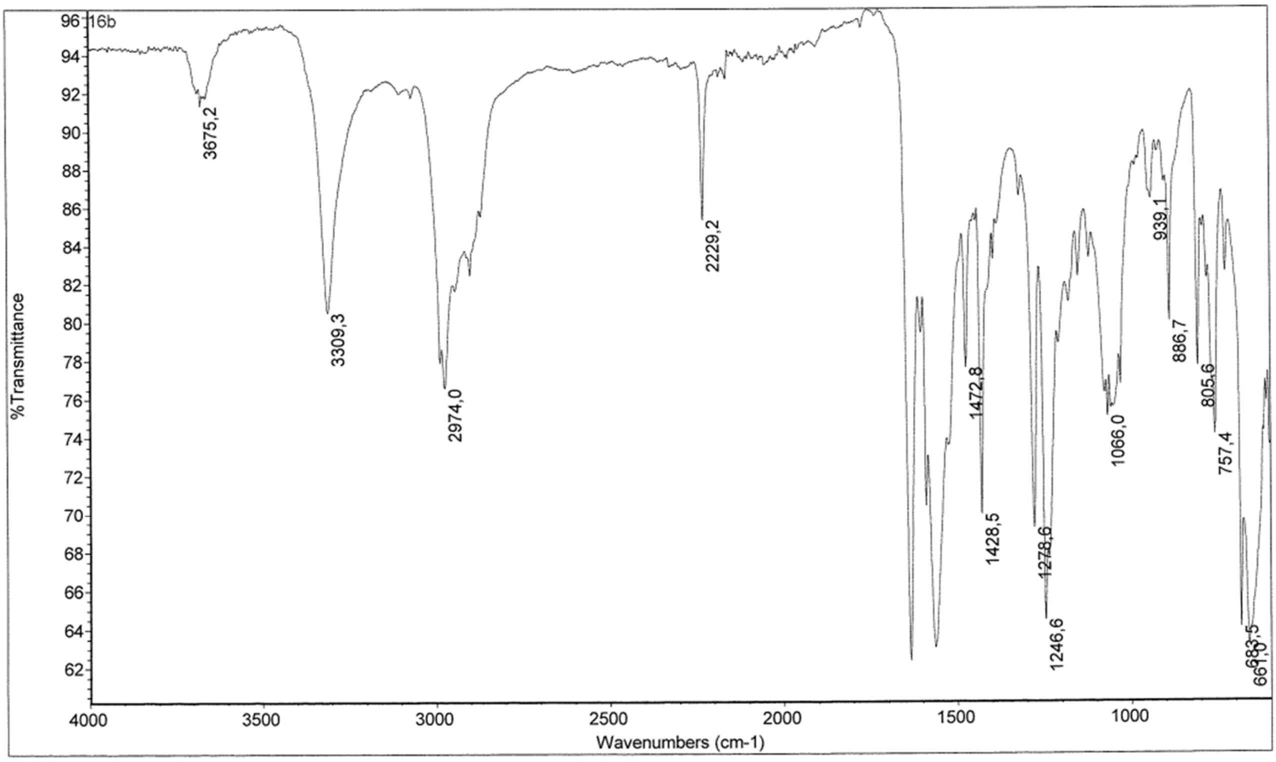

<u>Figure S74 :</u> IR spectrum of compound **(16b)**

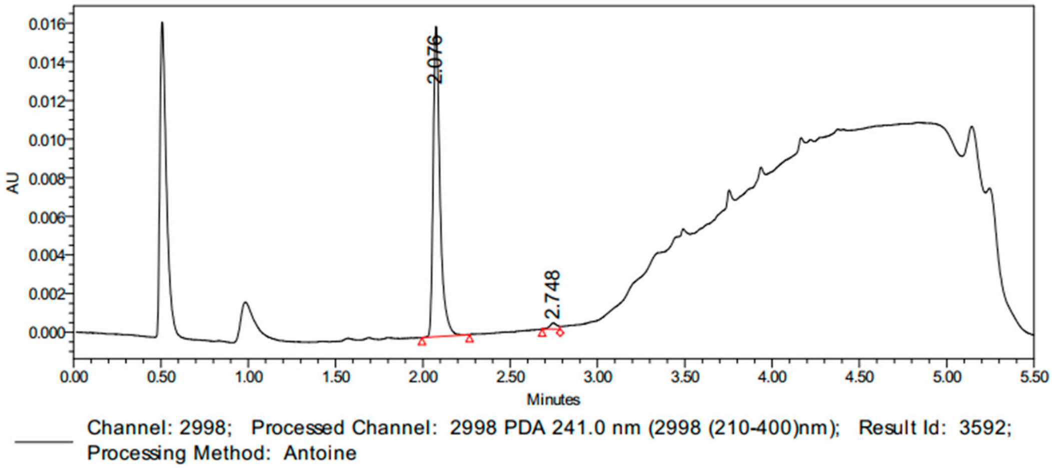

Processed Channel Descr.: 2998 PDA 241.0 nm (2998 (210-400)nm)

	Processed Channel Descr.	RT	Area	Height	% Height
1	2998 PDA 241.0 nm (2998 (210-400)nm)	2.076	42973	16064	98.13
2	2998 PDA 241.0 nm (2998 (210-400)nm)	2.748	919	306	1.87

<u>Figure S75 :</u> LC chromatogram of compound **(16b)**

Compound 17b

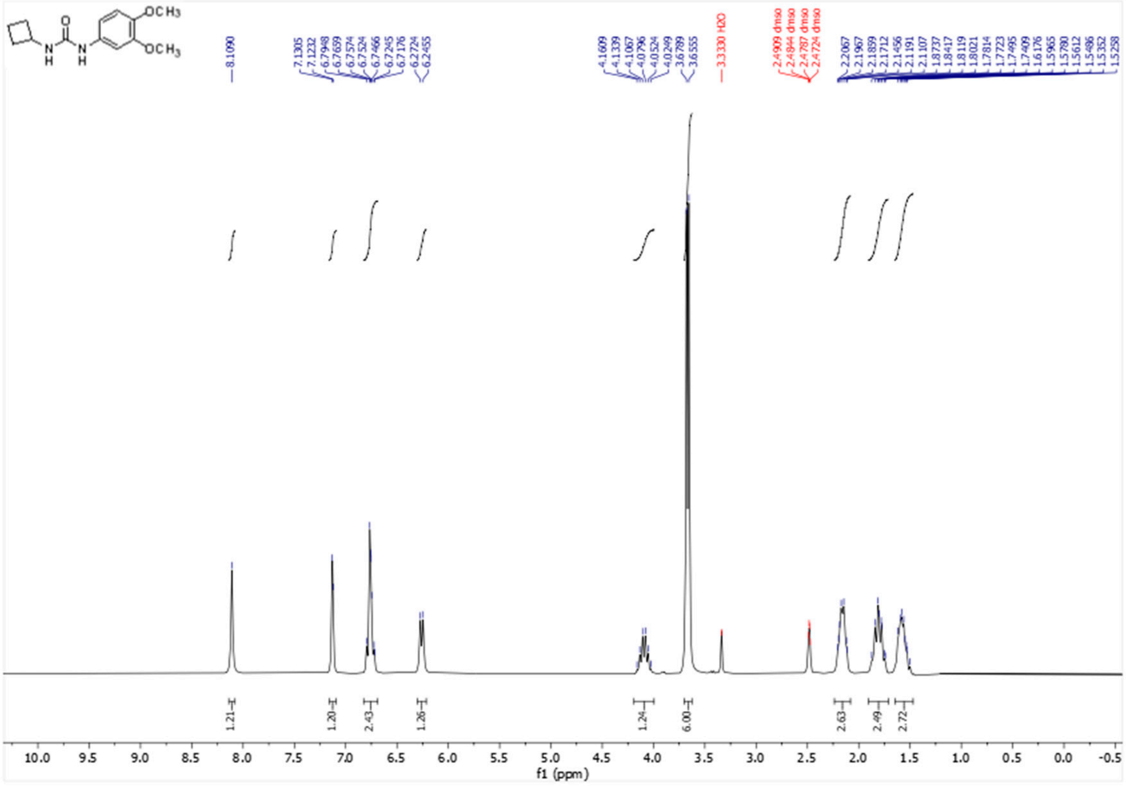

Figure S76 : ^{1}H NMR Spectrum of compound **(17b)**

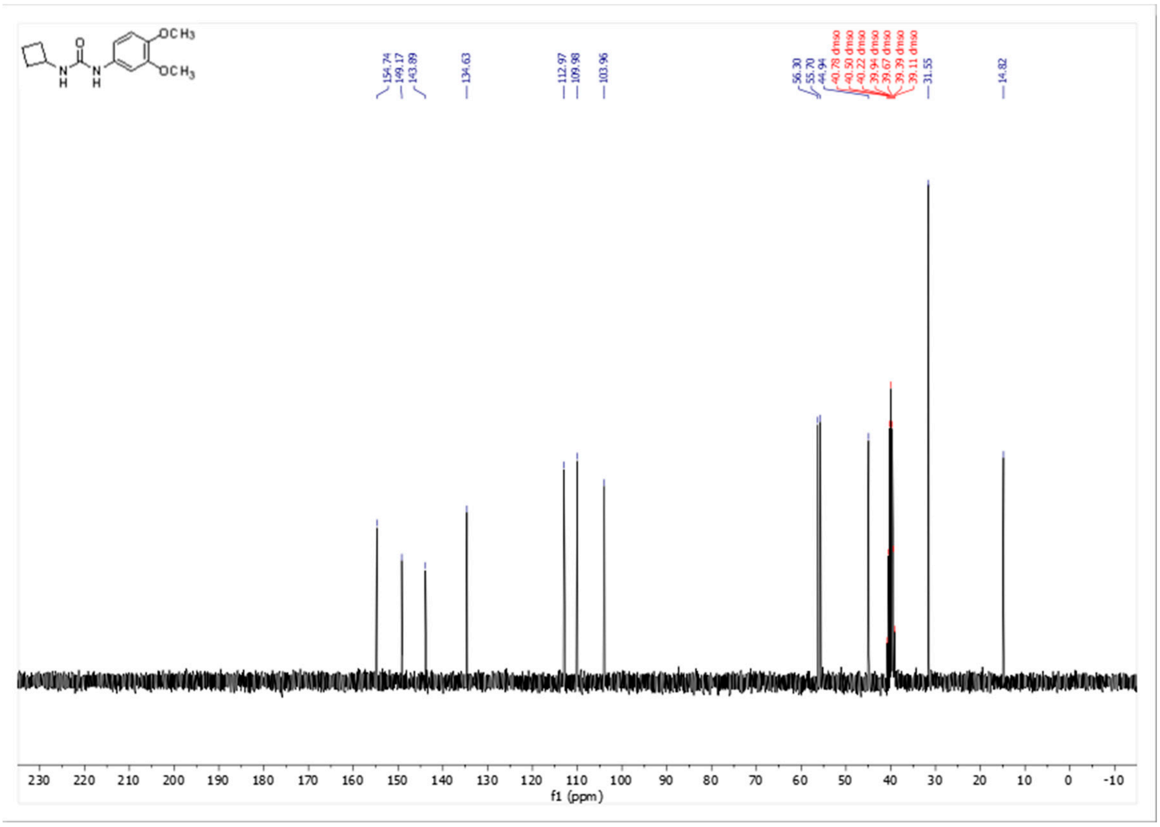

Figure S77 : ^{13}C NMR Spectrum of compound **(17b)**

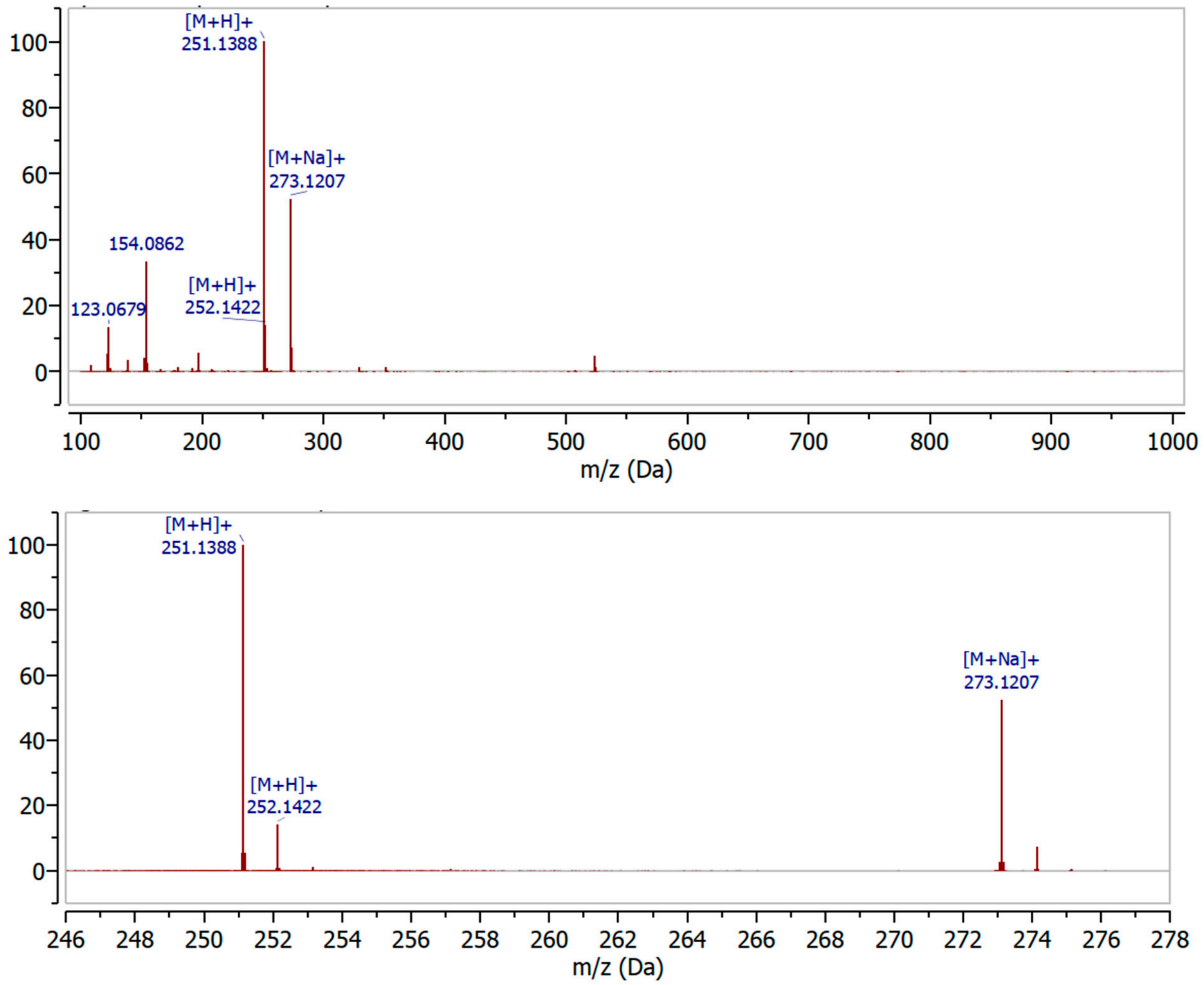

<u>Figure S78 :</u> MS spectrum of compound **(17b)**

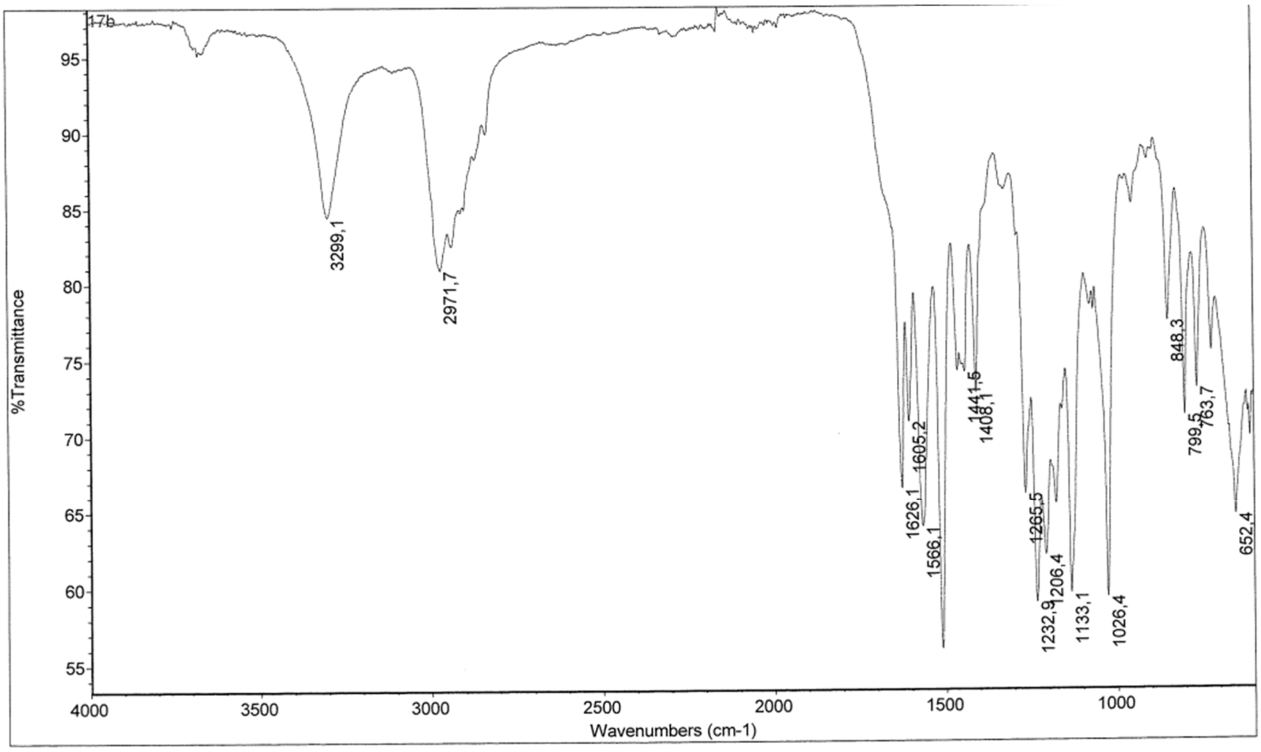

Figure S79 : IR spectrum of compound **(17b)**

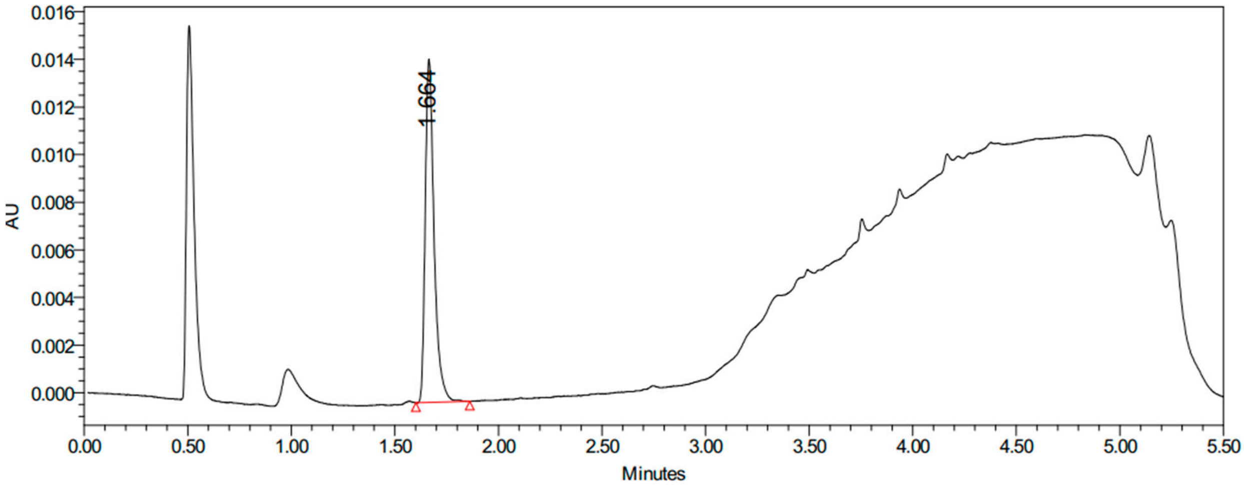

Channel: 2998; Processed Channel: 2998 PDA 241.0 nm (2998 (210-400)nm); Result Id: 3533; Processing Method: Antoine

Processed Channel Descr.: 2998 PDA 241.0 nm (2998 (210-400)nm)

	Processed Channel Descr.	RT	Area	Height	% Height
1	2998 PDA 241.0 nm (2998 (210-400)nm)	1.664	43062	14426	100.00

Figure S80 : LC chromatogram of compound **(17b)**

Compound 18b

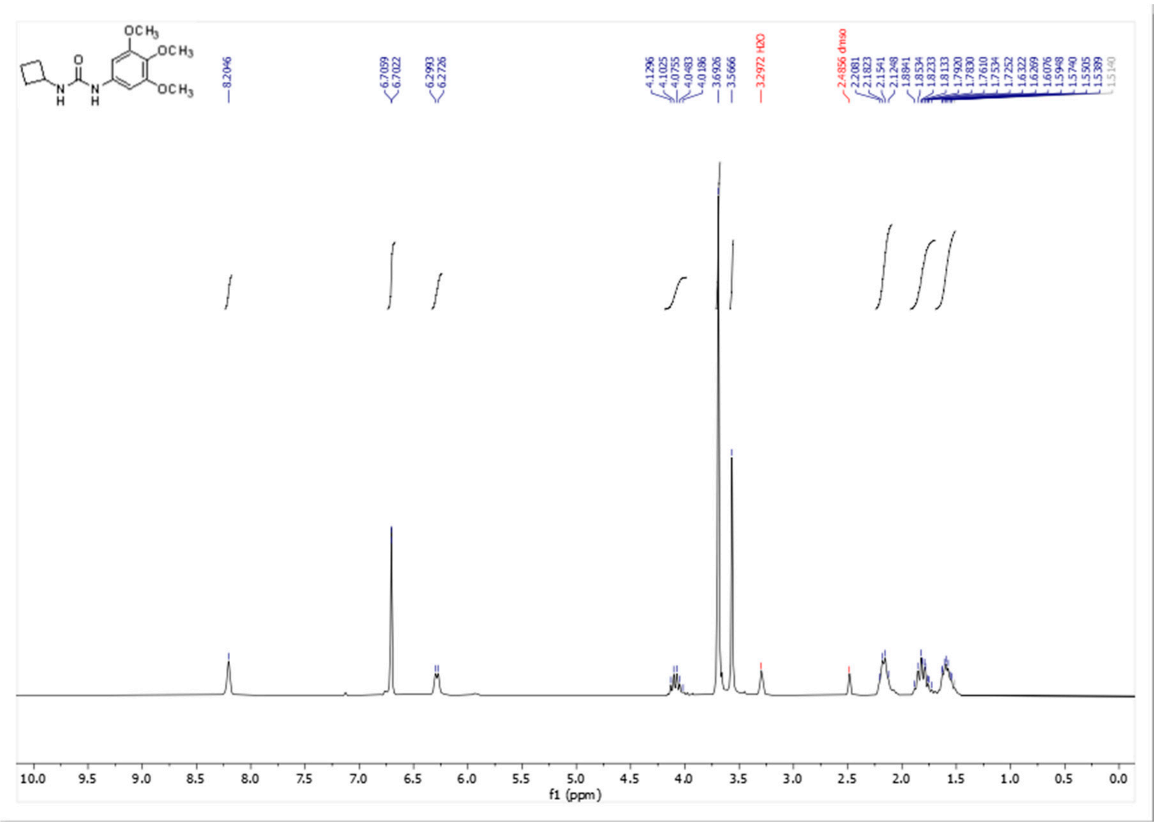

Figure S81 : ^{1}H NMR Spectrum of compound **(18b)**

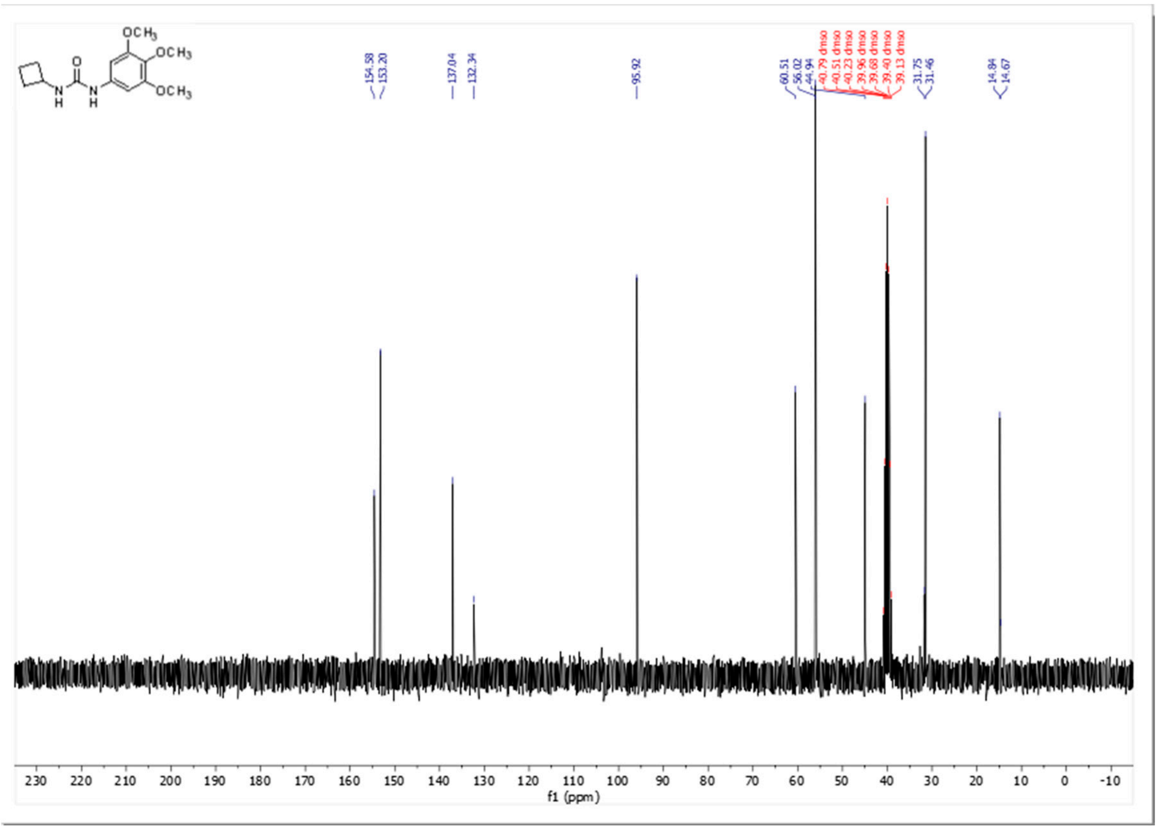

Figure S82 : ^{13}C NMR Spectrum of compound **(18b)**

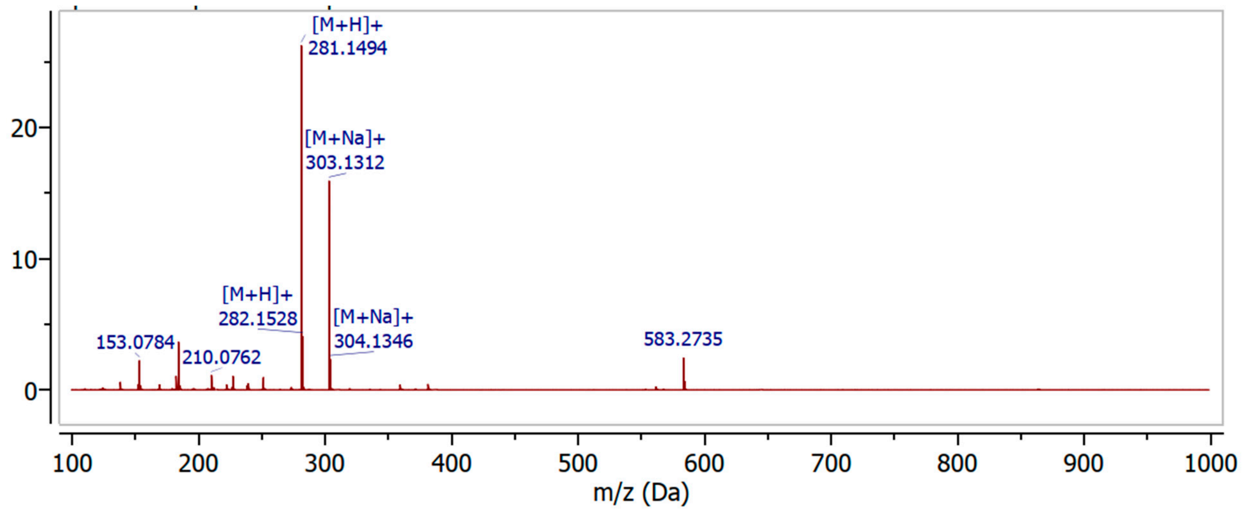

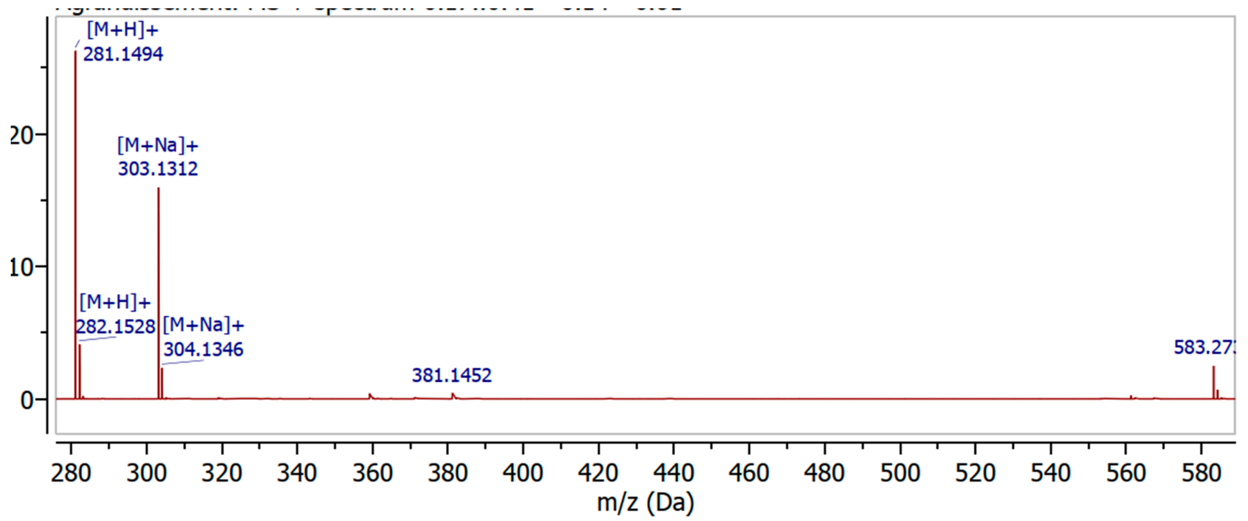

Composé trouvé: C14H20N2O4

Masse mesurée	Masse attendue	Intensité	Erreur (ppm)	Erreur (Da)	Ion identifié	Formule confirmée
281.1494	281.1496	2222412045	-0.8	-0.0002	[M+H]+	C14H20N2O4
282.1528	282.1527	340704996	0.2	0.0000	[M+H]+	C14H20N2O4
303.1312	303.1315	1333466695	-0.9	-0.0003	[M+Na]+	C14H20N2O4
304.1346	304.1346	196540767	0.0	0.0000	[M+Na]+	C14H20N2O4

<u>Figure S83 :</u> MS spectrum of compound **(18b)**

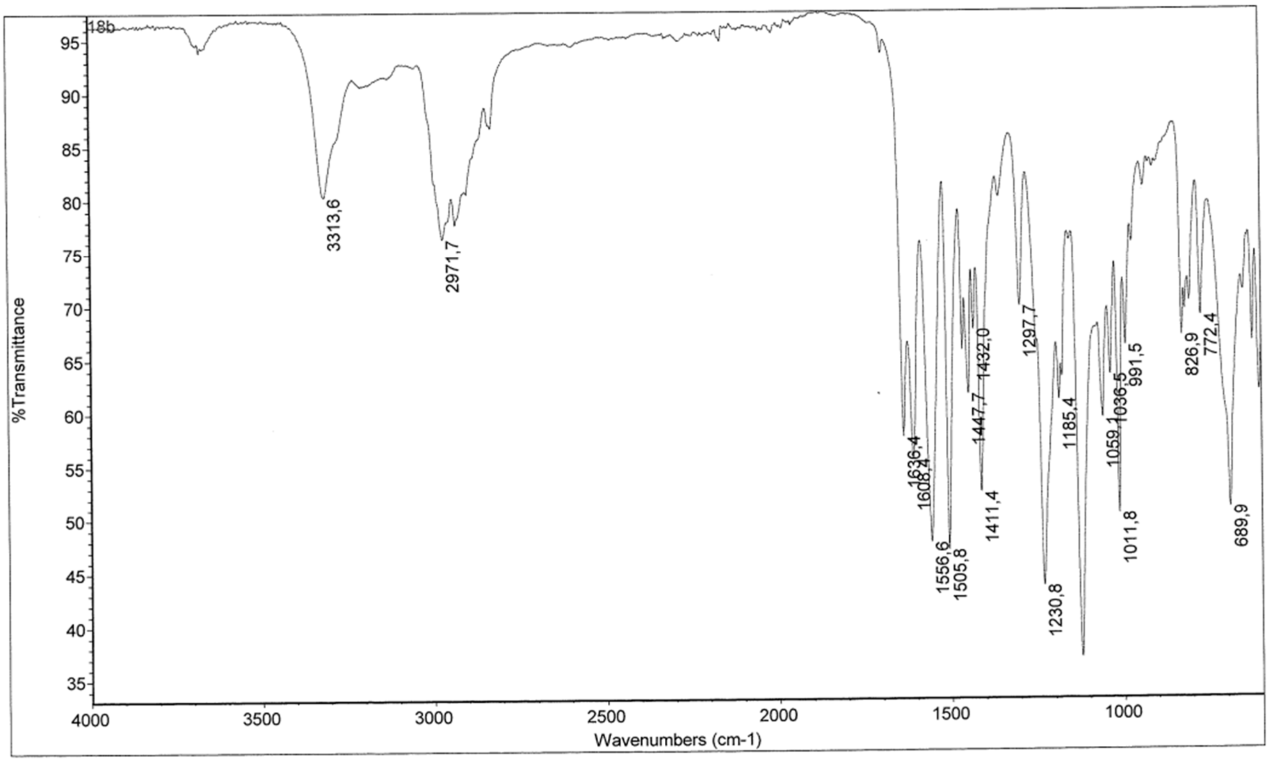

Figure S84 : IR spectrum of compound **(18b)**

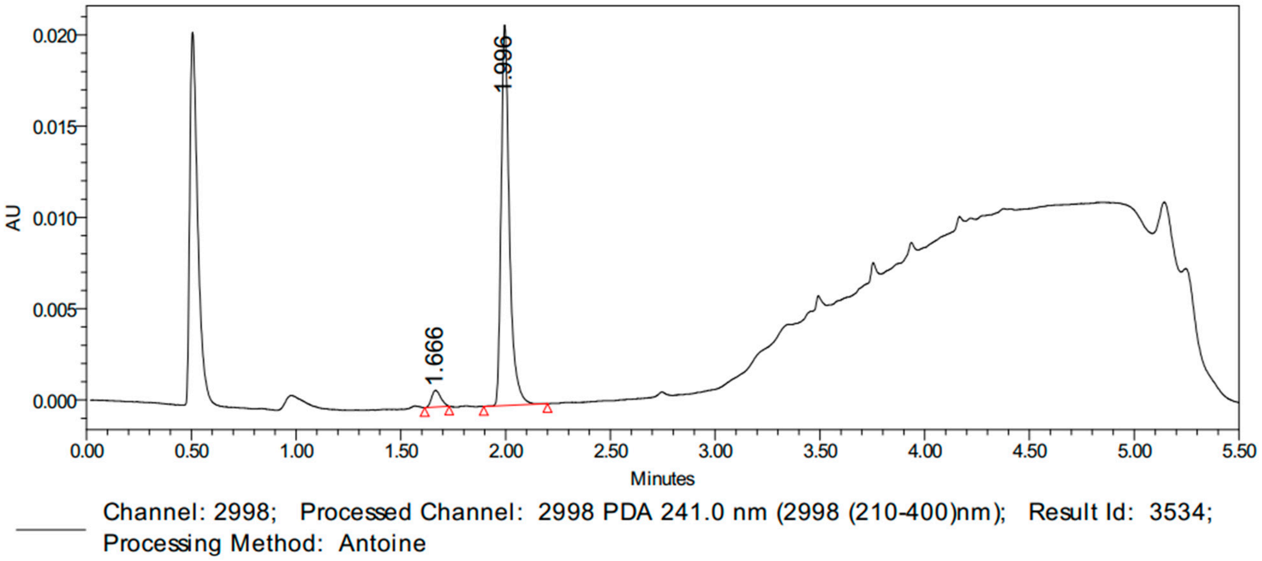

Processed Channel Descr.: 2998 PDA 241.0 nm (2998 (210-400)nm)

	Processed Channel Descr.	RT	Area	Height	% Height
1	2998 PDA 241.0 nm (2998 (210-400)nm)	1.666	2781	912	4.19
2	2998 PDA 241.0 nm (2998 (210-400)nm)	1.996	56982	20855	95.81

Figure S85 : LC chromatogram of compound **(18b)**

Compound 19b

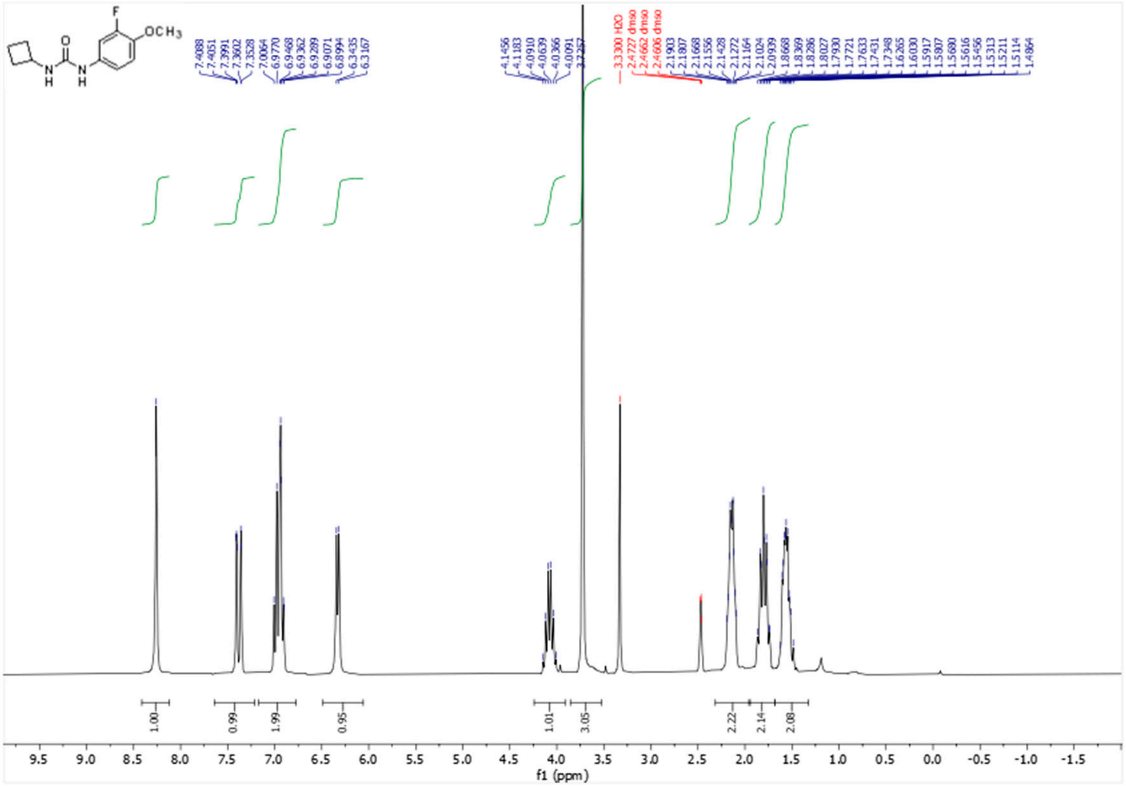

Figure S86 : ^{1}H NMR Spectrum of compound **(19b)**

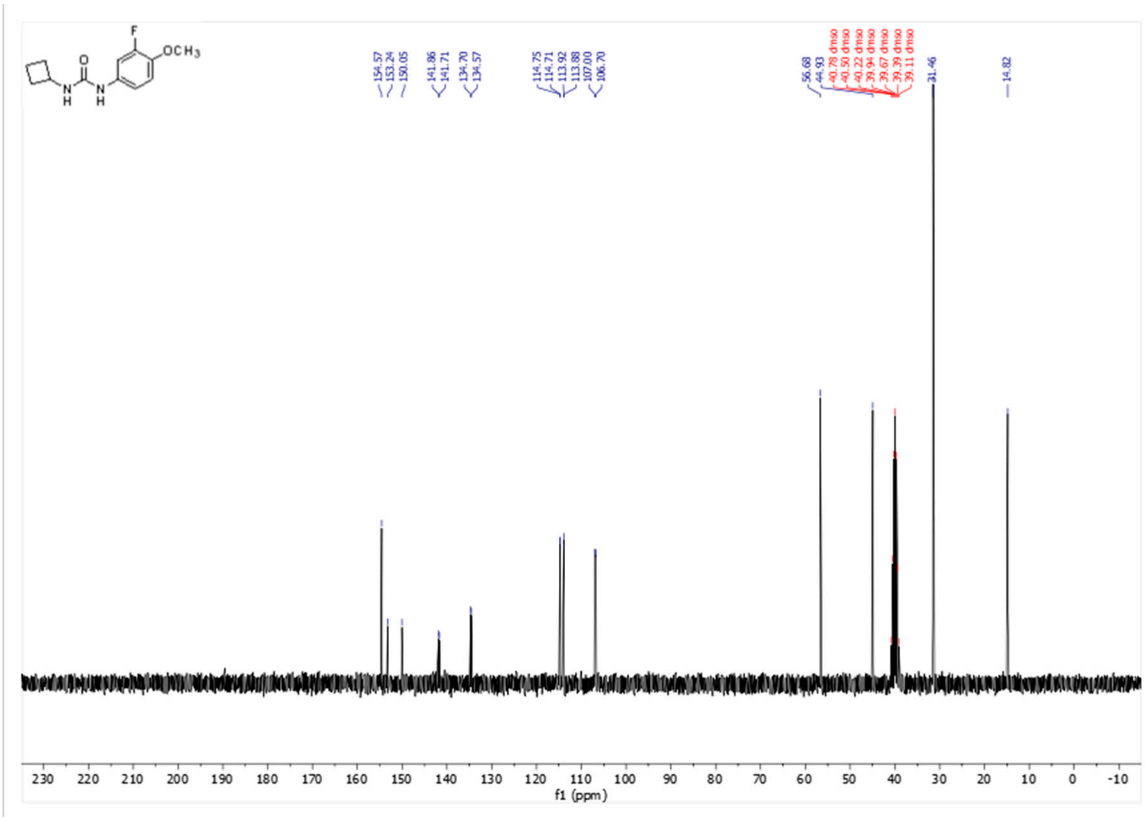

Figure S87 : ^{13}C NMR Spectrum of compound **(19b)**

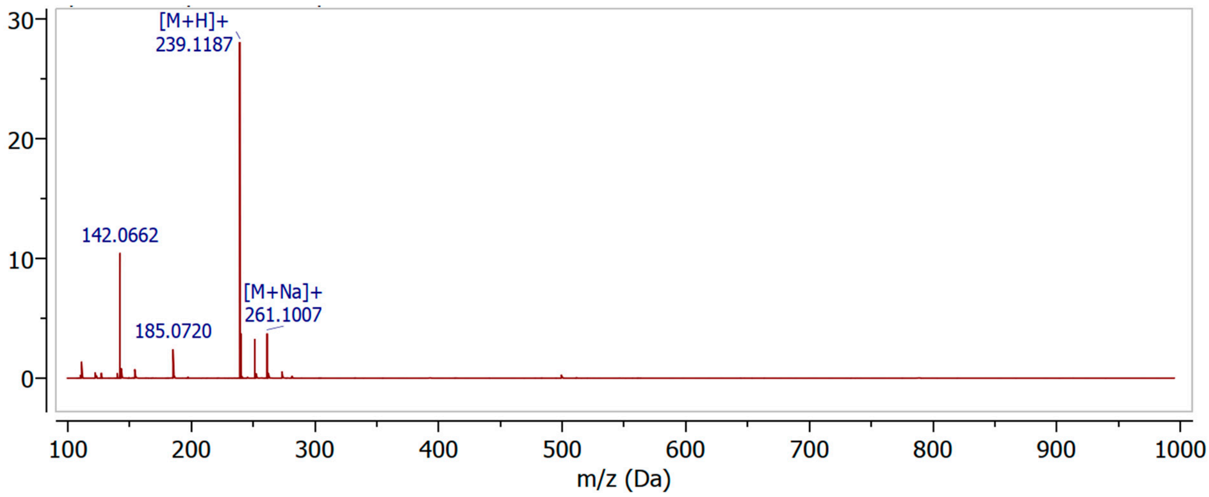

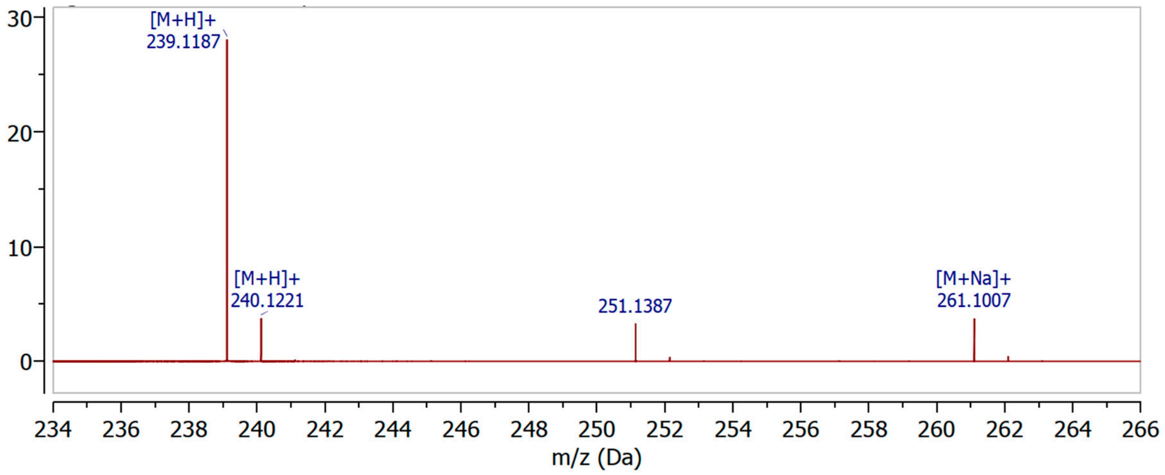

Composé trouvé: C12H15FN2O2

Masse mesurée	Masse attendue	Intensité	Erreur (ppm)	Erreur (Da)	Ion identifié	Formule confirmée
239.1187	239.1190	208850474	-1.3	-0.0003	[M+H]+	C12H15FN2O2
240.1221	240.1221	28201543	-0.1	-0.0000	[M+H]+	C12H15FN2O2
261.1007	261.1010	28303763	-1.2	-0.0003	[M+Na]+	C12H15FN2O2

<u>Figure S88 :</u> MS spectrum of compound **(19b)**

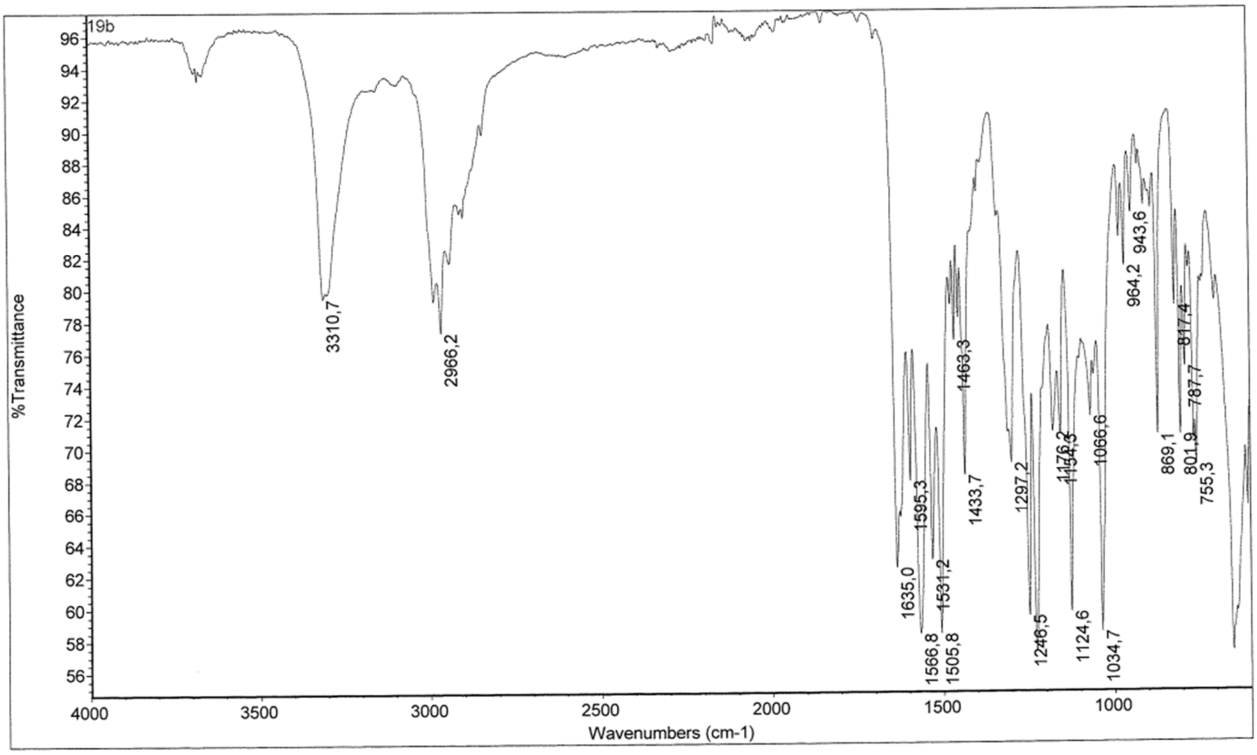

Figure S89 : IR spectrum of compound **(19b)**

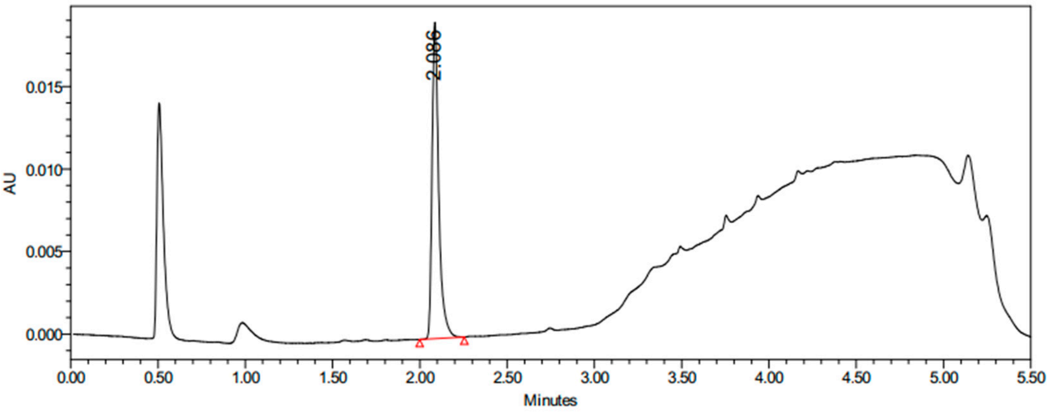

Channel: 2998; Processed Channel: 2998 PDA 241.0 nm (2998 (210-400)nm); Result Id: 3537; Processing Method: Antoine

Processed Channel Descr.: 2998 PDA 241.0 nm (2998 (210-400)nm)

	Processed Channel Descr.	RT	Area	Height	% Height
1	2998 PDA 241.0 nm (2998 (210-400)nm)	2.086	50627	19171	100.00

Figure S90 : LC chromatogram of compound **(19b)**

Compound 20b

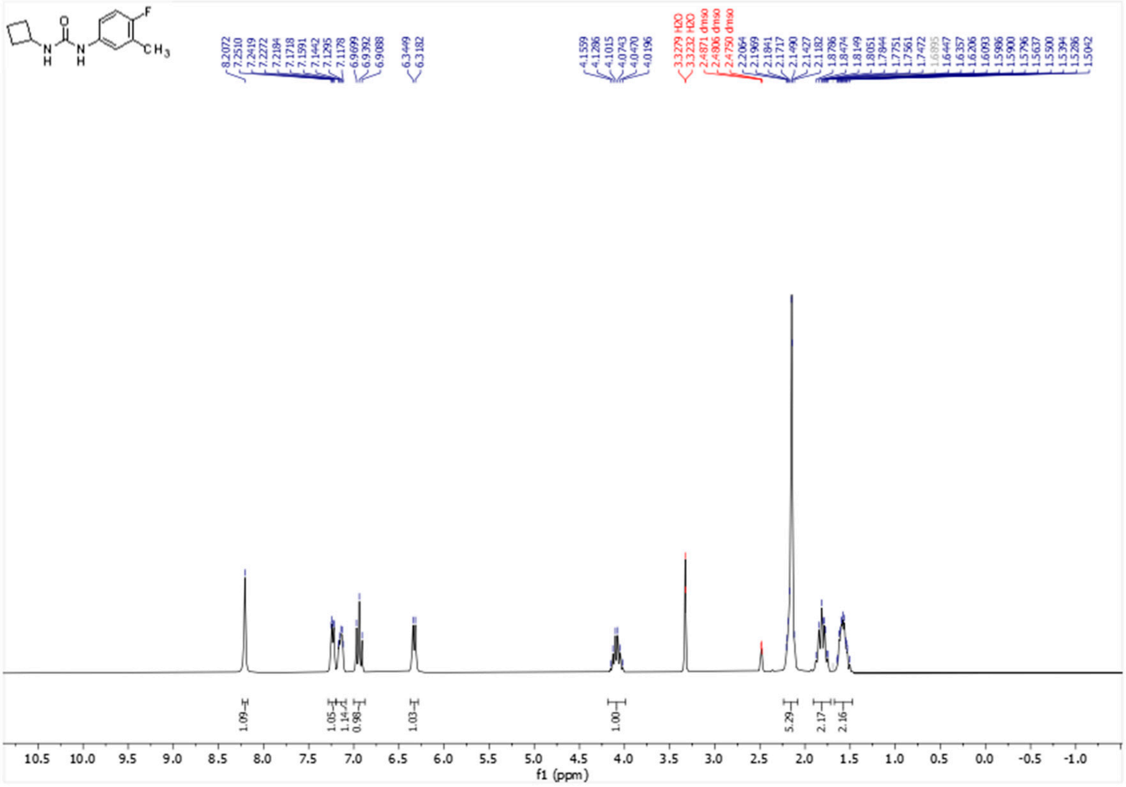

Figure S91 : ^{1}H NMR Spectrum of compound **(20b)**

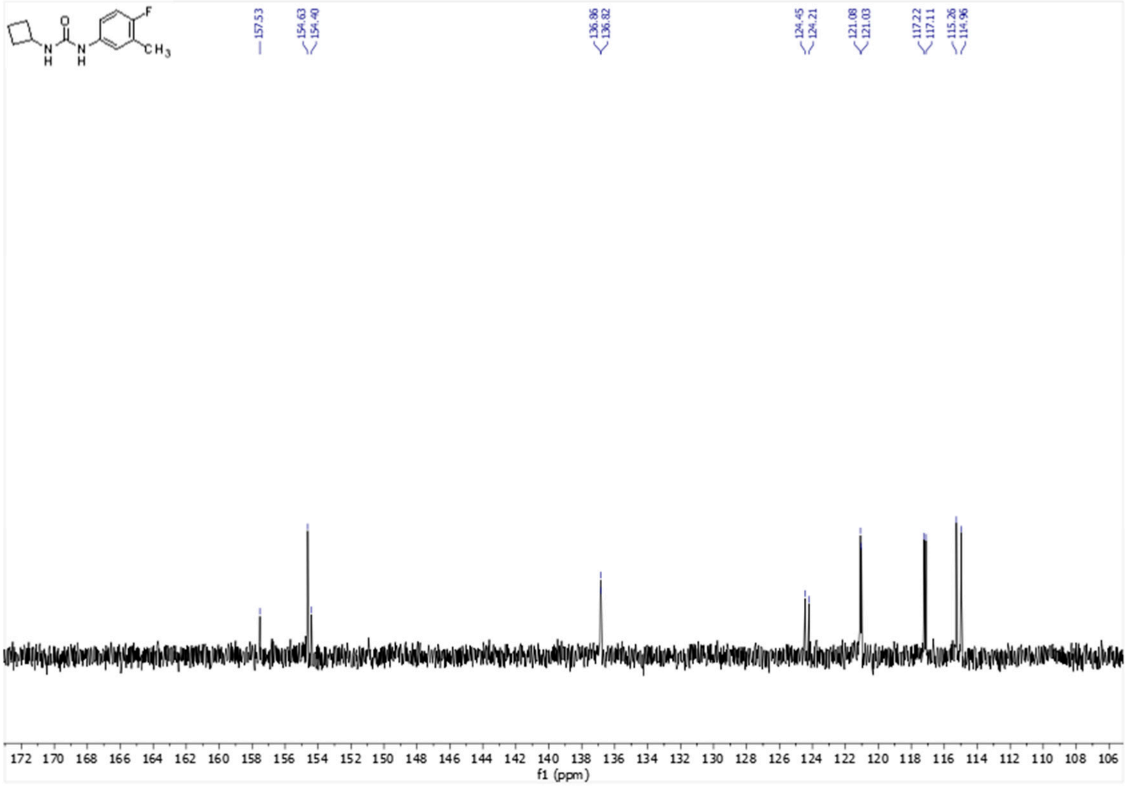

Figure S92 : ^{13}C NMR Spectrum of compound **(20b)**

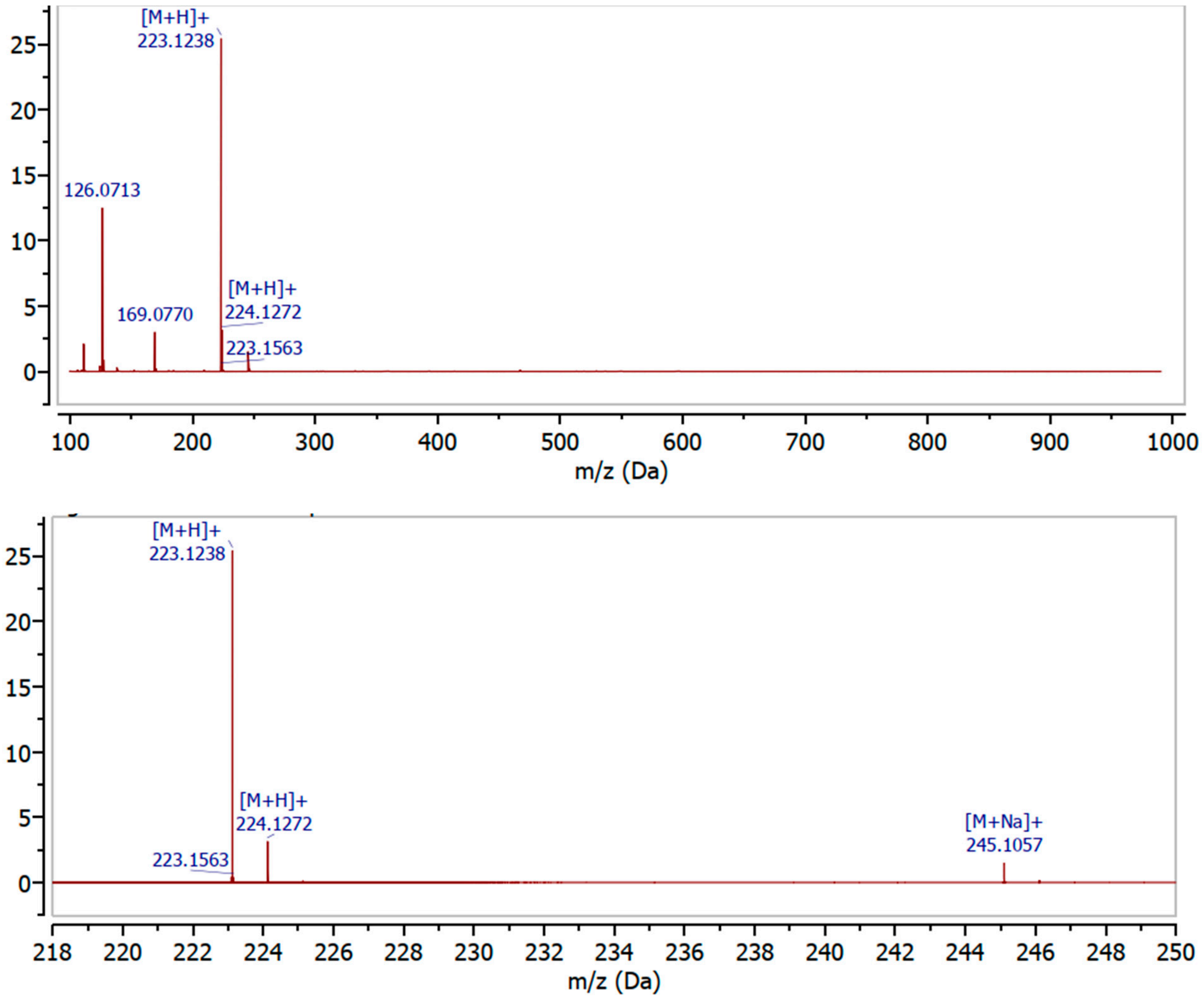

Composé trouvé: C12H15FN2O

Masse mesurée	Masse attendue	Intensité	Erreur (ppm)	Erreur (Da)	Ion identifié	Formule confirmée
223.1238	223.1241	390685529	-1.5	-0.0003	[M+H]+	C12H15FN2O
224.1272	224.1272	48763414	0.0	0.0000	[M+H]+	C12H15FN2O
245.1057	245.1061	23075253	-1.5	-0.0004	[M+Na]+	C12H15FN2O

Figure S93 : MS spectrum of compound **(20b)**

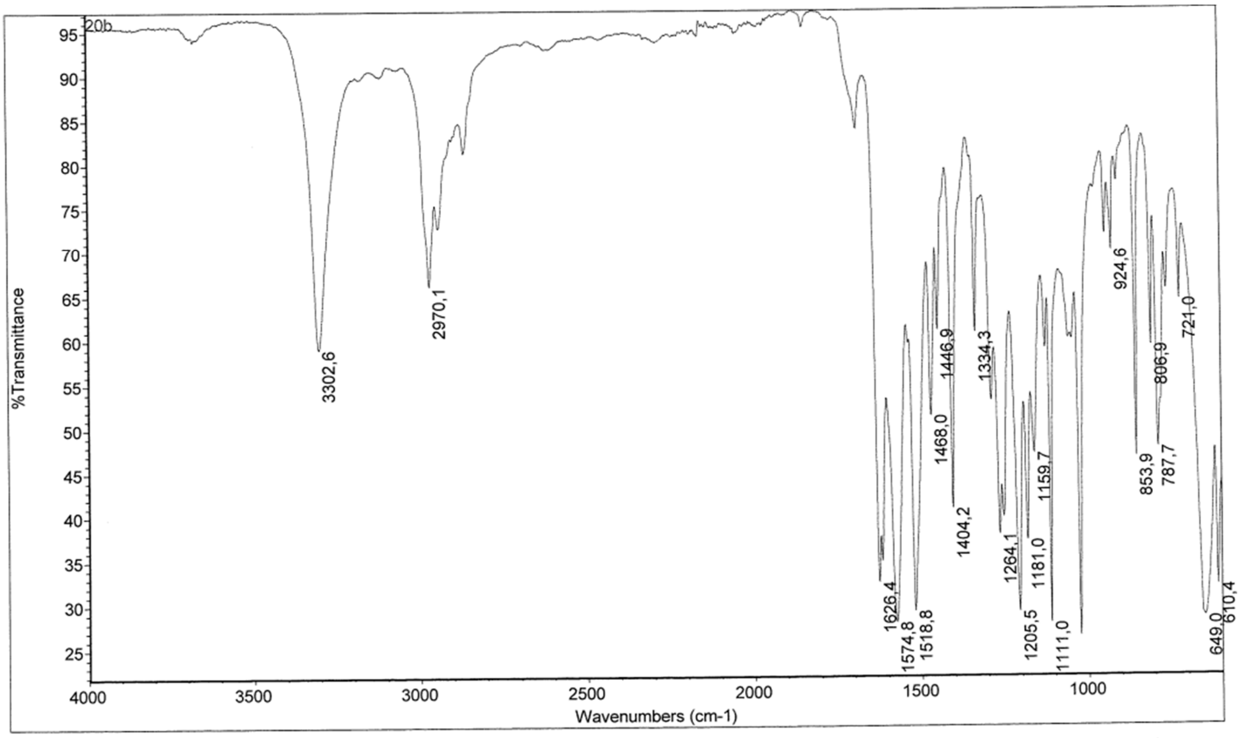

<u>Figure S94 :</u> IR spectrum of compound **(20b)**

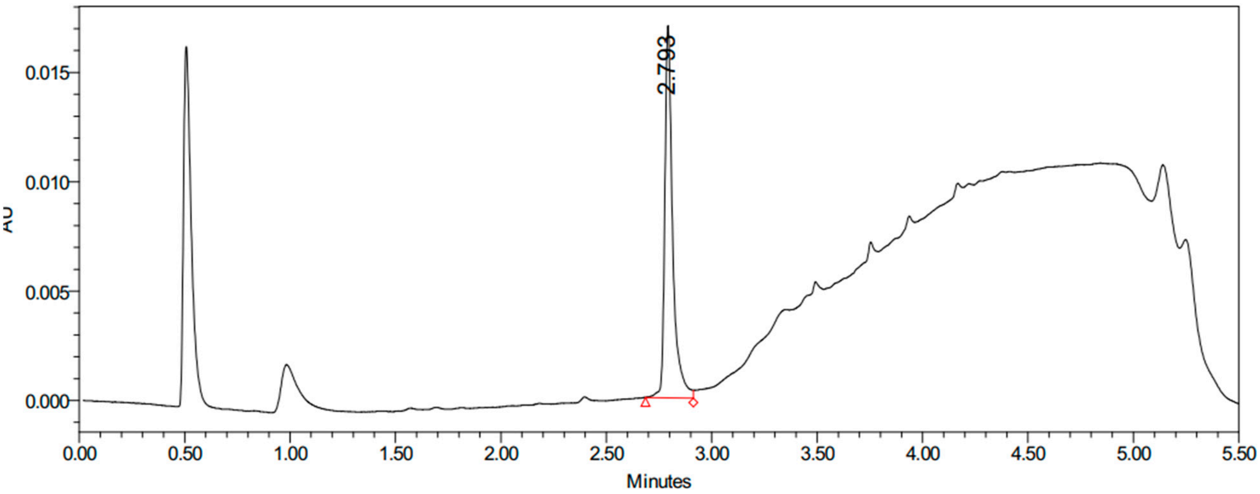

Channel: 2998; Processed Channel: 2998 PDA 241.0 nm (2998 (210-400)nm); Result Id: 3558; Processing Method: Antoine

Processed Channel Descr.: 2998 PDA 241.0 nm (2998 (210-400)nm)

	Processed Channel Descr.	RT	Area	Height	% Height
1	2998 PDA 241.0 nm (2998 (210-400)nm)	2.793	44550	17033	100.00

<u>Figure S95 :</u> LC chromatogram of compound **(20b)**

Compound 21b

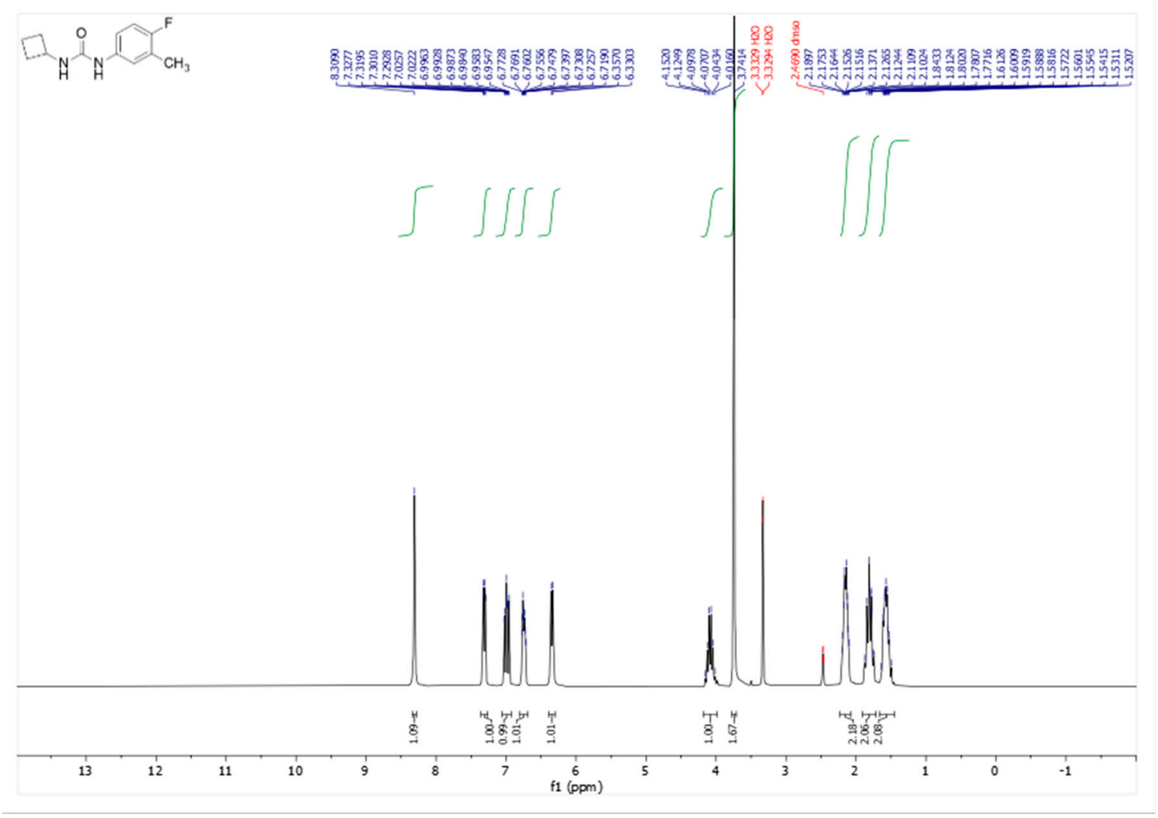

Figure S96 : ^{1}H NMR Spectrum of compound **(21b)**

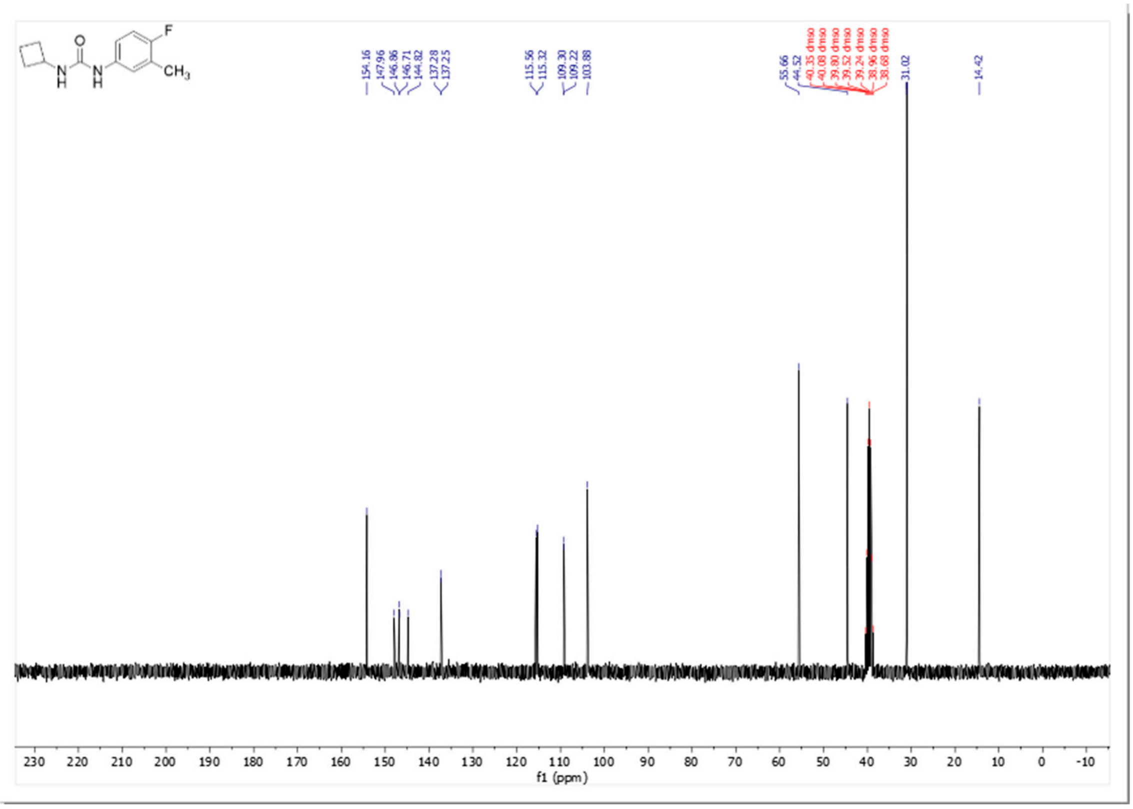

Figure S97 : ^{13}C NMR Spectrum of compound **(21b)**

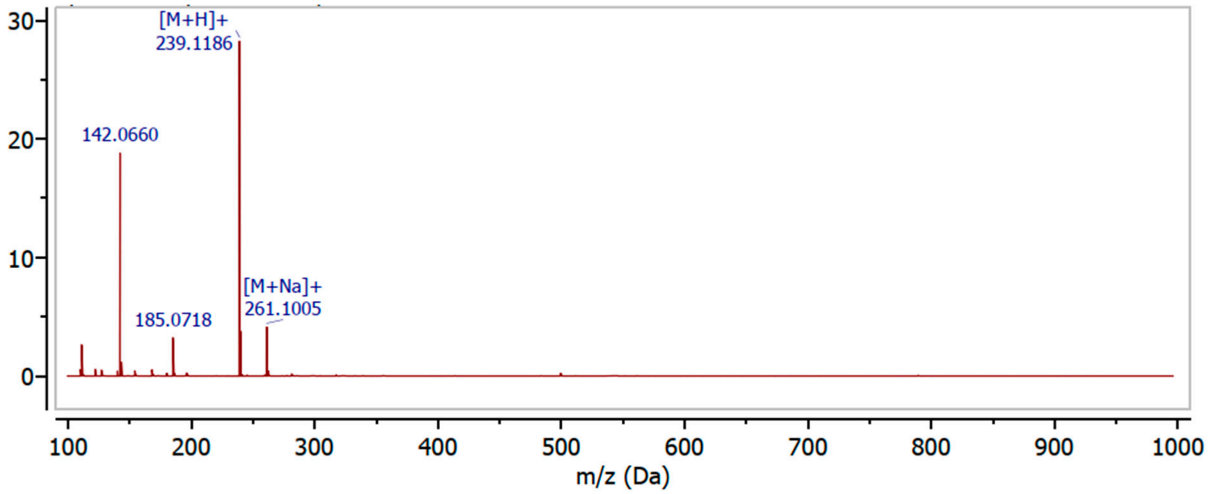

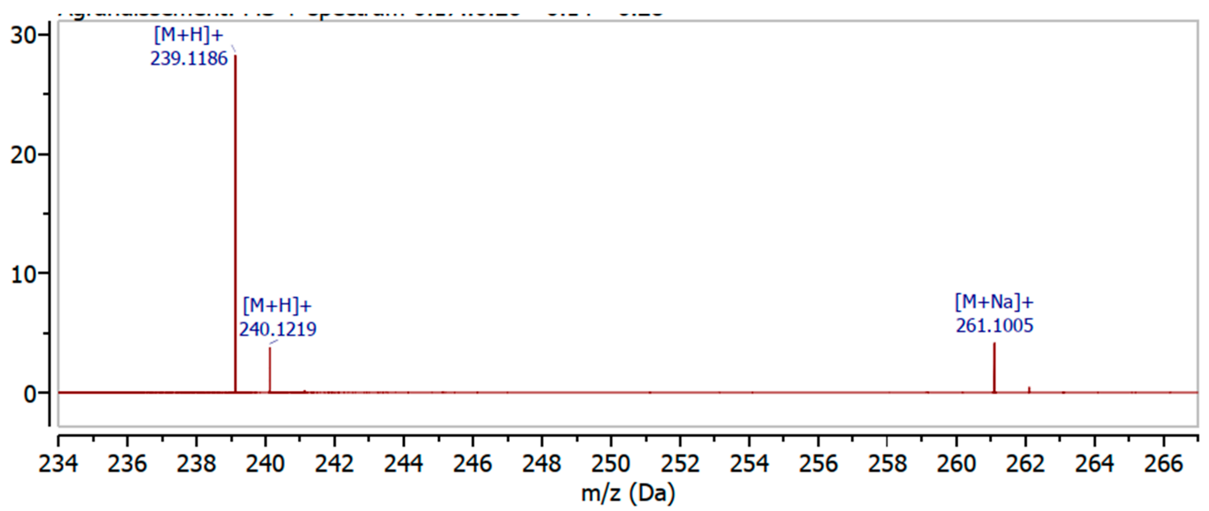

Composé trouvé: C12H15FN2O2

Masse mesurée	Masse attendue	Intensité	Erreur (ppm)	Erreur (Da)	Ion identifié	Formule confirmée
239.1186	239.1190	453153880	-2.0	-0.0005	[M+H]+	C12H15FN2O2
240.1219	240.1221	60440980	-0.8	-0.0002	[M+H]+	C12H15FN2O2
261.1005	261.1010	68605368	-1.9	-0.0005	[M+Na]+	C12H15FN2O2
262.1039	262.1040	7400087	-0.7	-0.0002	[M+Na]+	C12H15FN2O2

Figure S98 : MS spectrum of compound **(21b)**

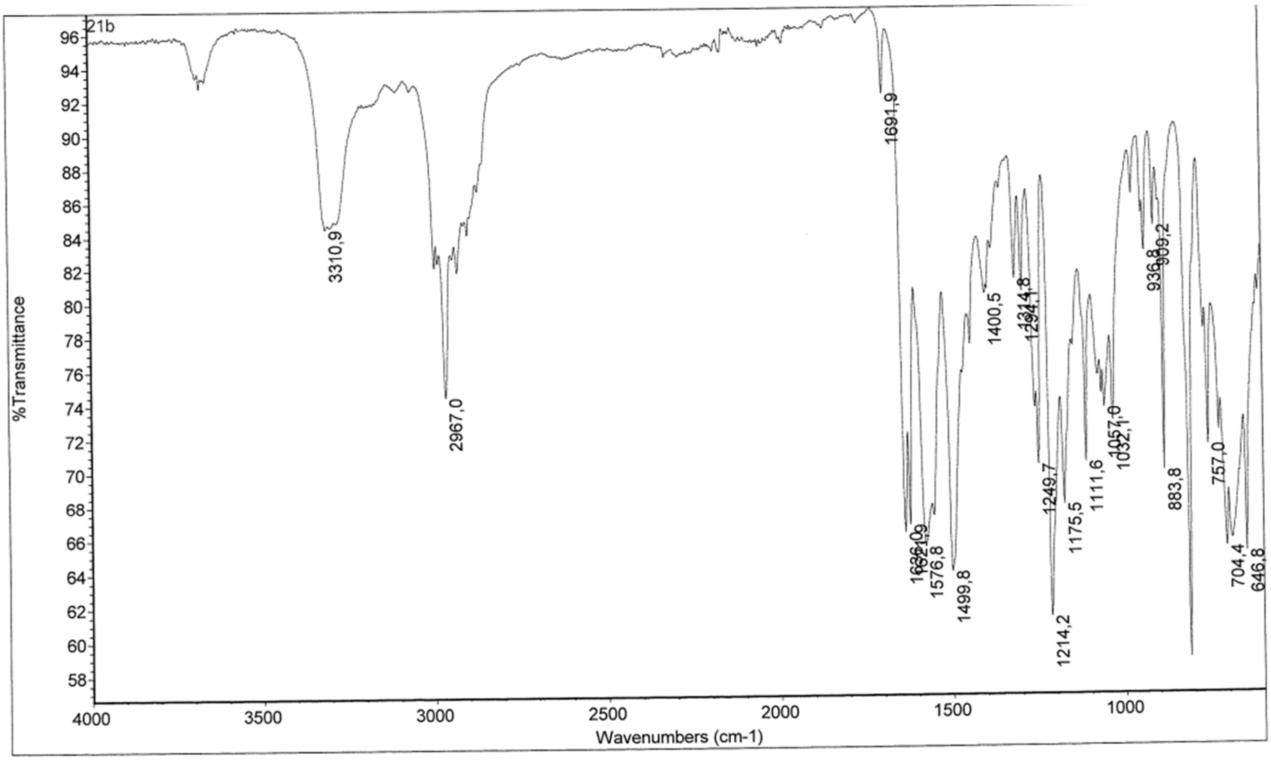

Figure S99 : IR spectrum of compound (21b)

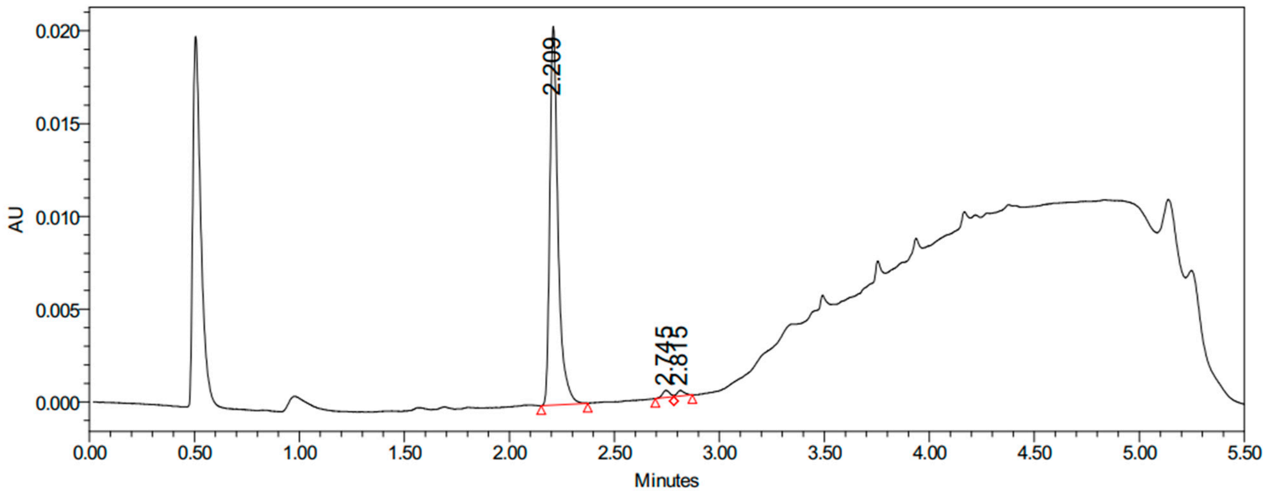

Channel: 2998; Processed Channel: 2998 PDA 241.0 nm (2998 (210-400)nm); Result Id: 3595;
Processing Method: Antoine

Processed Channel Descr.: 2998 PDA 241.0 nm (2998 (210-400)nm)

	Processed Channel Descr.	RT	Area	Height	% Height
1	2998 PDA 241.0 nm (2998 (210-400)nm)	2.209	55799	20417	96.81
2	2998 PDA 241.0 nm (2998 (210-400)nm)	2.745	882	378	1.79
3	2998 PDA 241.0 nm (2998 (210-400)nm)	2.815	715	295	1.40

Figure S100 : LC chromatogram of compound (21b)

Compound 22b

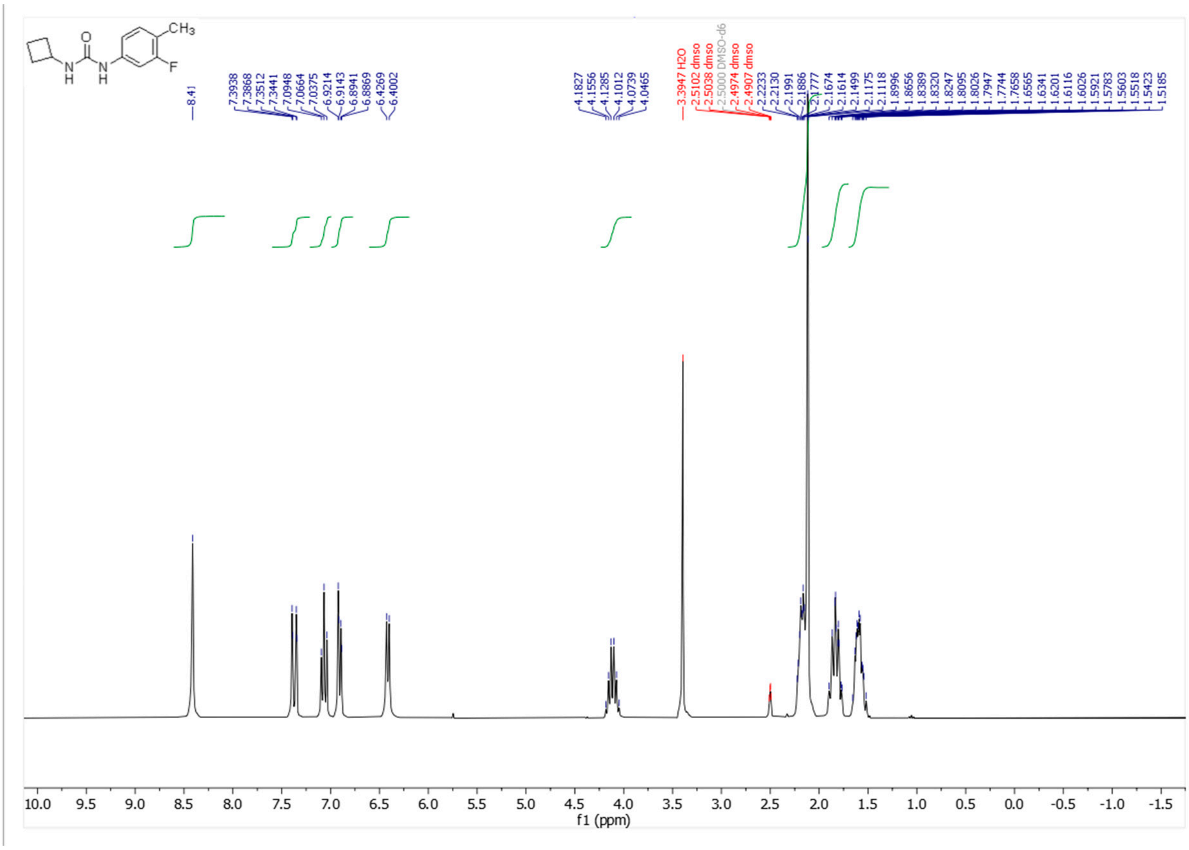

Figure S101 : ^{1}H NMR Spectrum of compound **(22b)**

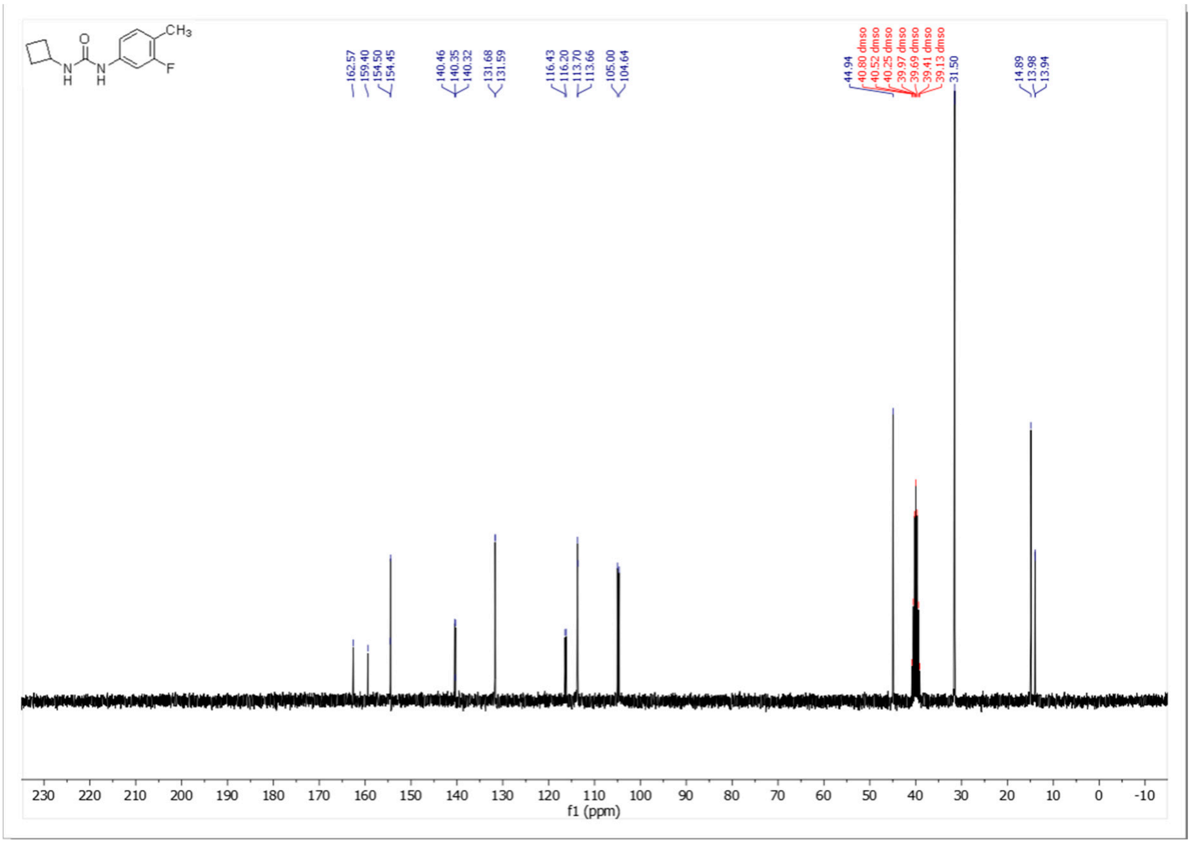

Figure S102 : ^{13}C NMR Spectrum of compound **(22b)**

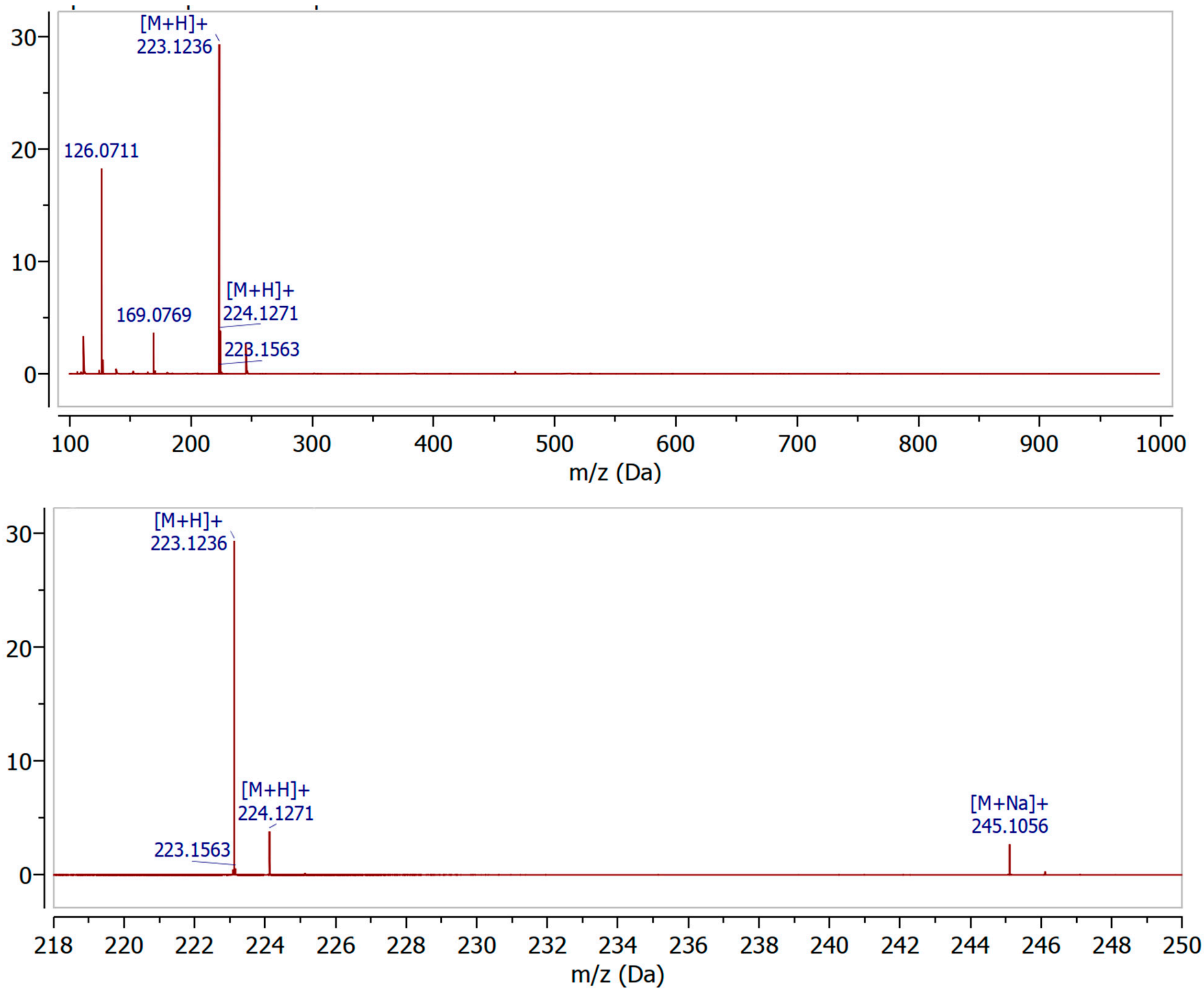

Composé trouvé: $C_{12}H_{15}FN_2O$

Masse mesurée	Masse attendue	Intensité	Erreur (ppm)	Erreur (Da)	Ion identifié	Formule confirmée
223.1236	223.1241	380300584	-2.2	-0.0005	[M+H]+	$C_{12}H_{15}FN_2O$
224.1271	224.1272	49070120	-0.6	-0.0001	[M+H]+	$C_{12}H_{15}FN_2O$
245.1056	245.1061	36426716	-1.7	-0.0004	[M+Na]+	$C_{12}H_{15}FN_2O$

Figure 103: MS spectrum of compound **(22b)**

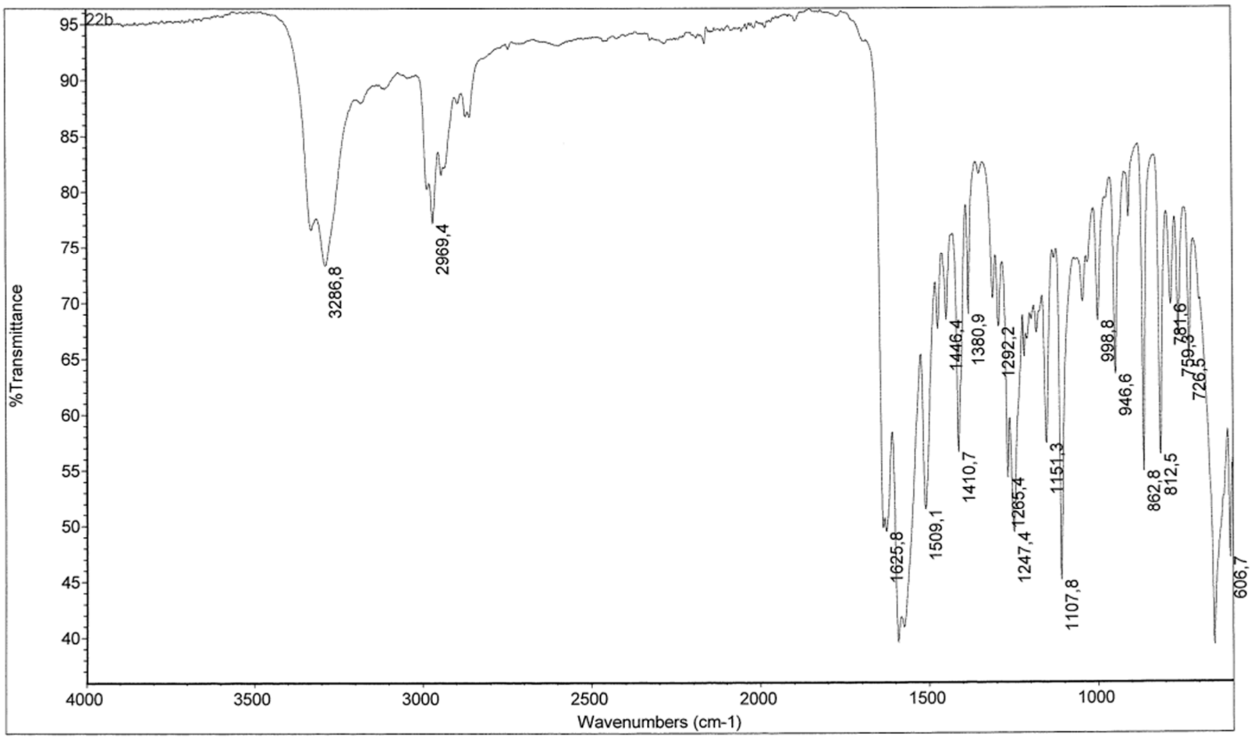

Figure S104 : IR spectrum of compound **(22b)**

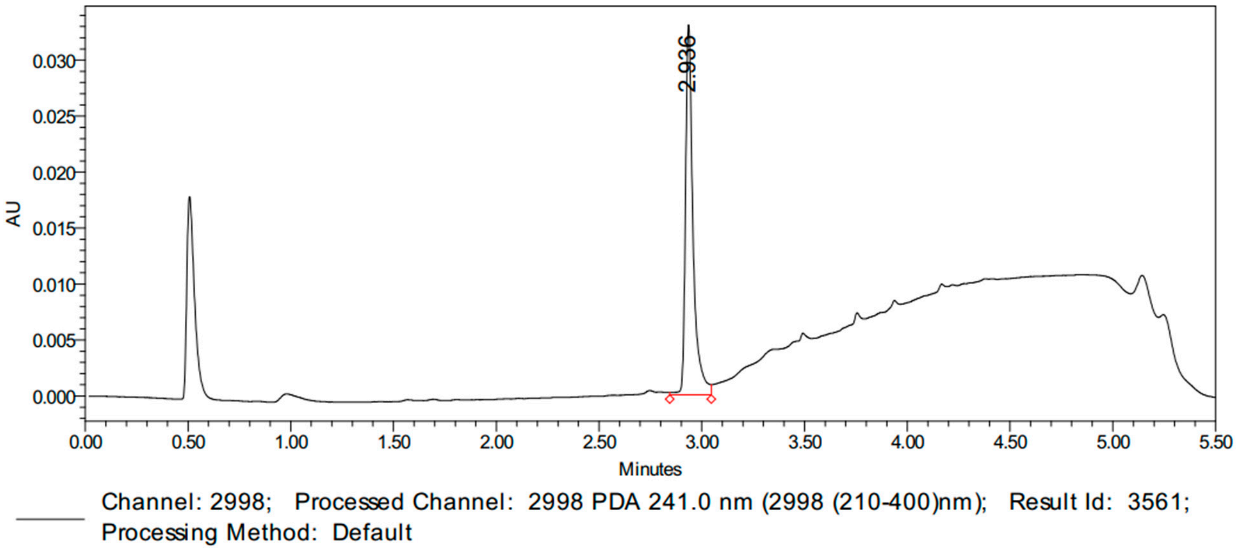

Processed Channel Descr.: 2998 PDA 241.0 nm (2998 (210-400)nm)

	Processed Channel Descr.	RT	Area	Height	% Height
1	2998 PDA 241.0 nm (2998 (210-400)nm)	2.936	83771	33082	100.00

Figure S105 : LC chromatogram of compound **(22b)**

Compound 23b

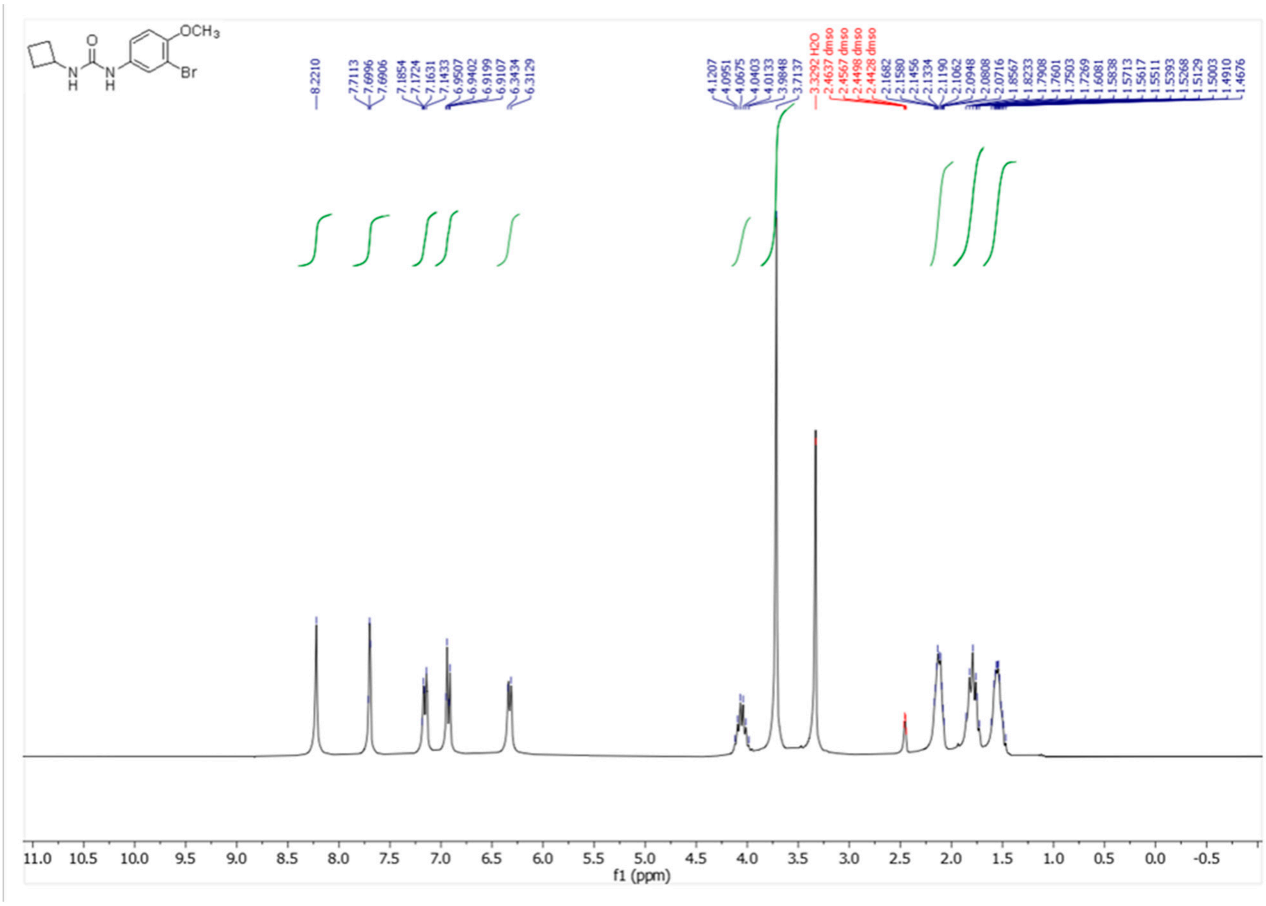

Figure S106 : ^{1}H NMR Spectrum of compound **(23b)**

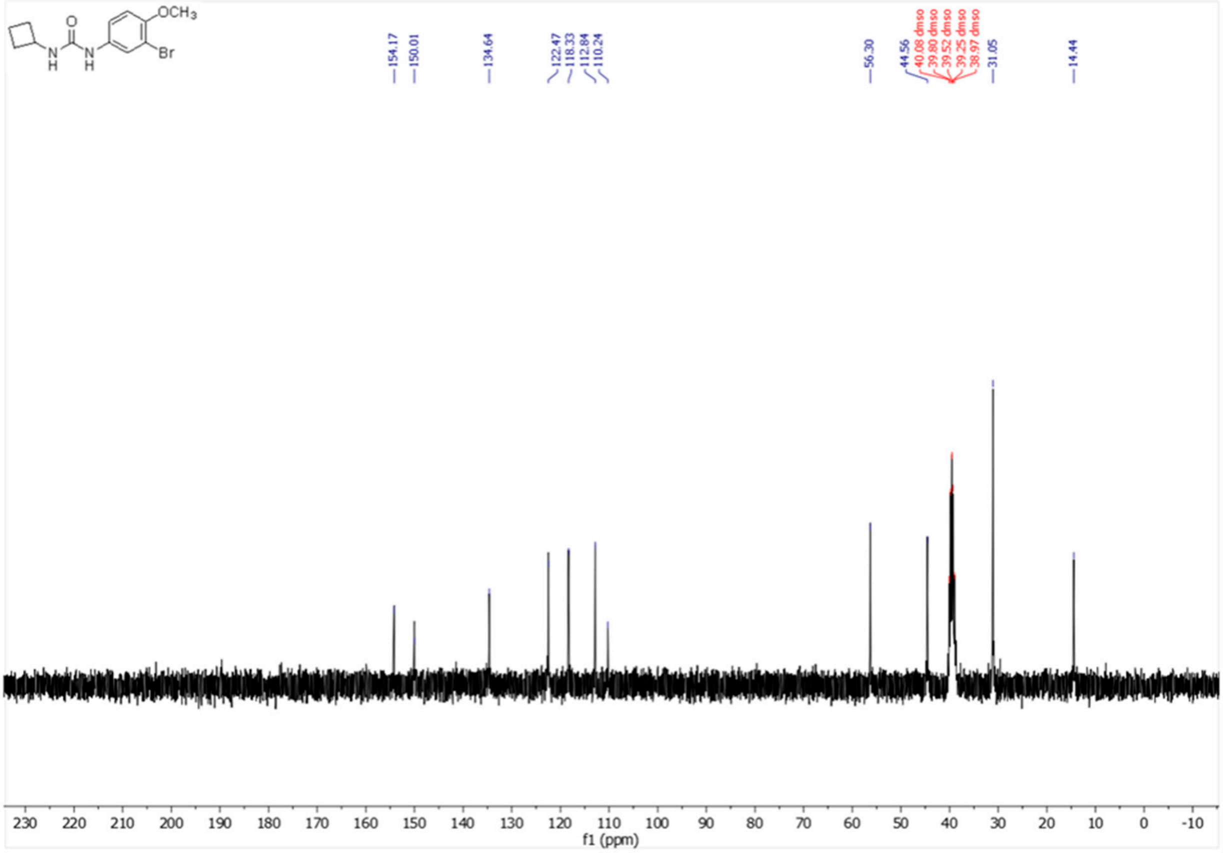

Figure S107 : ^{13}C NMR Spectrum of compound **(23b)**

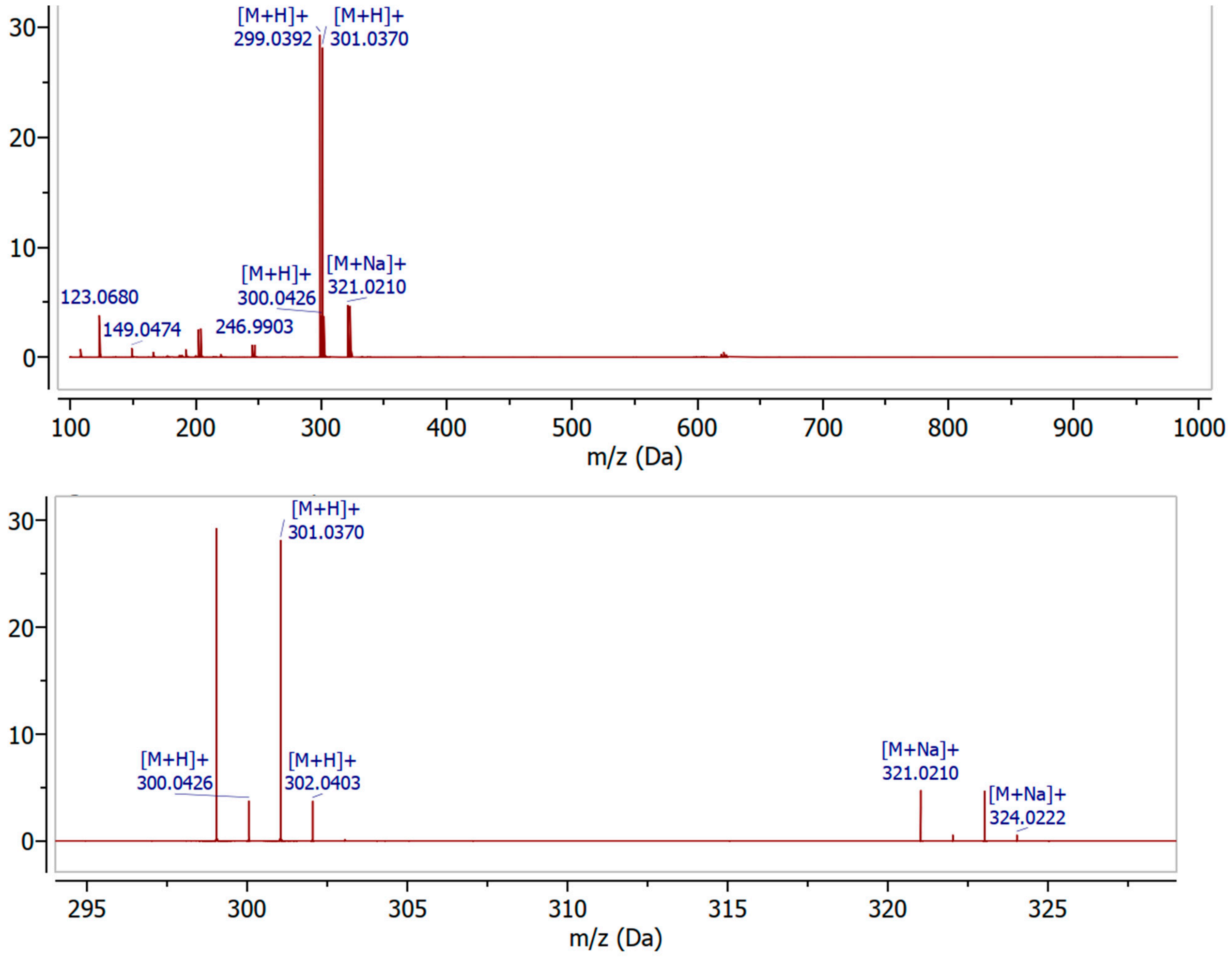

Composé trouvé: C12H15BrN2O2

Masse mesurée	Masse attendue	Intensité	Erreur (ppm)	Erreur (Da)	Ion identifié	Formule confirmée
299.0392	299.0390	181313014	0.8	0.0002	[M+H]+	C12H15BrN2O2
300.0426	300.0420	23501323	1.8	0.0005	[M+H]+	C12H15BrN2O2
301.0370	301.0370	178332909	-0.2	-0.0001	[M+H]+	C12H15BrN2O2
302.0403	302.0400	22751018	0.9	0.0003	[M+H]+	C12H15BrN2O2
321.0210	321.0209	30088259	0.4	0.0001	[M+Na]+	C12H15BrN2O2
322.0244	322.0240	3520379	1.2	0.0004	[M+Na]+	C12H15BrN2O2
323.0190	323.0190	28481885	0.1	0.0000	[M+Na]+	C12H15BrN2O2
324.0222	324.0220	3475174	0.8	0.0003	[M+Na]+	C12H15BrN2O2

<u>Figure 108:</u> MS spectrum of compound **(23b)**

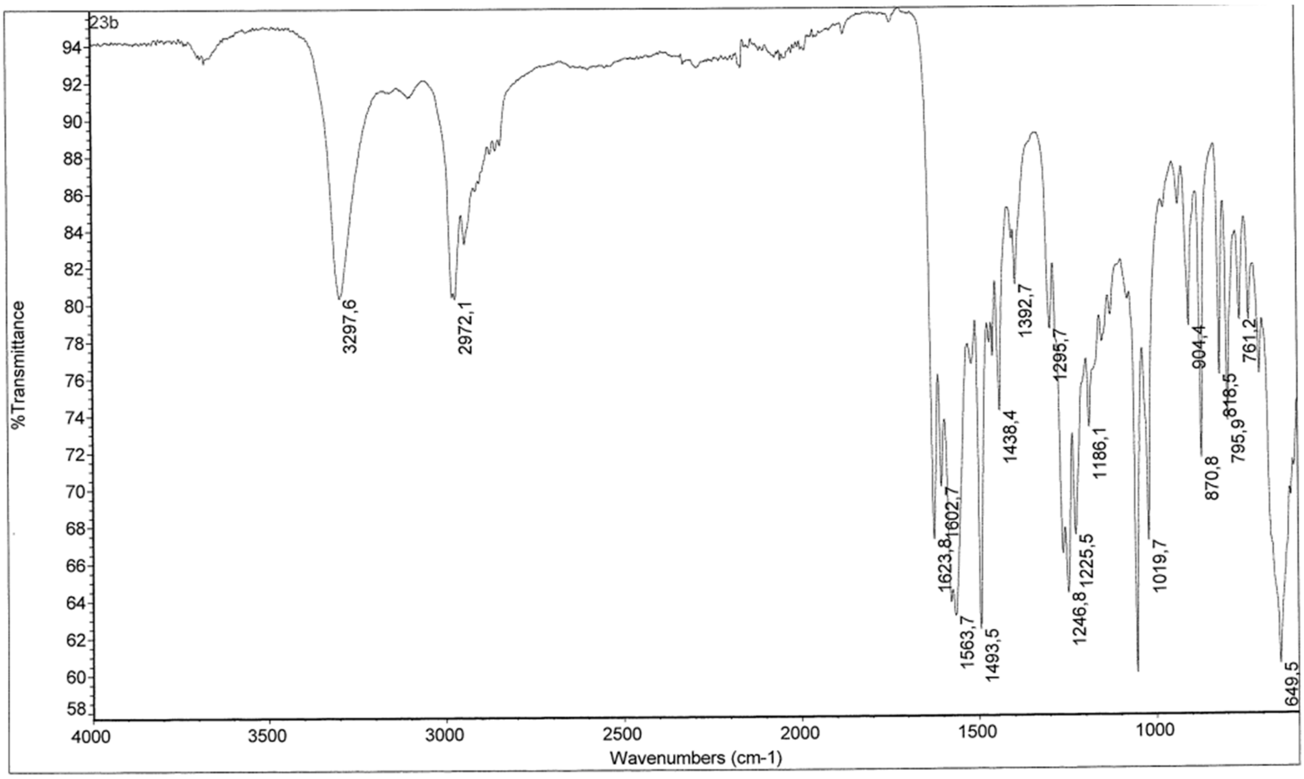

Figure S109 : IR spectrum of compound **(23b)**

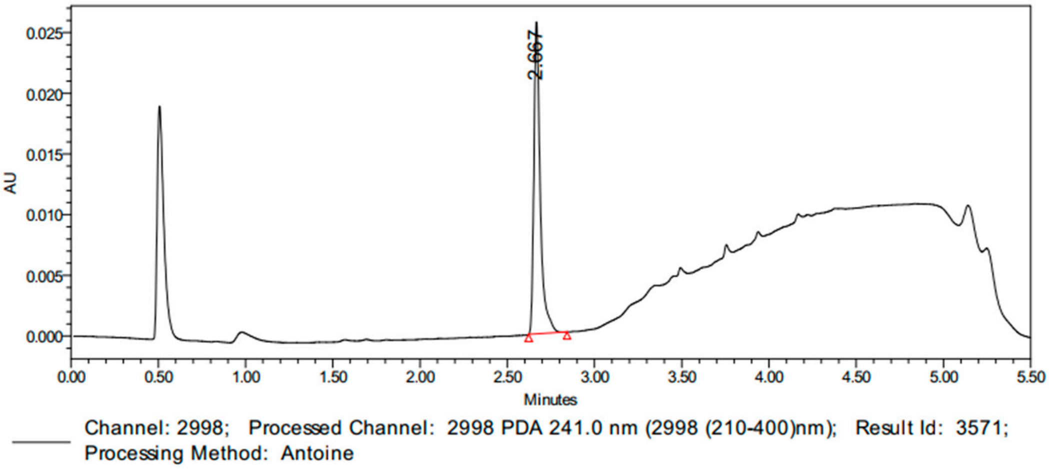

Processed Channel Descr.: 2998 PDA 241.0 nm (2998 (210-400)nm)

	Processed Channel Descr.	RT	Area	Height	% Height
1	2998 PDA 241.0 nm (2998 (210-400)nm)	2.667	64913	25737	100.00

Figure S110 : LC chromatogram of compound **(23b)**

Compound 24b

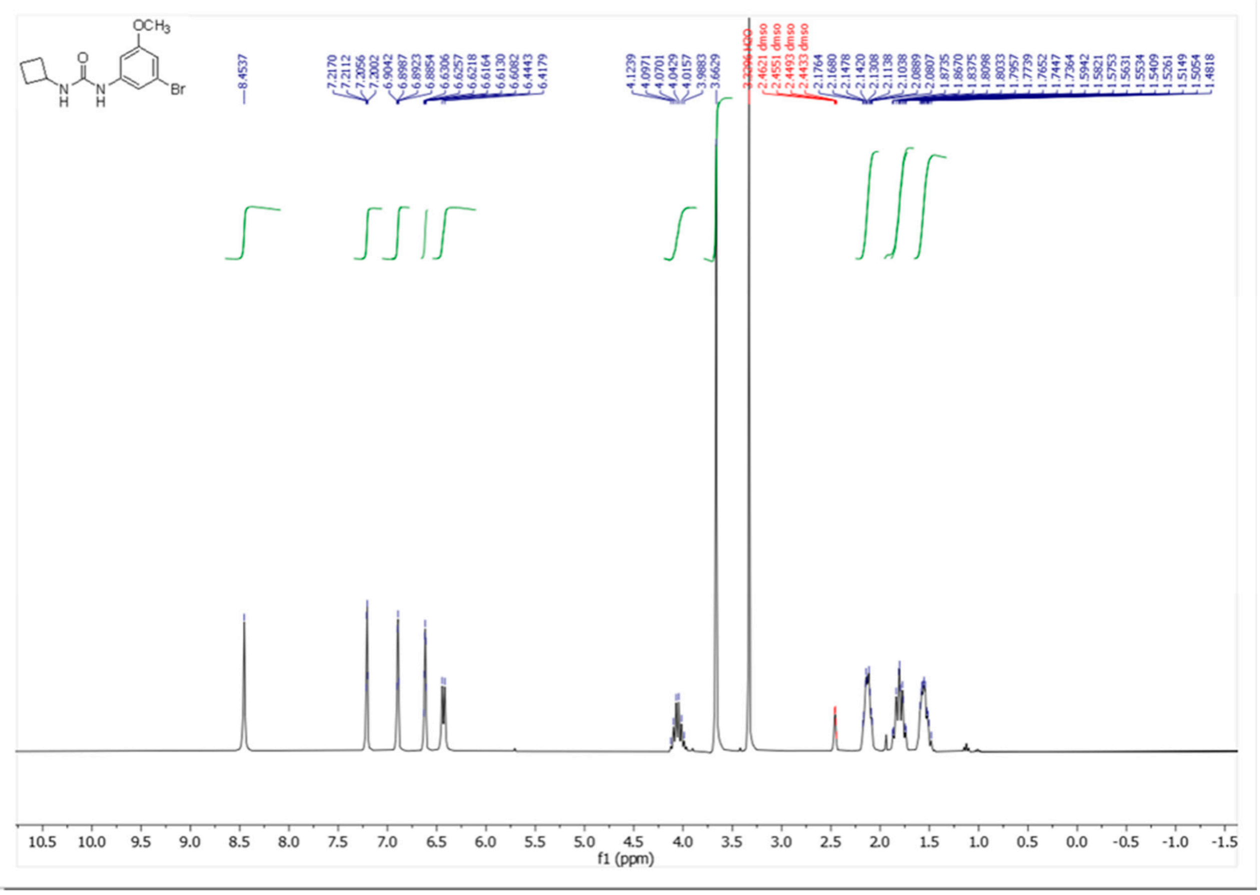

Figure S111 : ^{1}H NMR Spectrum of compound **(24b)**

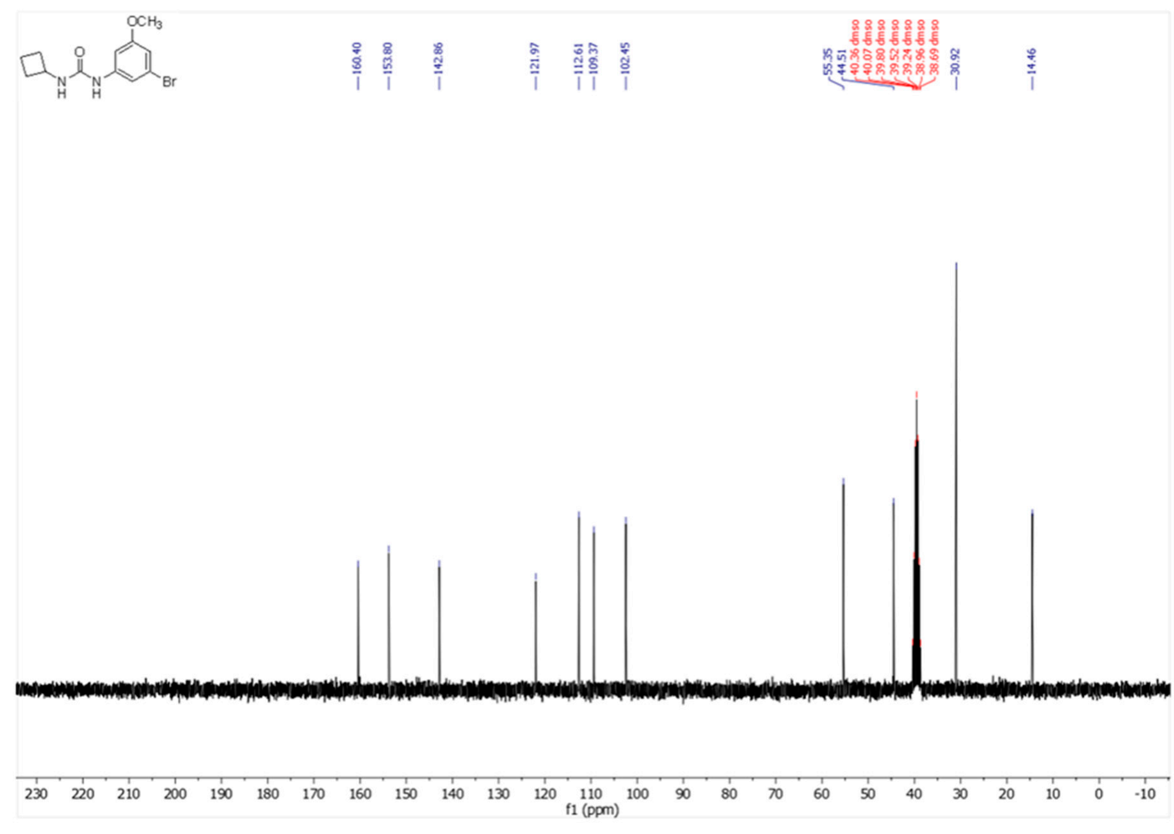

Figure S112 : ^{13}C NMR Spectrum of compound **(24b)**

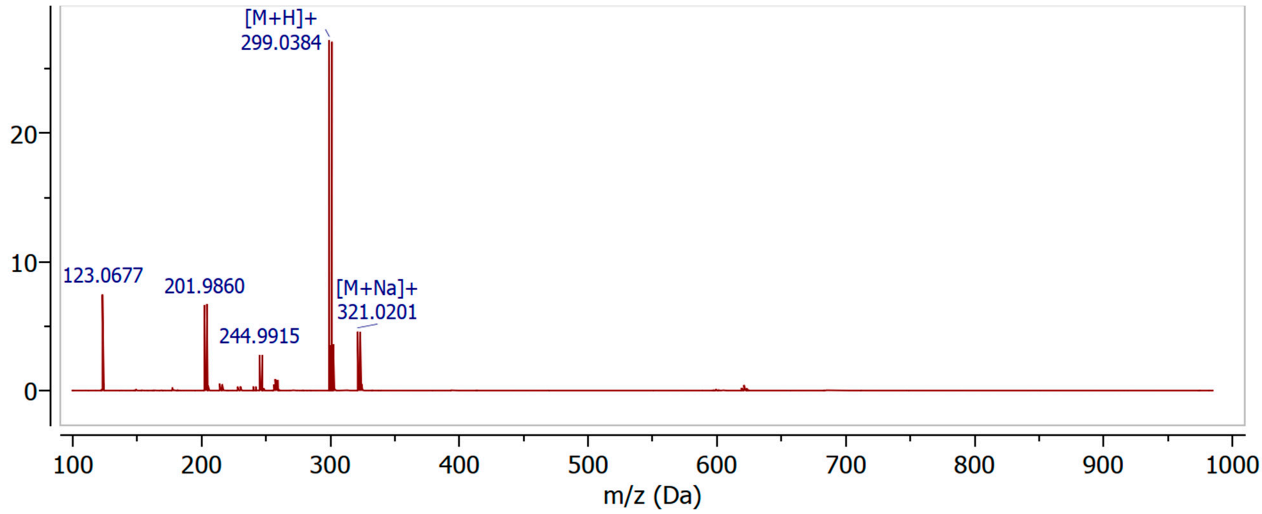

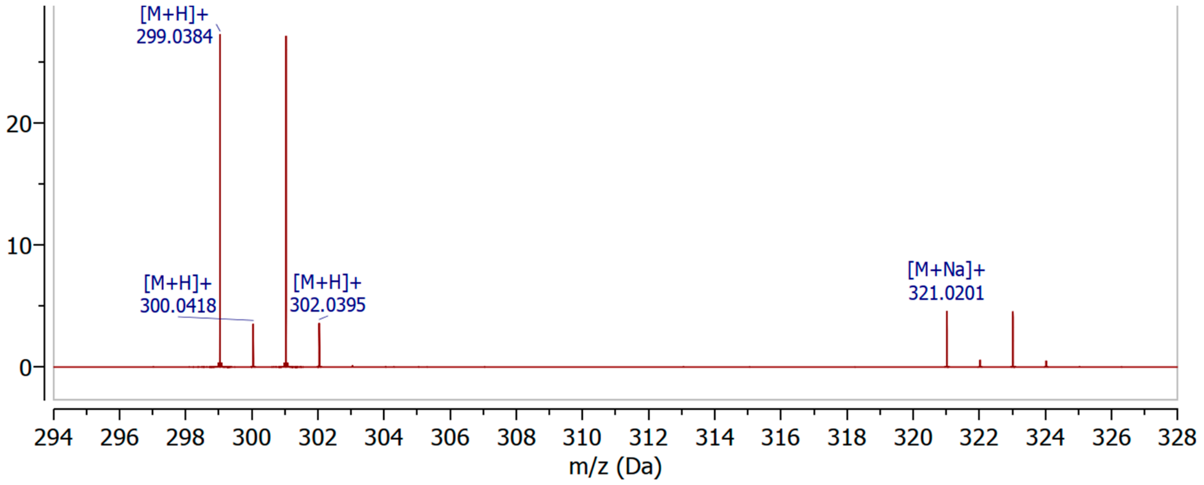

Composé trouvé: C12H15BrN2O2

Masse mesurée	Masse attendue	Intensité	Erreur (ppm)	Erreur (Da)	Ion identifié	Formule confirmée
299.0384	299.0390	177737605	-1.9	-0.0006	[M+H]+	C12H15BrN2O2
300.0418	300.0420	23002150	-0.9	-0.0003	[M+H]+	C12H15BrN2O2
301.0362	301.0370	174368209	-2.8	-0.0009	[M+H]+	C12H15BrN2O2
302.0395	302.0400	22477331	-1.8	-0.0005	[M+H]+	C12H15BrN2O2
321.0201	321.0209	29426040	-2.4	-0.0008	[M+Na]+	C12H15BrN2O2
323.0180	323.0190	28418169	-2.9	-0.0009	[M+Na]+	C12H15BrN2O2

Figure 113: MS spectrum of compound **(24b)**

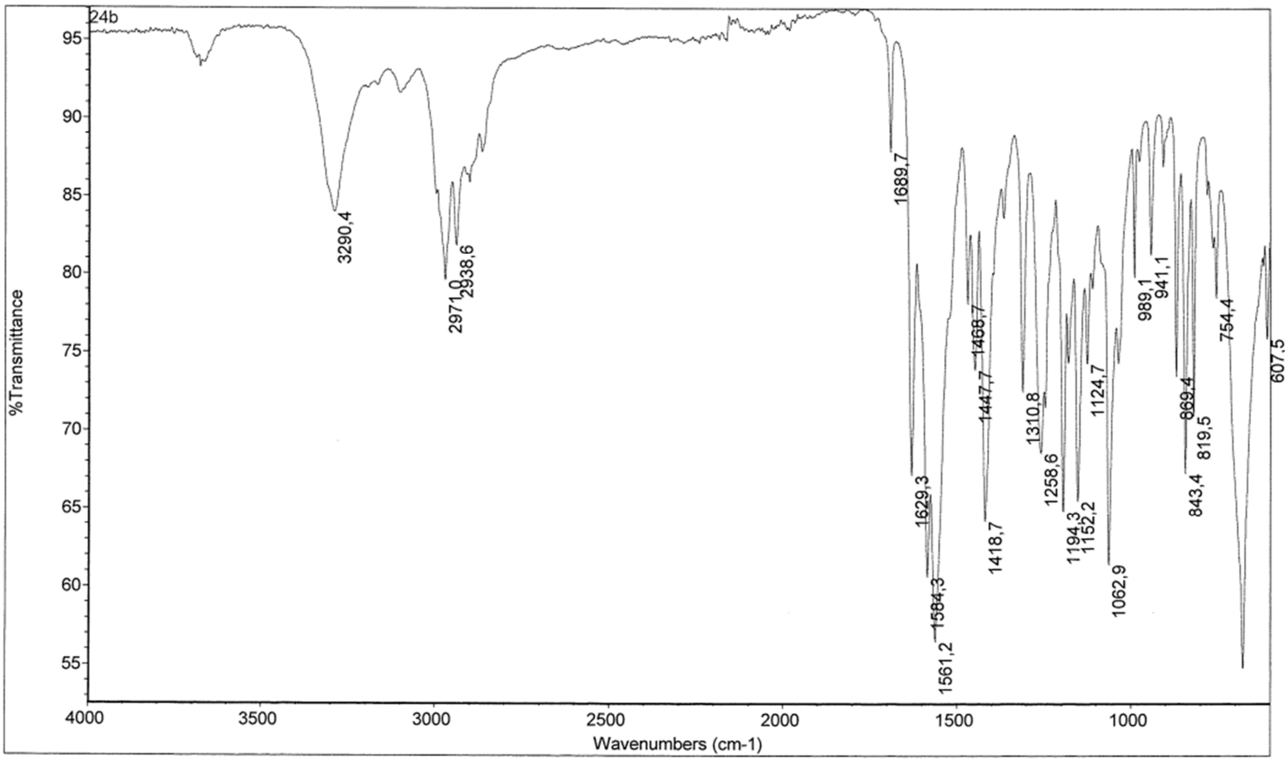

Figure S114 : IR spectrum of compound **(24b)**

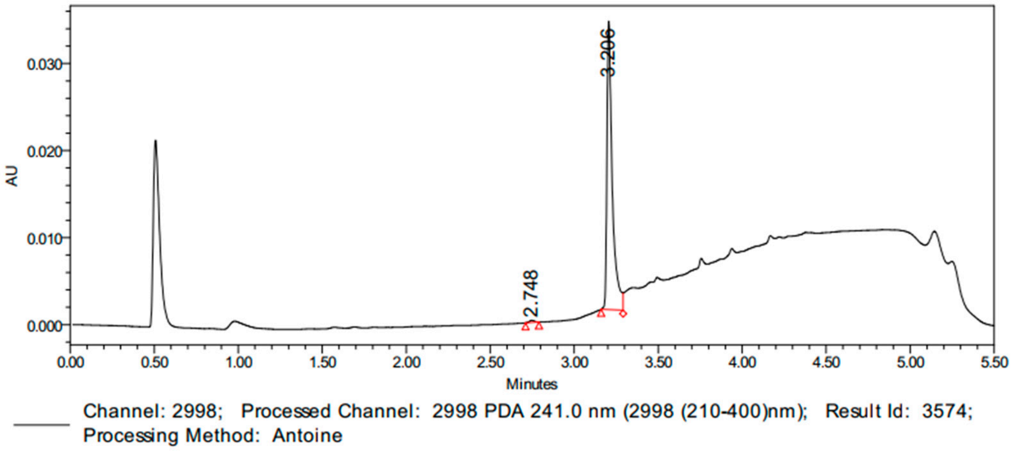

Processed Channel Descr.: 2998 PDA 241.0 nm (2998 (210-400)nm)

	Processed Channel Descr.	RT	Area	Height	% Height
1	2998 PDA 241.0 nm (2998 (210-400)nm)	2.748	520	238	0.71
2	2998 PDA 241.0 nm (2998 (210-400)nm)	3.206	72883	33191	99.29

Figure S115 : LC chromatogram of compound **(24b)**

Table S1 : AutoDock Vina docking scores (kcal/mol) for TPPU, *t*-AUCB, and compounds **4b**, **10b**, and **16b** in the human sEH epoxide hydrolase (EH) pocket (PDB: 3I28). For each ligand, the four top-ranked poses (Pose Rank 1-4) are reported; more negative values indicate more favorable predicted binding.

	Epoxyde hydrolase cavity	
**Compound**	**Pose Rank**	**Vina score Kcal/mol**
**TPPU**	1	-9.5
	2	-9.1
	3	-9
	4	-8.7
***t*-AUCB**	1	-10.3
	2	-9.5
	3	-9.2
	4	-9
**4b**	1	-5.8
	2	-5.4
	3	-5.3
	4	-5.2
**10b**	1	-7
	2	-6.4
	3	-5.7
	4	-4.9
**16b**	1	-6.3
	2	-6.2
	3	-5.8
	4	-5.2

Table S2 : Docking-derived energetic differences ($\Delta\Delta G$, kcal/mol) and estimated fold differences relative to the reference inhibitors TPPU and *t*-AUCB in the sEH EH pocket. $\Delta\Delta G$ values were computed for each pose of **4b**, **10b**, and **16b** using the corresponding Vina score from Table 1 and the top-ranked reference scores (TPPU: -9.5 kcal/mol; t-AUCB: -10.3 kcal/mol). Fold differences are reported as Kd_{test}/Kd_{ref}.

T (K)	RT (kcal/mol)
298	0.592126

Compound	ΔΔG vs TPPU (kcal/mol)	ΔΔG vs *t*-AUCB (kcal/mol)	Fold vs TPPU	Fold vs *t*-AUCB
**4b**	3.7	4.5	517.3	1997.7
	4.1	4.9	1016.6	3925.6
	4.2	5.0	1203.6	4647.8
	4.3	5.1	1425.1	5502.9
**10b**	2.5	3.3	68.2	263.3
	3.1	3.9	187.8	725.2
	3.8	4.6	612.5	2365.2
	4.6	5.4	2365.2	9133.3
**16b**	3.2	4.0	222.4	858.6
	3.3	4.1	263.3	1016.6
	3.7	4.5	517.3	1997.7
	4.3	5.1	1425.1	5502.9
